# Supplementary material for: ER-phagy restrains inflammatory responses through its receptor UBAC2
Source: EMBO J. 2024 Sep 16;43(21):13. doi: 10.1038/s44318-024-00232-z (PMC11535055; doi:10.1038/s44318-024-00232-z)
Supplement: Supplementary file 1 — Appendix [file 44318_2024_232_MOESM1_ESM.pdf]

Appendix for

ER-phagy restrains inflammatory responses through its receptor UBAC2

Table of Contents

Appendix Figure S1 page 2

Appendix Figure S2 page 3

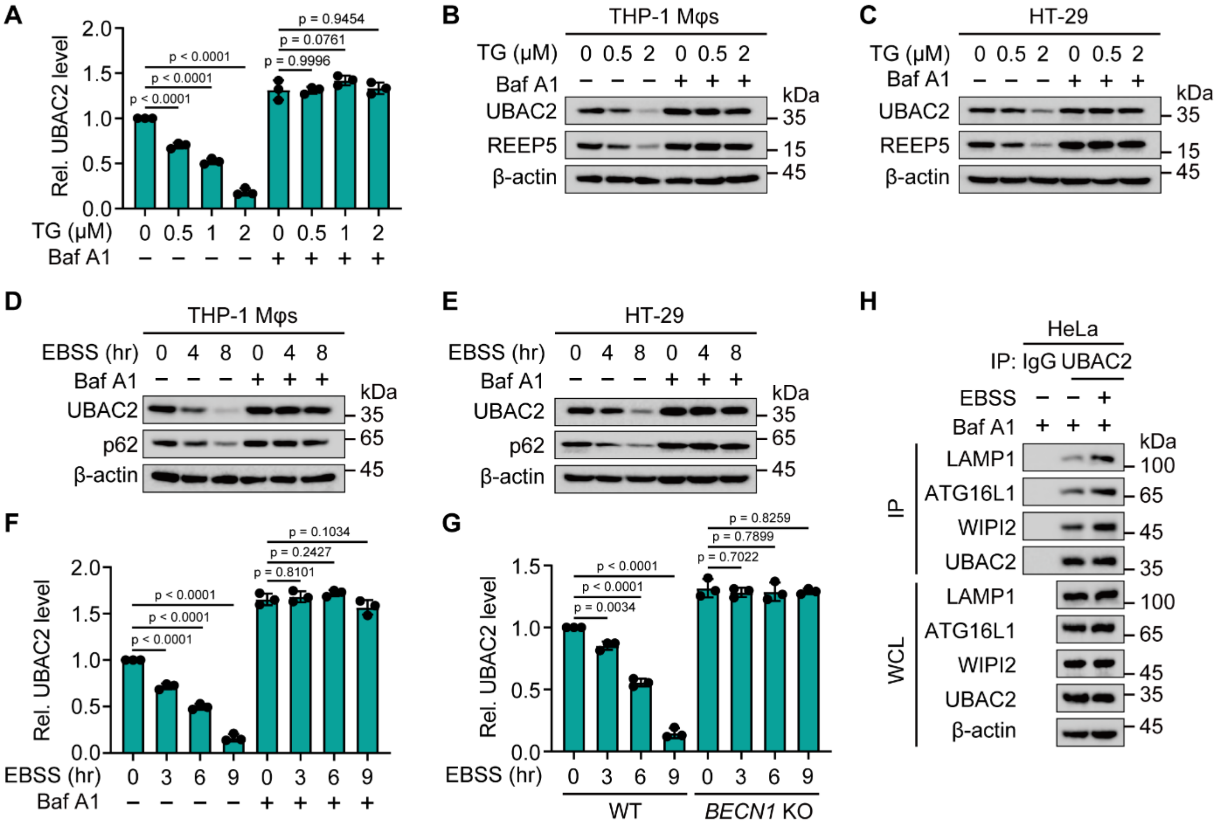

Appendix Fig S1. UBAC2 undergoes degradation in autophagy.

(**A**) The quantification of UBAC2 protein abundance from Fig. 1F. (**B**, **C**) THP-1-derived macrophages (THP-1 Mφs) (**B**) or HT-29 cells (**C**) were treated with indicated dosages of TG in the absence or presence of Baf A1 (0.2 μM), and the lysates were detected by immunoblot. (**D**, **E**) THP-1 Mφs (**D**) or HT-29 cells (**E**) were cultured in EBSS for indicated time points with or without Baf A1 (0.2 μM) treatment, and the lysates were detected by immunoblot. (**F**) The quantification of UBAC2 protein abundance in similar samples as Fig. 1H from three biologically independent experiments. (**G**) The quantification of UBAC2 protein abundance in similar samples as Fig. 1I from three biologically independent experiments. (**H**) Extracts of HeLa cells cultured in EBSS for 3 hr with Baf A1 (0.2 μM) treatment were subjected to immunoprecipitation with anti-UBAC2 and immunoblot analysis with indicated antibodies. Data information: For (**B**, **C**, **E**, **F**, **H**), one representative experiment out of three was shown. In (**A**, **D**, **G**), data are presented as the mean ± SEM of three independent biological experiments. The statistical significance of the difference was analyzed by unpaired two-tailed Student's *t* test, and the *P* values were shown.

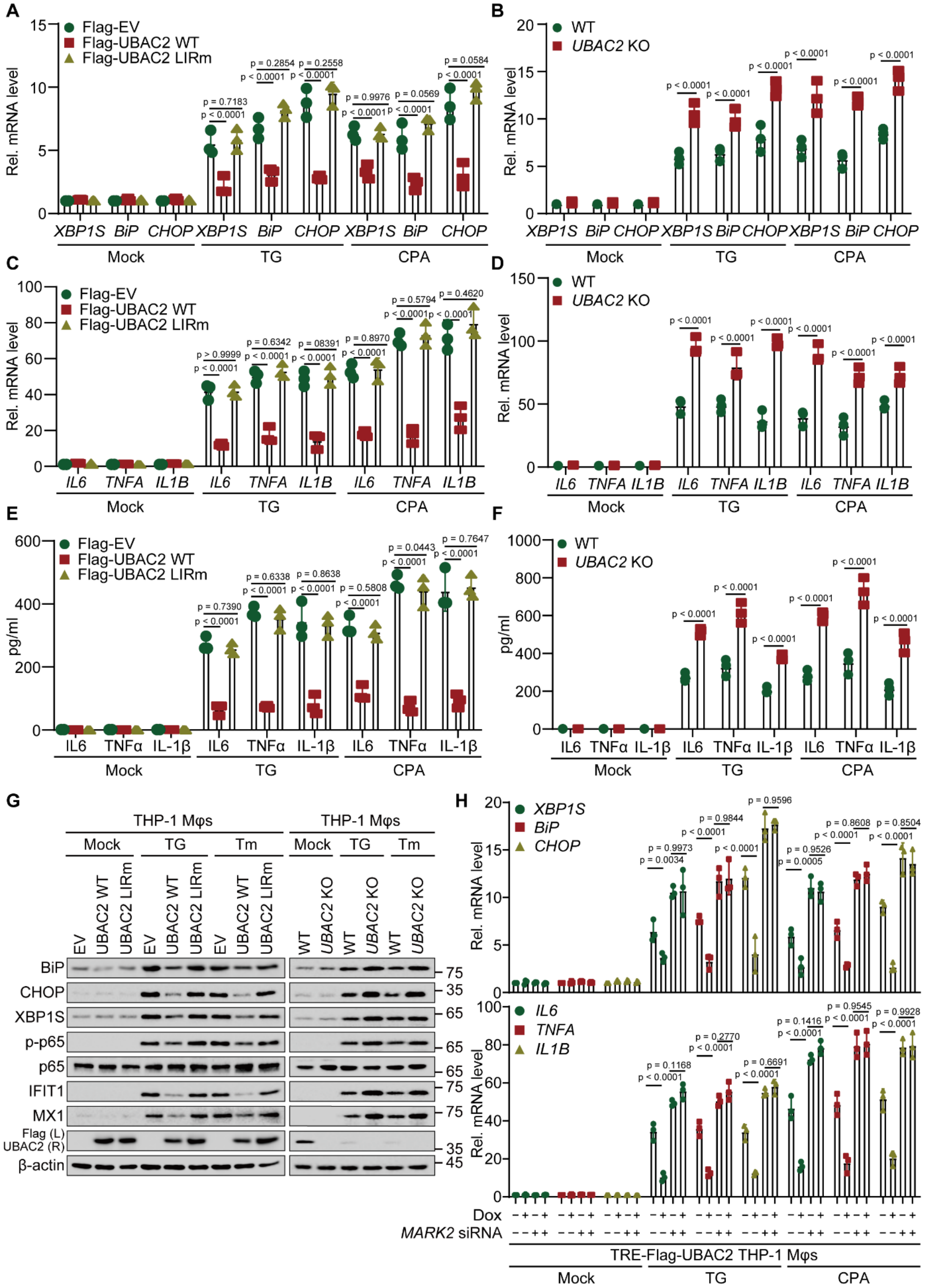

Appendix Fig S2. UBAC2-mediated ER-phagy suppresses inflammatory responses.

(**A**) qPCR analysis of ER-stress inducible transcripts in WT or LIRm UBAC2 inducible THP-

1-derived macrophages (THP-1 Mφs) were incubated with Dox (200 ng/mL) for overnight and treated with TG (1 μM) or CPA (10 μM) for 6 hr. (**B**) qPCR analysis of ER-stress inducible transcripts in WT or *UBAC2* knockout (KO) THP-1 cells with TG (1 μM) or CPA (10 μM) treatment for 6 hr. (**C**) qPCR analysis of expression of inflammatory genes in WT or LIRm UBAC2 inducible THP-1 Mφs were incubated with Dox (200 ng/mL) for overnight and treated with TG (1 μM) or CPA (10 μM) for 12 hr. (**D**) qPCR analysis of expression of inflammatory genes in WT or *UBAC2* KO THP-1 Mφs with TG (1 μM) or CPA (10 μM) treatment for 12 hr. (**E**) ELISA of inflammatory cytokines in WT or LIRm UBAC2 inducible THP-1 Mφs were incubated with Dox (200 ng/mL) for overnight and treated with TG (1 μM) or CPA (10 μM) for 24 hr. (**F**) ELISA of inflammatory cytokines in WT or *UBAC2* KO THP-1 Mφs with TG (1 μM) or CPA (10 μM) treatment for 24 hr. (**G**) Lysates from THP-1 Mφs overexpressing WT or LIRm UBAC2 and WT or *UBAC2* KO THP-1 Mφ were treated with TG (1 μM) or TM (5 μg/mL) for 18 hr and harvested for immunoblot analysis. (**H**) Flag-UBAC2 inducible THP-1 Mφs incubated with or without Dox (200 ng/mL) for overnight were transfected with or scramble or *MARK2*-specific siRNA. After incubation with TG (1 μM) or CPA (10 μM) for 12 hr, the expression of ER-stress inducible transcripts (up) and inflammatory genes (down) was analyzed by qPCR. Data information: For (**G**), one representative experiment out of three was shown. In (**A**–**F**, **H**), data are presented as the mean ± SEM of three independent biological experiments. The statistical significance of the difference was analyzed by unpaired two-tailed Student's *t* test, and the *P* values were shown.
