# Supplementary figures and images for: ER-phagy restrains inflammatory responses through its receptor UBAC2 (part 1 of 2)
Source: EMBO J. 2024 Sep 16;43(21):13. doi: 10.1038/s44318-024-00232-z (PMC11535055; doi:10.1038/s44318-024-00232-z)

Source data: Figure 1D.

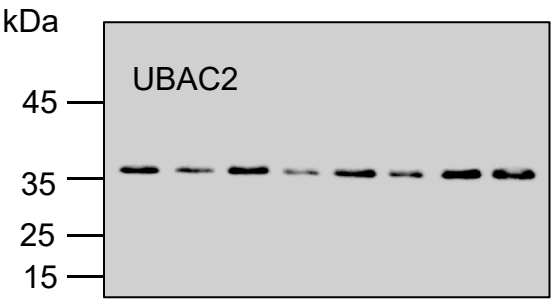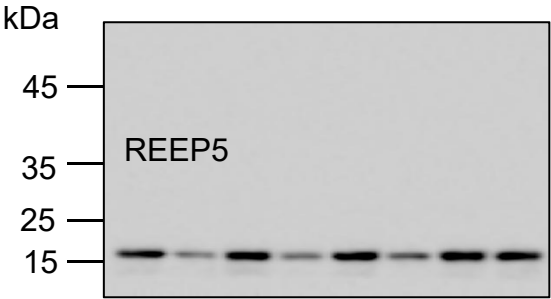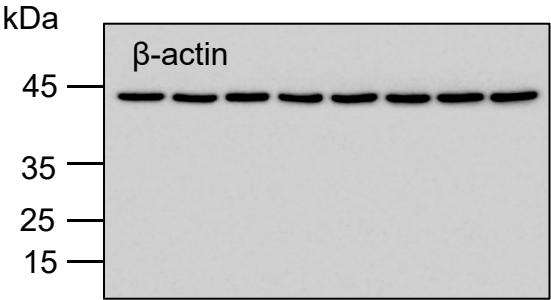

Supplement: Supplementary file 3 — Source data Fig. 1 [file 44318_2024_232_MOESM3_ESM.zip › Figure 1/Figure 1D.pdf]

Source data: Figure 1F.

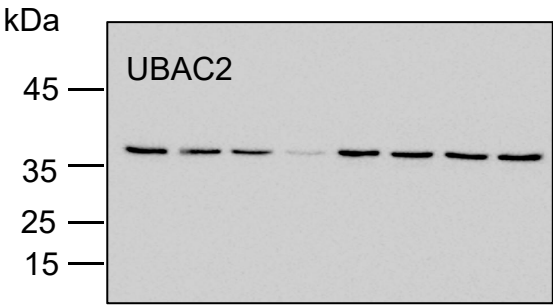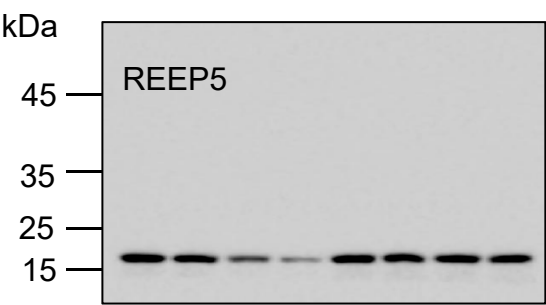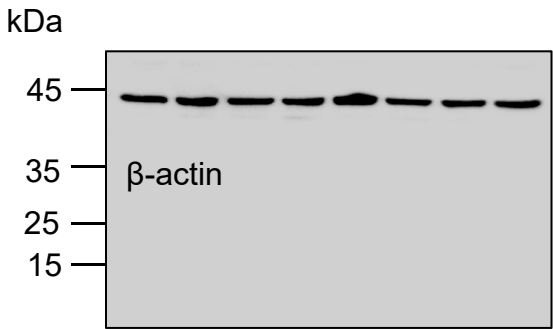

Supplement: Supplementary file 3 — Source data Fig. 1 [file 44318_2024_232_MOESM3_ESM.zip › Figure 1/Figure 1F.pdf]

Source data: Figure 1H.

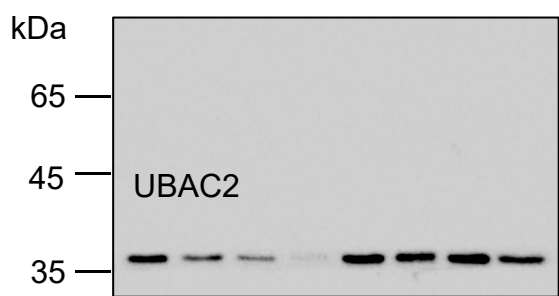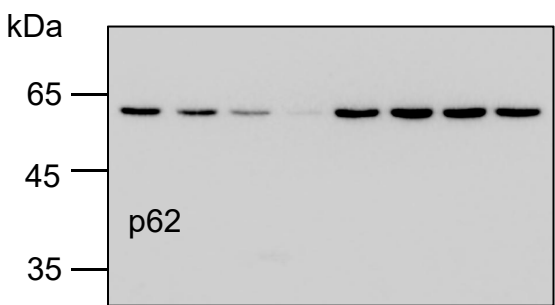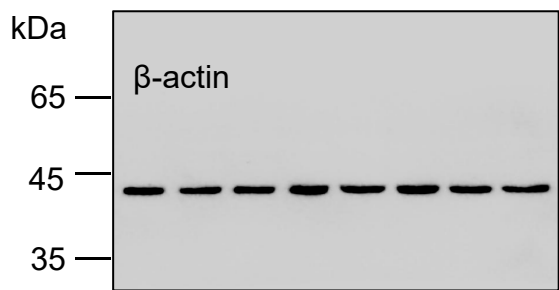

Supplement: Supplementary file 3 — Source data Fig. 1 [file 44318_2024_232_MOESM3_ESM.zip › Figure 1/Figure 1H.pdf]

Source data: Figure 1I.

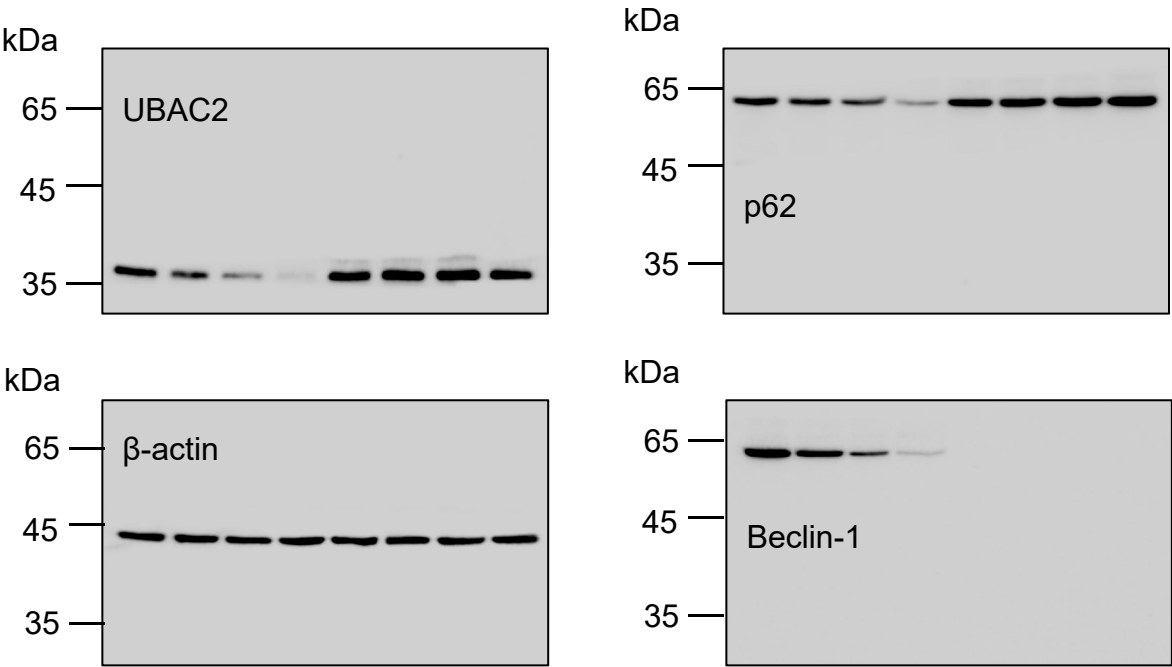

Supplement: Supplementary file 3 — Source data Fig. 1 [file 44318_2024_232_MOESM3_ESM.zip › Figure 1/Figure 1I.pdf]

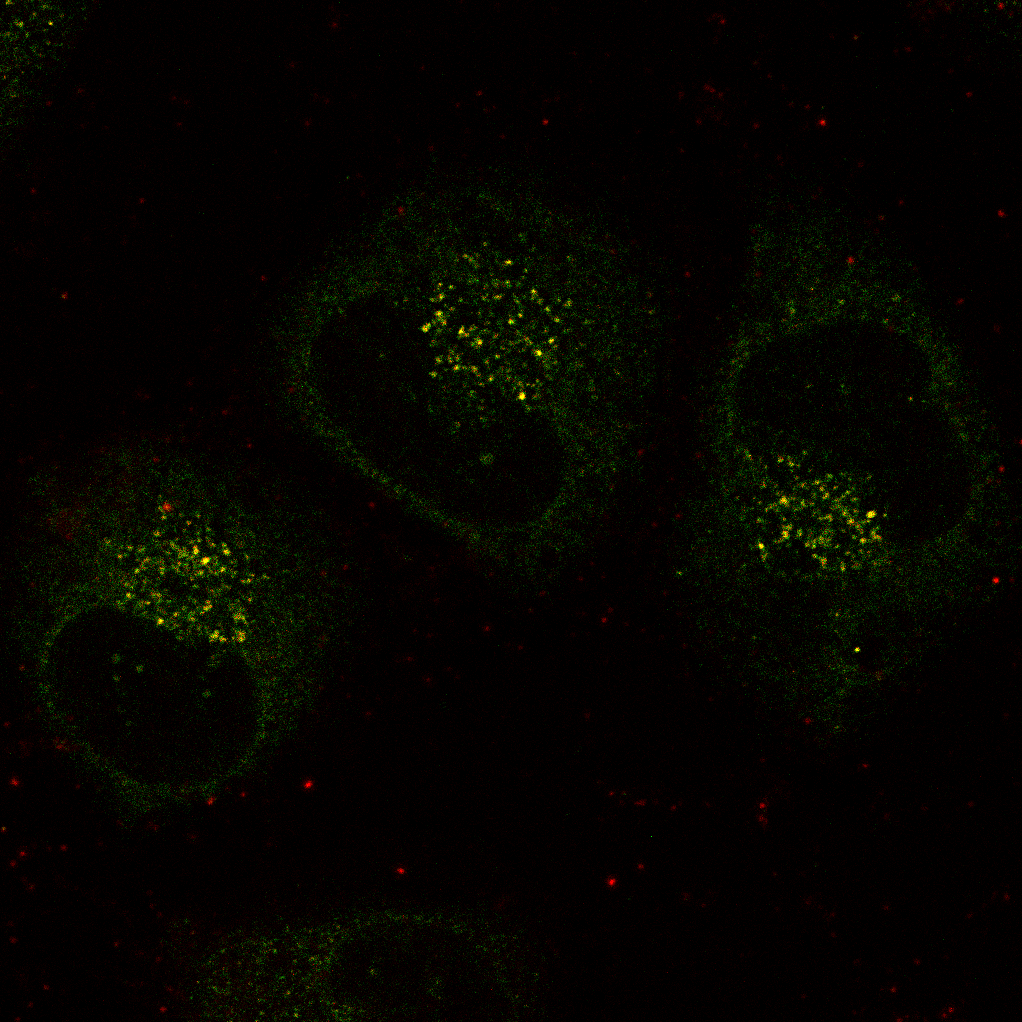

Supplement: Supplementary file 3 — Source data Fig. 1 [file 44318_2024_232_MOESM3_ESM.zip › Figure 1/Figure 1J/EBSS/Merge.tif]

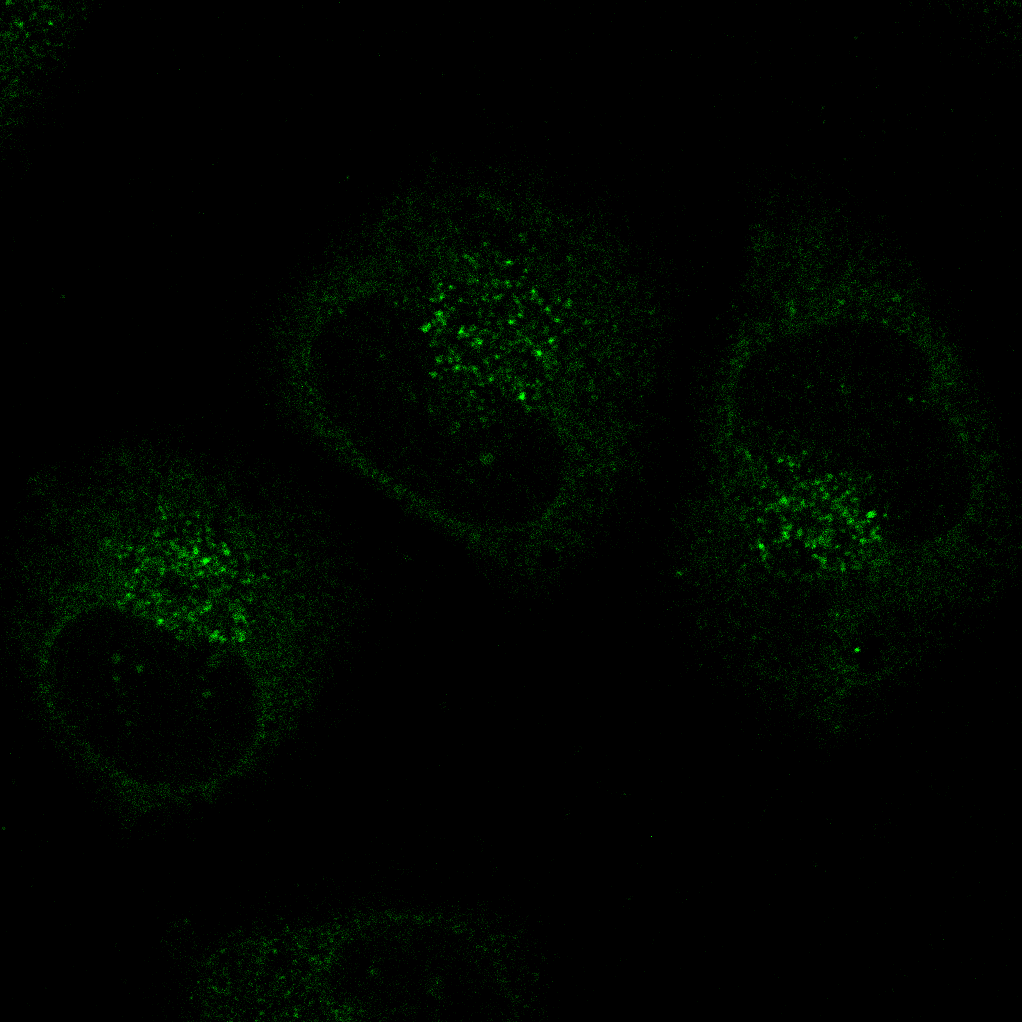

Supplement: Supplementary file 3 — Source data Fig. 1 [file 44318_2024_232_MOESM3_ESM.zip › Figure 1/Figure 1J/EBSS/UBAC2.tif]

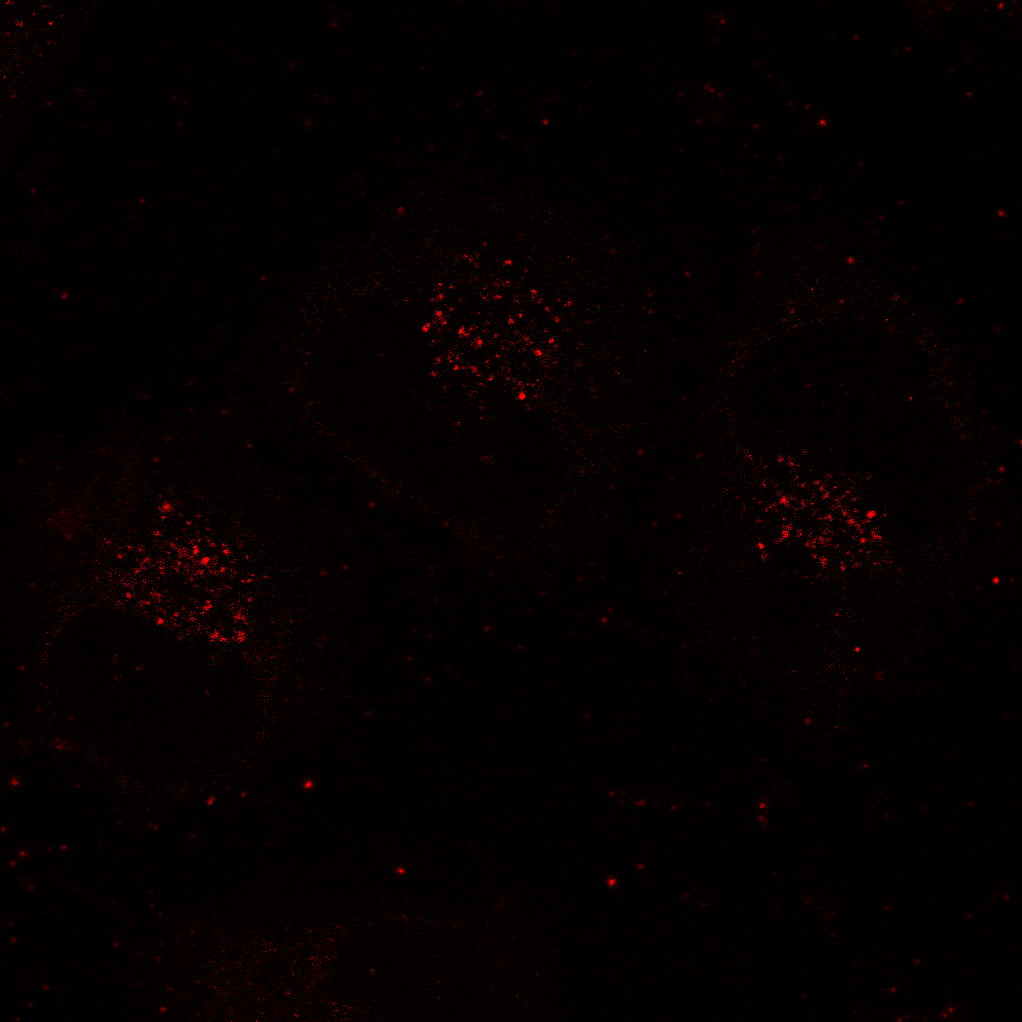

Supplement: Supplementary file 3 — Source data Fig. 1 [file 44318_2024_232_MOESM3_ESM.zip › Figure 1/Figure 1J/EBSS/WIPI2.tif]

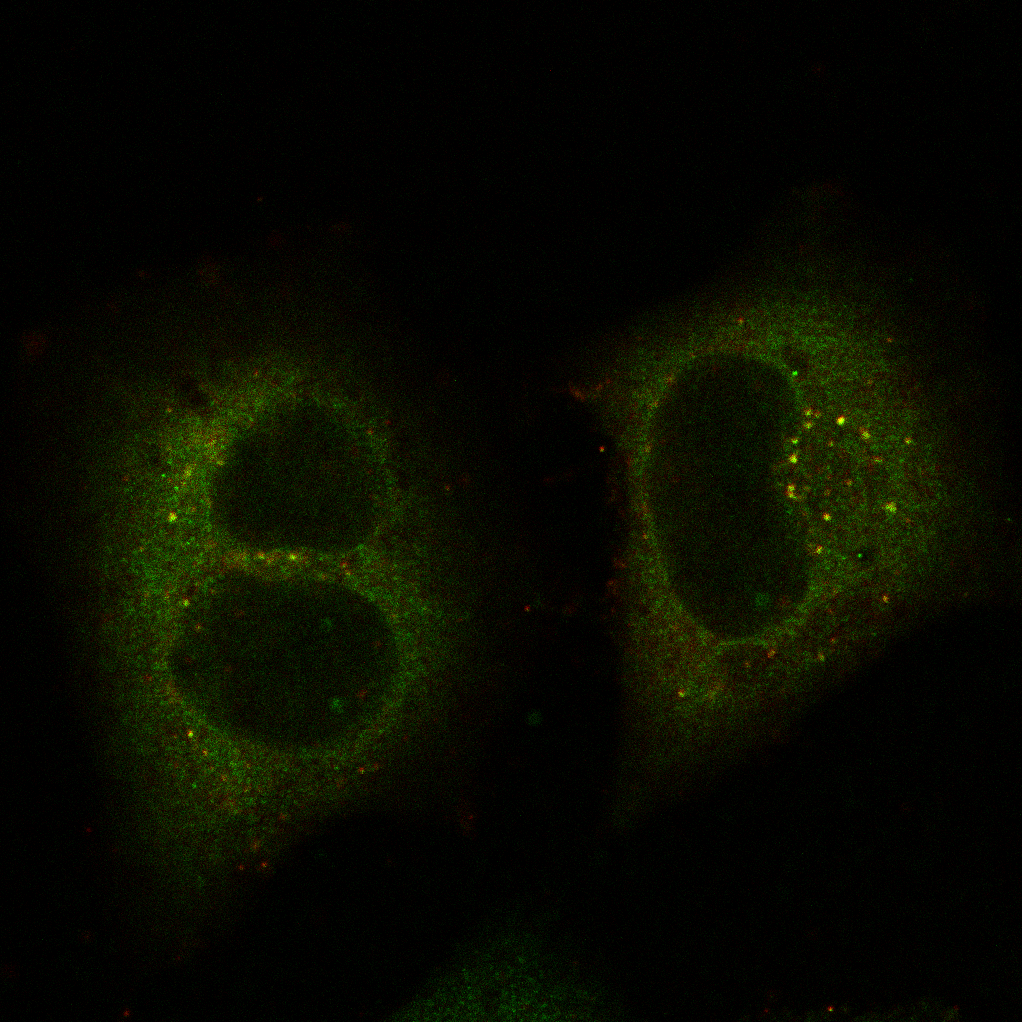

Supplement: Supplementary file 3 — Source data Fig. 1 [file 44318_2024_232_MOESM3_ESM.zip › Figure 1/Figure 1J/MOCK/Merge.tif]

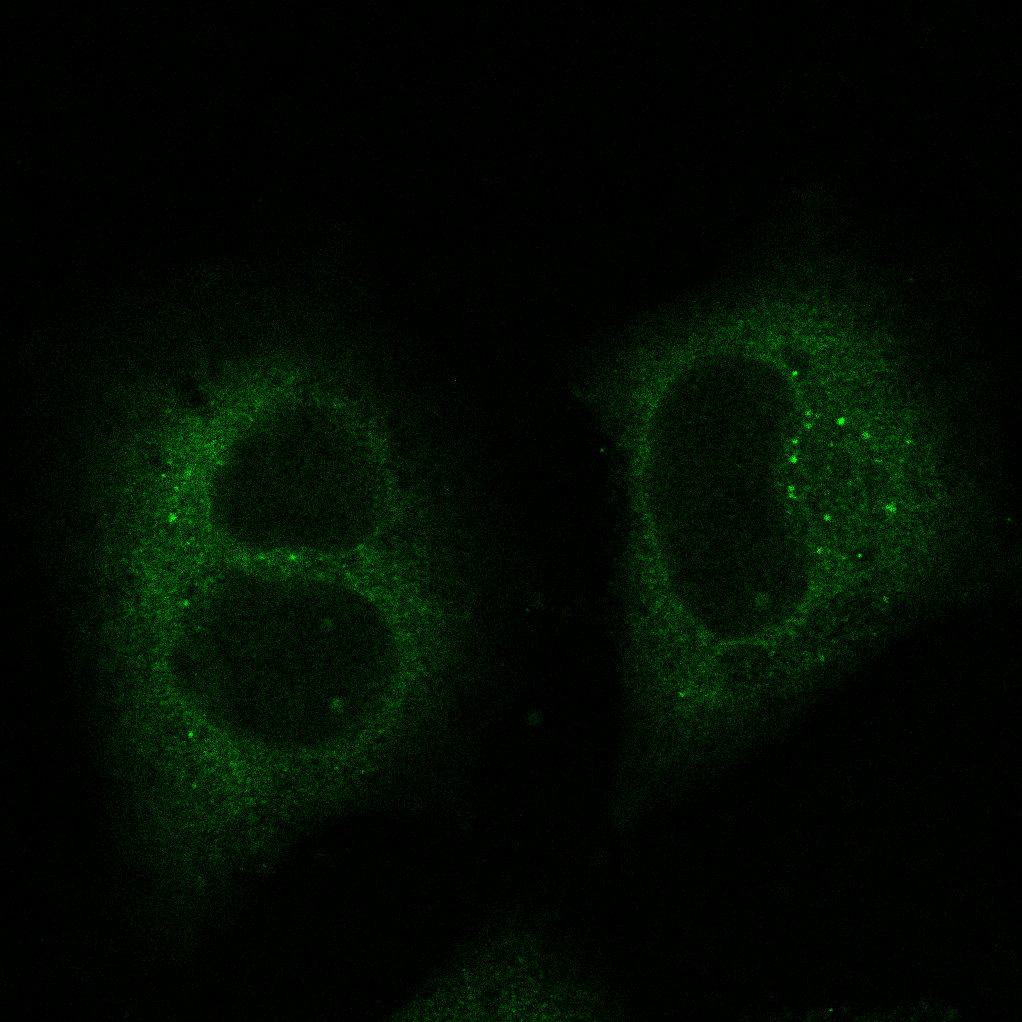

Supplement: Supplementary file 3 — Source data Fig. 1 [file 44318_2024_232_MOESM3_ESM.zip › Figure 1/Figure 1J/MOCK/UBAC2.tif]

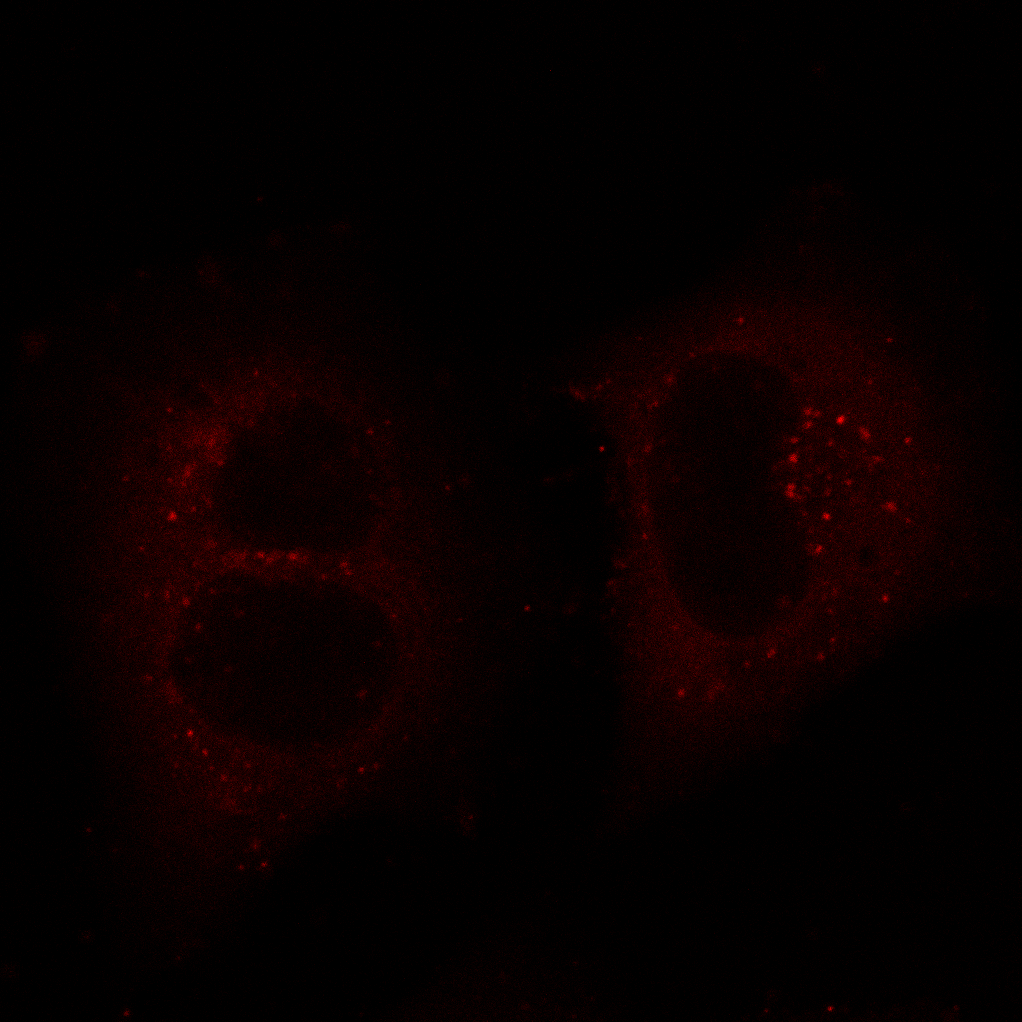

Supplement: Supplementary file 3 — Source data Fig. 1 [file 44318_2024_232_MOESM3_ESM.zip › Figure 1/Figure 1J/MOCK/WIPI2.tif]

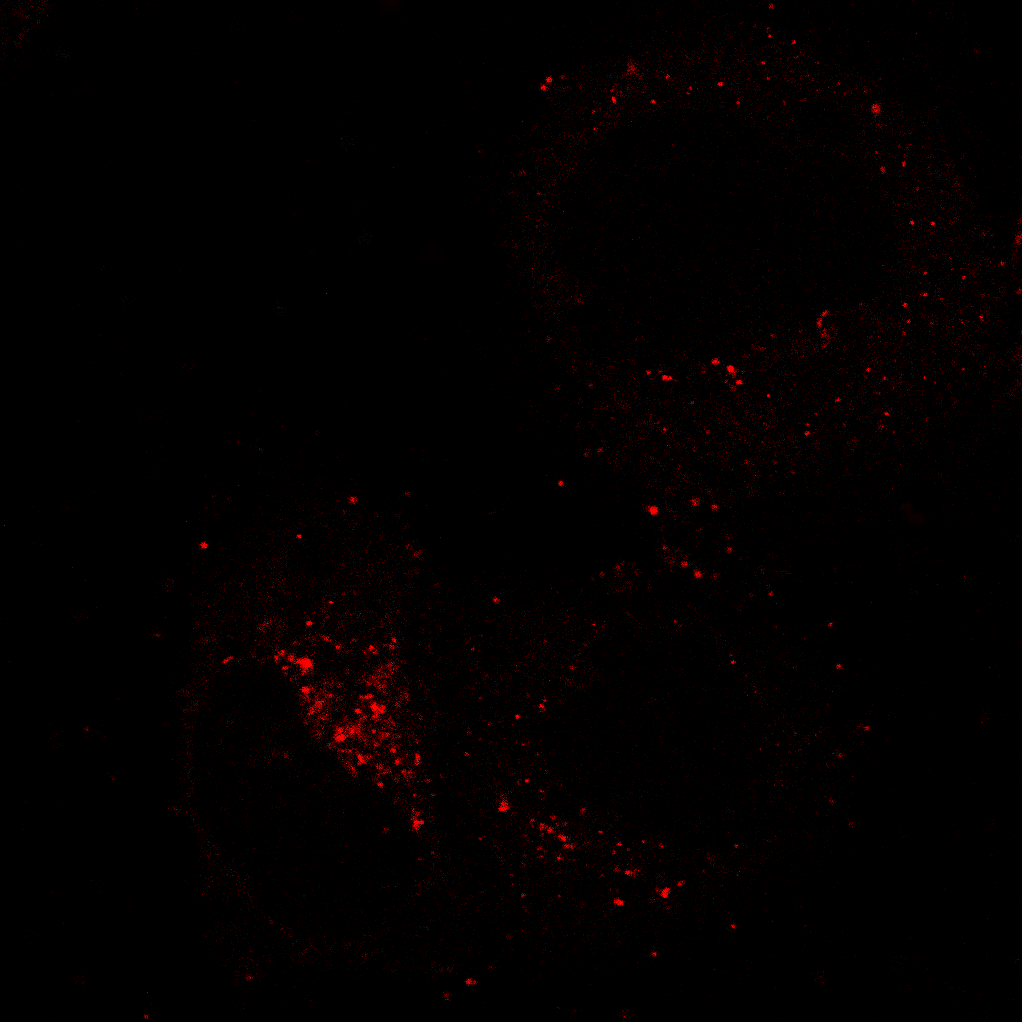

Supplement: Supplementary file 3 — Source data Fig. 1 [file 44318_2024_232_MOESM3_ESM.zip › Figure 1/Figure 1L/EBSS/ATG16L.tif]

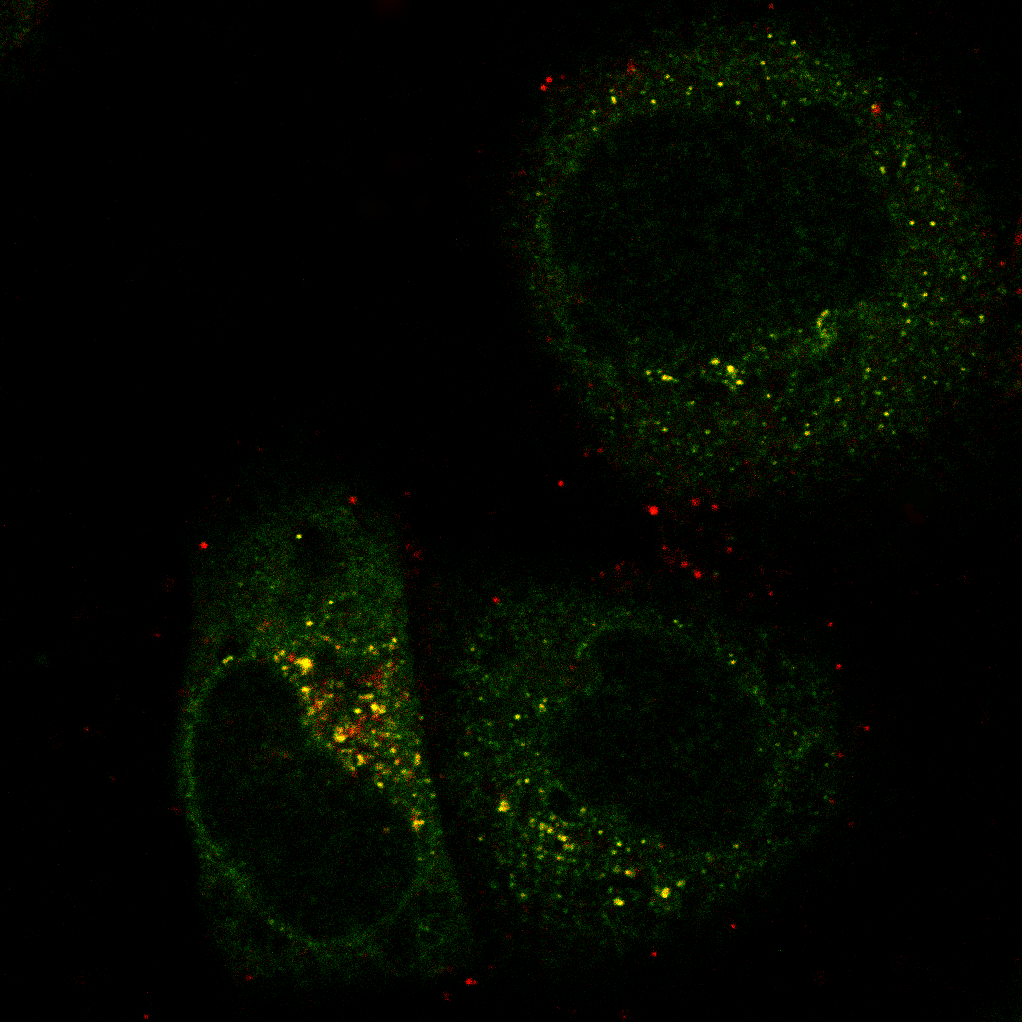

Supplement: Supplementary file 3 — Source data Fig. 1 [file 44318_2024_232_MOESM3_ESM.zip › Figure 1/Figure 1L/EBSS/Merge.tif]

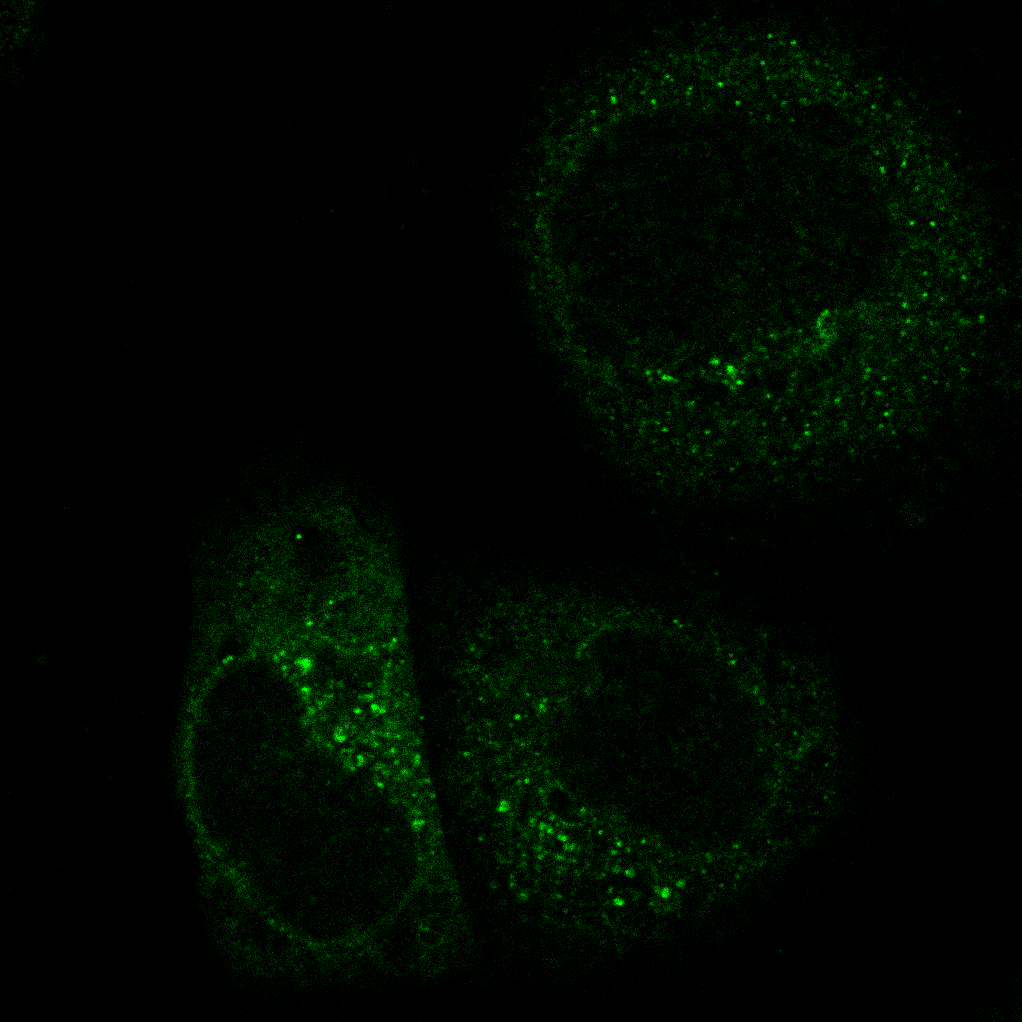

Supplement: Supplementary file 3 — Source data Fig. 1 [file 44318_2024_232_MOESM3_ESM.zip › Figure 1/Figure 1L/EBSS/UBAC2.tif]

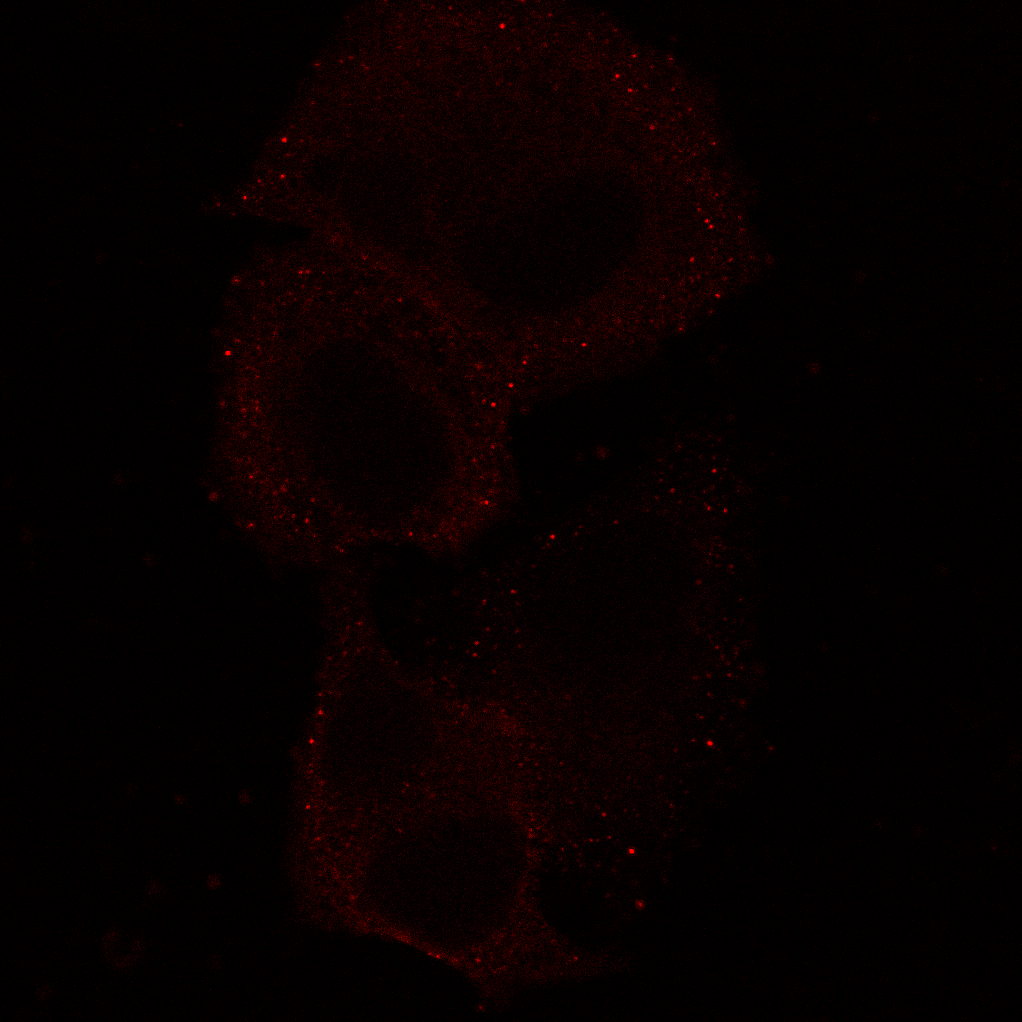

Supplement: Supplementary file 3 — Source data Fig. 1 [file 44318_2024_232_MOESM3_ESM.zip › Figure 1/Figure 1L/MOCK/ATG16L.tif]

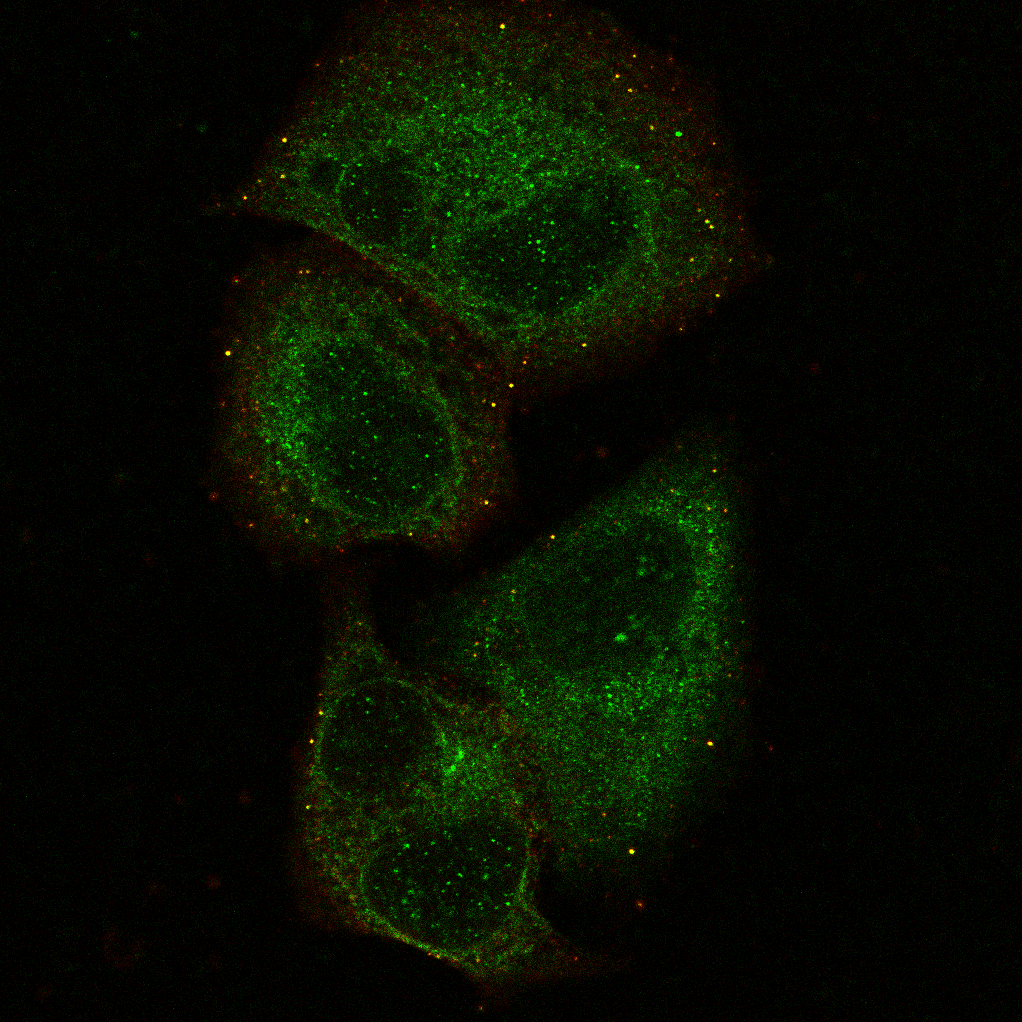

Supplement: Supplementary file 3 — Source data Fig. 1 [file 44318_2024_232_MOESM3_ESM.zip › Figure 1/Figure 1L/MOCK/Merge.tif]

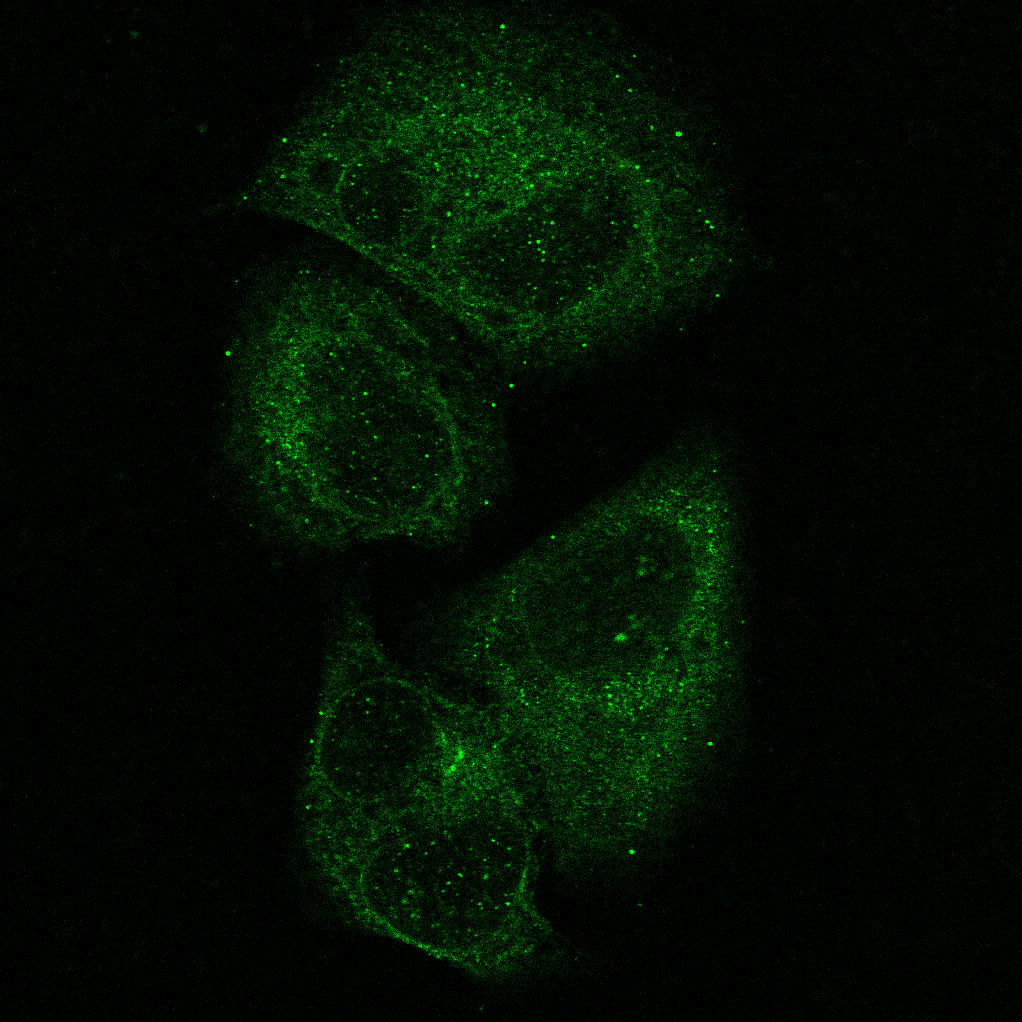

Supplement: Supplementary file 3 — Source data Fig. 1 [file 44318_2024_232_MOESM3_ESM.zip › Figure 1/Figure 1L/MOCK/UBAC2.tif]

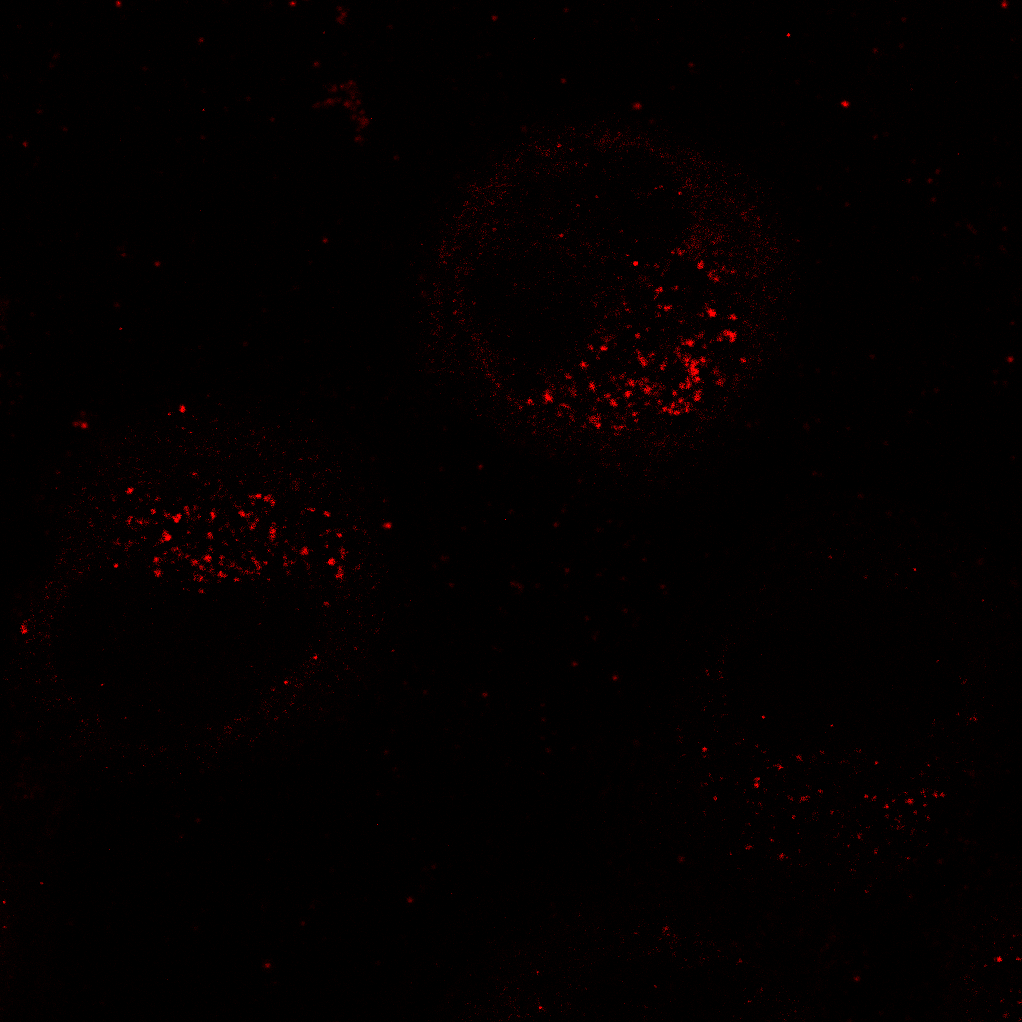

Supplement: Supplementary file 3 — Source data Fig. 1 [file 44318_2024_232_MOESM3_ESM.zip › Figure 1/Figure 1N/EBSS/LAMP1.tif]

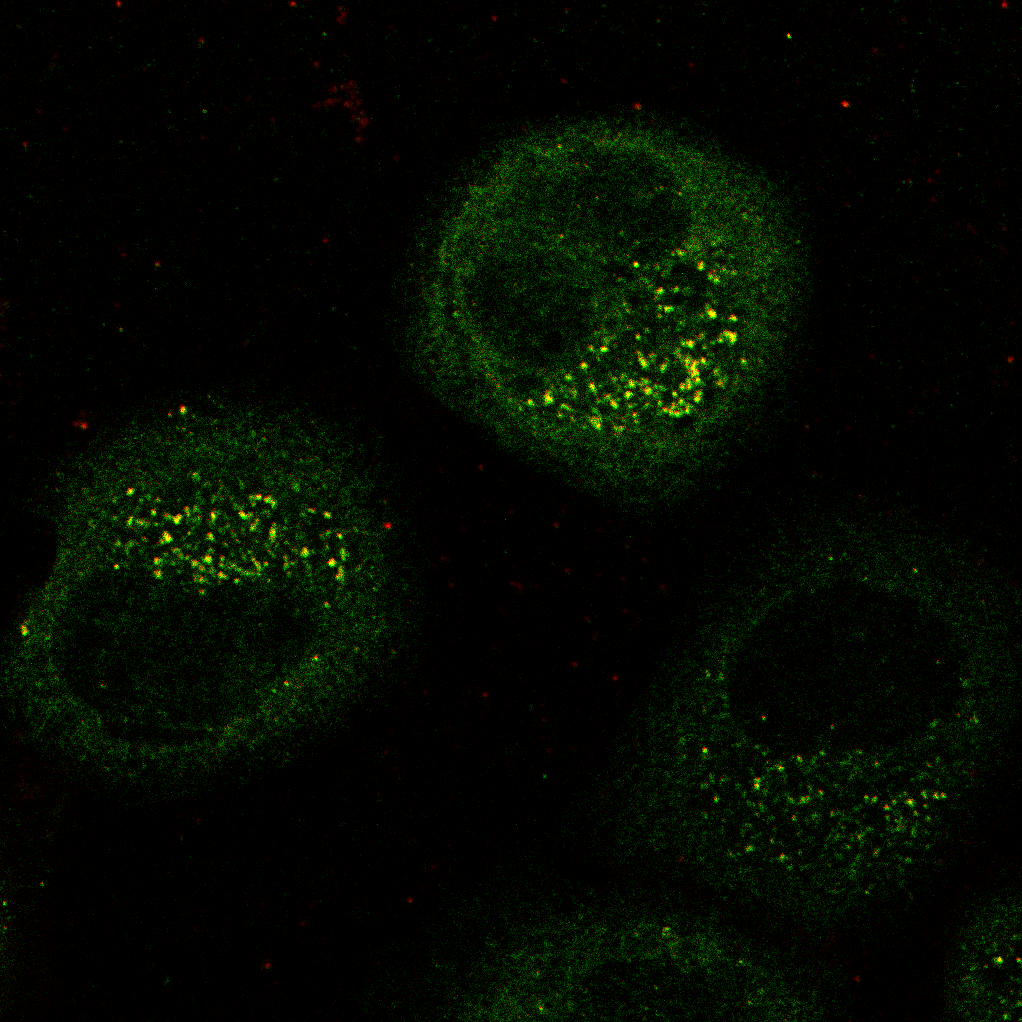

Supplement: Supplementary file 3 — Source data Fig. 1 [file 44318_2024_232_MOESM3_ESM.zip › Figure 1/Figure 1N/EBSS/Merge.tif]

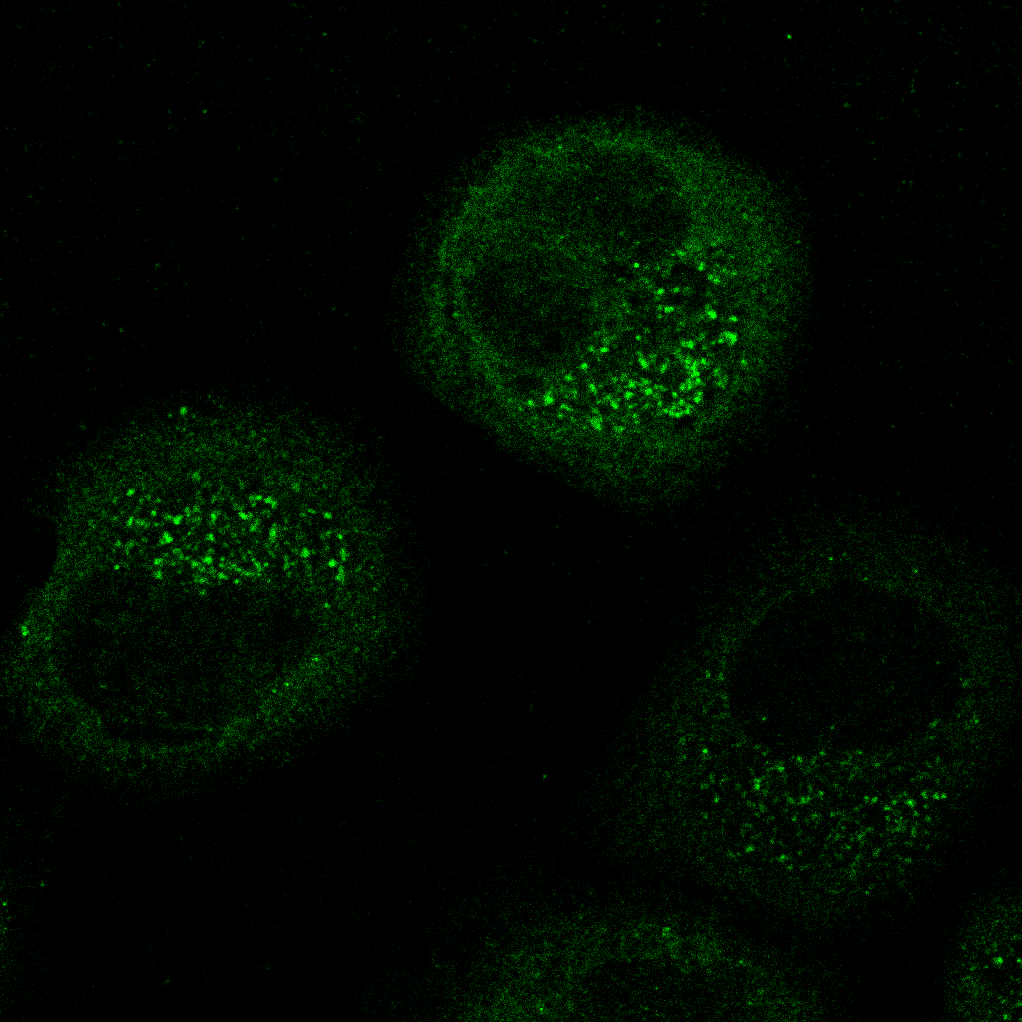

Supplement: Supplementary file 3 — Source data Fig. 1 [file 44318_2024_232_MOESM3_ESM.zip › Figure 1/Figure 1N/EBSS/UBAC2.tif]

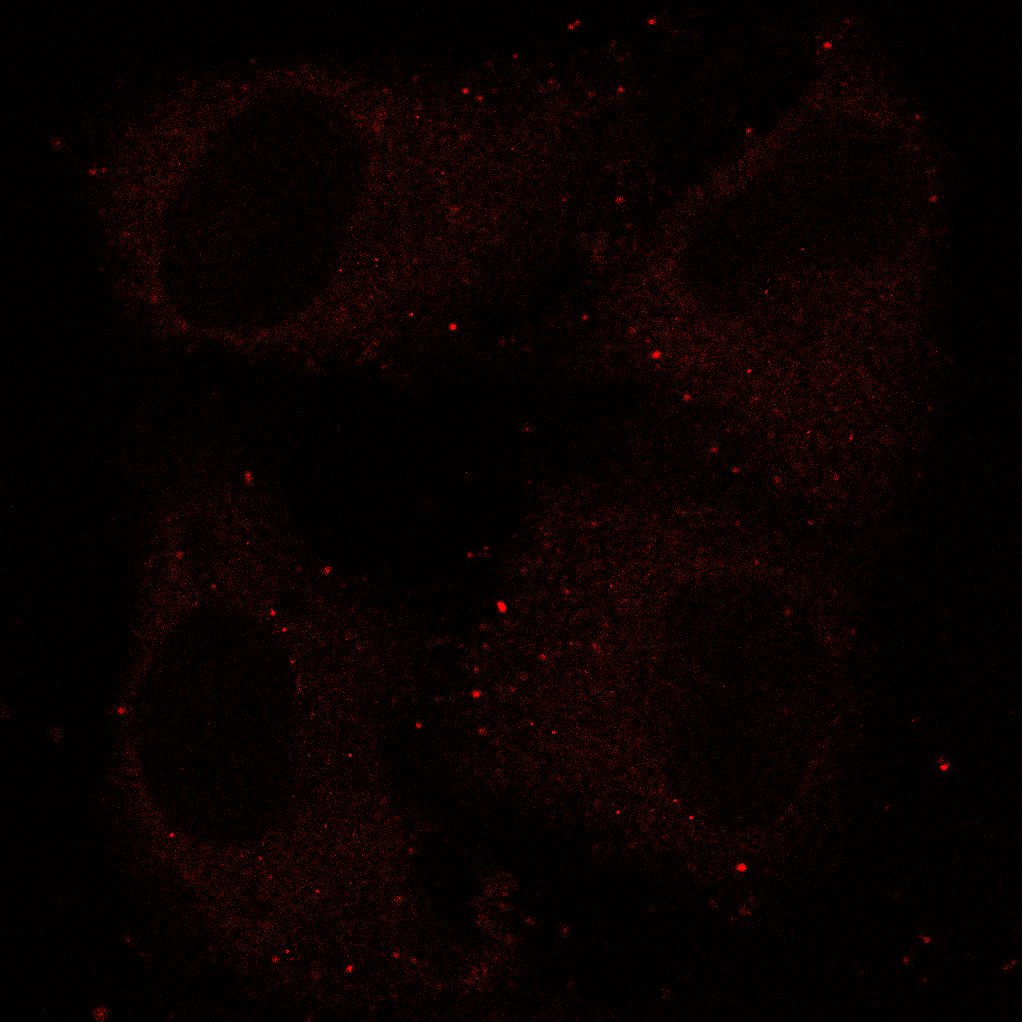

Supplement: Supplementary file 3 — Source data Fig. 1 [file 44318_2024_232_MOESM3_ESM.zip › Figure 1/Figure 1N/MOCK/LAMP1.tif]

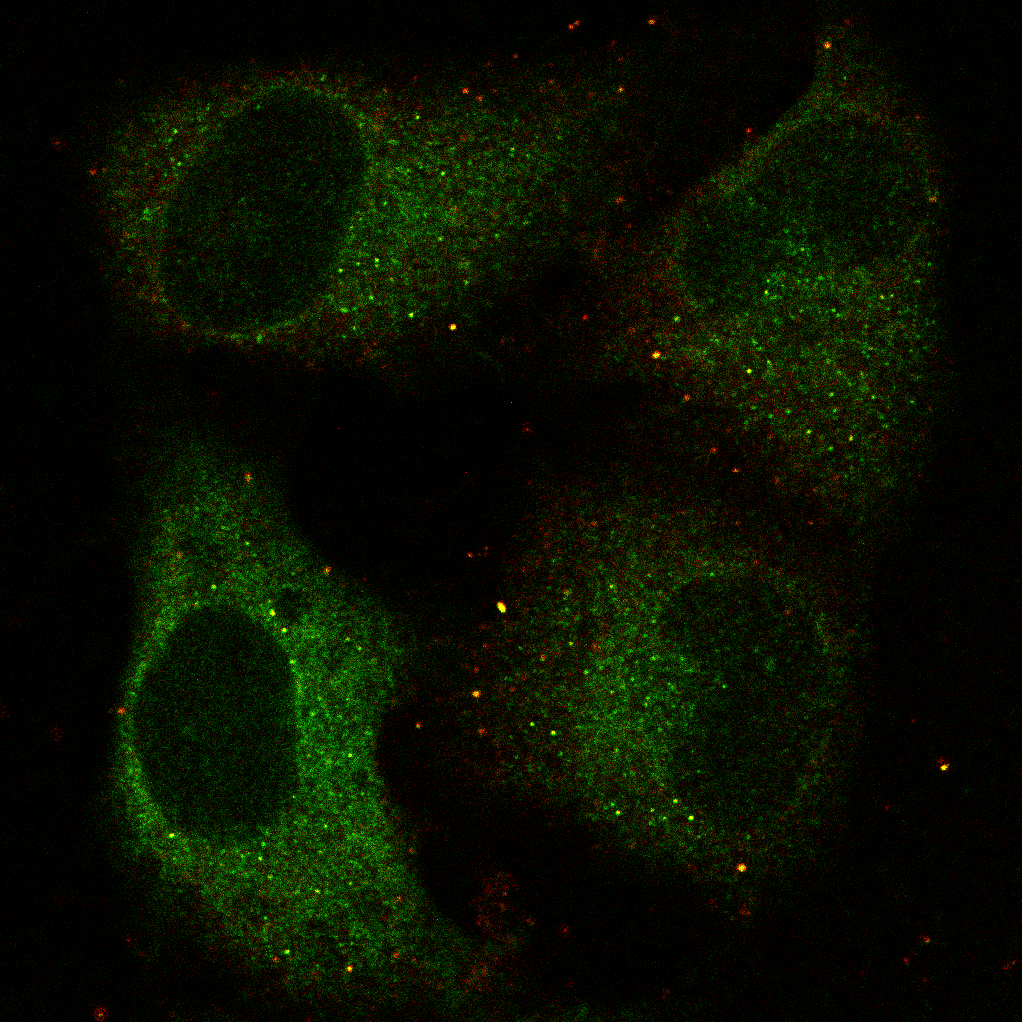

Supplement: Supplementary file 3 — Source data Fig. 1 [file 44318_2024_232_MOESM3_ESM.zip › Figure 1/Figure 1N/MOCK/Merge.tif]

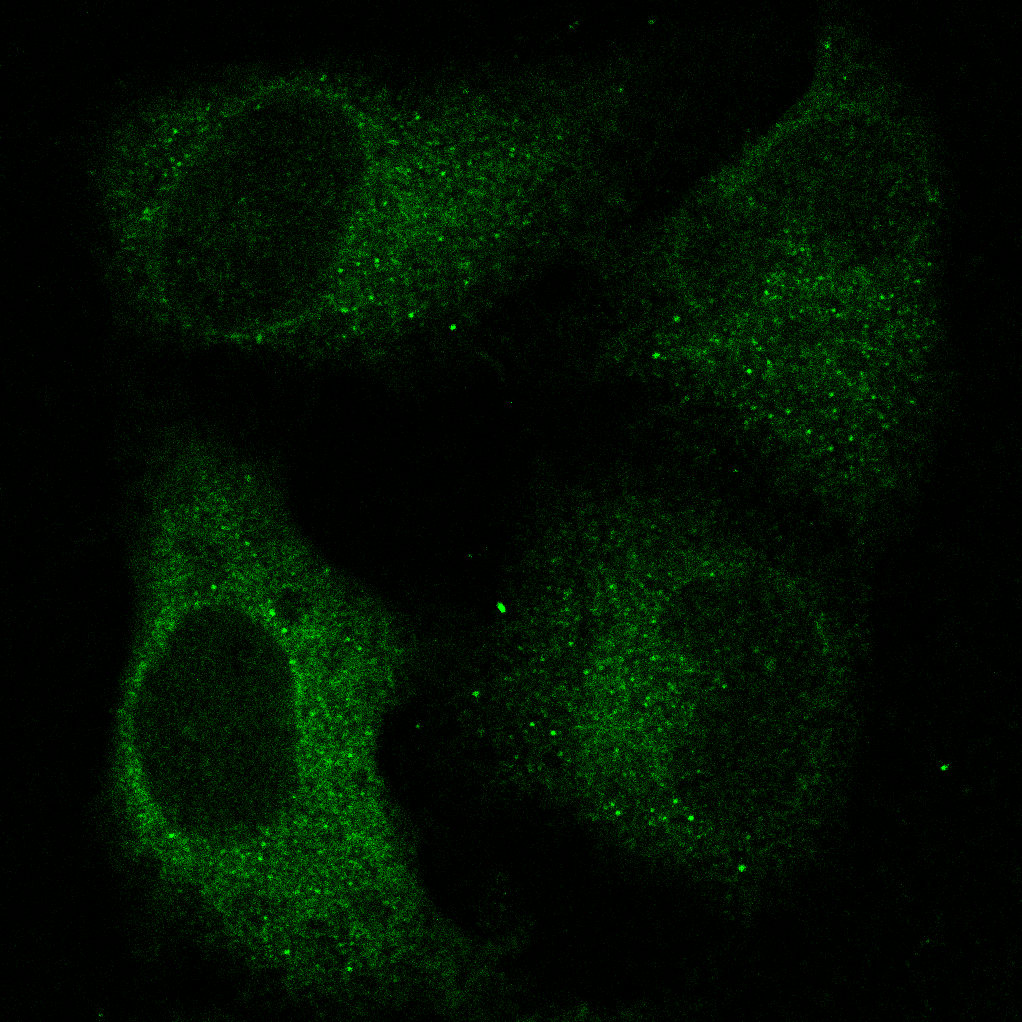

Supplement: Supplementary file 3 — Source data Fig. 1 [file 44318_2024_232_MOESM3_ESM.zip › Figure 1/Figure 1N/MOCK/UBAC2.tif]

Source data: Figure 2A.

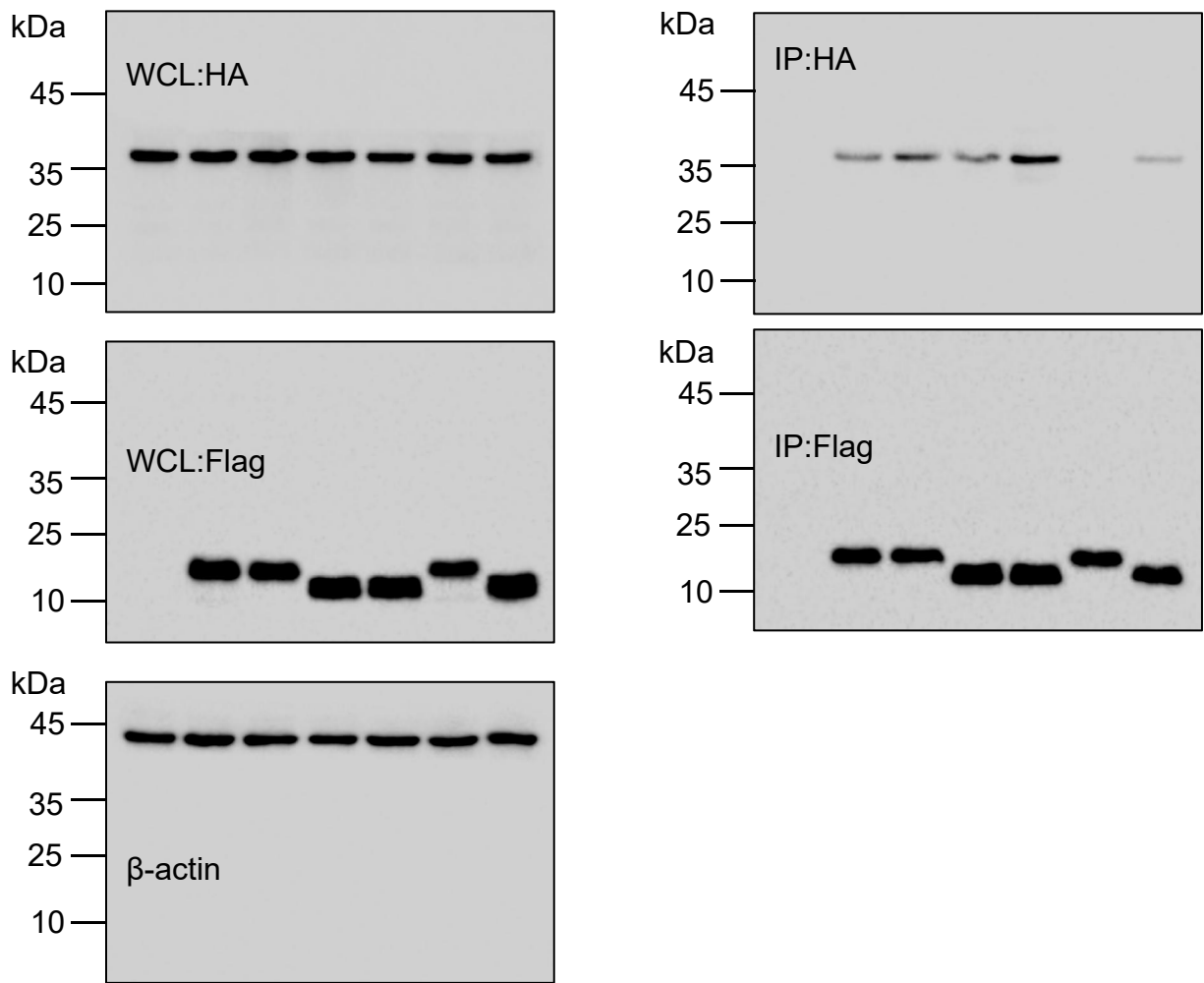

Supplement: Supplementary file 4 — Source data Fig. 2 [file 44318_2024_232_MOESM4_ESM.zip › Figure 2/Figure 2A.pdf]

Source data: Figure 2B.

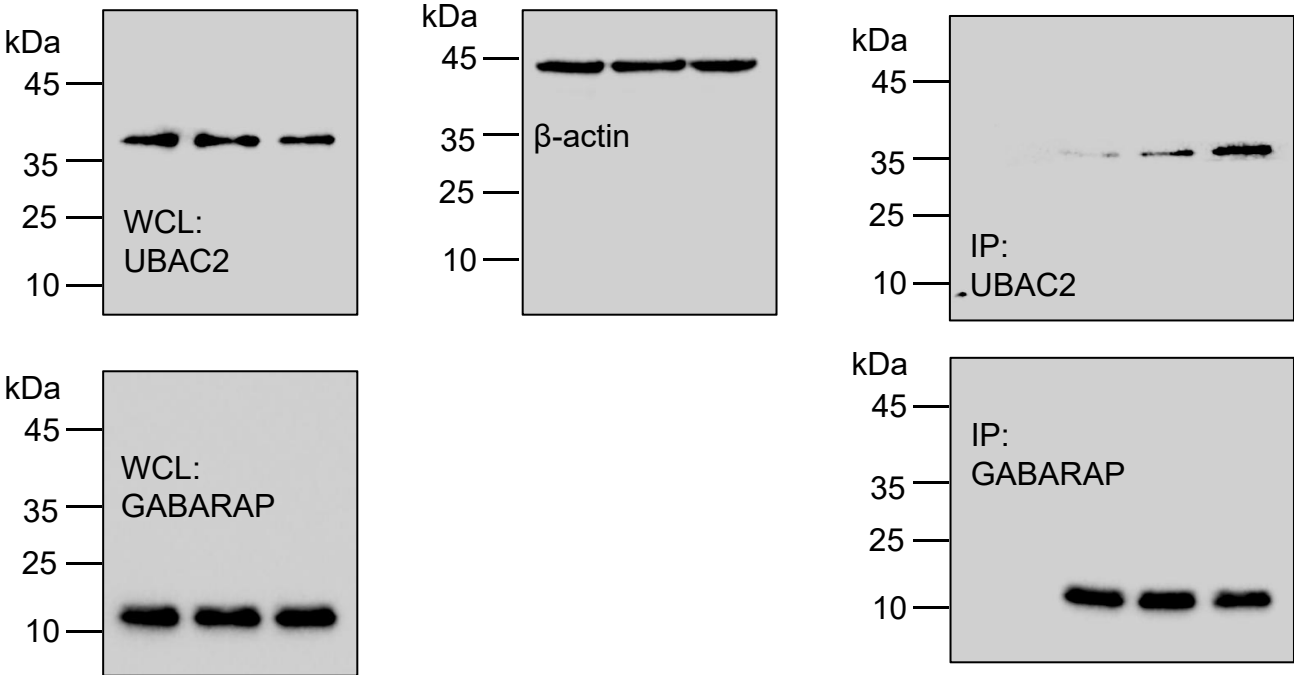

Supplement: Supplementary file 4 — Source data Fig. 2 [file 44318_2024_232_MOESM4_ESM.zip › Figure 2/Figure 2B.pdf]

Source data: Figure 2C.

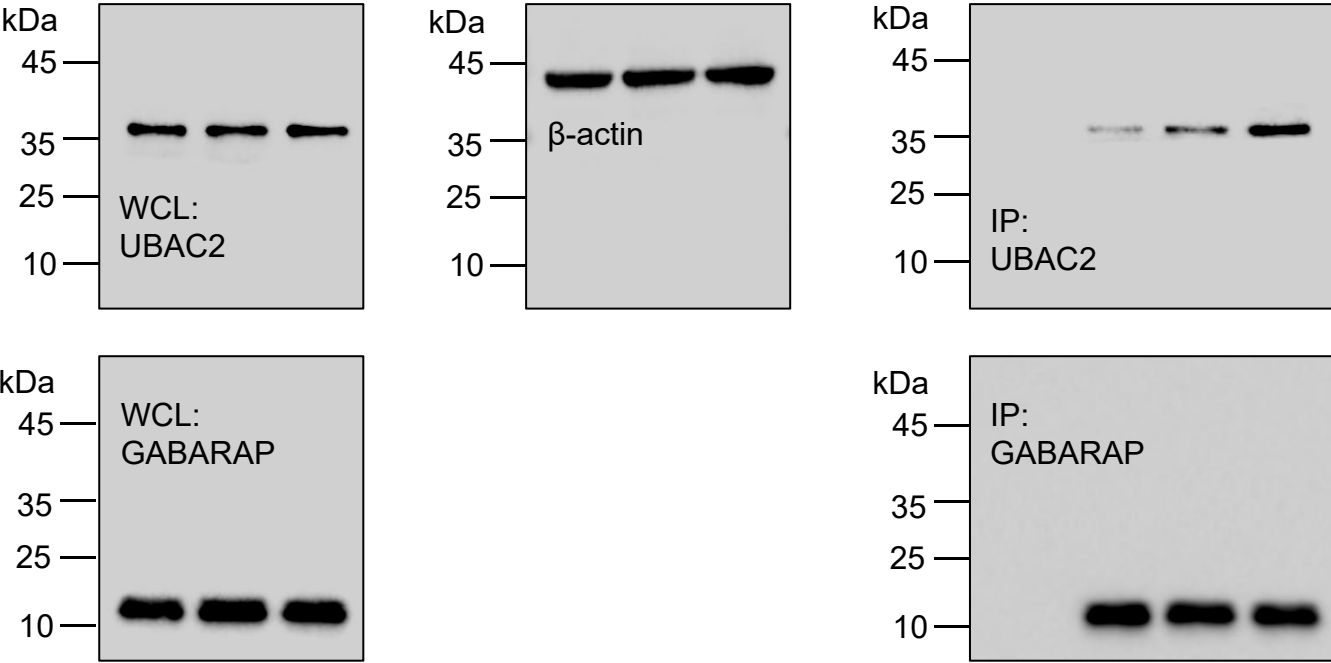

Supplement: Supplementary file 4 — Source data Fig. 2 [file 44318_2024_232_MOESM4_ESM.zip › Figure 2/Figure 2C.pdf]

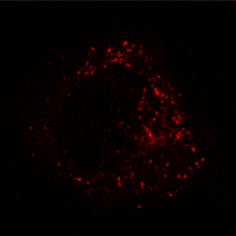

Supplement: Supplementary file 4 — Source data Fig. 2 [file 44318_2024_232_MOESM4_ESM.zip › Figure 2/Figure 2D/EBSS/GABARAP.tif]

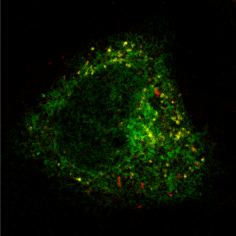

Supplement: Supplementary file 4 — Source data Fig. 2 [file 44318_2024_232_MOESM4_ESM.zip › Figure 2/Figure 2D/EBSS/Merge.tif]

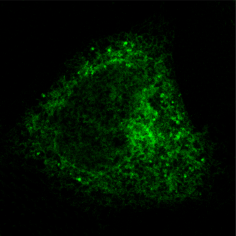

Supplement: Supplementary file 4 — Source data Fig. 2 [file 44318_2024_232_MOESM4_ESM.zip › Figure 2/Figure 2D/EBSS/UBAC2.tif]

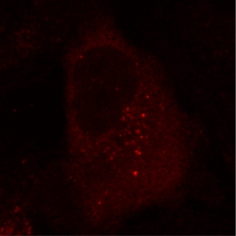

Supplement: Supplementary file 4 — Source data Fig. 2 [file 44318_2024_232_MOESM4_ESM.zip › Figure 2/Figure 2D/MOCK/GABARAP.tif]

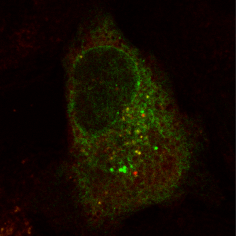

Supplement: Supplementary file 4 — Source data Fig. 2 [file 44318_2024_232_MOESM4_ESM.zip › Figure 2/Figure 2D/MOCK/Merge.tif]

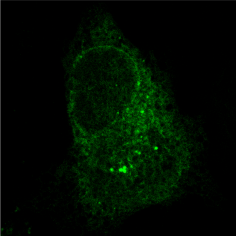

Supplement: Supplementary file 4 — Source data Fig. 2 [file 44318_2024_232_MOESM4_ESM.zip › Figure 2/Figure 2D/MOCK/UBAC2.tif]

Source data: Figure 2G.

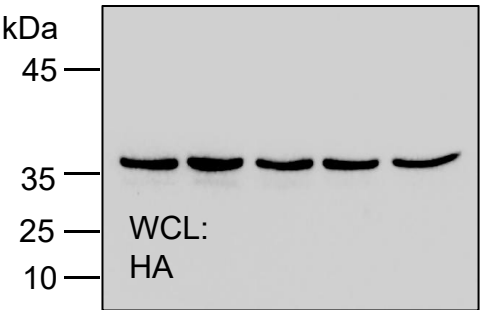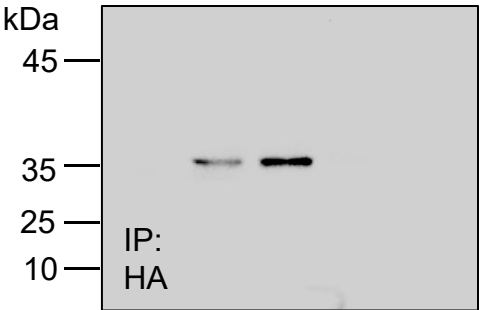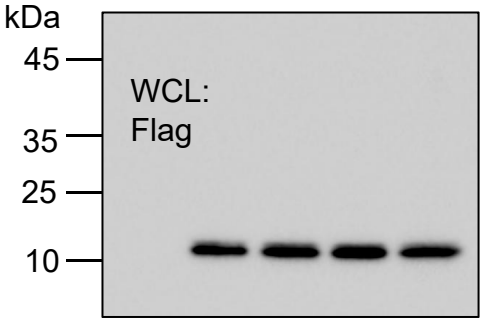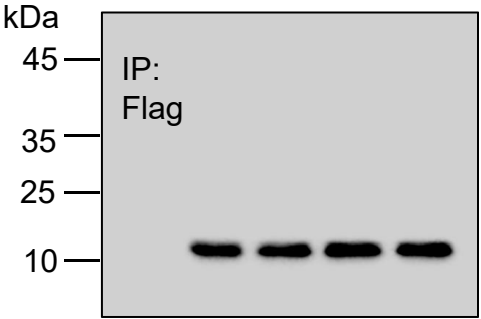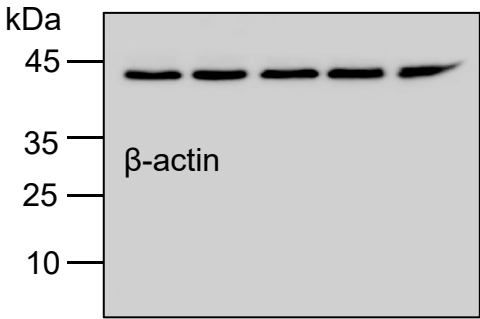

Supplement: Supplementary file 4 — Source data Fig. 2 [file 44318_2024_232_MOESM4_ESM.zip › Figure 2/Figure 2G.pdf]

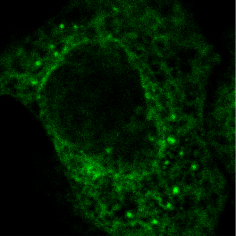

Supplement: Supplementary file 4 — Source data Fig. 2 [file 44318_2024_232_MOESM4_ESM.zip › Figure 2/Figure 2H/LIRM/Flag.tif]

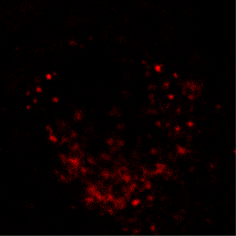

Supplement: Supplementary file 4 — Source data Fig. 2 [file 44318_2024_232_MOESM4_ESM.zip › Figure 2/Figure 2H/LIRM/GABARAP.tif]

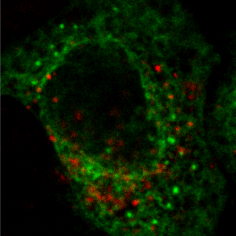

Supplement: Supplementary file 4 — Source data Fig. 2 [file 44318_2024_232_MOESM4_ESM.zip › Figure 2/Figure 2H/LIRM/Merge.tif]

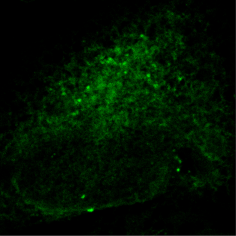

Supplement: Supplementary file 4 — Source data Fig. 2 [file 44318_2024_232_MOESM4_ESM.zip › Figure 2/Figure 2H/WT/Flag.tif]

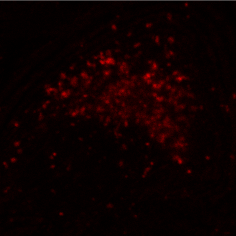

Supplement: Supplementary file 4 — Source data Fig. 2 [file 44318_2024_232_MOESM4_ESM.zip › Figure 2/Figure 2H/WT/GABARAP.tif]

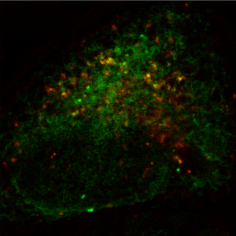

Supplement: Supplementary file 4 — Source data Fig. 2 [file 44318_2024_232_MOESM4_ESM.zip › Figure 2/Figure 2H/WT/Merge.tif]

Source data: Figure 2J.

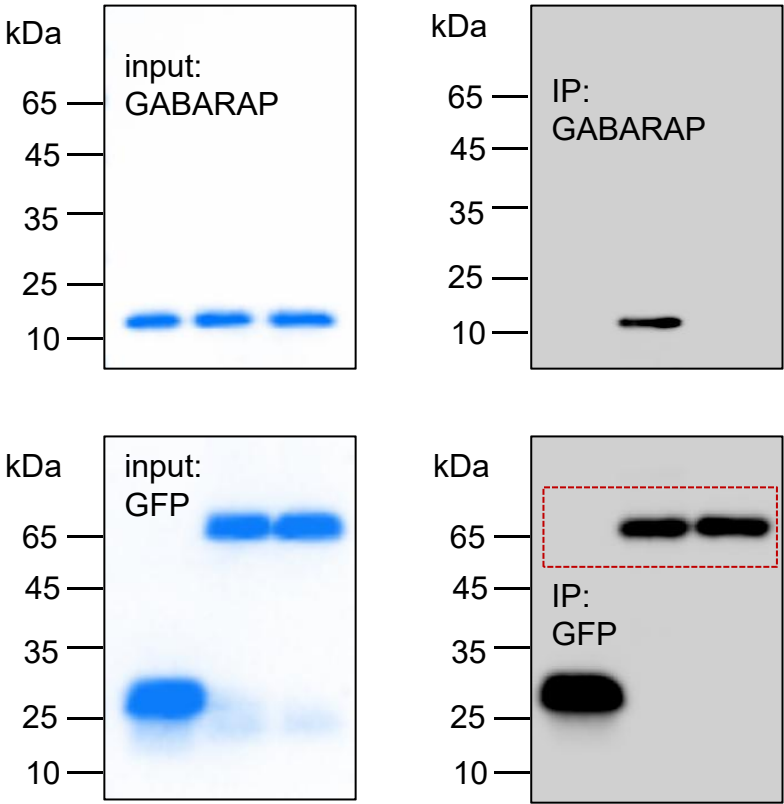

Supplement: Supplementary file 4 — Source data Fig. 2 [file 44318_2024_232_MOESM4_ESM.zip › Figure 2/Figure 2J.pdf]

Source data: Figure 2K.

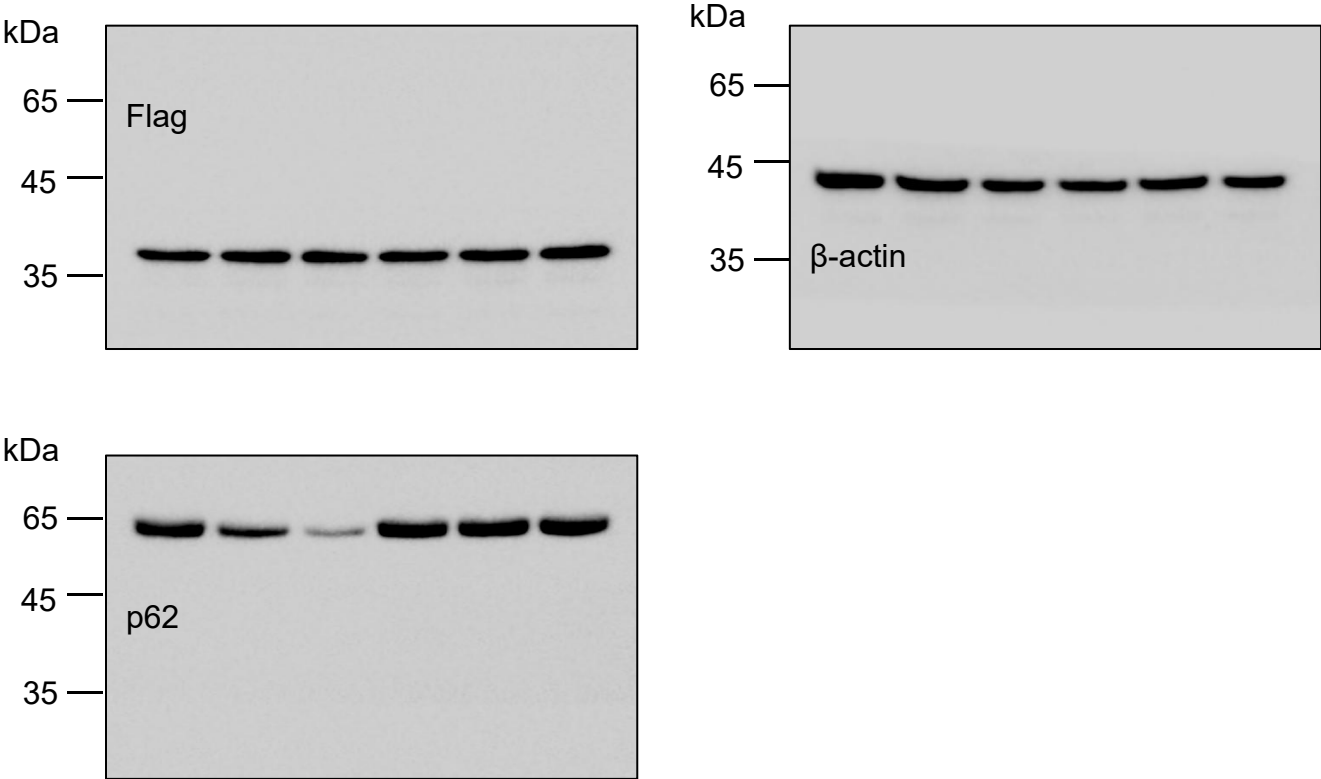

Supplement: Supplementary file 4 — Source data Fig. 2 [file 44318_2024_232_MOESM4_ESM.zip › Figure 2/Figure 2K.pdf]

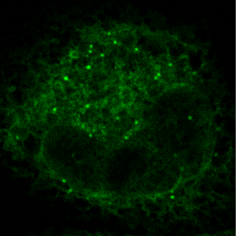

Supplement: Supplementary file 4 — Source data Fig. 2 [file 44318_2024_232_MOESM4_ESM.zip › Figure 2/Figure 2L/LIRM/GFP.tif]

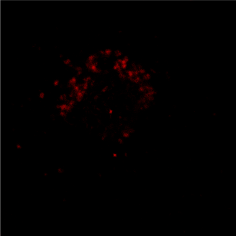

Supplement: Supplementary file 4 — Source data Fig. 2 [file 44318_2024_232_MOESM4_ESM.zip › Figure 2/Figure 2L/LIRM/LAMP1.tif]

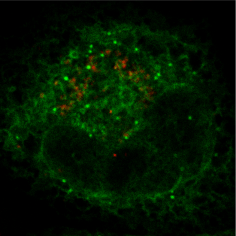

Supplement: Supplementary file 4 — Source data Fig. 2 [file 44318_2024_232_MOESM4_ESM.zip › Figure 2/Figure 2L/LIRM/Merge.tif]

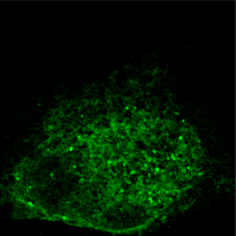

Supplement: Supplementary file 4 — Source data Fig. 2 [file 44318_2024_232_MOESM4_ESM.zip › Figure 2/Figure 2L/WT/GFP.tif]

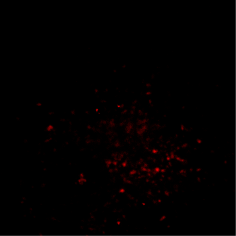

Supplement: Supplementary file 4 — Source data Fig. 2 [file 44318_2024_232_MOESM4_ESM.zip › Figure 2/Figure 2L/WT/LAMP1.tif]

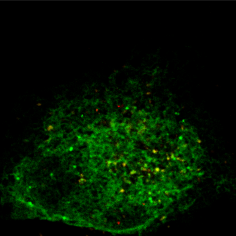

Supplement: Supplementary file 4 — Source data Fig. 2 [file 44318_2024_232_MOESM4_ESM.zip › Figure 2/Figure 2L/WT/marge.tif]

Source data: Figure 2N.

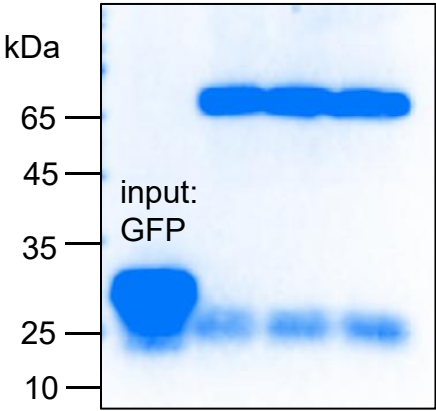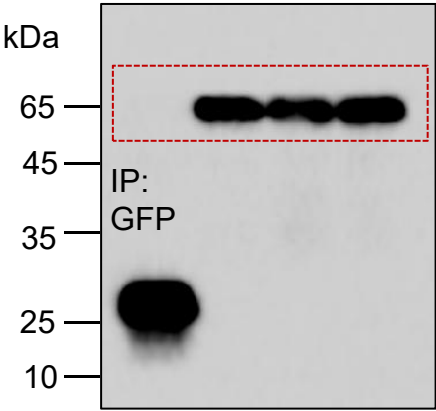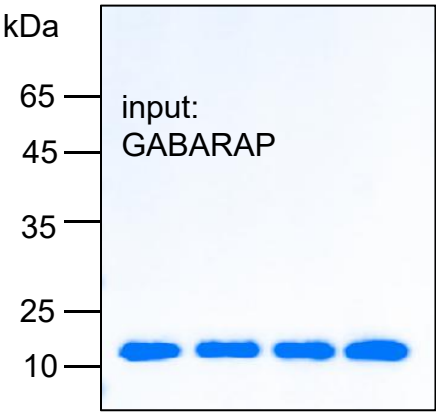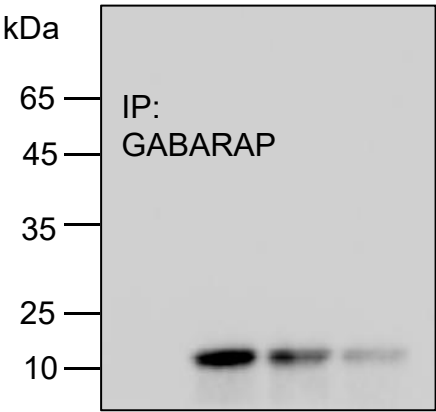

Supplement: Supplementary file 4 — Source data Fig. 2 [file 44318_2024_232_MOESM4_ESM.zip › Figure 2/Figure 2N.pdf]

Source data: Figure 3B.

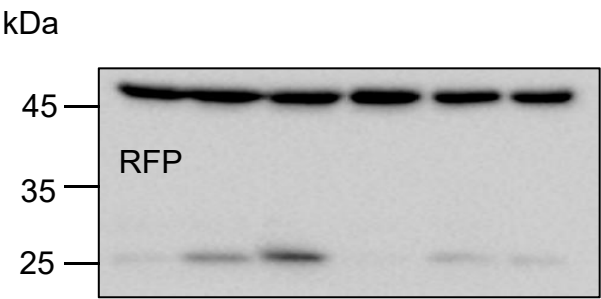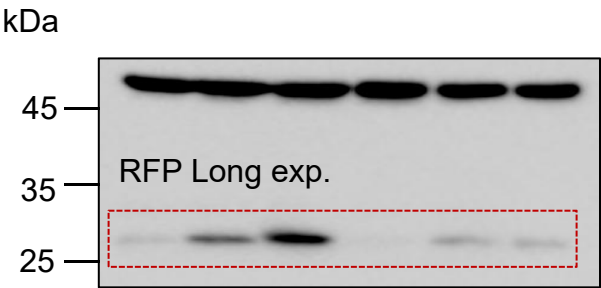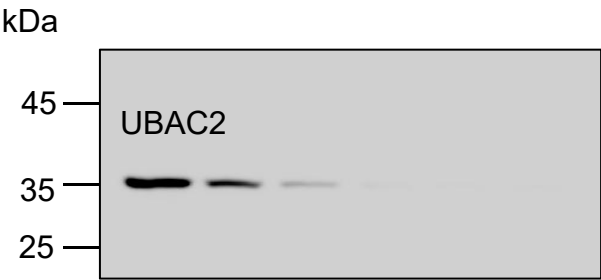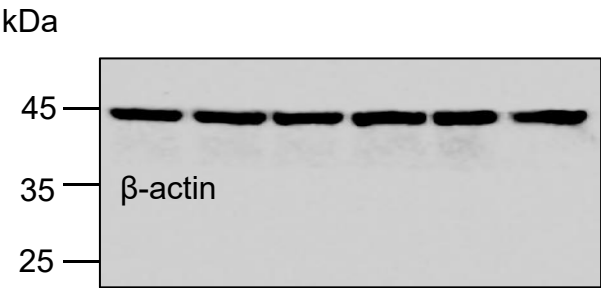

Supplement: Supplementary file 5 — Source data Fig. 3 [file 44318_2024_232_MOESM5_ESM.zip › Figure 3/Figure 3B.pdf]

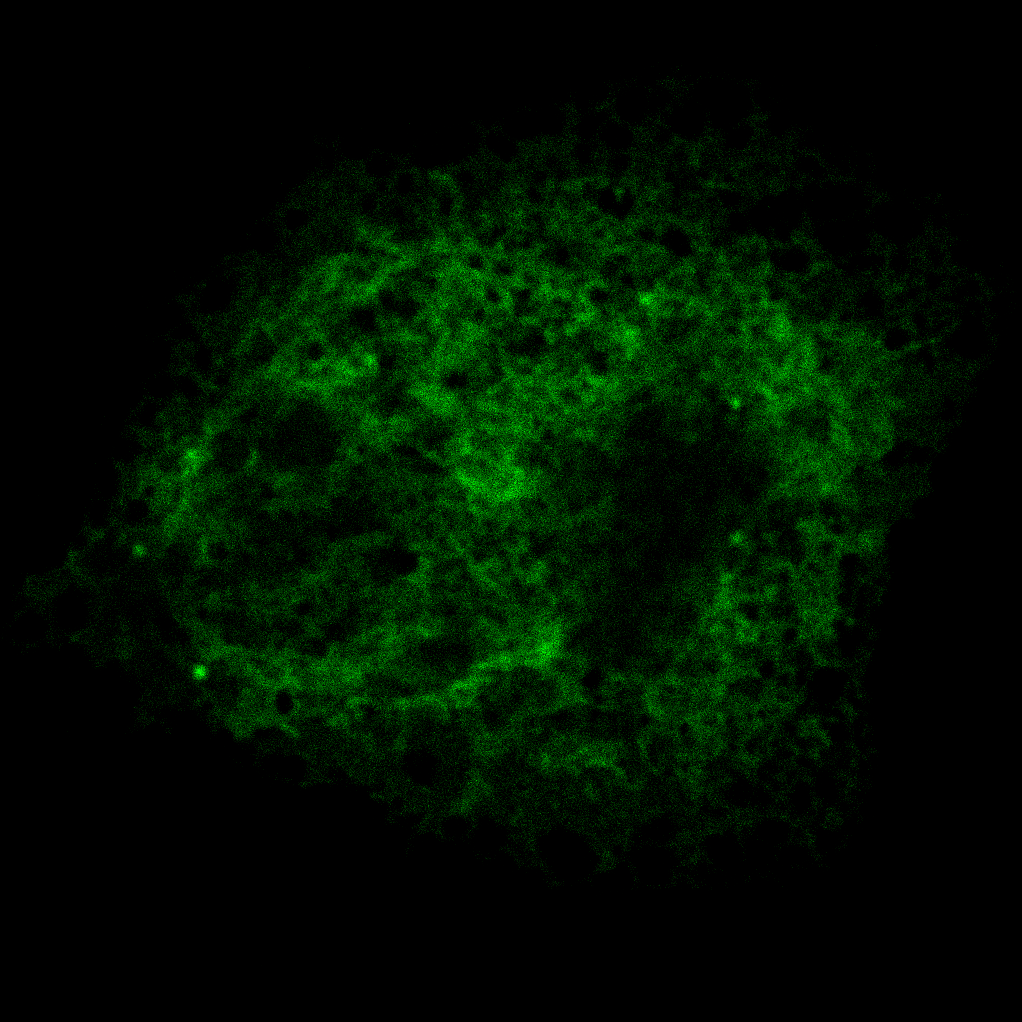

Supplement: Supplementary file 5 — Source data Fig. 3 [file 44318_2024_232_MOESM5_ESM.zip › Figure 3/Figure 3D/UBAC2 KO/GFP.tif]

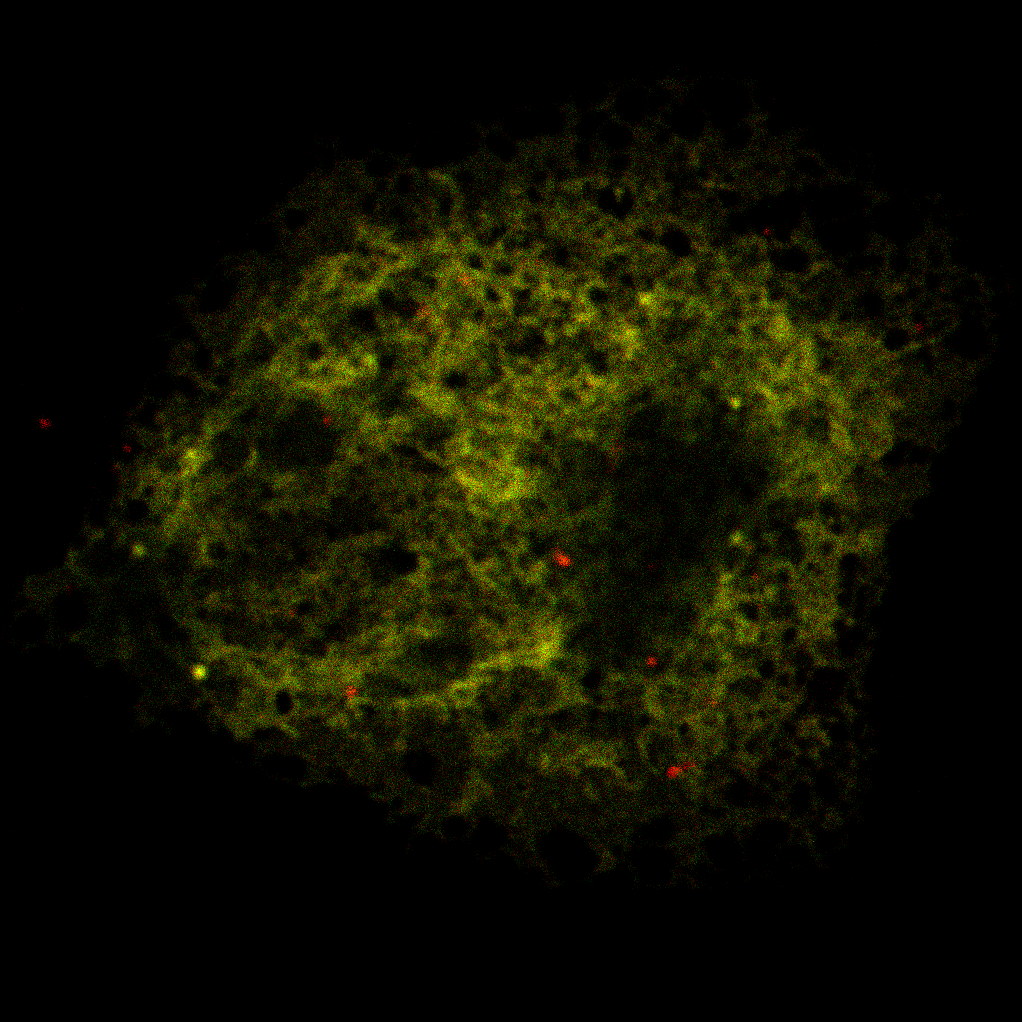

Supplement: Supplementary file 5 — Source data Fig. 3 [file 44318_2024_232_MOESM5_ESM.zip › Figure 3/Figure 3D/UBAC2 KO/Merge.tif]

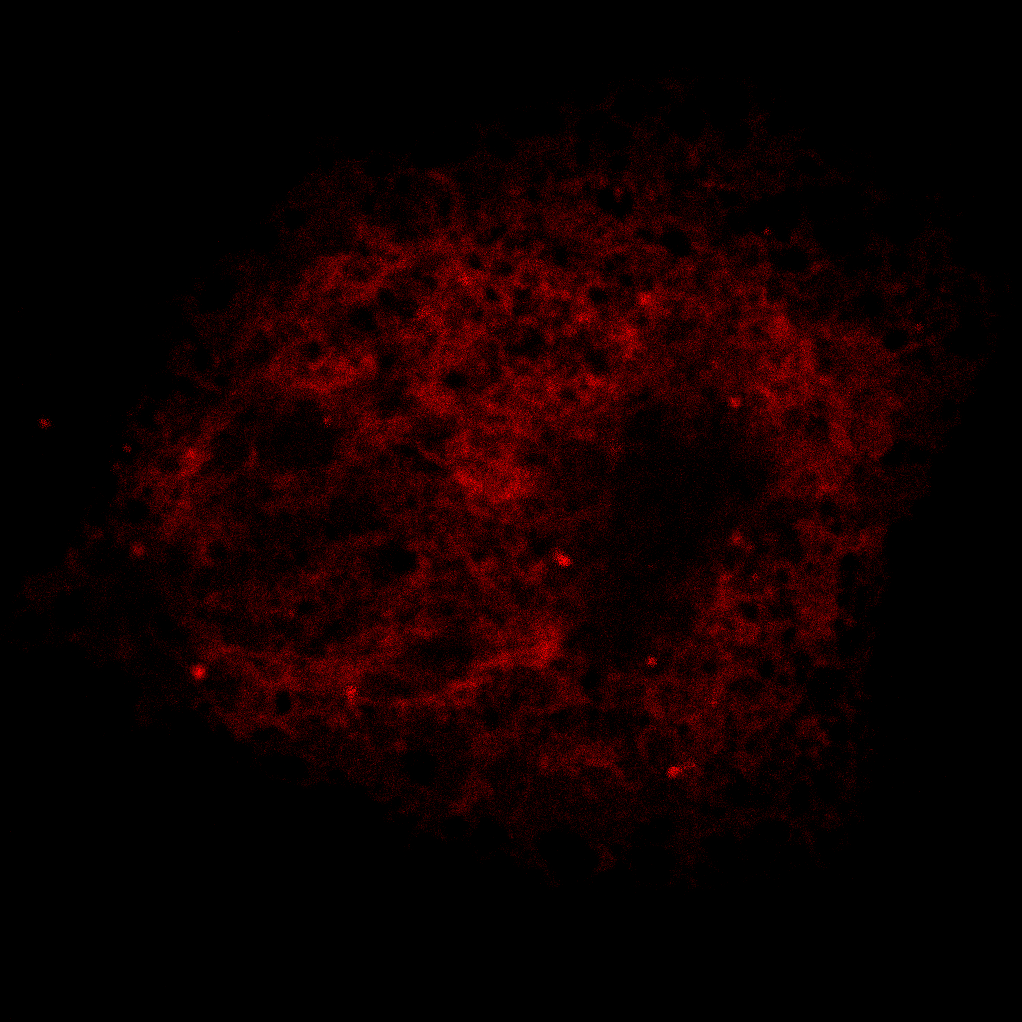

Supplement: Supplementary file 5 — Source data Fig. 3 [file 44318_2024_232_MOESM5_ESM.zip › Figure 3/Figure 3D/UBAC2 KO/RFP.tif]

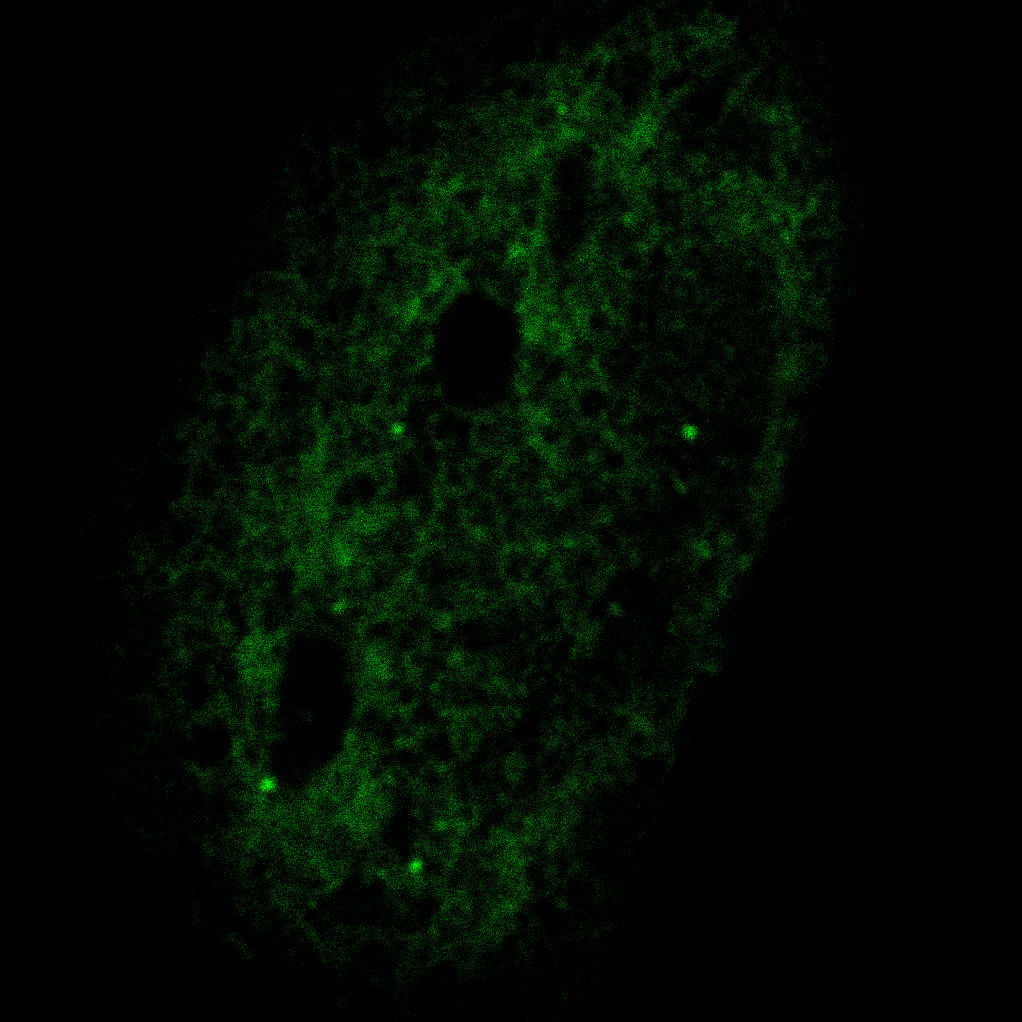

Supplement: Supplementary file 5 — Source data Fig. 3 [file 44318_2024_232_MOESM5_ESM.zip › Figure 3/Figure 3D/WT/GFP.tif]

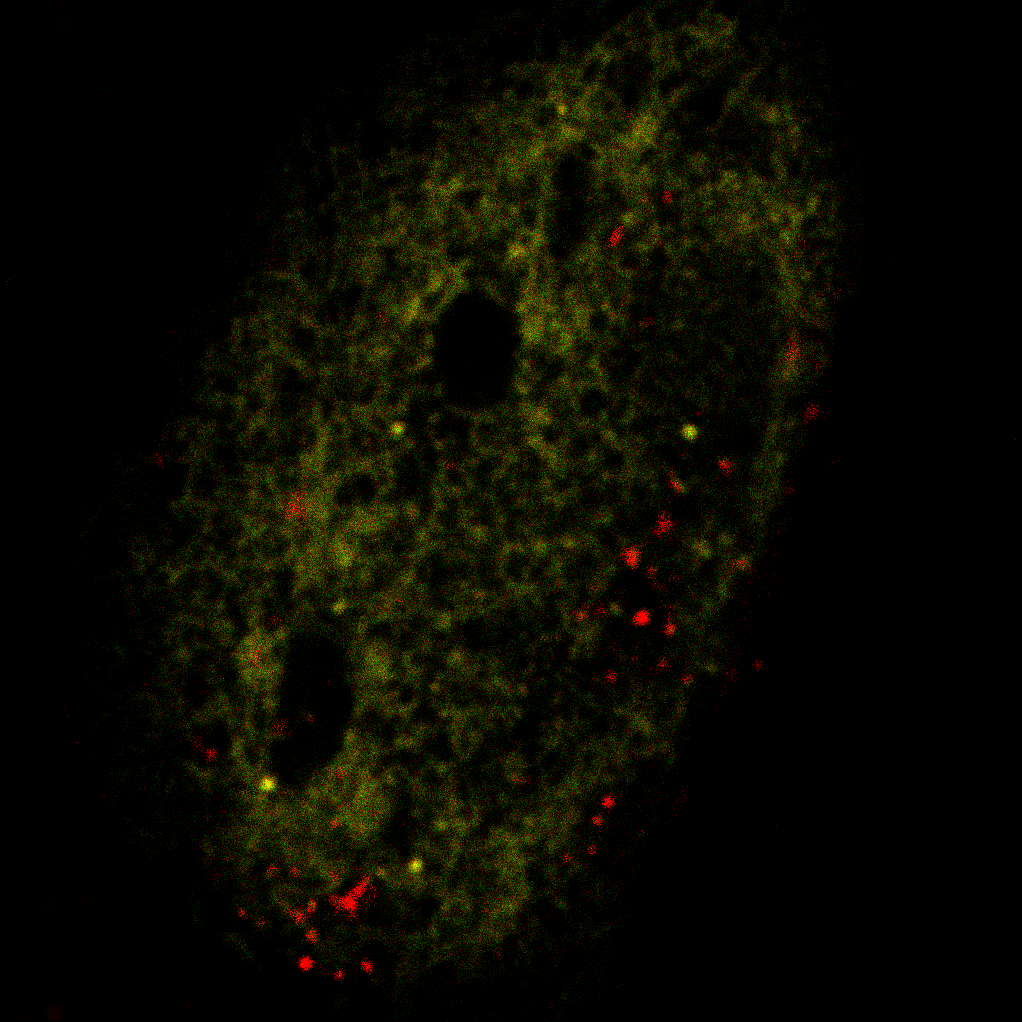

Supplement: Supplementary file 5 — Source data Fig. 3 [file 44318_2024_232_MOESM5_ESM.zip › Figure 3/Figure 3D/WT/Merge.tif]

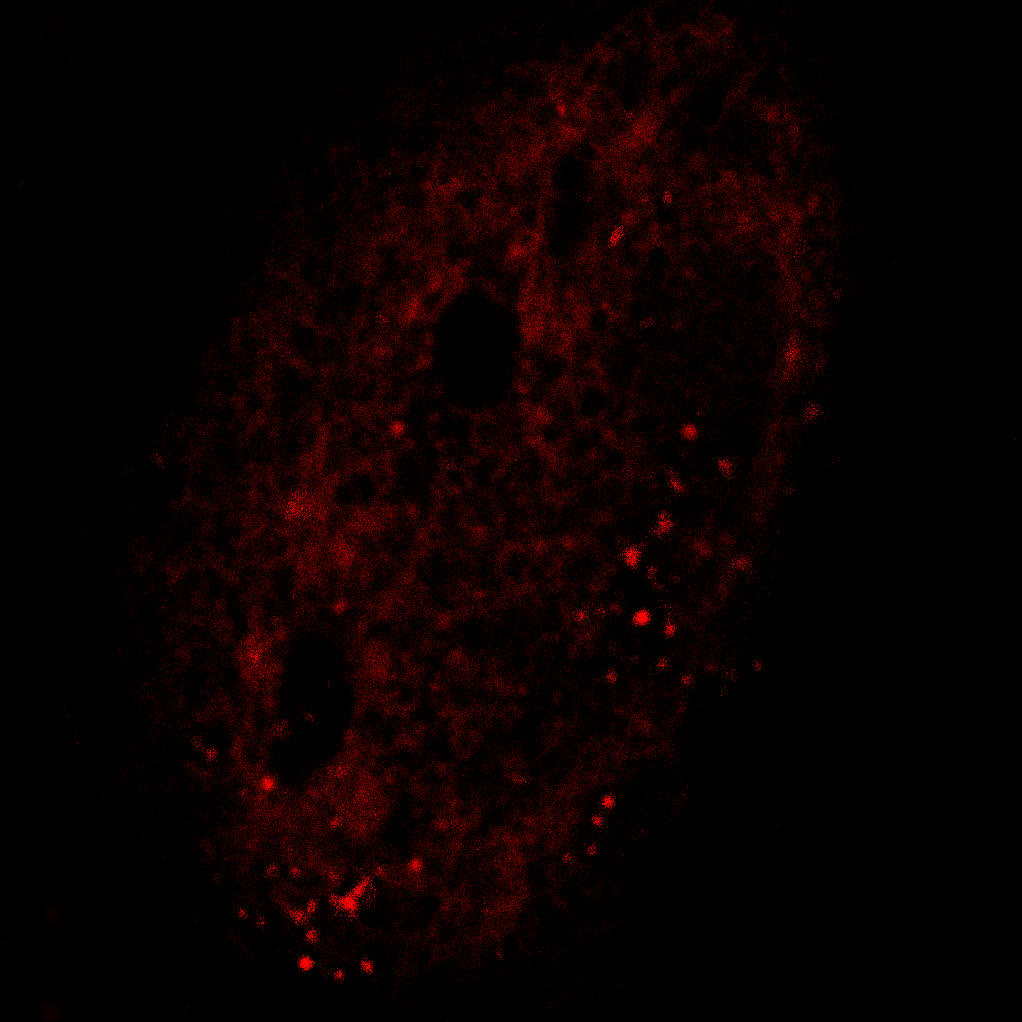

Supplement: Supplementary file 5 — Source data Fig. 3 [file 44318_2024_232_MOESM5_ESM.zip › Figure 3/Figure 3D/WT/RFP.tif]

Source data: Figure 3F.

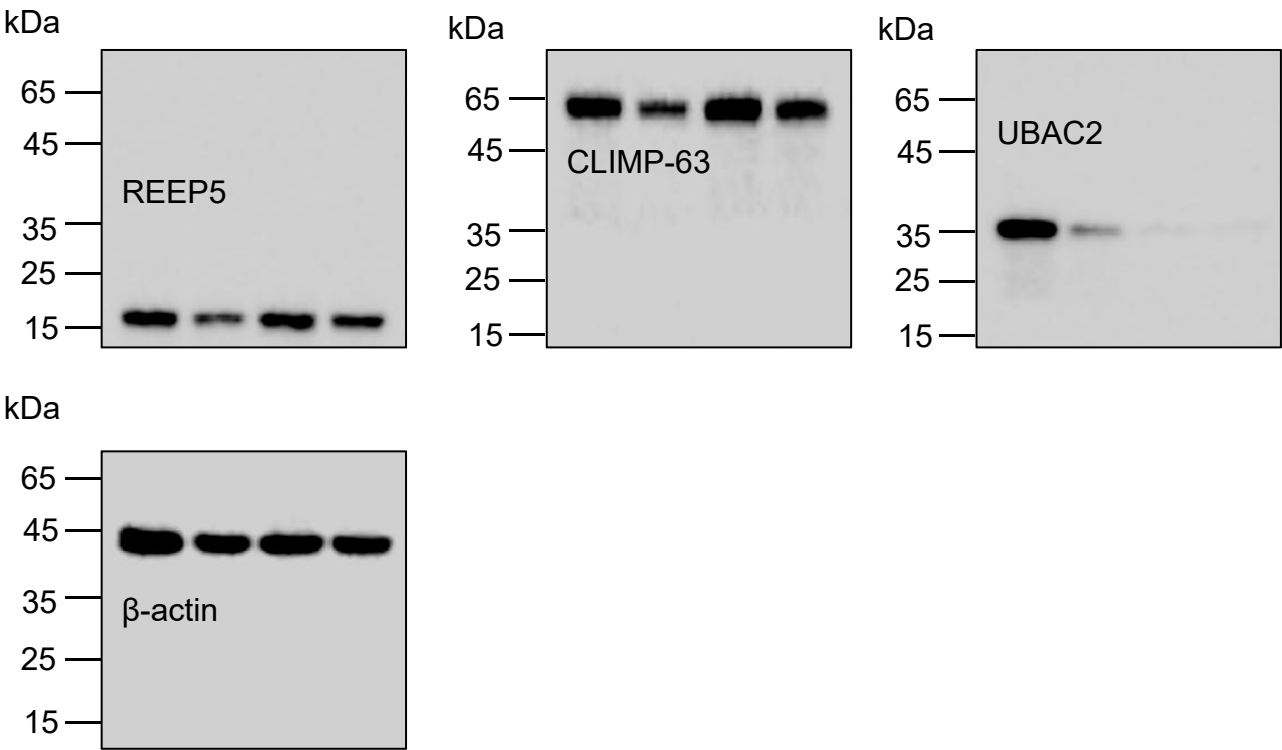

Supplement: Supplementary file 5 — Source data Fig. 3 [file 44318_2024_232_MOESM5_ESM.zip › Figure 3/Figure 3F.pdf]

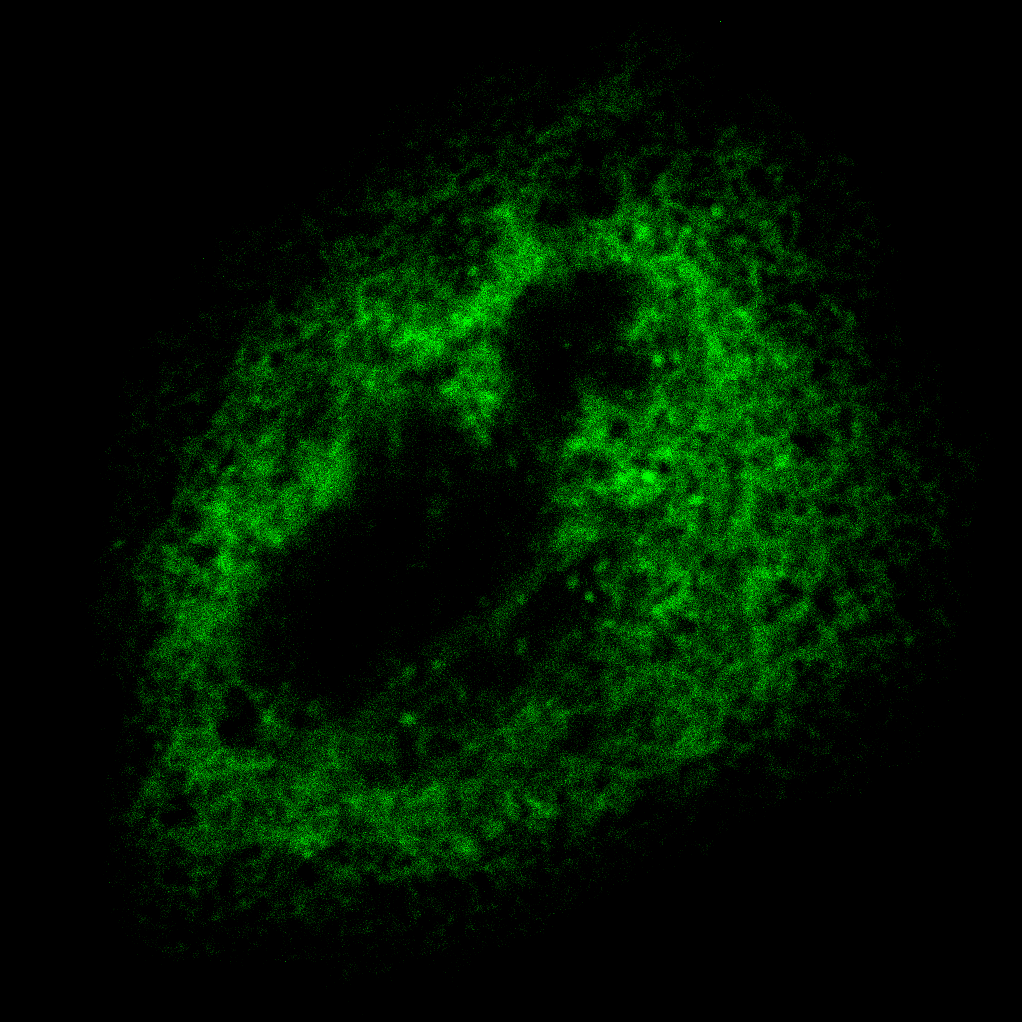

Supplement: Supplementary file 5 — Source data Fig. 3 [file 44318_2024_232_MOESM5_ESM.zip › Figure 3/Figure 3G/-/GFP.tif]

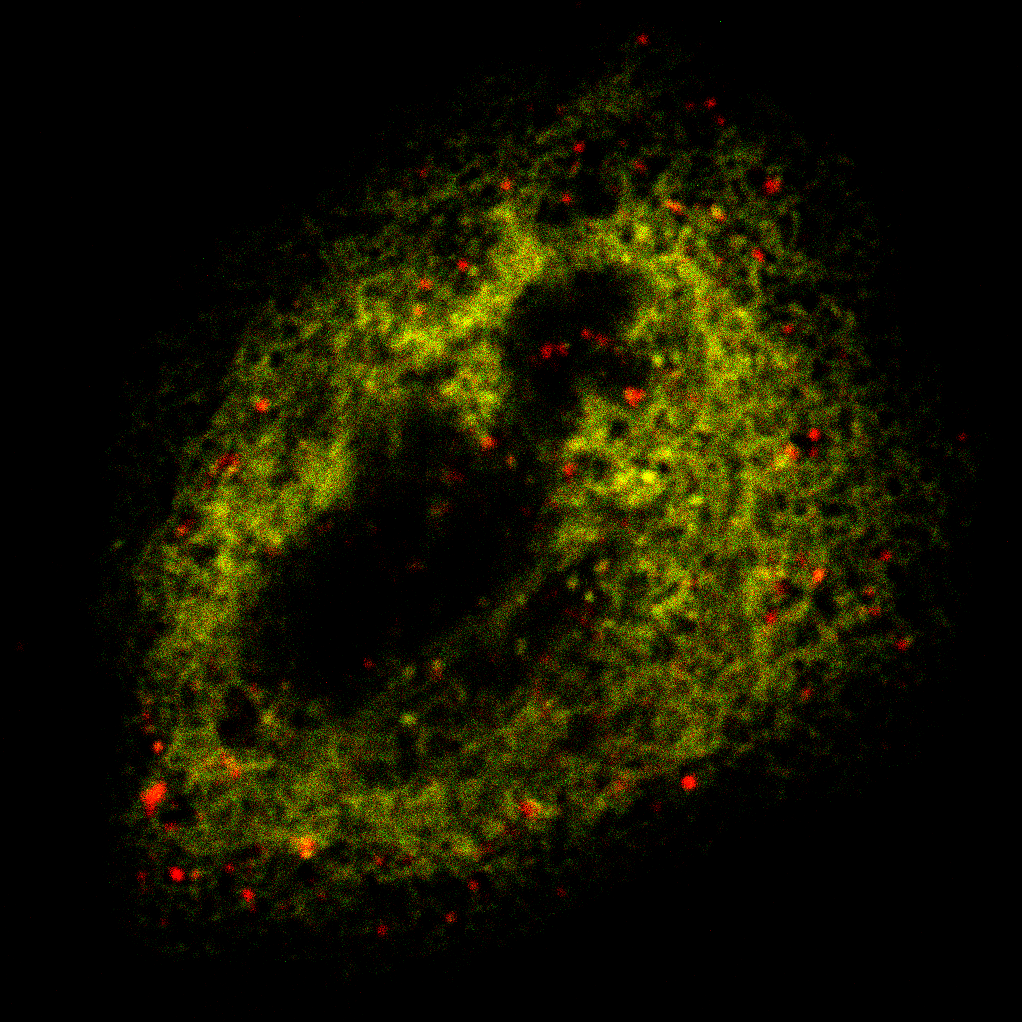

Supplement: Supplementary file 5 — Source data Fig. 3 [file 44318_2024_232_MOESM5_ESM.zip › Figure 3/Figure 3G/-/Merge.tif]

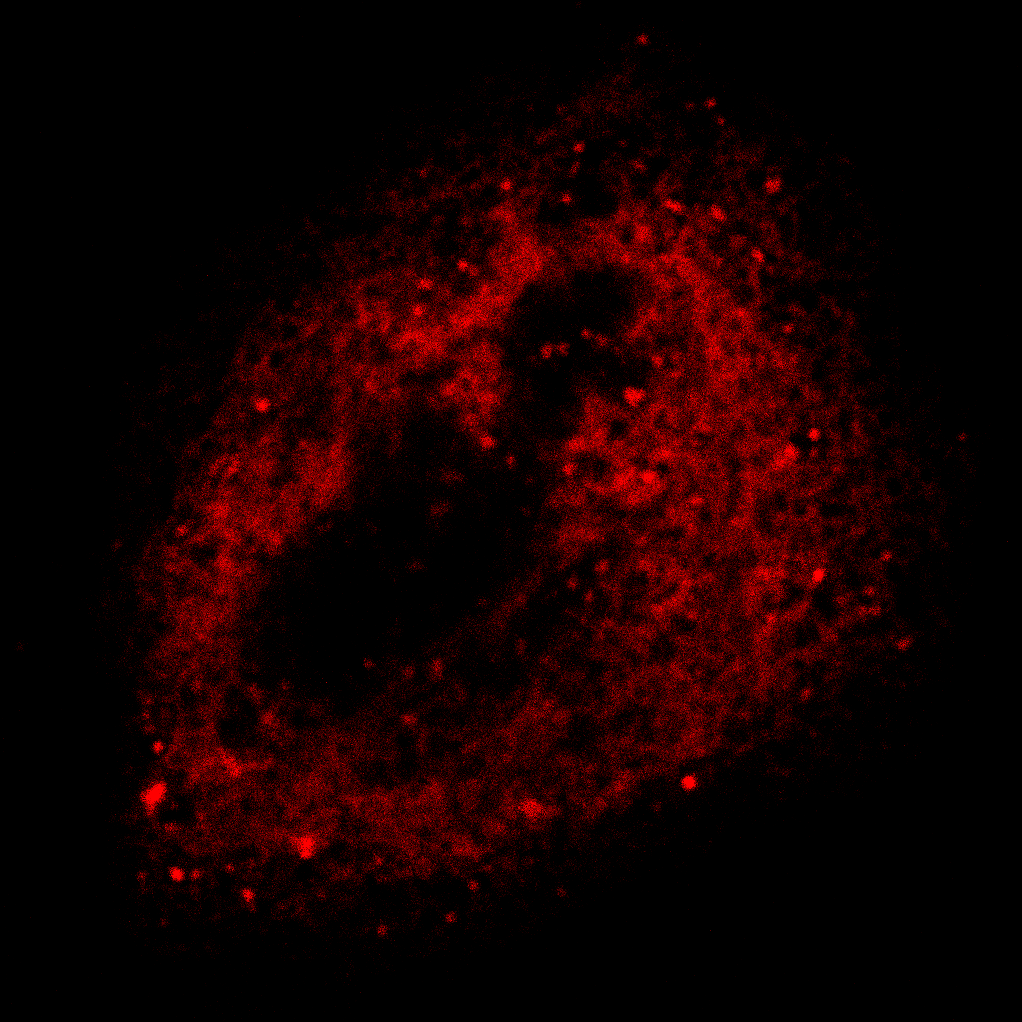

Supplement: Supplementary file 5 — Source data Fig. 3 [file 44318_2024_232_MOESM5_ESM.zip › Figure 3/Figure 3G/-/RFP.tif]

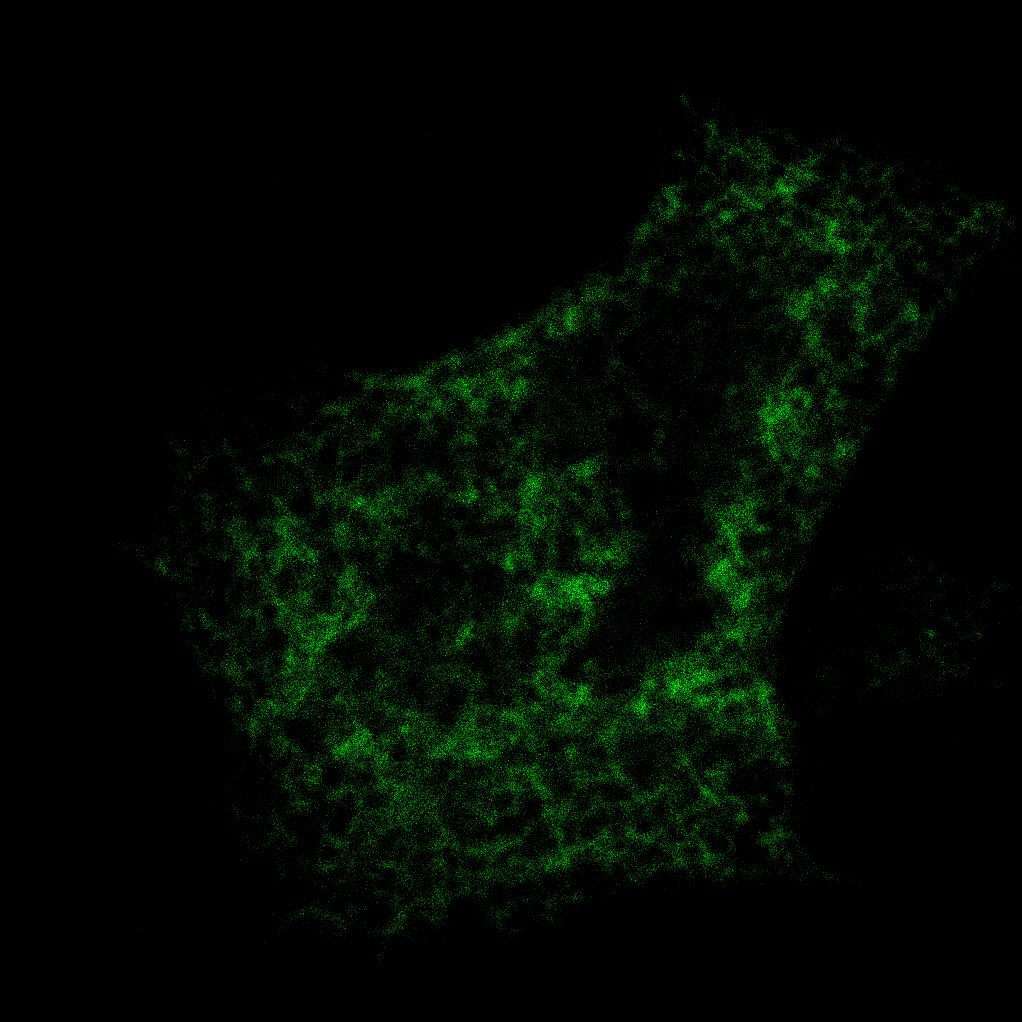

Supplement: Supplementary file 5 — Source data Fig. 3 [file 44318_2024_232_MOESM5_ESM.zip › Figure 3/Figure 3G/LIRM/GFP.tif]

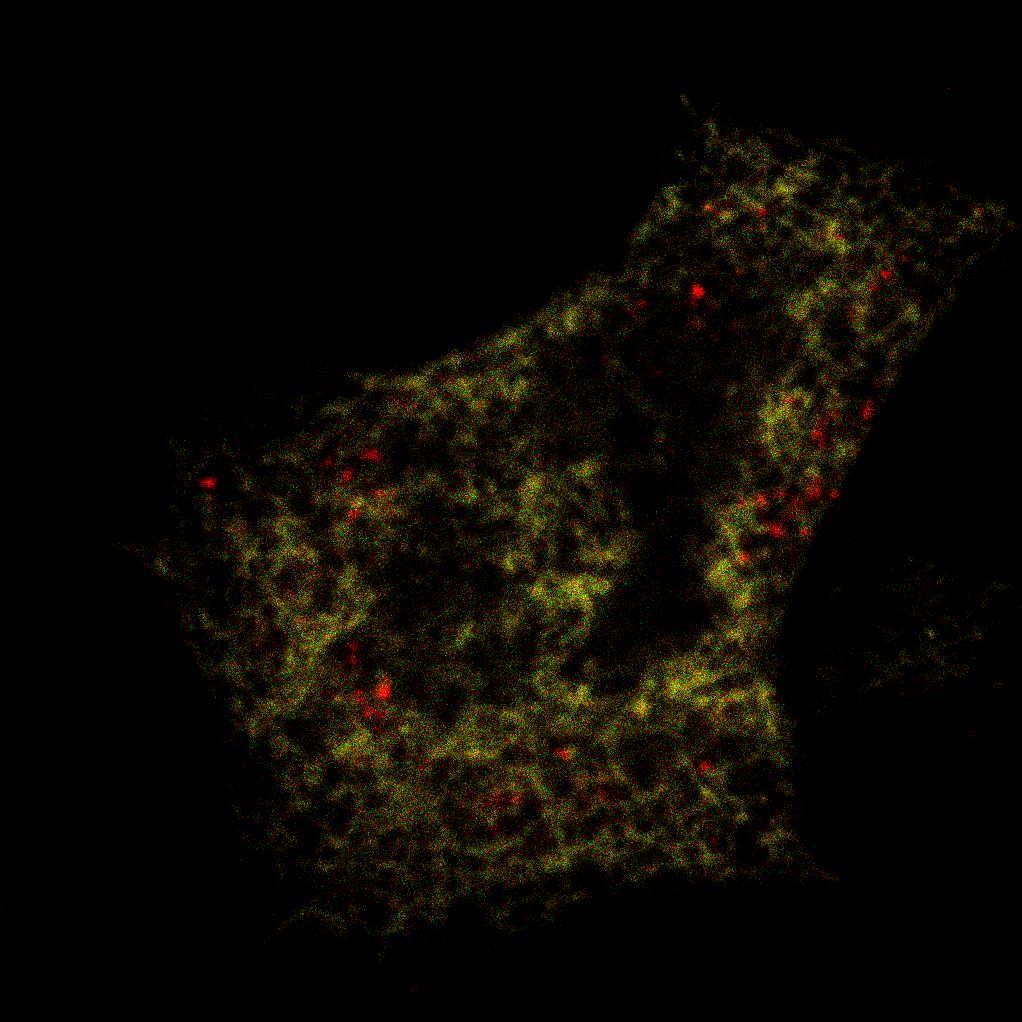

Supplement: Supplementary file 5 — Source data Fig. 3 [file 44318_2024_232_MOESM5_ESM.zip › Figure 3/Figure 3G/LIRM/Merge.tif]

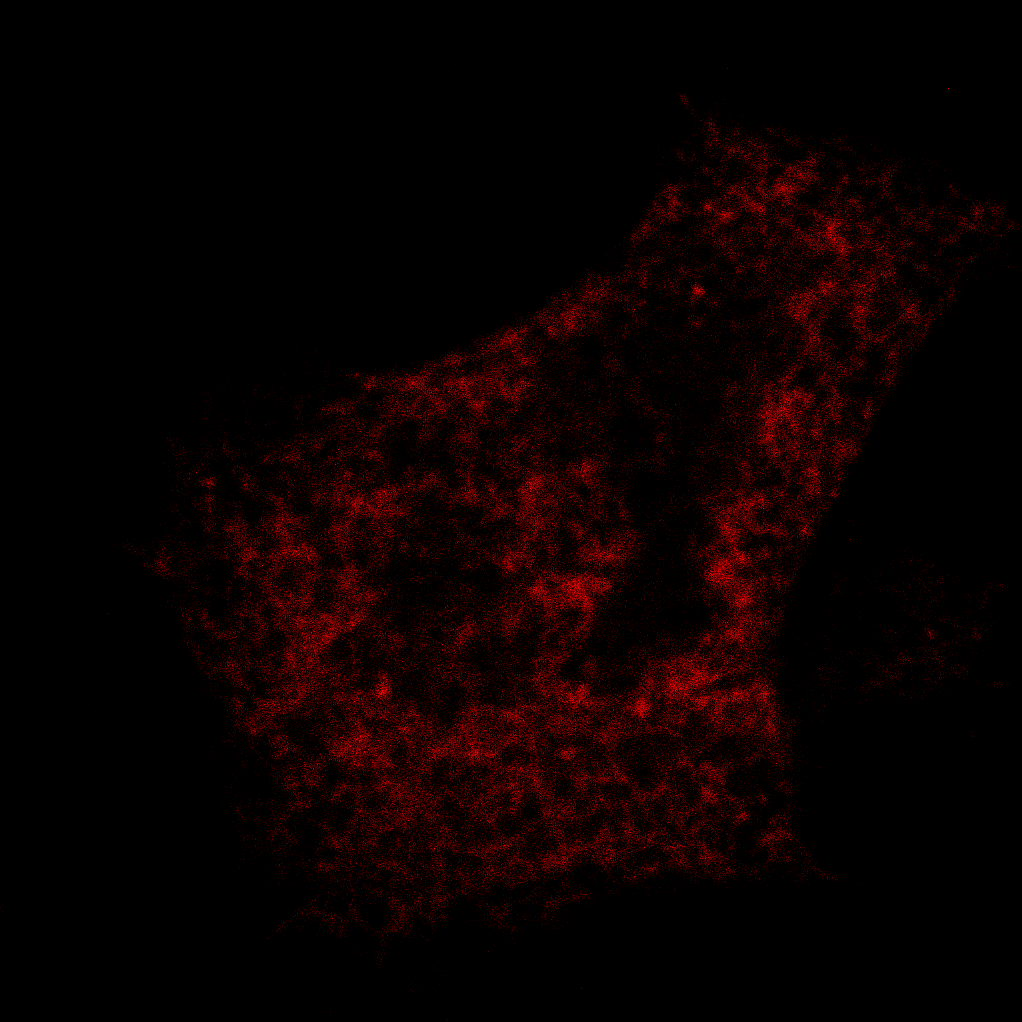

Supplement: Supplementary file 5 — Source data Fig. 3 [file 44318_2024_232_MOESM5_ESM.zip › Figure 3/Figure 3G/LIRM/RFP.tif]

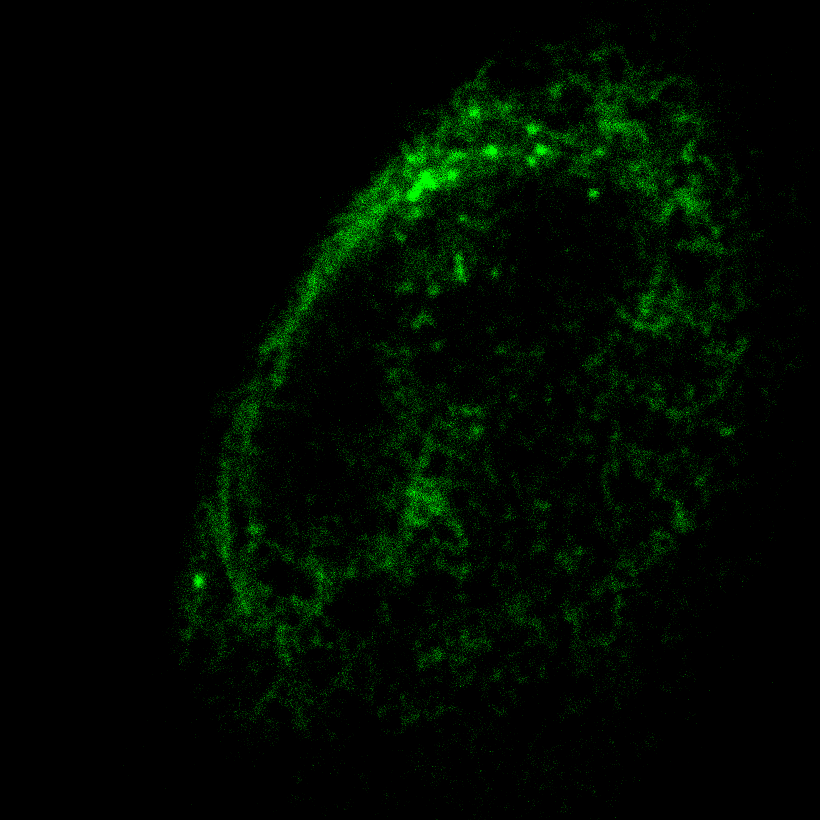

Supplement: Supplementary file 5 — Source data Fig. 3 [file 44318_2024_232_MOESM5_ESM.zip › Figure 3/Figure 3G/WT/GFP.tif]

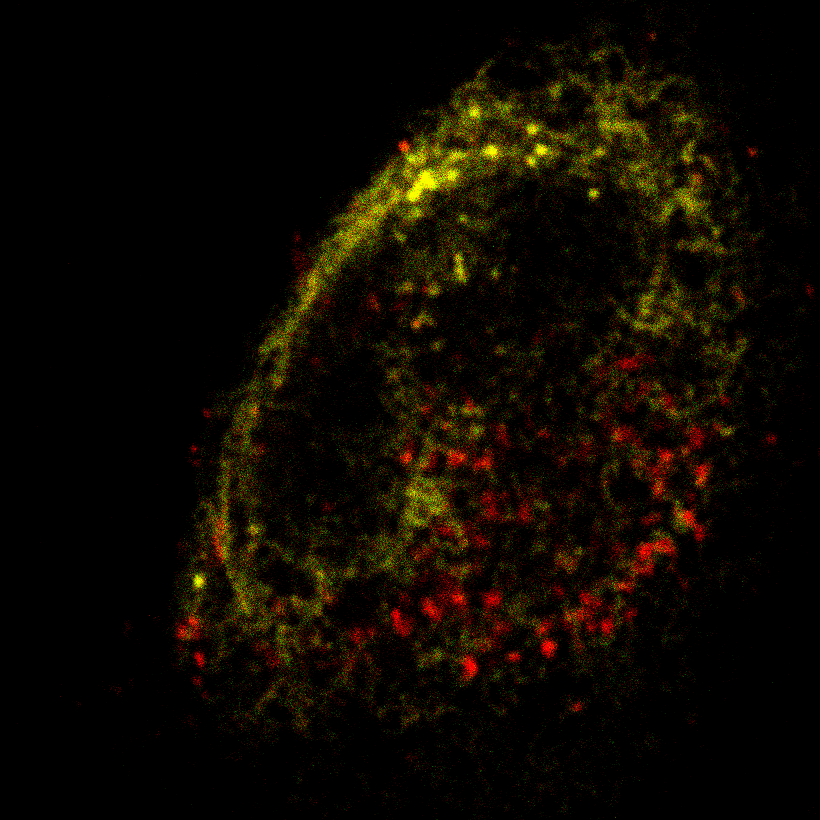

Supplement: Supplementary file 5 — Source data Fig. 3 [file 44318_2024_232_MOESM5_ESM.zip › Figure 3/Figure 3G/WT/Merge.tif]

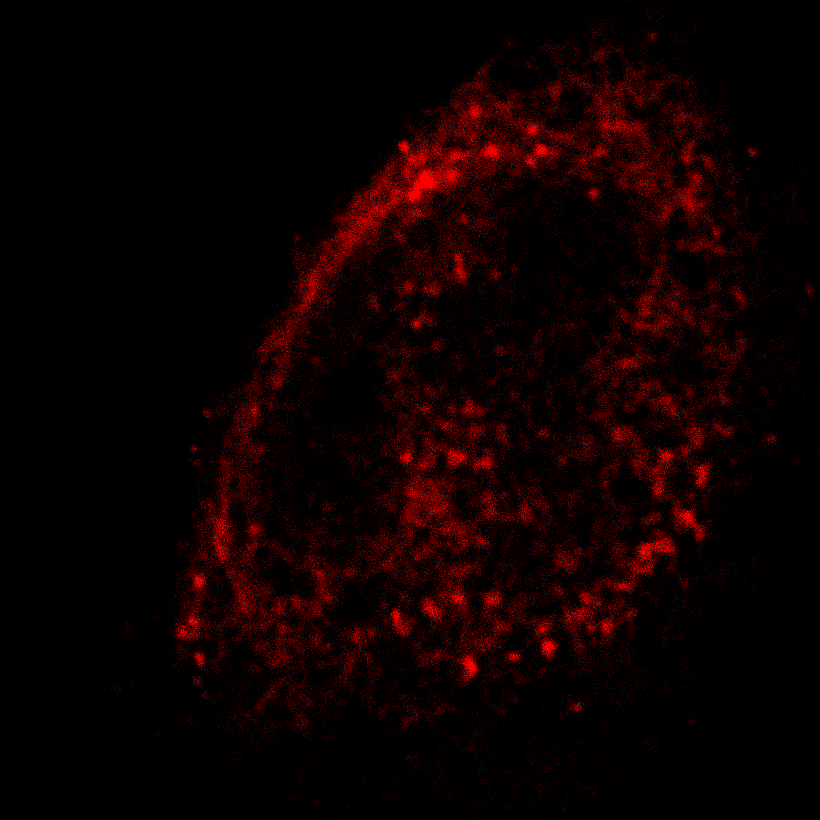

Supplement: Supplementary file 5 — Source data Fig. 3 [file 44318_2024_232_MOESM5_ESM.zip › Figure 3/Figure 3G/WT/RFP.tif]

Source data: Figure 3l.

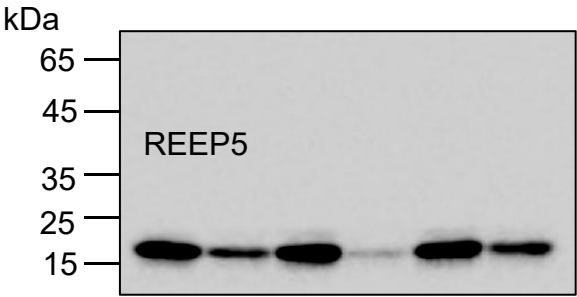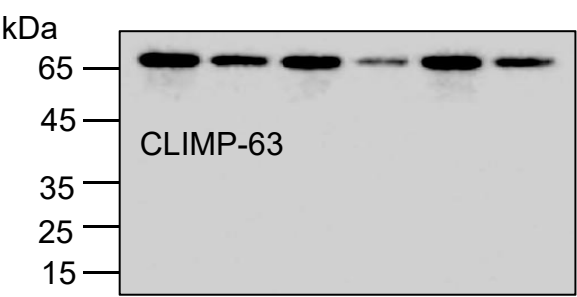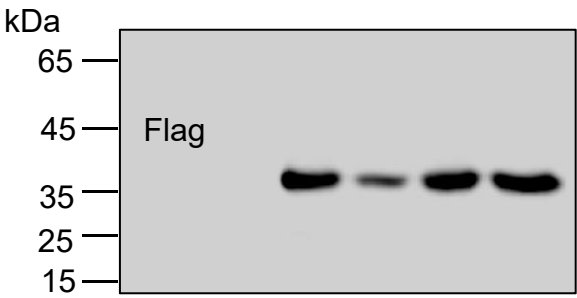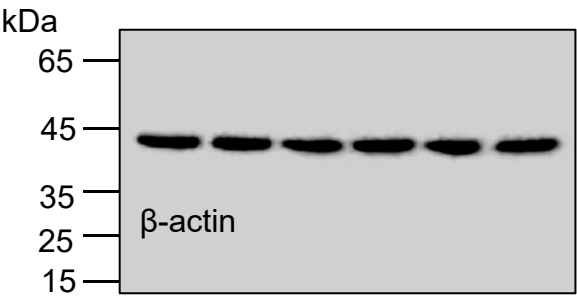

Supplement: Supplementary file 5 — Source data Fig. 3 [file 44318_2024_232_MOESM5_ESM.zip › Figure 3/Figure 3I.pdf]

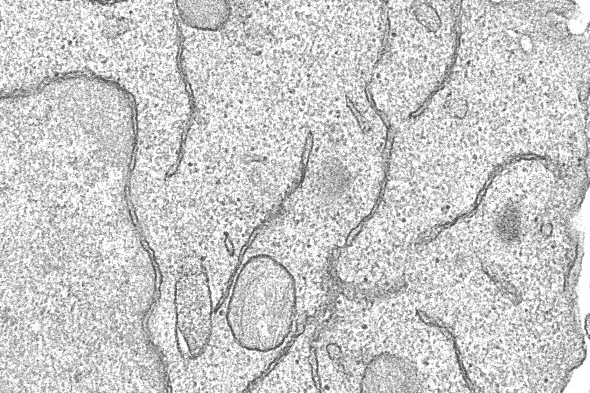

Supplement: Supplementary file 5 — Source data Fig. 3 [file 44318_2024_232_MOESM5_ESM.zip › Figure 3/Figure 3J/UBAC2 KO/Flag-EV.tif]

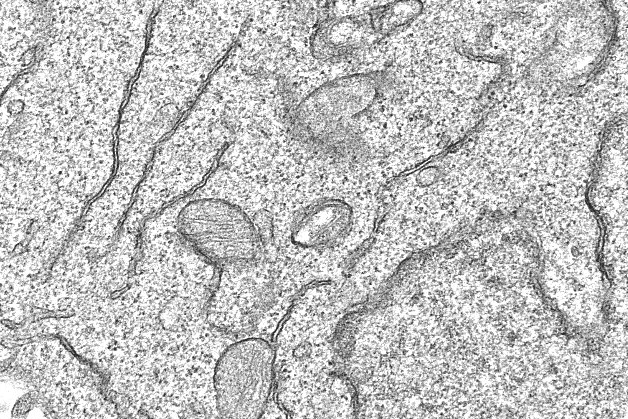

Supplement: Supplementary file 5 — Source data Fig. 3 [file 44318_2024_232_MOESM5_ESM.zip › Figure 3/Figure 3J/UBAC2 KO/Flag-UBAC2 LIRm.tif]

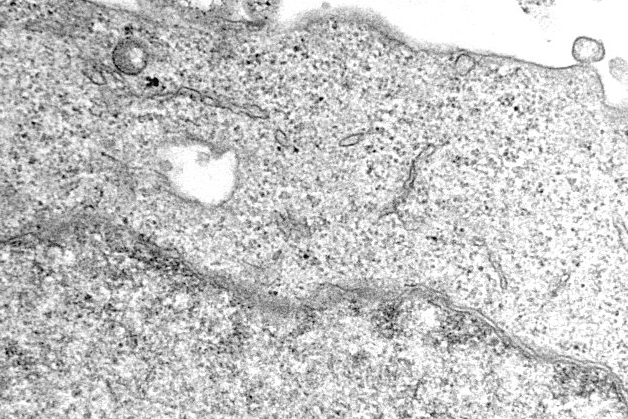

Supplement: Supplementary file 5 — Source data Fig. 3 [file 44318_2024_232_MOESM5_ESM.zip › Figure 3/Figure 3J/UBAC2 KO/Flag-UBAC2 WT.tif]

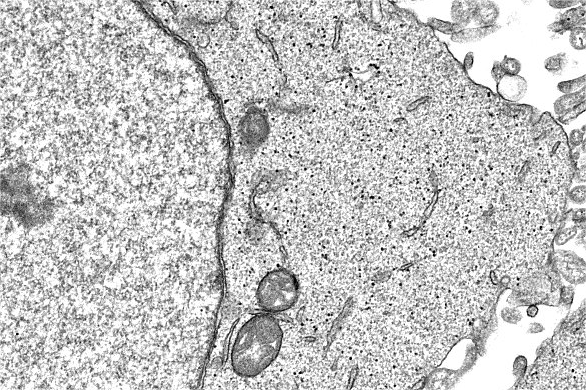

Supplement: Supplementary file 5 — Source data Fig. 3 [file 44318_2024_232_MOESM5_ESM.zip › Figure 3/Figure 3J/WT/Flag-EV.png]

Source data: Figure 3K.

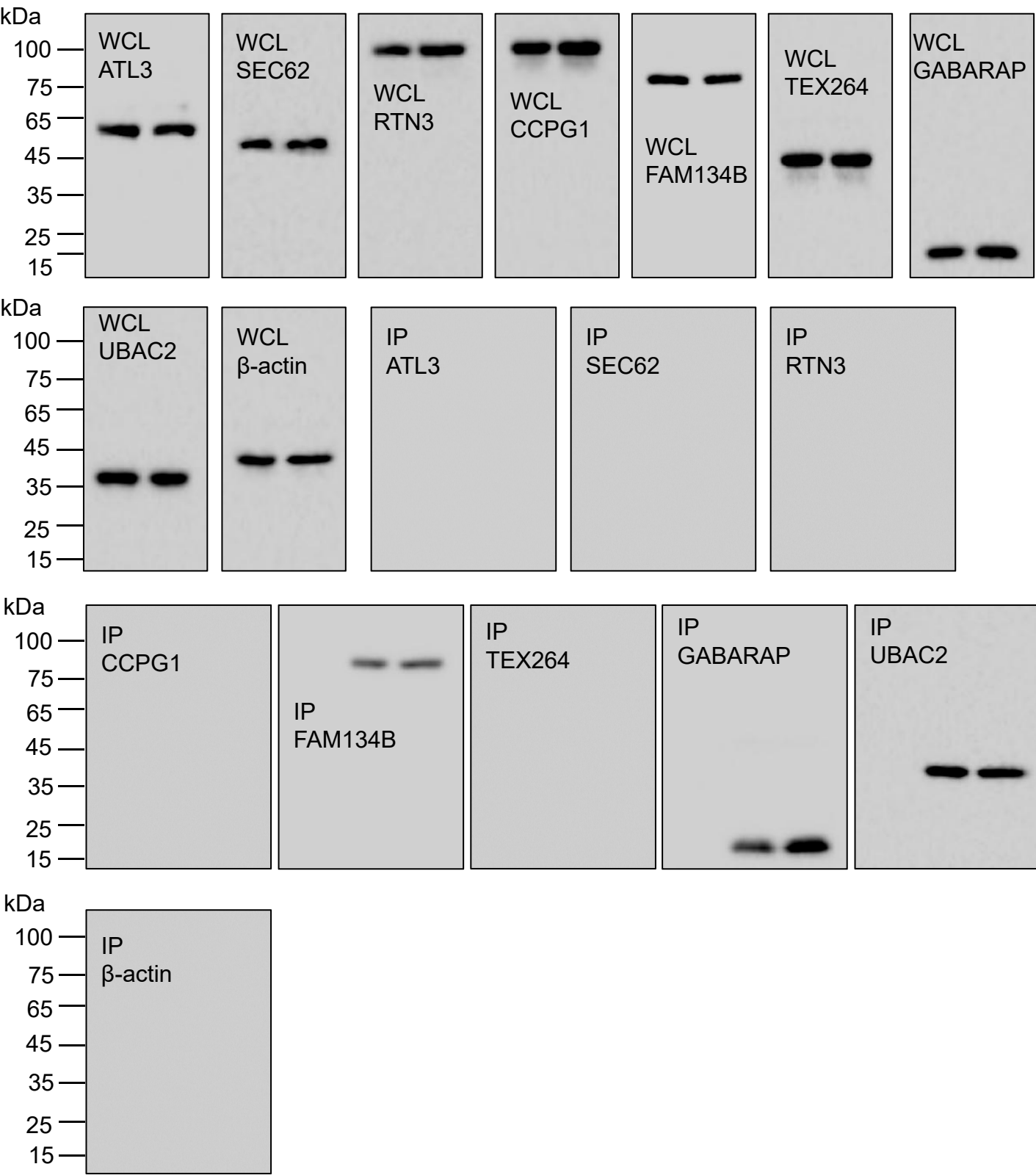

Supplement: Supplementary file 5 — Source data Fig. 3 [file 44318_2024_232_MOESM5_ESM.zip › Figure 3/Figure 3K.pdf]

Source data: Figure 3L.

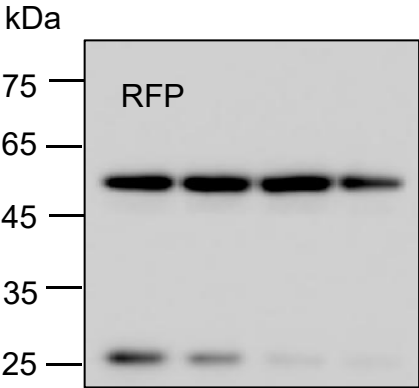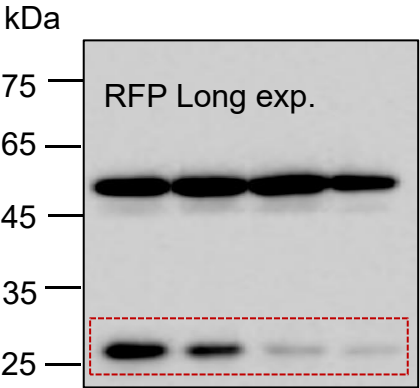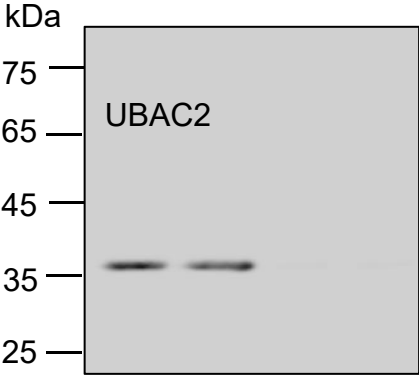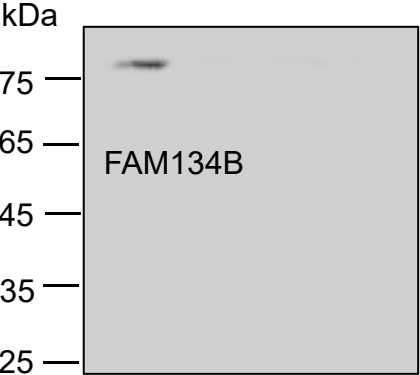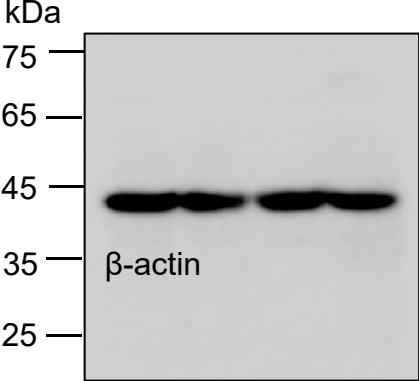

Supplement: Supplementary file 5 — Source data Fig. 3 [file 44318_2024_232_MOESM5_ESM.zip › Figure 3/Figure 3L.pdf]

Source data: Figure 4B.

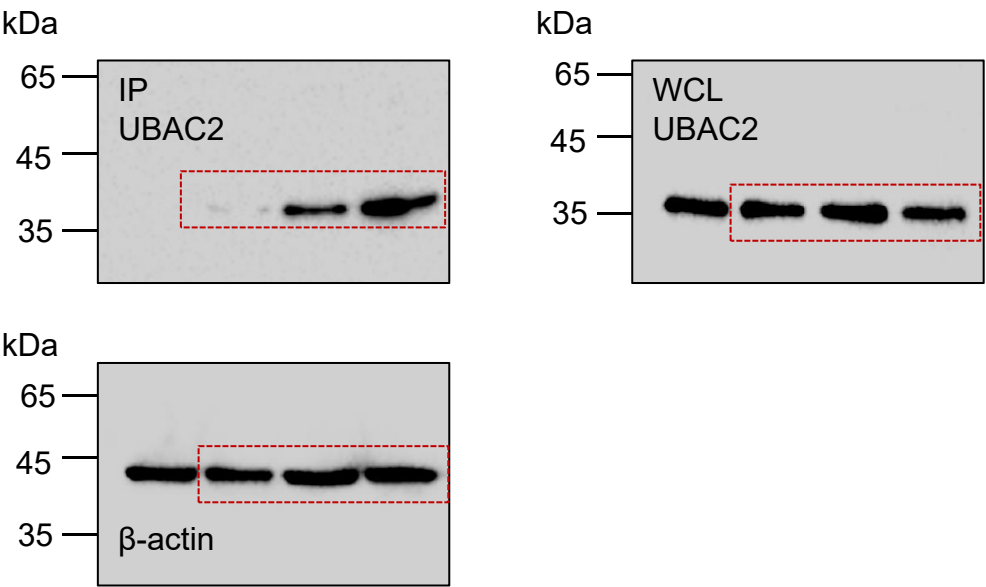

Supplement: Supplementary file 6 — Source data Fig. 4 [file 44318_2024_232_MOESM6_ESM.zip › Figure 4/Figure 4B.pdf]

Source data: Figure 4C.

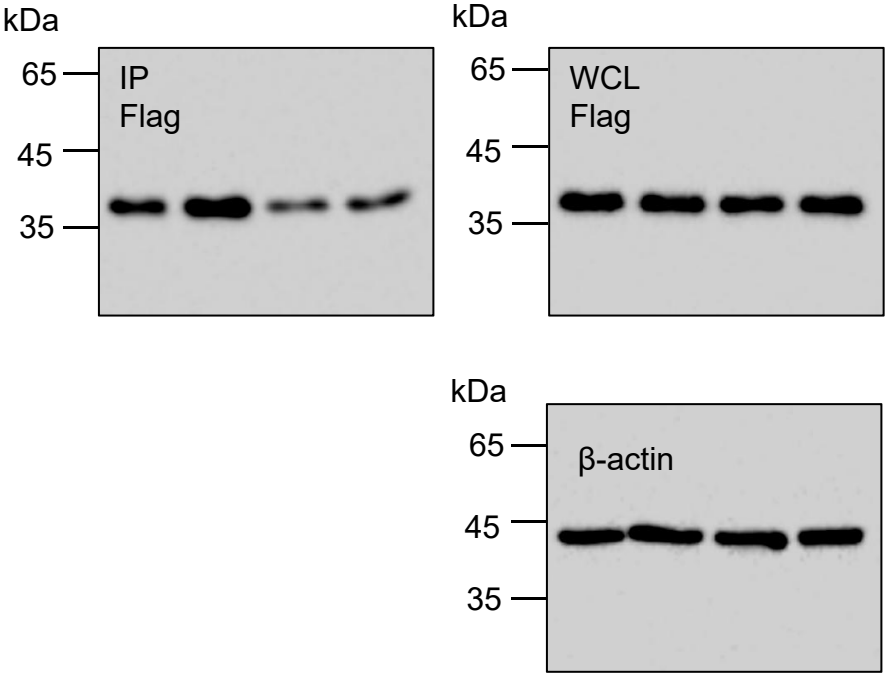

Supplement: Supplementary file 6 — Source data Fig. 4 [file 44318_2024_232_MOESM6_ESM.zip › Figure 4/Figure 4C.pdf]

Source data: Figure 4D.

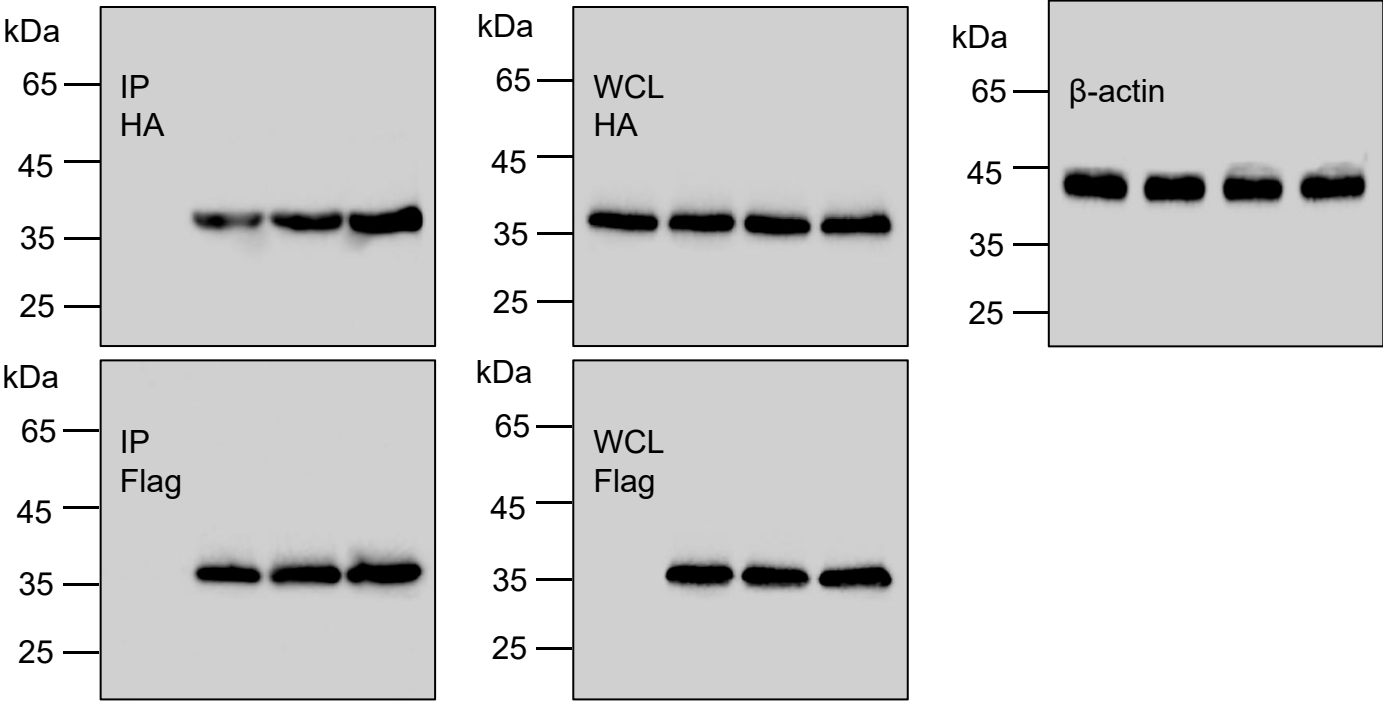

Supplement: Supplementary file 6 — Source data Fig. 4 [file 44318_2024_232_MOESM6_ESM.zip › Figure 4/Figure 4D.pdf]

Source data: Figure 4E.

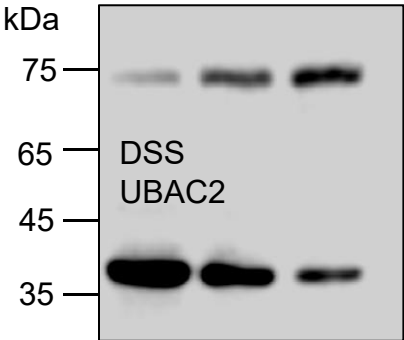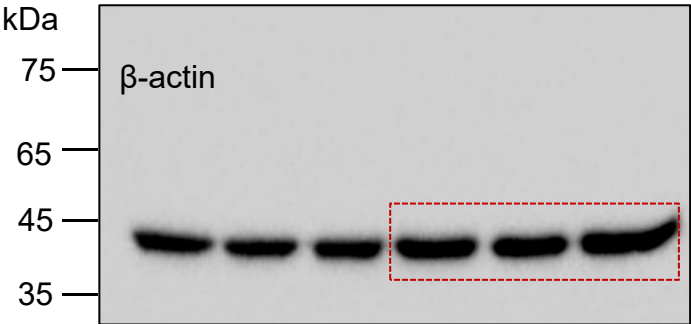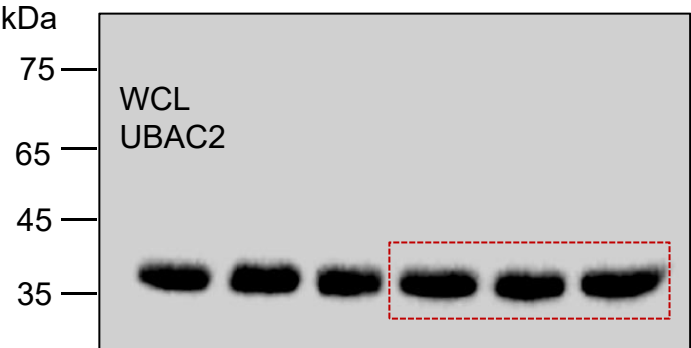

Supplement: Supplementary file 6 — Source data Fig. 4 [file 44318_2024_232_MOESM6_ESM.zip › Figure 4/Figure 4E.pdf]

Source data: Figure 4F.

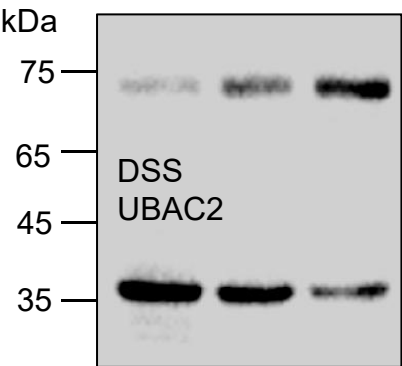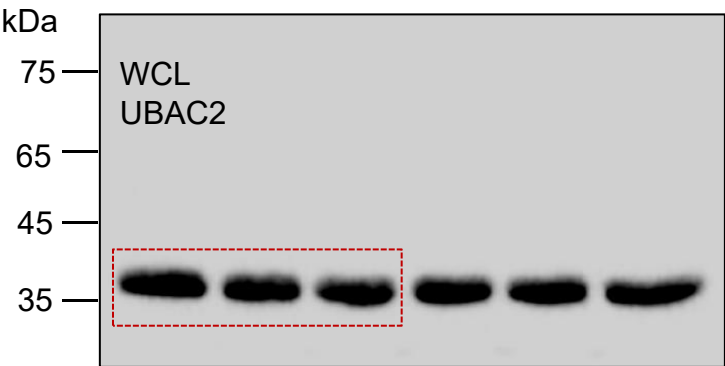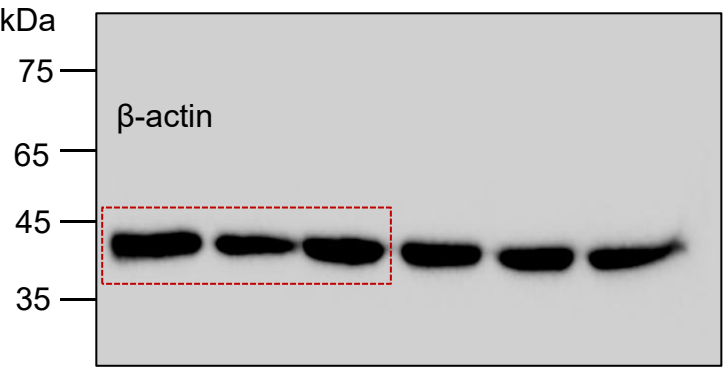

Supplement: Supplementary file 6 — Source data Fig. 4 [file 44318_2024_232_MOESM6_ESM.zip › Figure 4/Figure 4F.pdf]

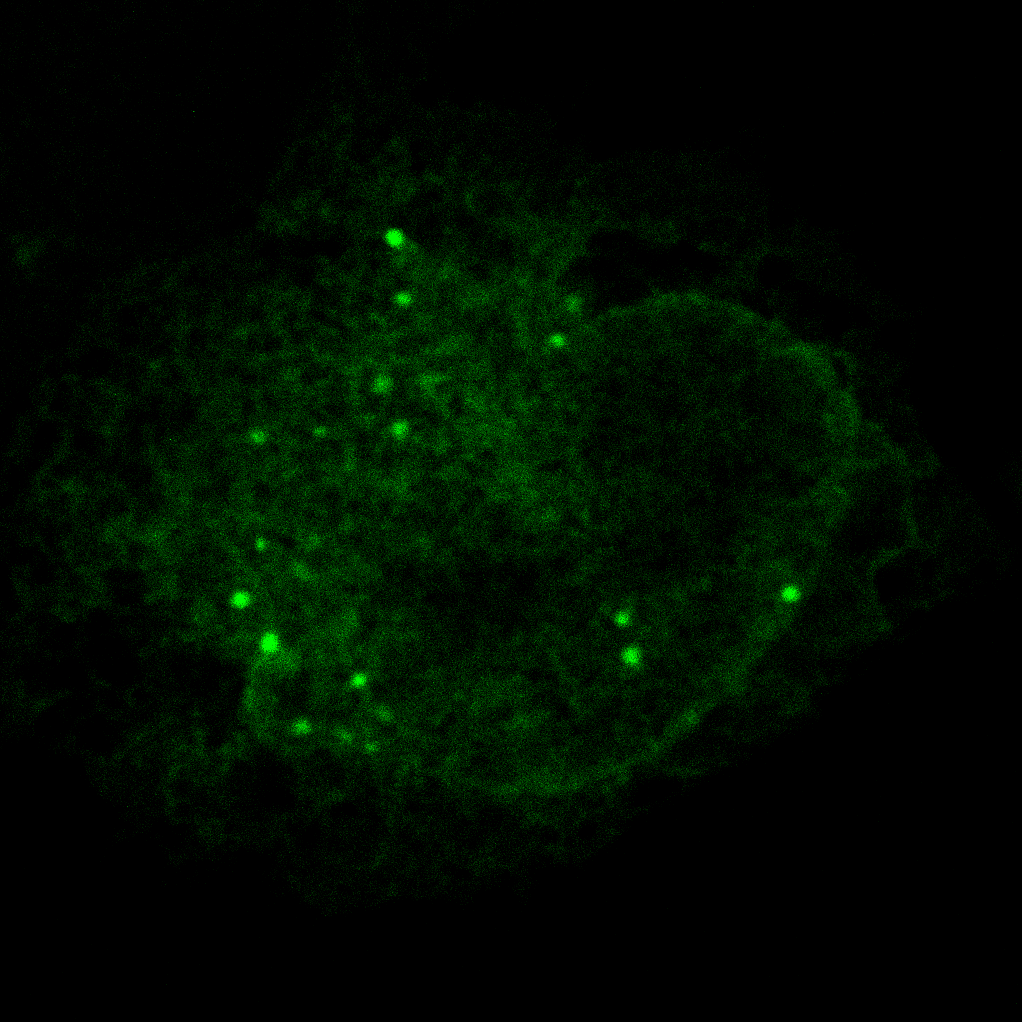

Supplement: Supplementary file 6 — Source data Fig. 4 [file 44318_2024_232_MOESM6_ESM.zip › Figure 4/Figure 4G/Baf A1.tif]

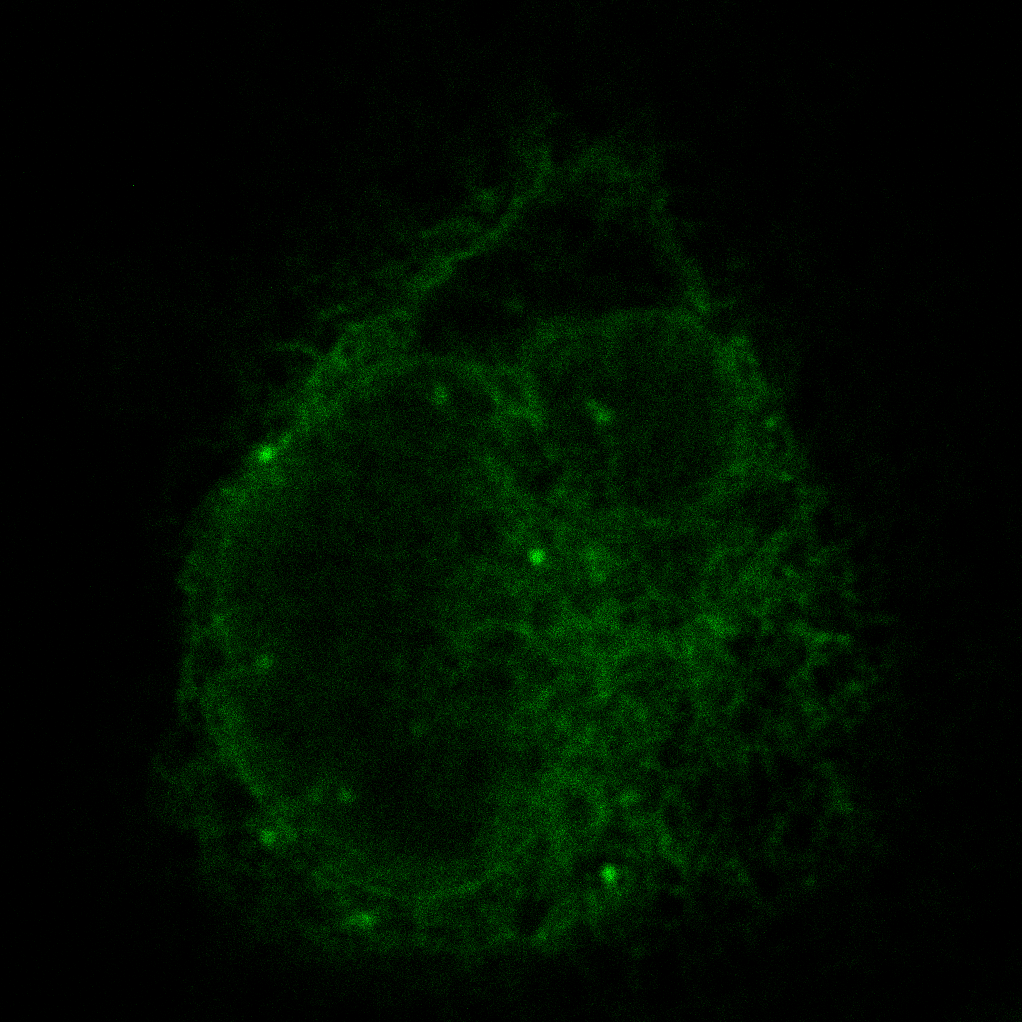

Supplement: Supplementary file 6 — Source data Fig. 4 [file 44318_2024_232_MOESM6_ESM.zip › Figure 4/Figure 4G/Mock.tif]

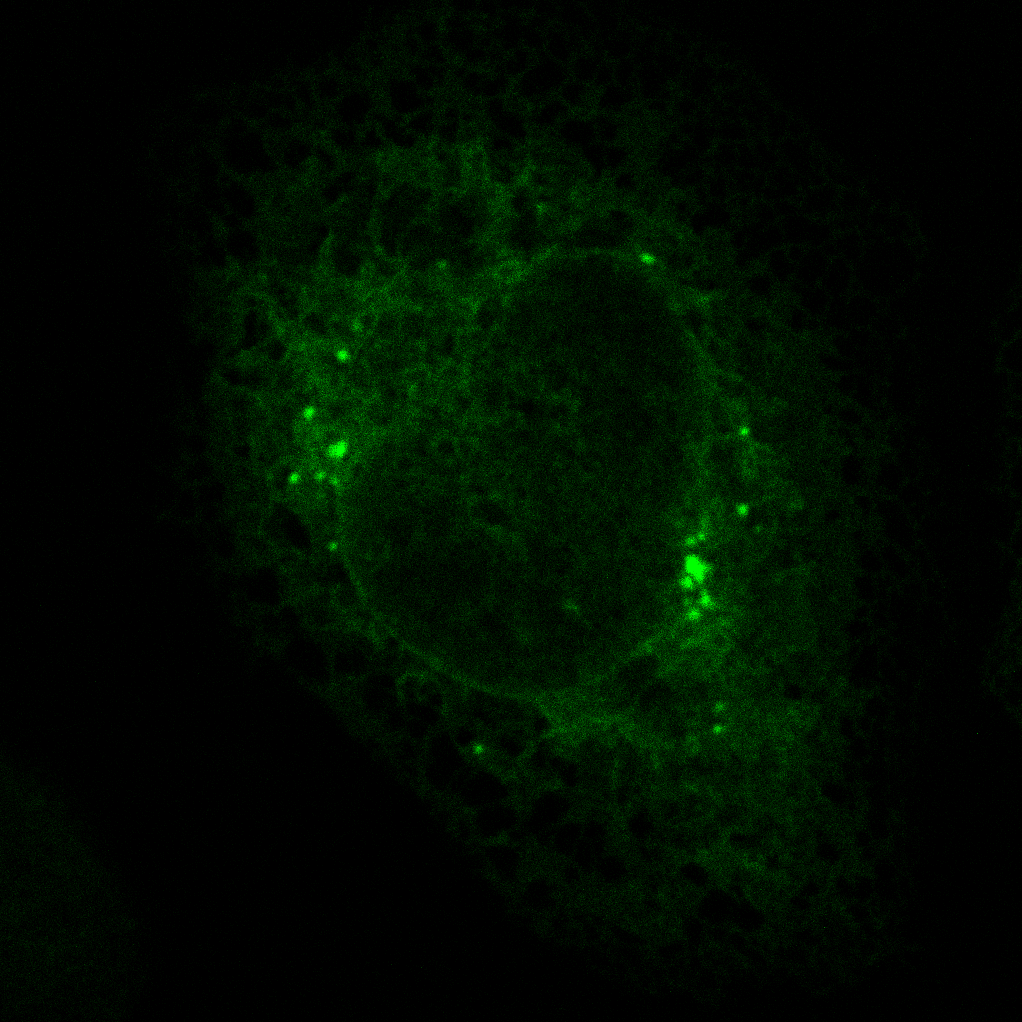

Supplement: Supplementary file 6 — Source data Fig. 4 [file 44318_2024_232_MOESM6_ESM.zip › Figure 4/Figure 4I/Mock.tif]

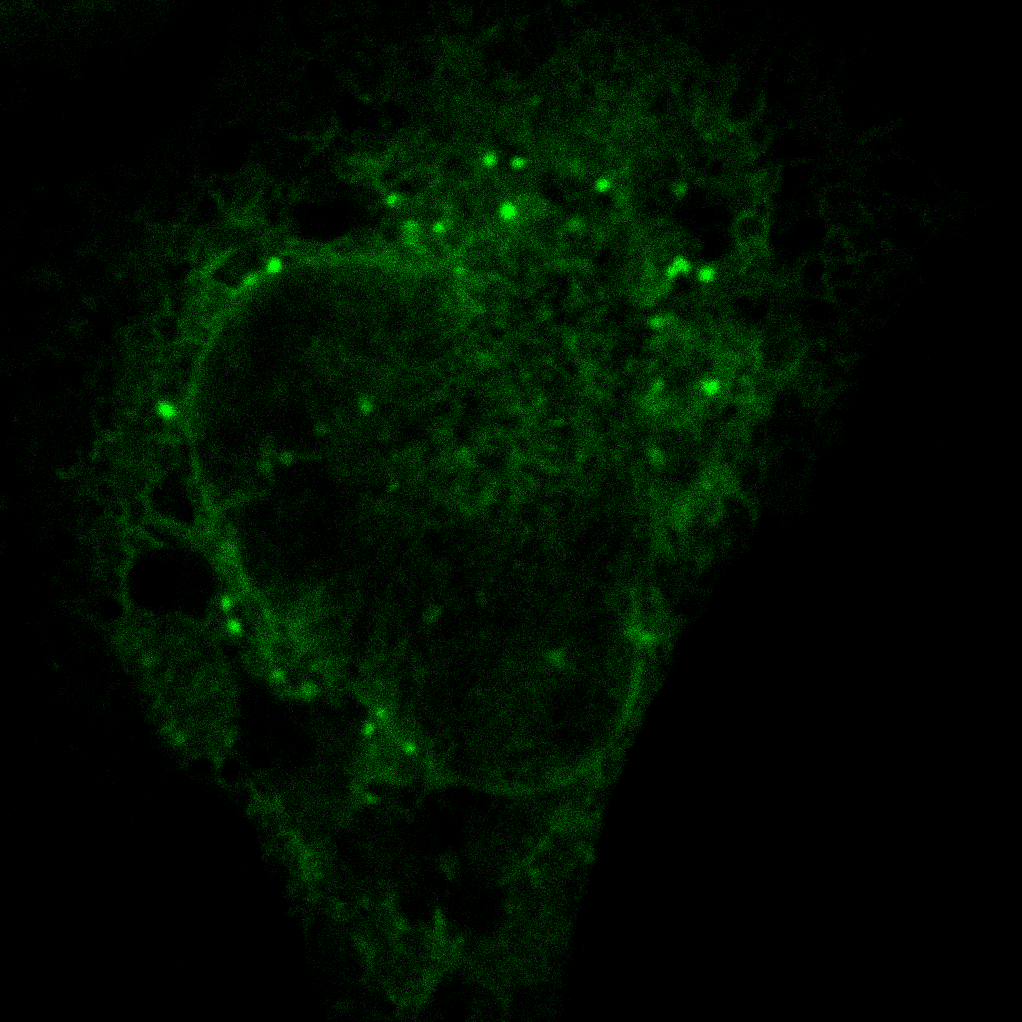

Supplement: Supplementary file 6 — Source data Fig. 4 [file 44318_2024_232_MOESM6_ESM.zip › Figure 4/Figure 4I/TG.tif]

Source data: Figure 4K.

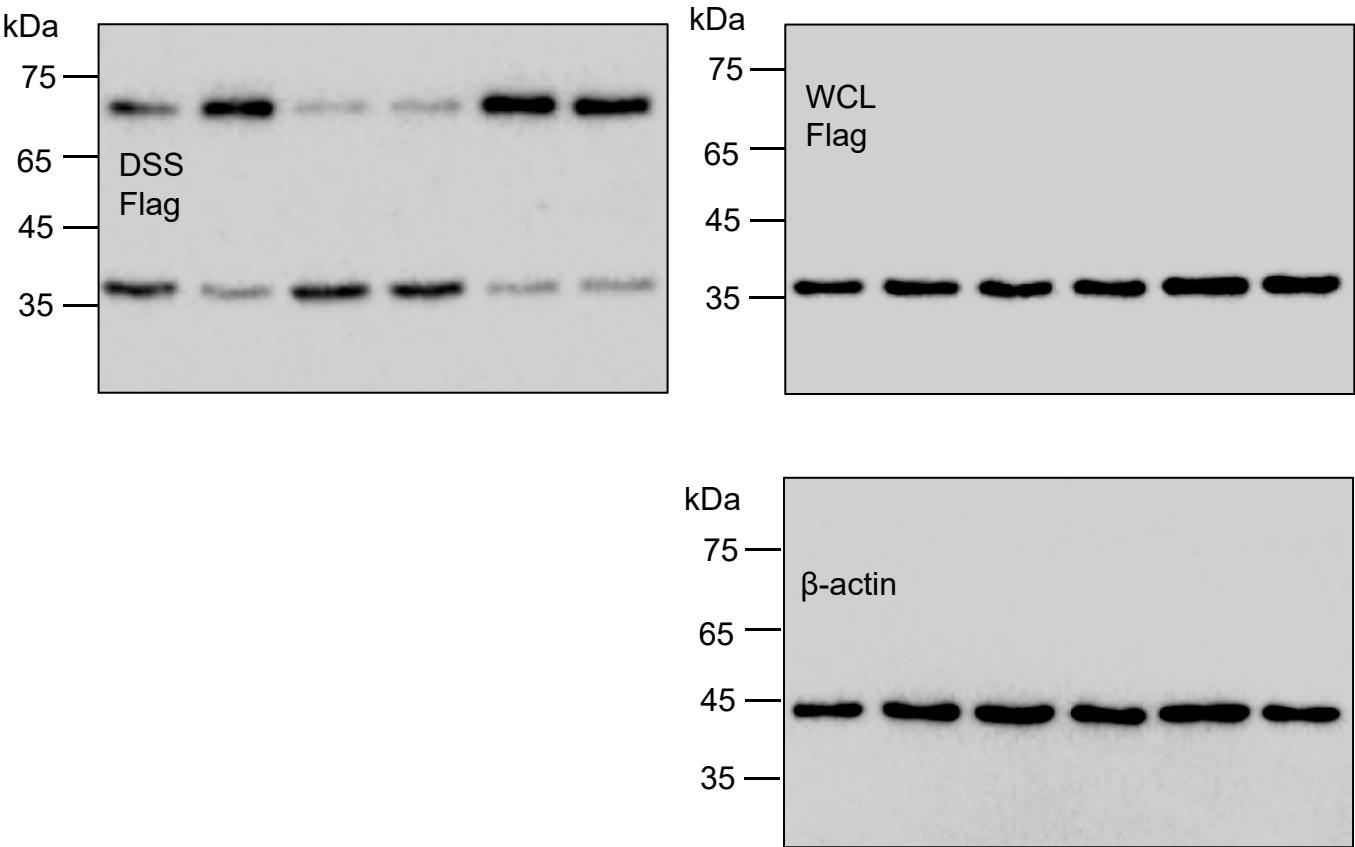

Supplement: Supplementary file 6 — Source data Fig. 4 [file 44318_2024_232_MOESM6_ESM.zip › Figure 4/Figure 4K.pdf]

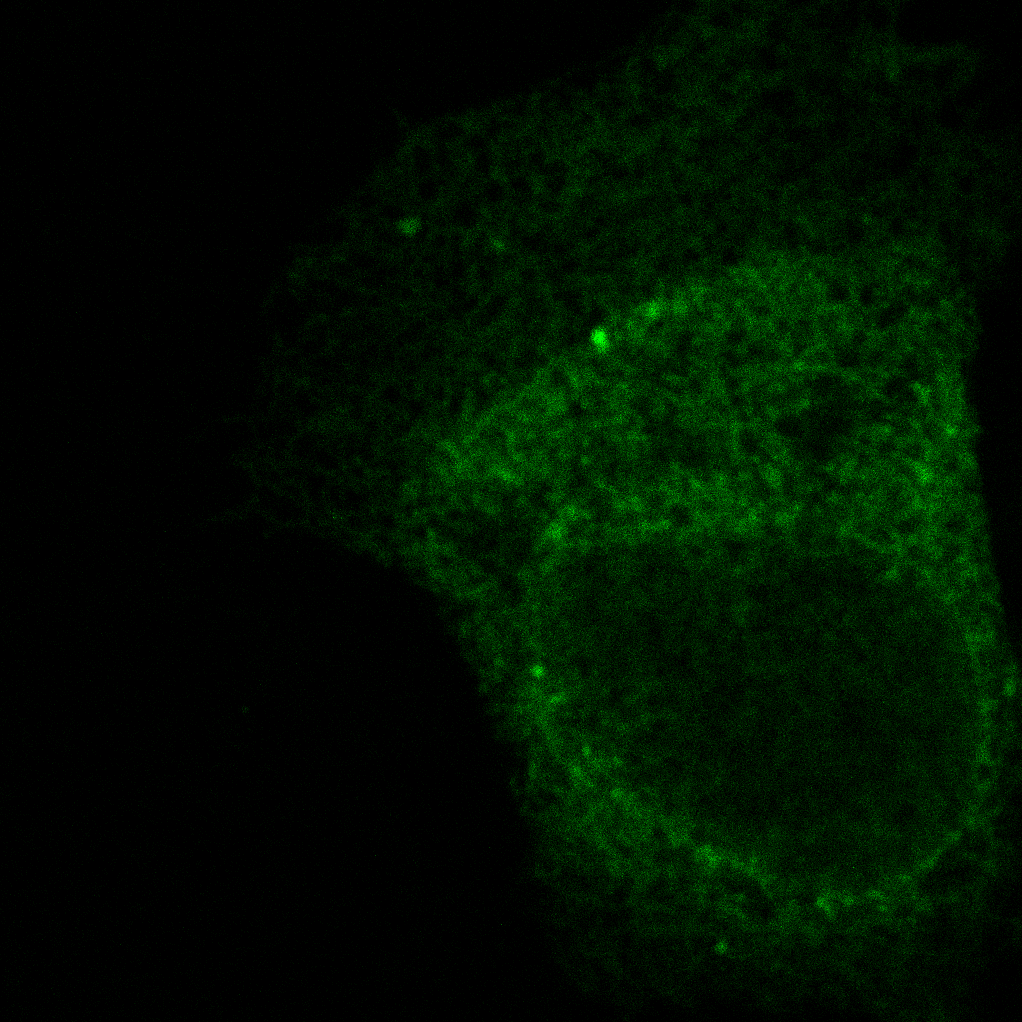

Supplement: Supplementary file 6 — Source data Fig. 4 [file 44318_2024_232_MOESM6_ESM.zip › Figure 4/Figure 4L/S223A.tif]

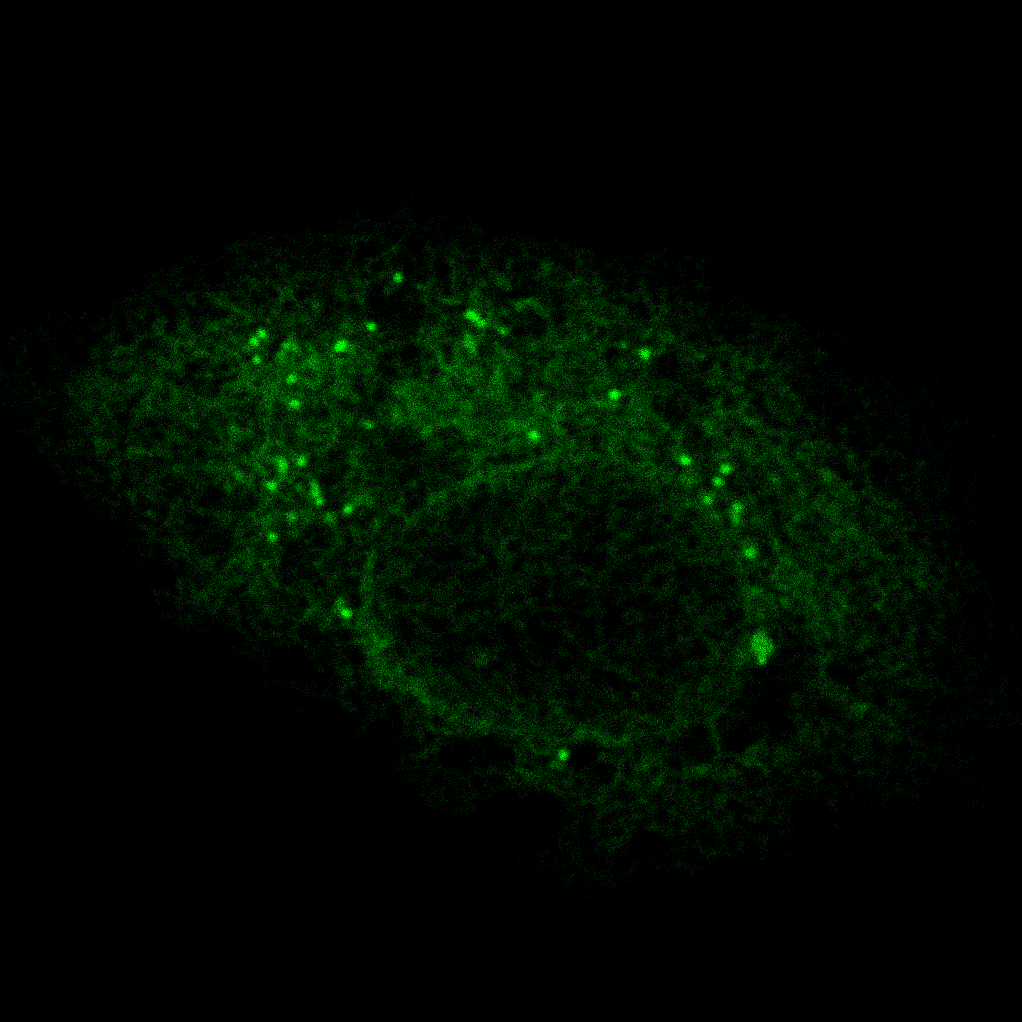

Supplement: Supplementary file 6 — Source data Fig. 4 [file 44318_2024_232_MOESM6_ESM.zip › Figure 4/Figure 4L/S223D.tif]

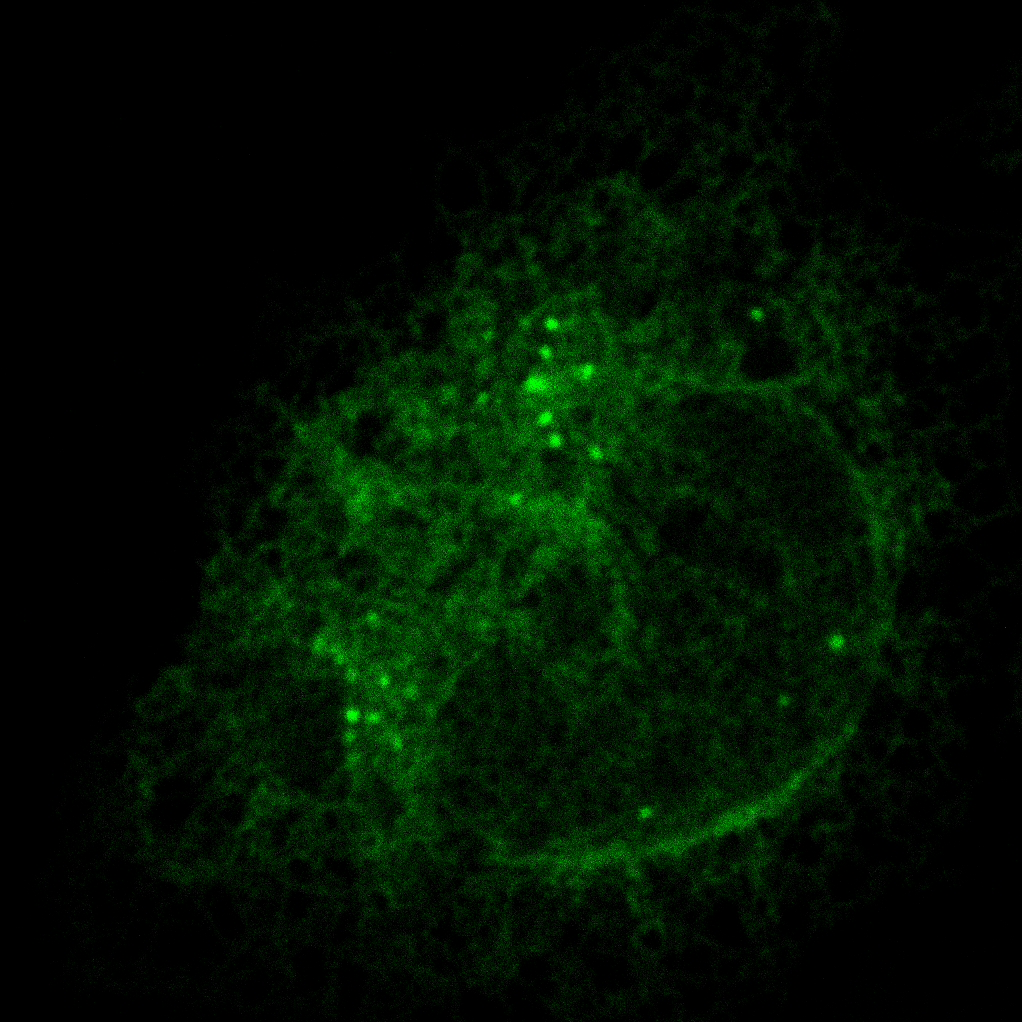

Supplement: Supplementary file 6 — Source data Fig. 4 [file 44318_2024_232_MOESM6_ESM.zip › Figure 4/Figure 4L/WT.tif]

Source data: Figure 4N.

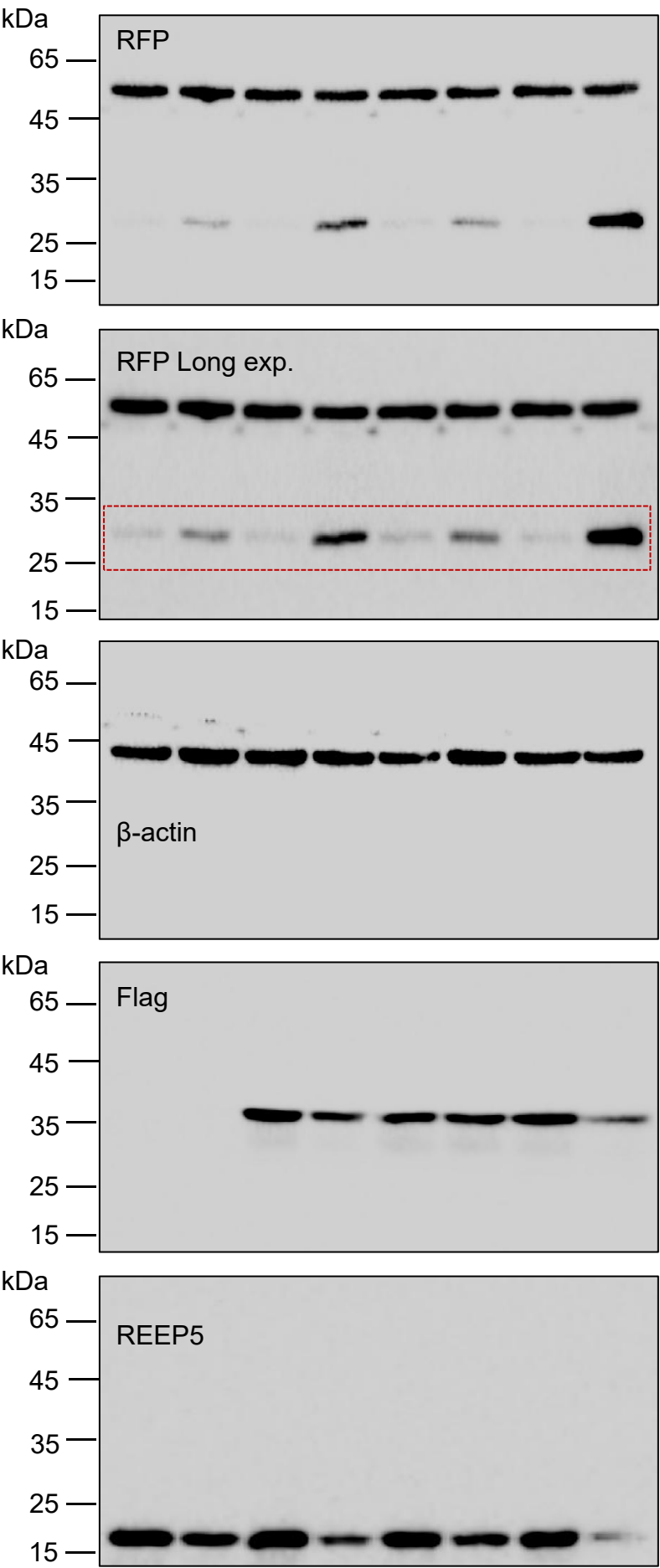

Supplement: Supplementary file 6 — Source data Fig. 4 [file 44318_2024_232_MOESM6_ESM.zip › Figure 4/Figure 4N.pdf]

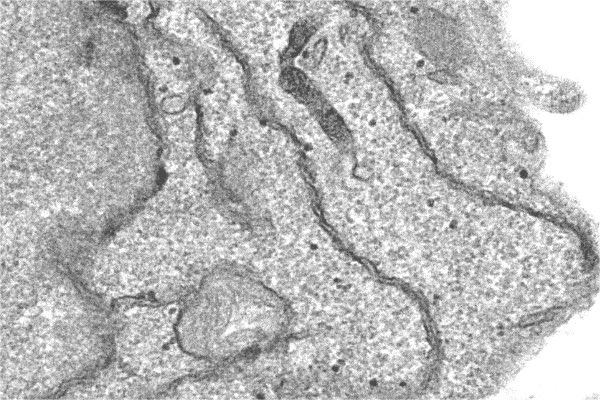

Supplement: Supplementary file 6 — Source data Fig. 4 [file 44318_2024_232_MOESM6_ESM.zip › Figure 4/Figure 4O/Flag-EV.png]

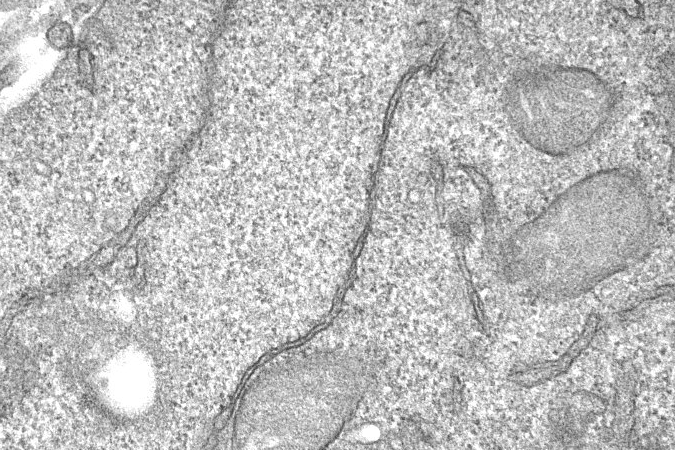

Supplement: Supplementary file 6 — Source data Fig. 4 [file 44318_2024_232_MOESM6_ESM.zip › Figure 4/Figure 4O/Flag-UBAC2 S223A.tif]

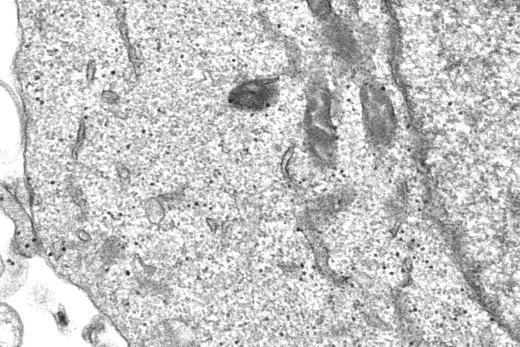

Supplement: Supplementary file 6 — Source data Fig. 4 [file 44318_2024_232_MOESM6_ESM.zip › Figure 4/Figure 4O/Flag-UBAC2 S223D.tif]

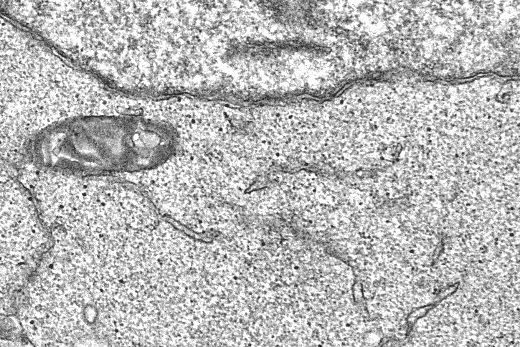

Supplement: Supplementary file 6 — Source data Fig. 4 [file 44318_2024_232_MOESM6_ESM.zip › Figure 4/Figure 4O/Flag-UBAC2.tif]

Source data: Figure 5B.

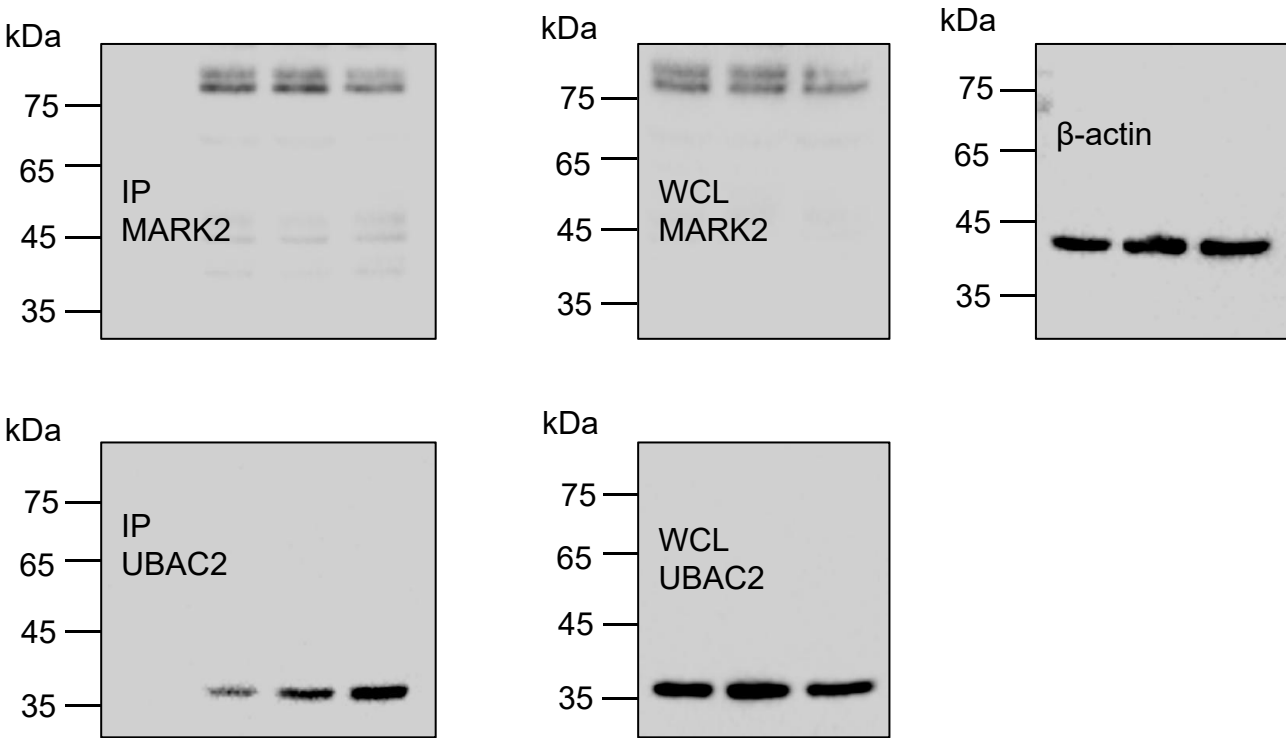

Supplement: Supplementary file 7 — Source data Fig. 5 [file 44318_2024_232_MOESM7_ESM.zip › Figure 5/Figure 5B.pdf]

Source data: Figure 5C.

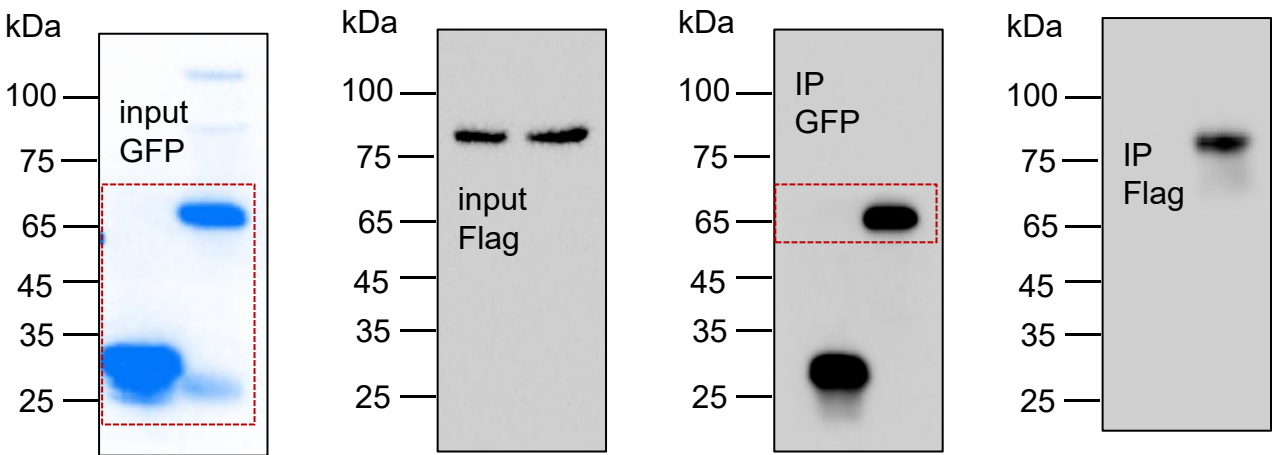

Supplement: Supplementary file 7 — Source data Fig. 5 [file 44318_2024_232_MOESM7_ESM.zip › Figure 5/Figure 5C.pdf]

Source data: Figure 5D.

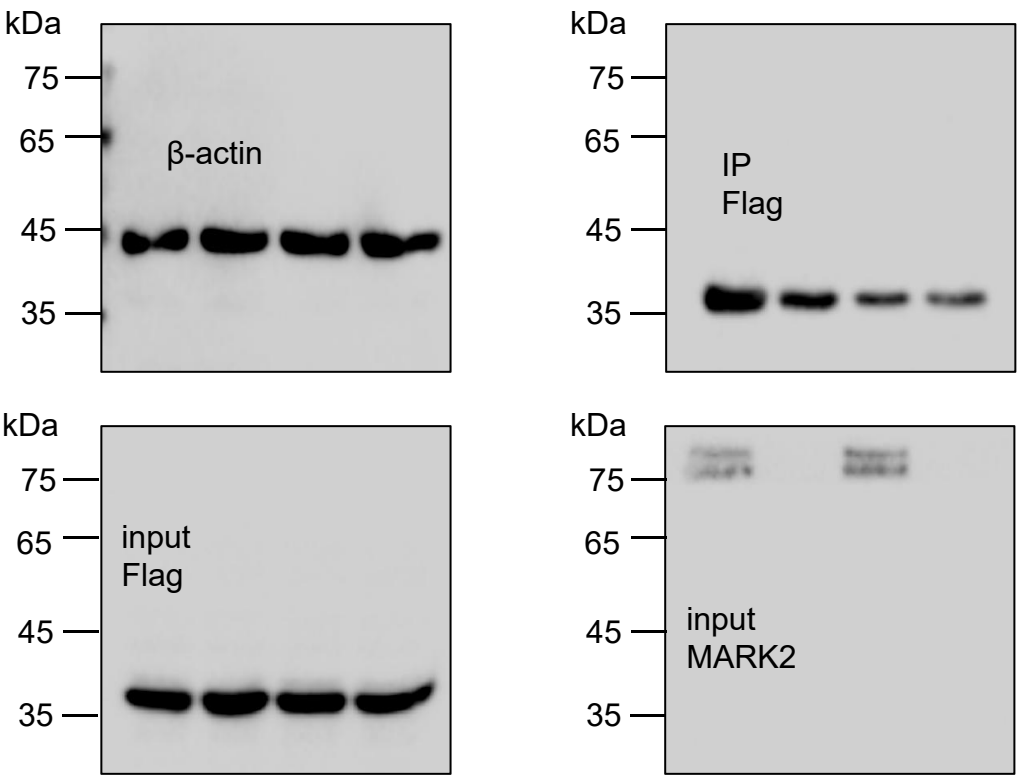

Supplement: Supplementary file 7 — Source data Fig. 5 [file 44318_2024_232_MOESM7_ESM.zip › Figure 5/Figure 5D.pdf]

Source data: Figure 5E.

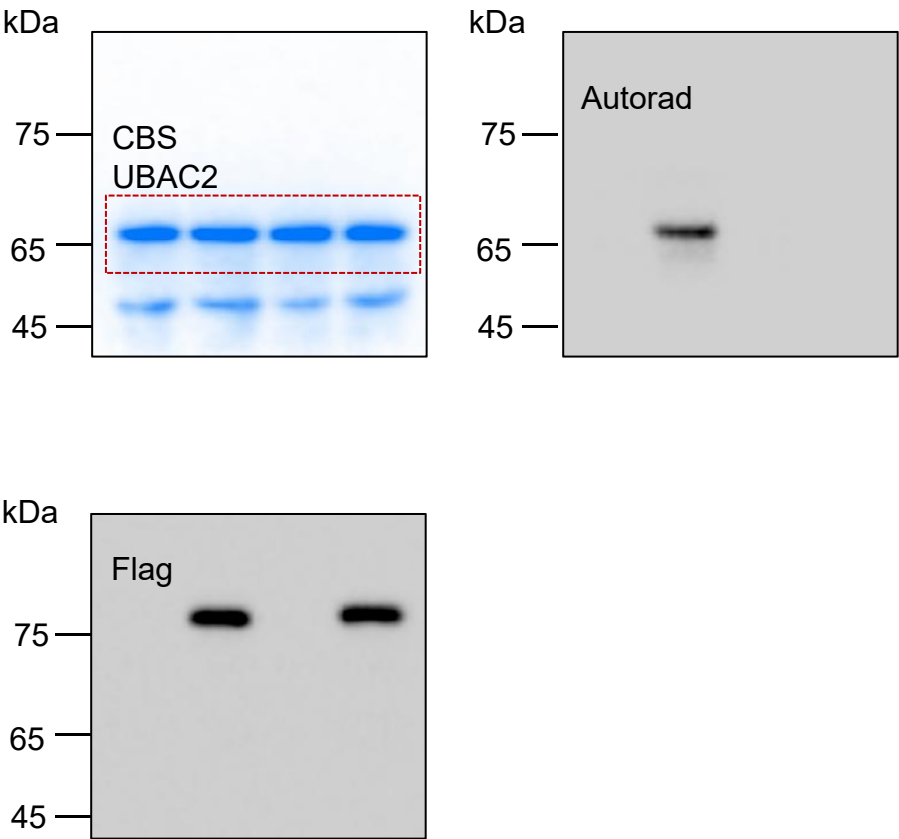

Supplement: Supplementary file 7 — Source data Fig. 5 [file 44318_2024_232_MOESM7_ESM.zip › Figure 5/Figure 5E.pdf]

Source data: Figure 5F.

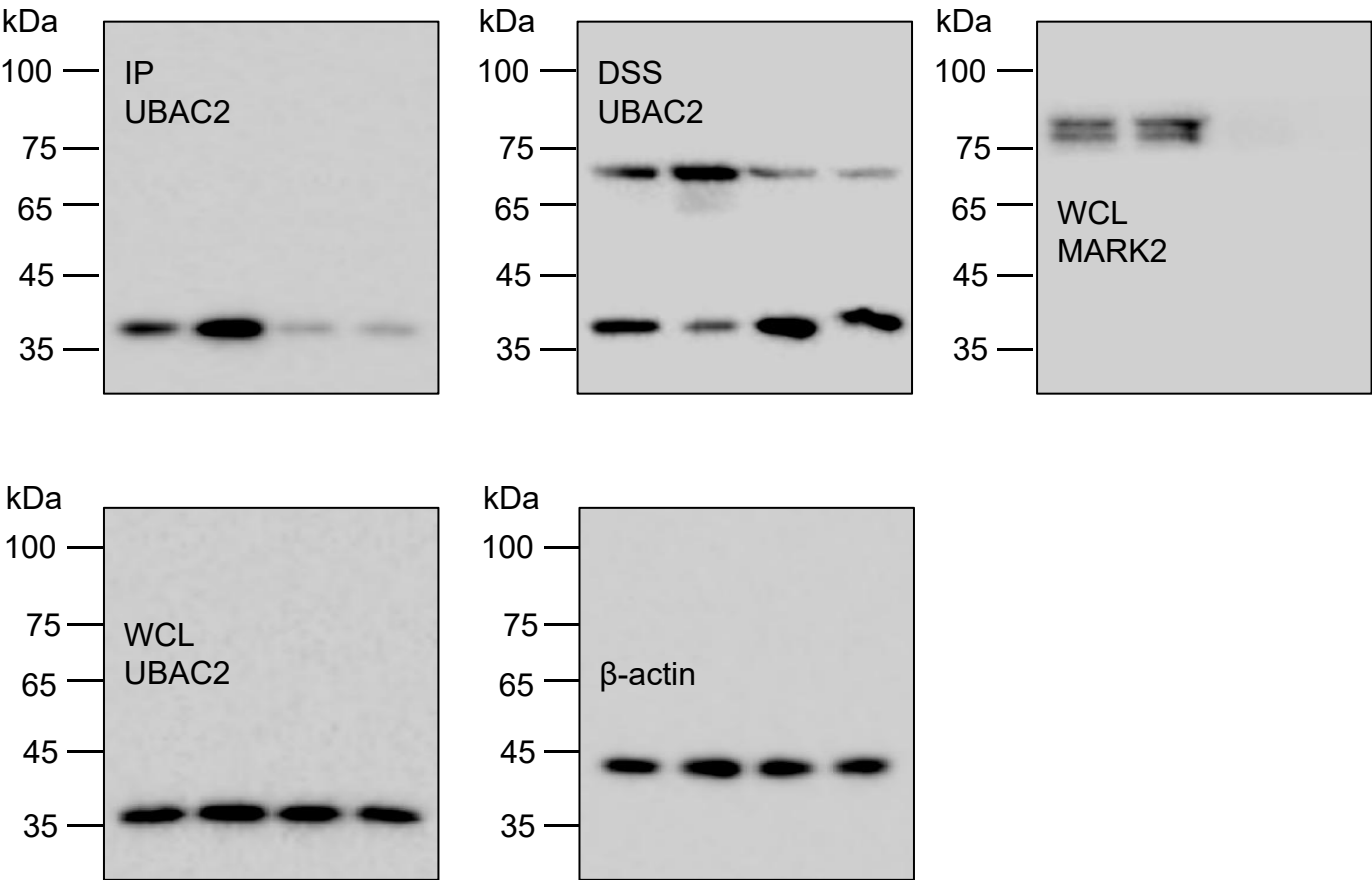

Supplement: Supplementary file 7 — Source data Fig. 5 [file 44318_2024_232_MOESM7_ESM.zip › Figure 5/Figure 5F.pdf]

Source data: Figure 5G.

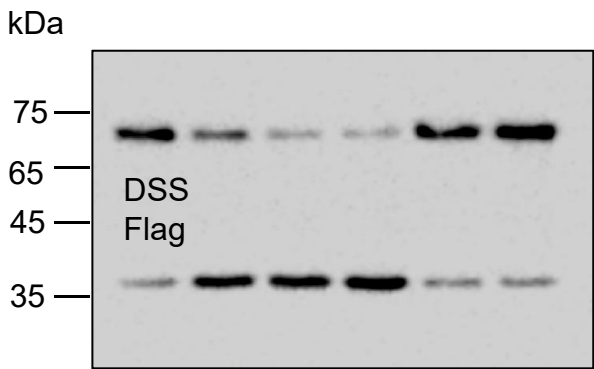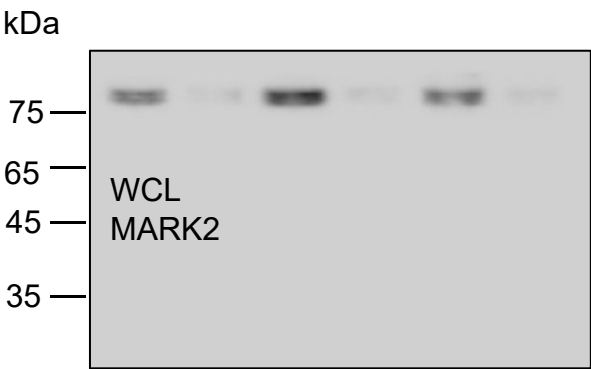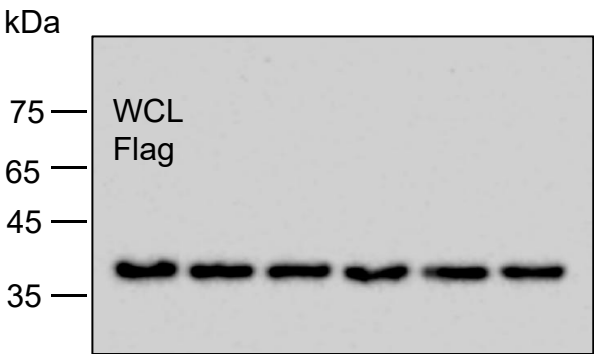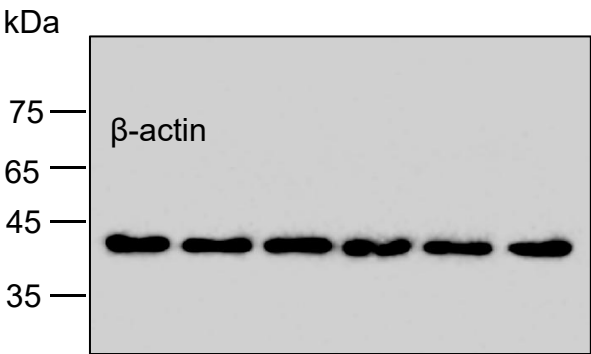

Supplement: Supplementary file 7 — Source data Fig. 5 [file 44318_2024_232_MOESM7_ESM.zip › Figure 5/Figure 5G.pdf]

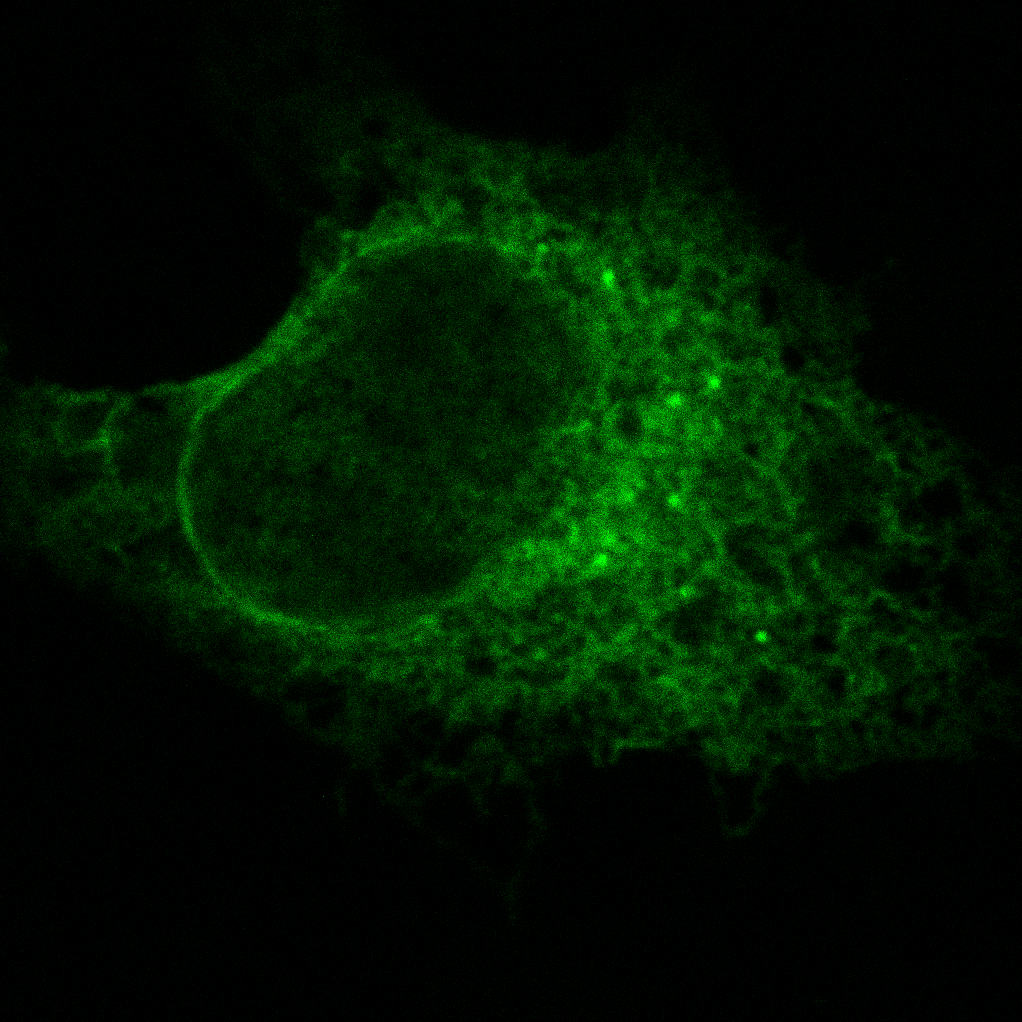

Supplement: Supplementary file 7 — Source data Fig. 5 [file 44318_2024_232_MOESM7_ESM.zip › Figure 5/Figure 5H/MARK2 siRNA/S223A.tif]

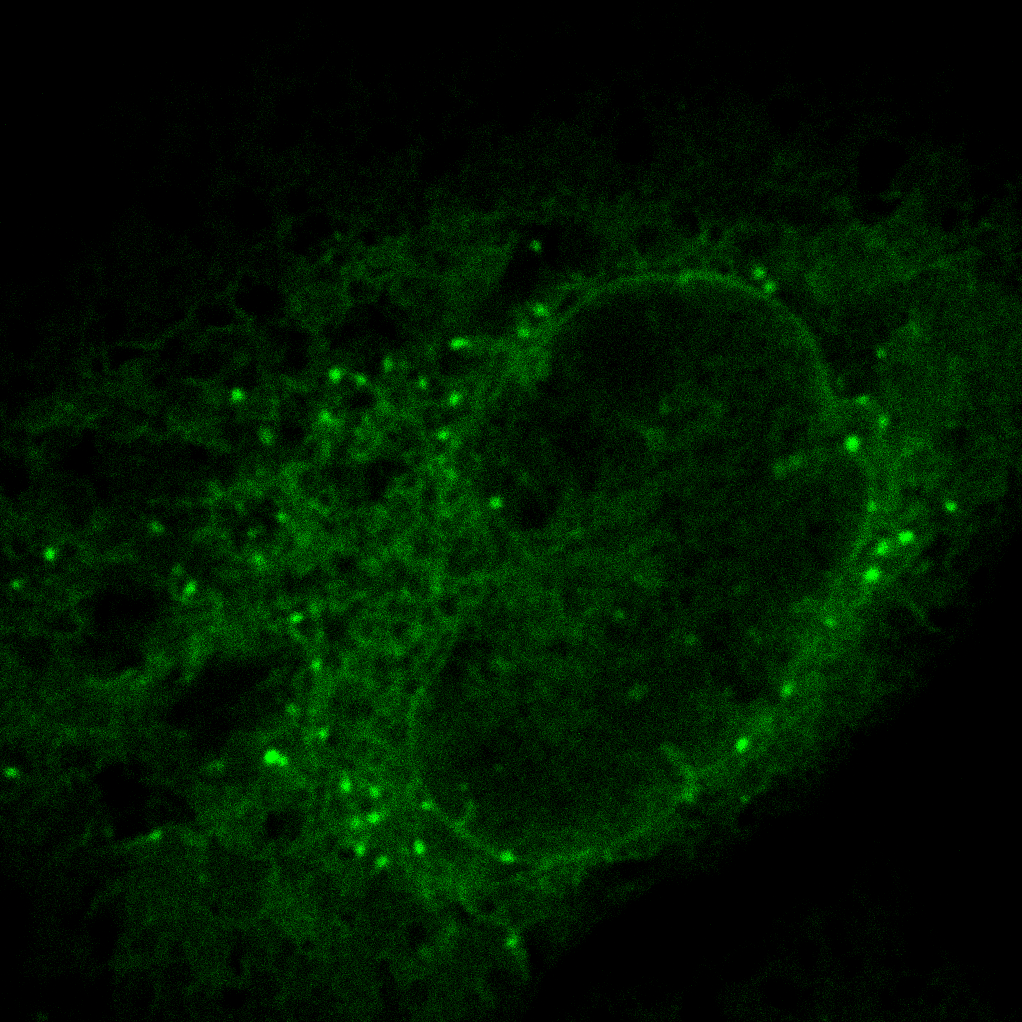

Supplement: Supplementary file 7 — Source data Fig. 5 [file 44318_2024_232_MOESM7_ESM.zip › Figure 5/Figure 5H/MARK2 siRNA/S223D.tif]

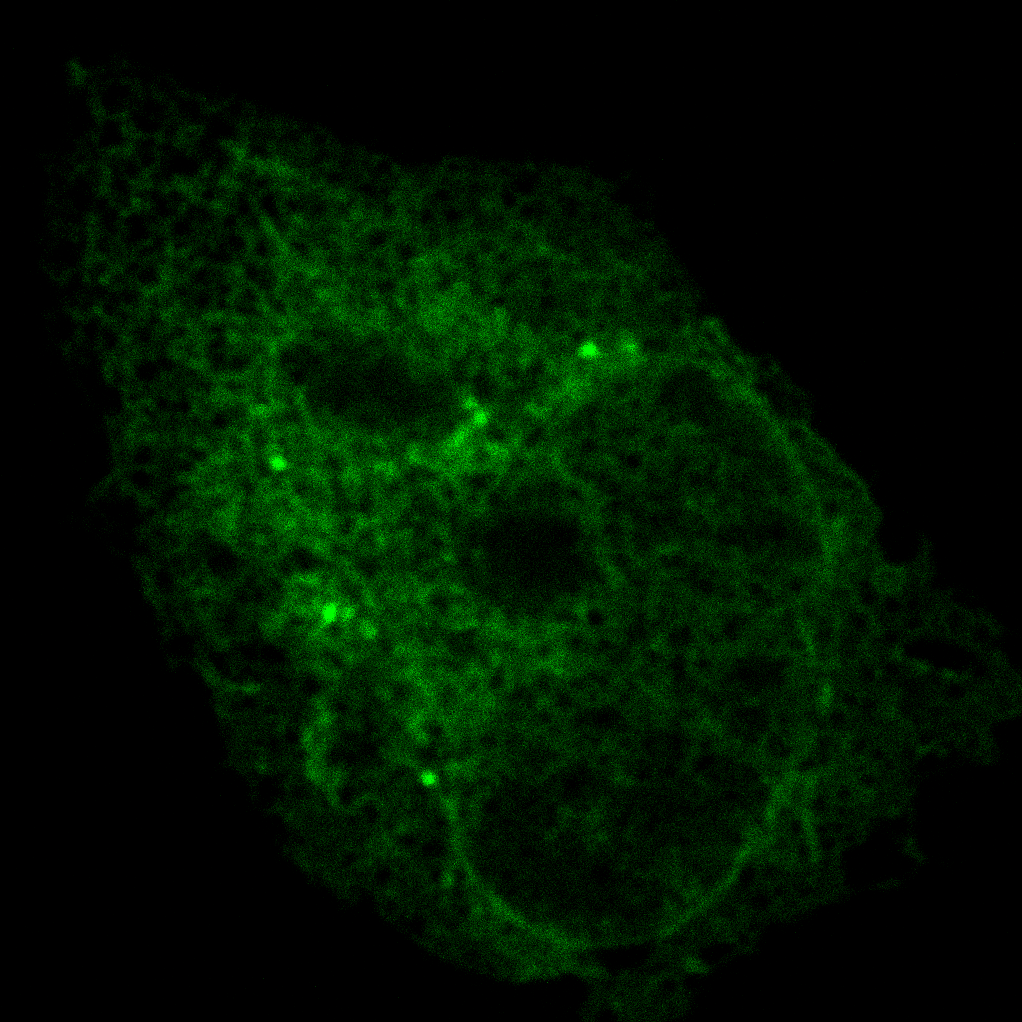

Supplement: Supplementary file 7 — Source data Fig. 5 [file 44318_2024_232_MOESM7_ESM.zip › Figure 5/Figure 5H/MARK2 siRNA/WT.tif]

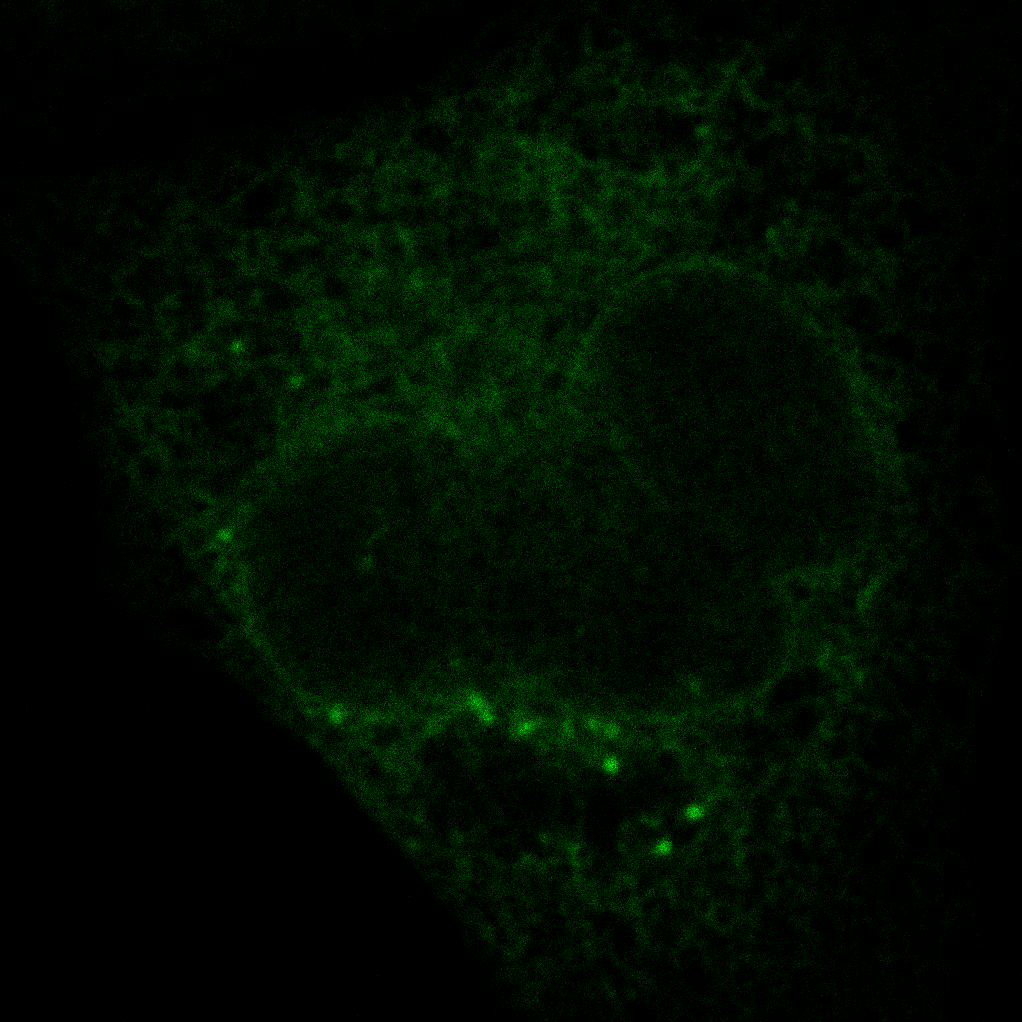

Supplement: Supplementary file 7 — Source data Fig. 5 [file 44318_2024_232_MOESM7_ESM.zip › Figure 5/Figure 5H/Scr siRNA/S223A.tif]

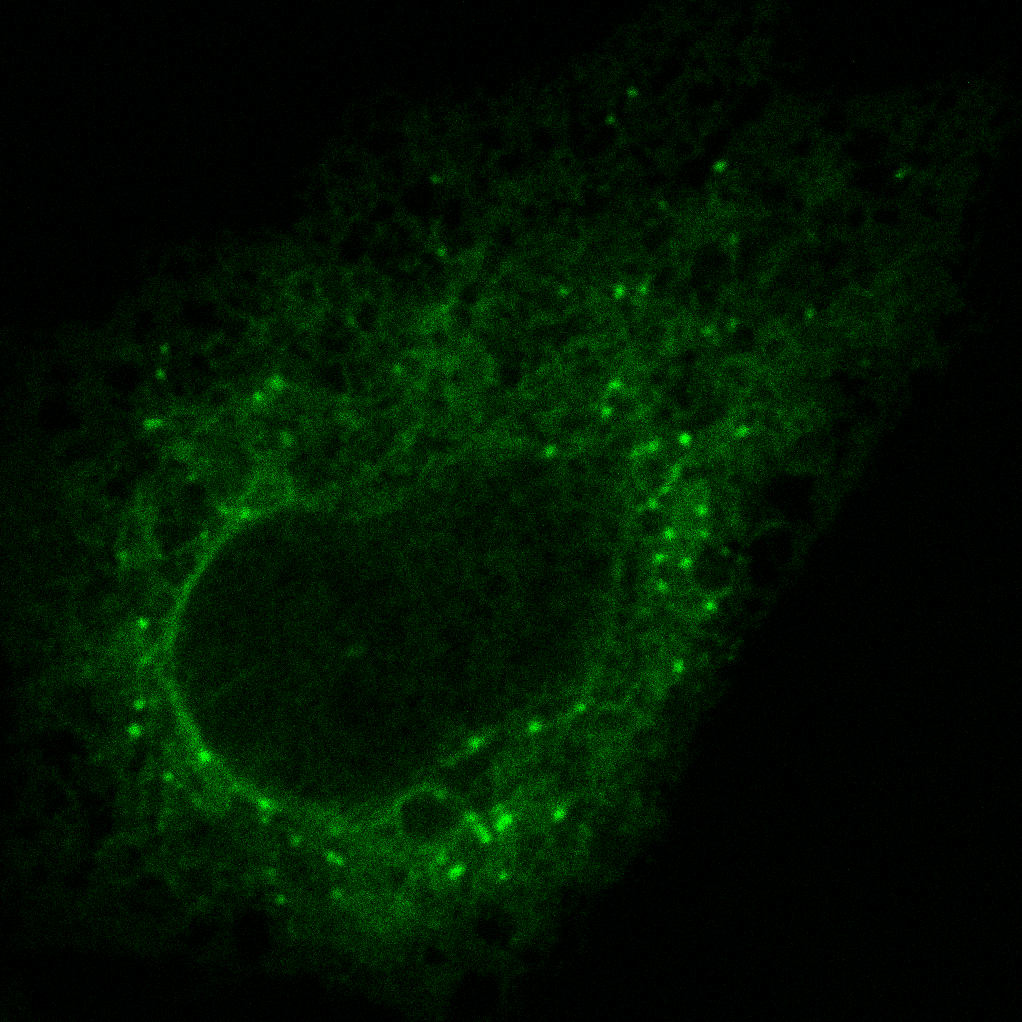

Supplement: Supplementary file 7 — Source data Fig. 5 [file 44318_2024_232_MOESM7_ESM.zip › Figure 5/Figure 5H/Scr siRNA/S223D.tif]
