# Supplementary figures and images for: ER-phagy restrains inflammatory responses through its receptor UBAC2 (part 2 of 2)
Source: EMBO J. 2024 Sep 16;43(21):13. doi: 10.1038/s44318-024-00232-z (PMC11535055; doi:10.1038/s44318-024-00232-z)

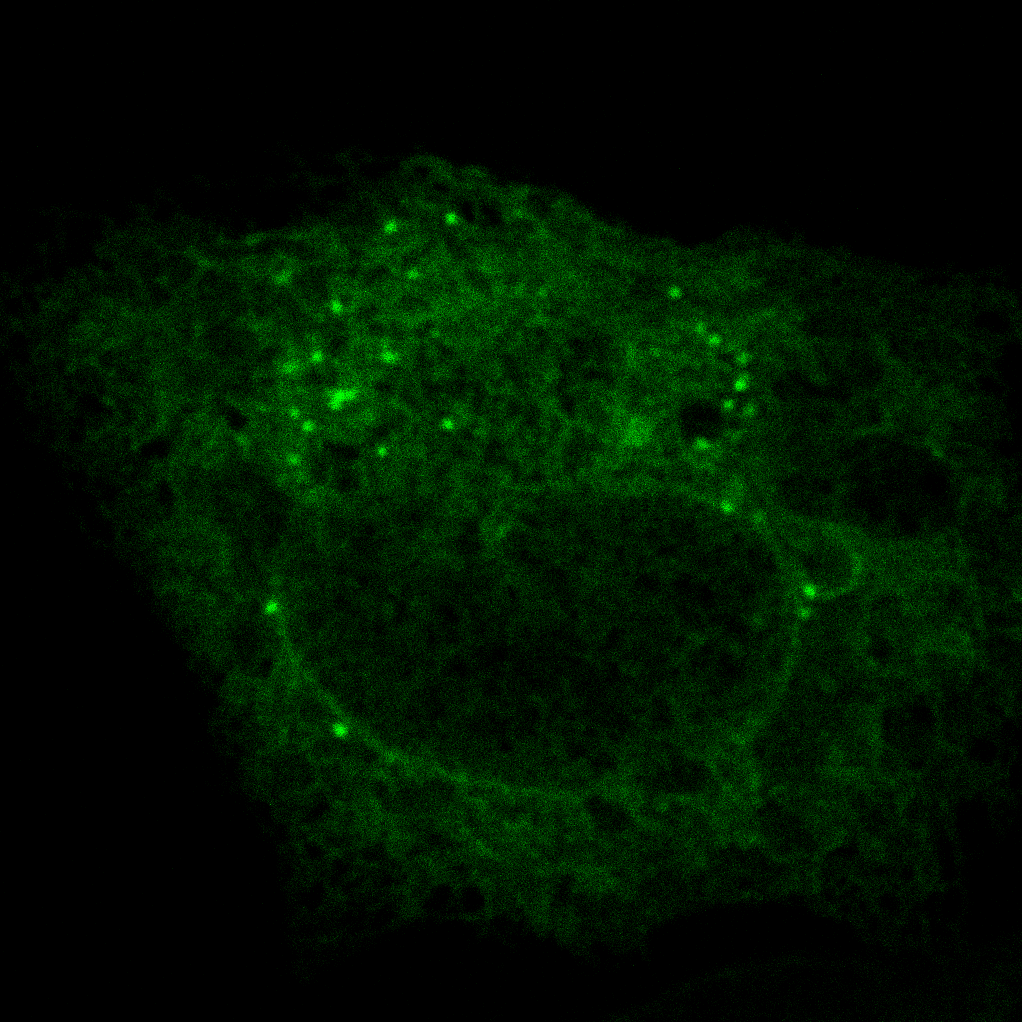

Supplement: Supplementary file 7 — Source data Fig. 5 [file 44318_2024_232_MOESM7_ESM.zip › Figure 5/Figure 5H/Scr siRNA/WT.tif]

Source data: Figure 5J.

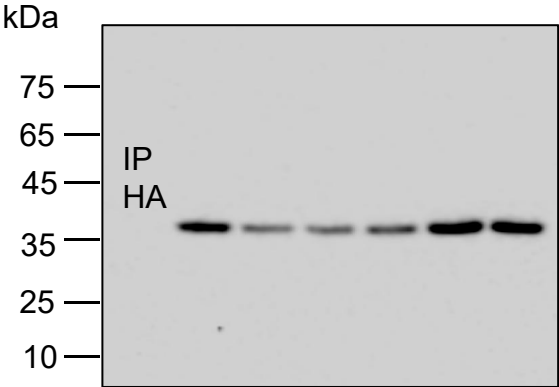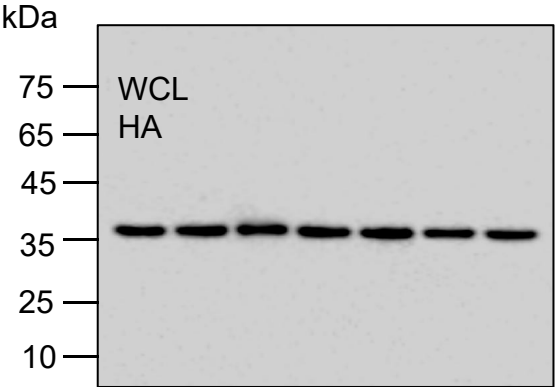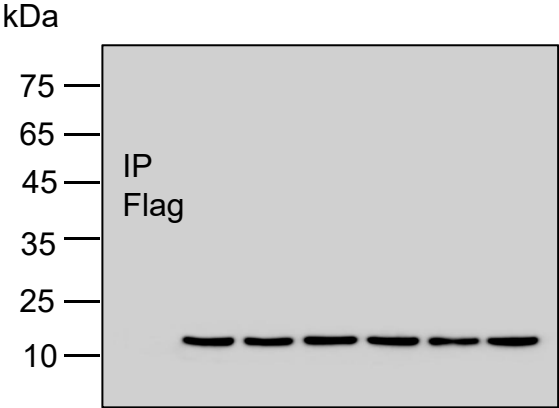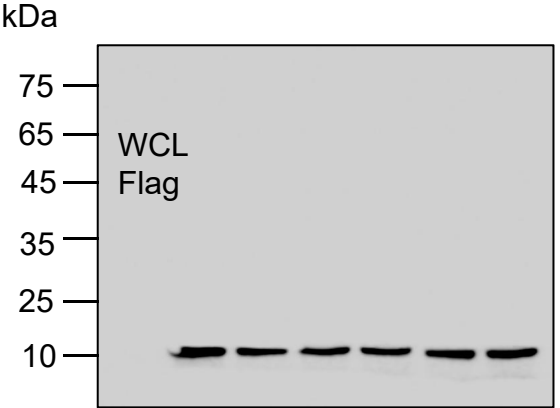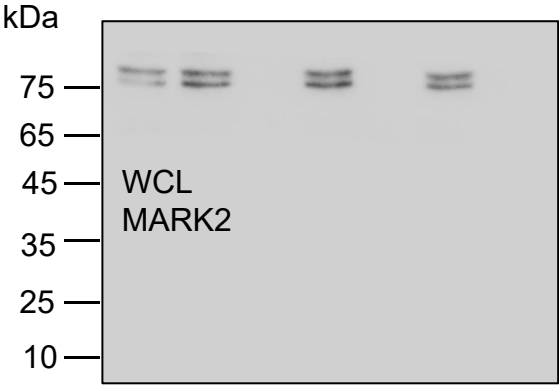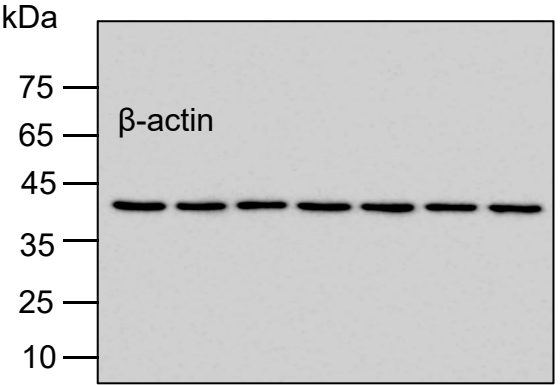

Supplement: Supplementary file 7 — Source data Fig. 5 [file 44318_2024_232_MOESM7_ESM.zip › Figure 5/Figure 5J.pdf]

Source data: Figure 5K.

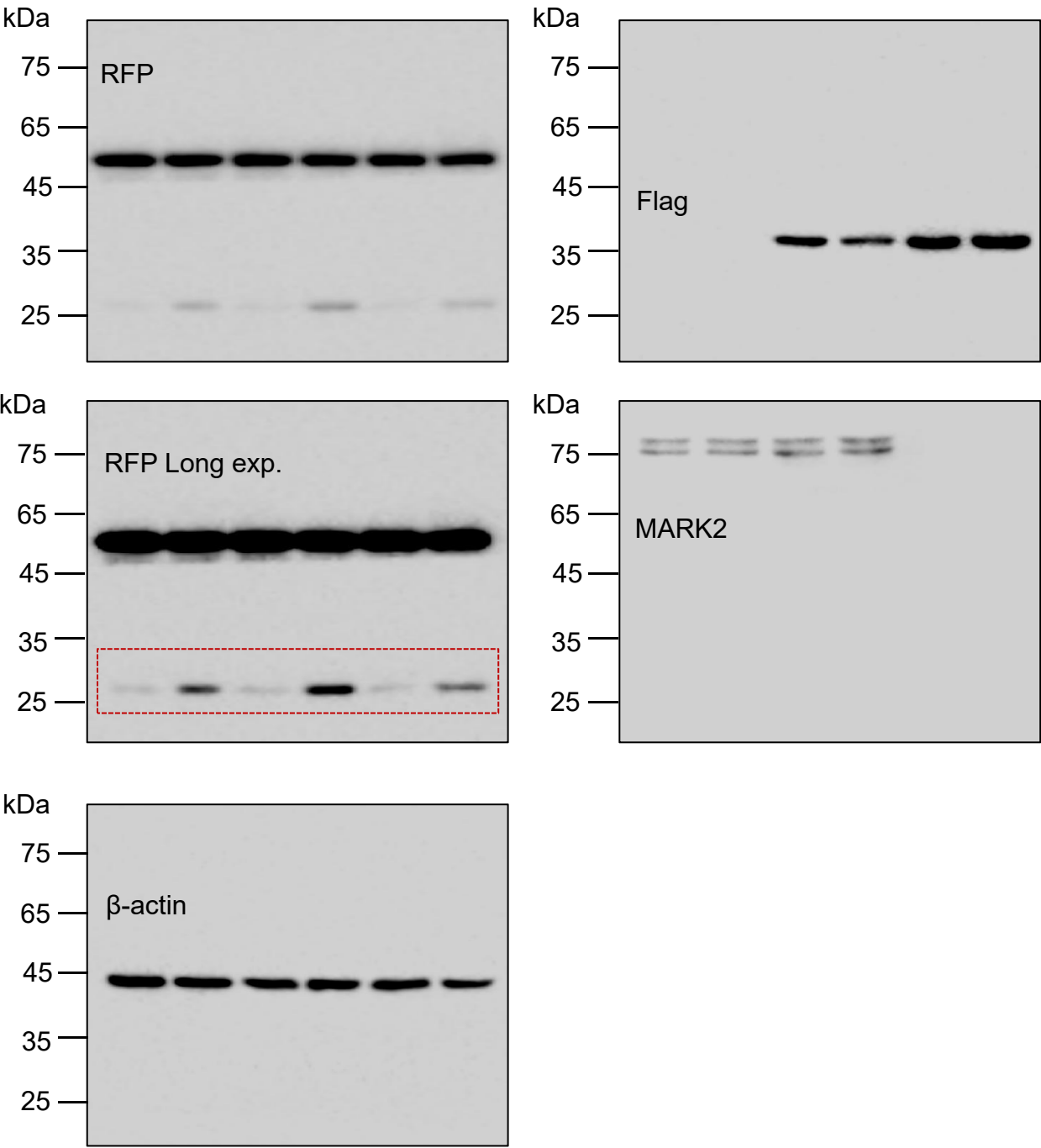

Supplement: Supplementary file 7 — Source data Fig. 5 [file 44318_2024_232_MOESM7_ESM.zip › Figure 5/Figure 5K.pdf]

Source data: Figure 5L.

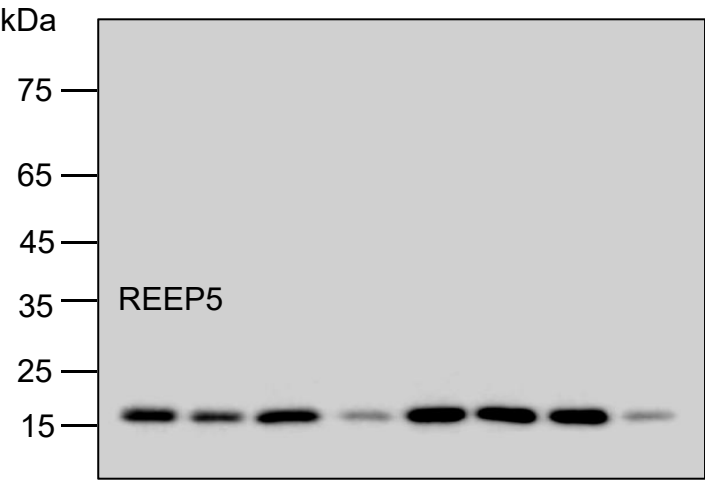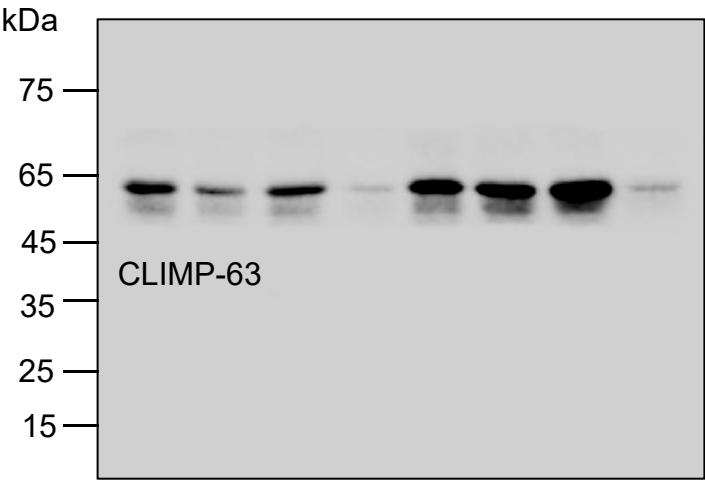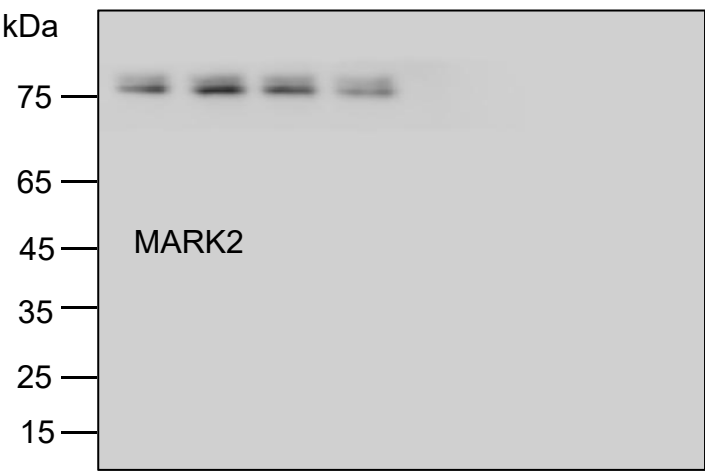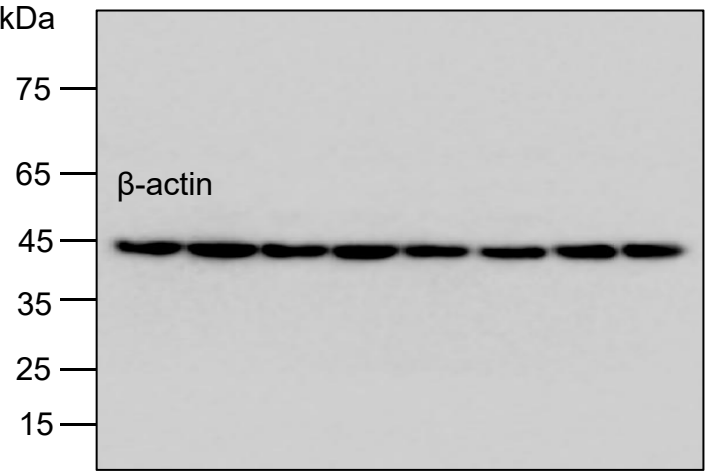

Supplement: Supplementary file 7 — Source data Fig. 5 [file 44318_2024_232_MOESM7_ESM.zip › Figure 5/Figure 5L.pdf]

Source data: Figure 6B.

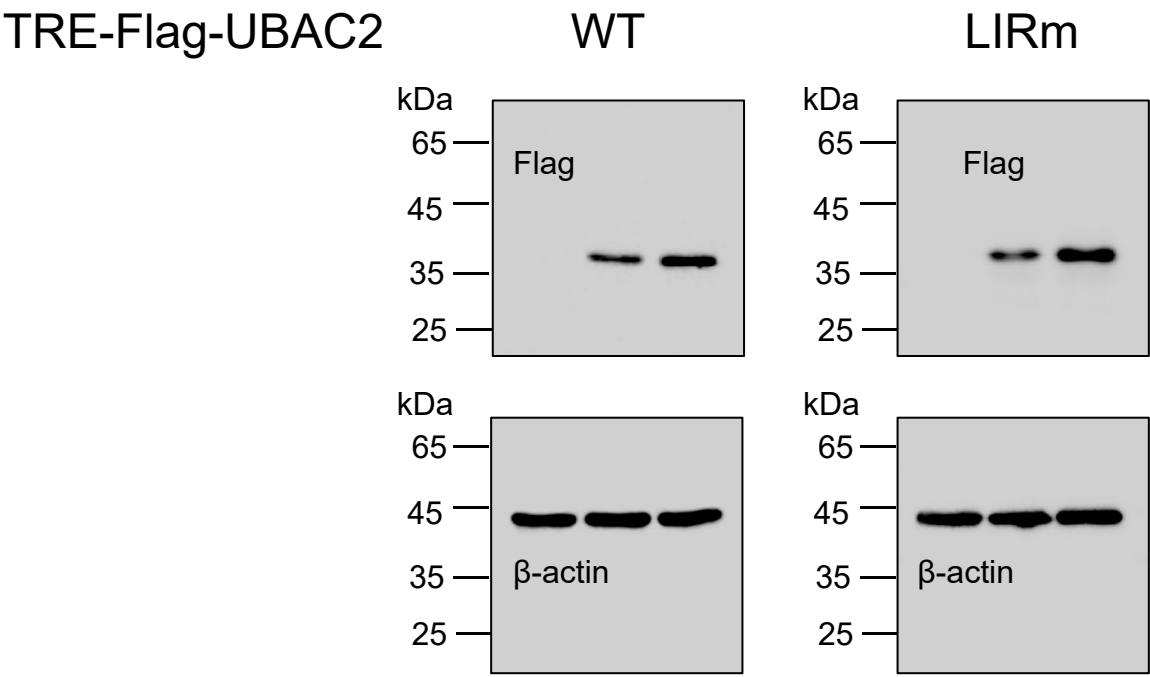

Supplement: Supplementary file 8 — Source data Fig. 6 [file 44318_2024_232_MOESM8_ESM.zip › Figure 6/Figure 6B.pdf]

Source data: Figure 7A.

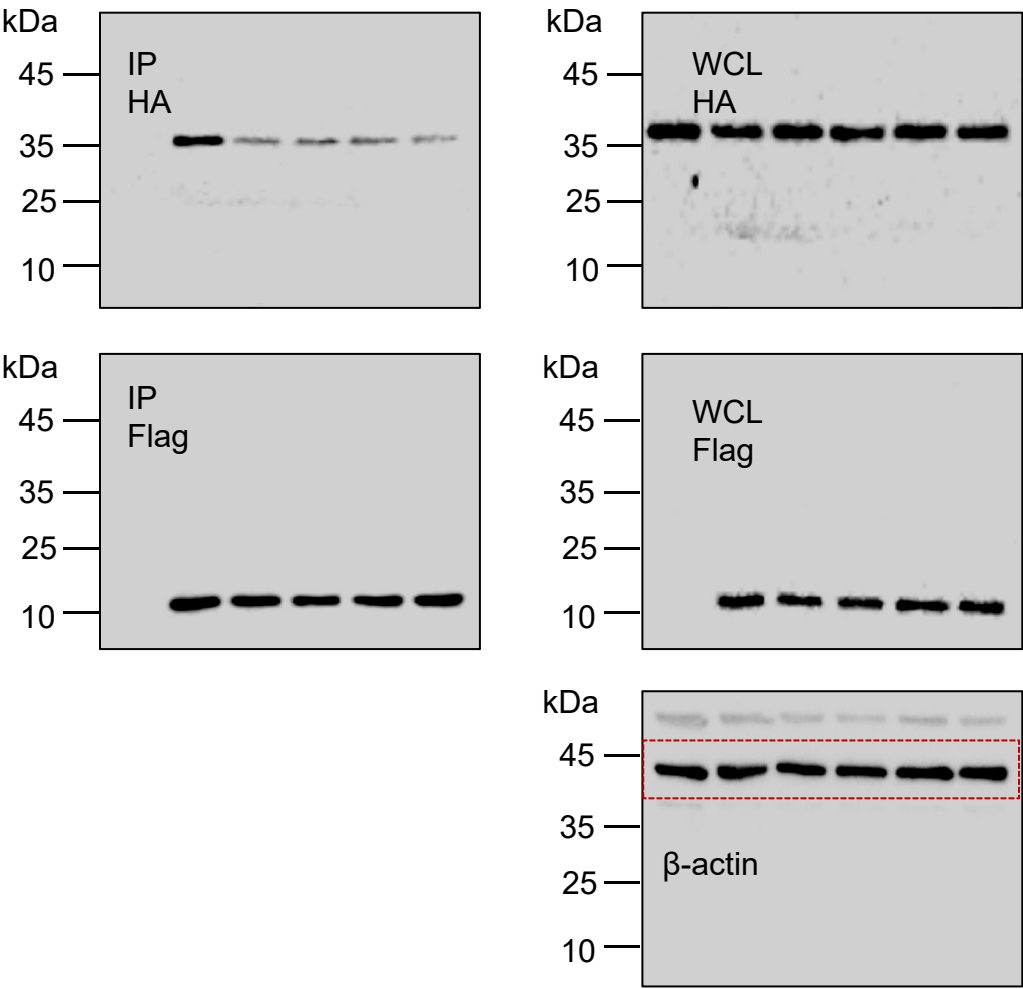

Supplement: Supplementary file 9 — Source data Fig. 7 [file 44318_2024_232_MOESM9_ESM.zip › Figure 7/Figure 7A.pdf]

Source data: Figure 7B.

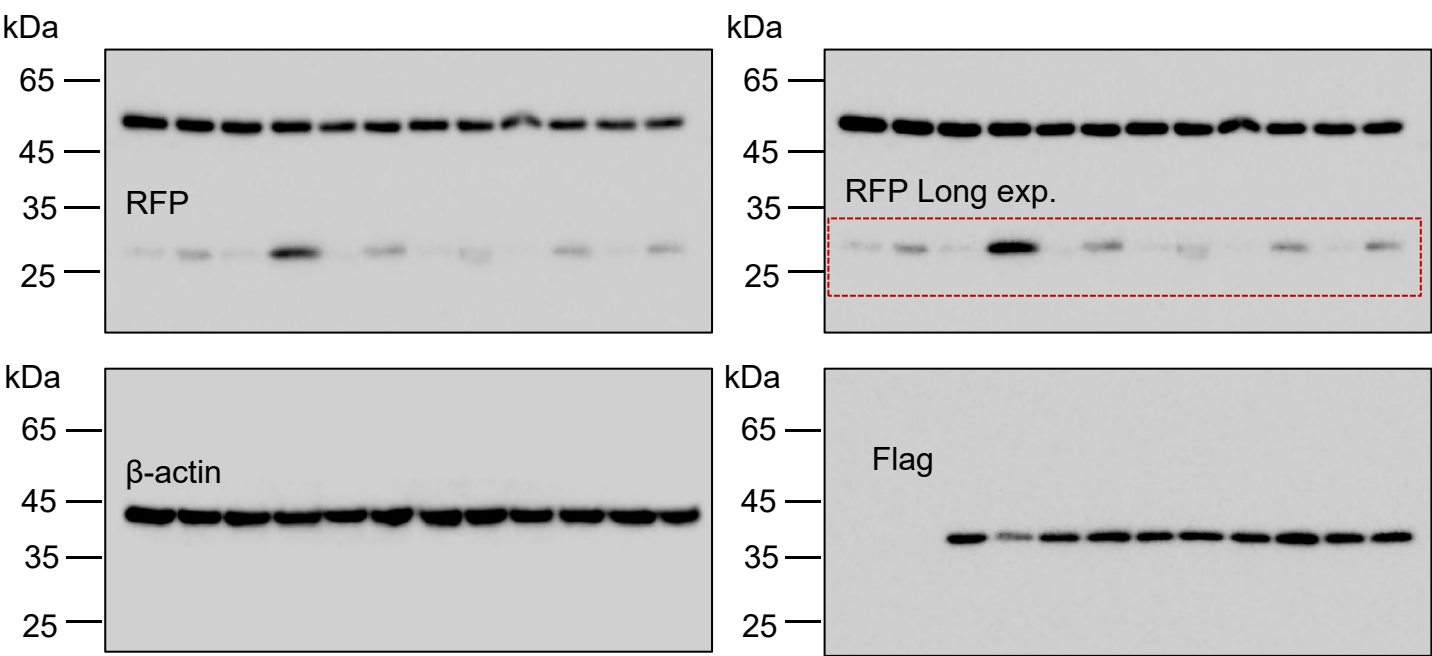

Supplement: Supplementary file 9 — Source data Fig. 7 [file 44318_2024_232_MOESM9_ESM.zip › Figure 7/Figure 7B.pdf]

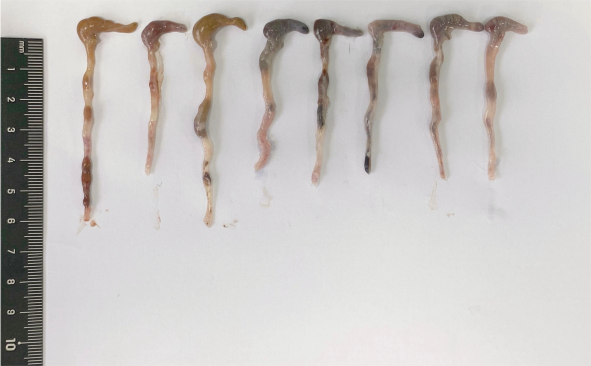

Supplement: Supplementary file 9 — Source data Fig. 7 [file 44318_2024_232_MOESM9_ESM.zip › Figure 7/Figure 7F/3% DSS.tif]

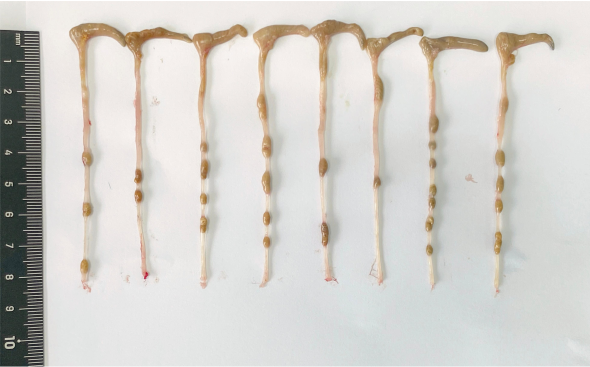

Supplement: Supplementary file 9 — Source data Fig. 7 [file 44318_2024_232_MOESM9_ESM.zip › Figure 7/Figure 7F/Water.tif]

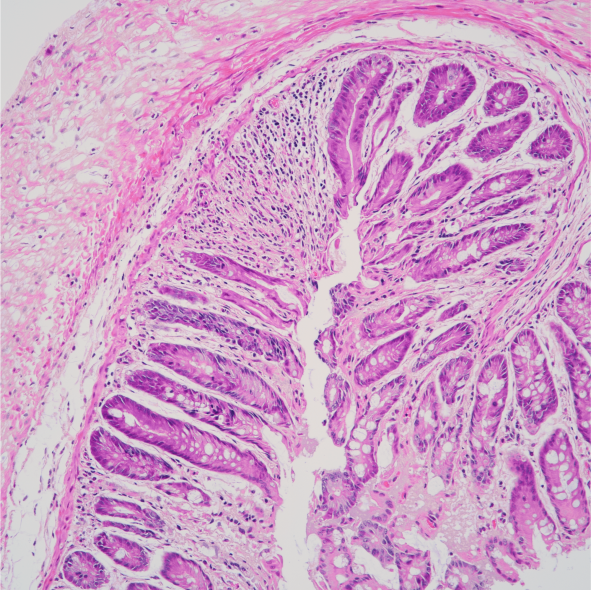

Supplement: Supplementary file 9 — Source data Fig. 7 [file 44318_2024_232_MOESM9_ESM.zip › Figure 7/Figure 7H/3% DSS/Scr siRNA/-.tif]

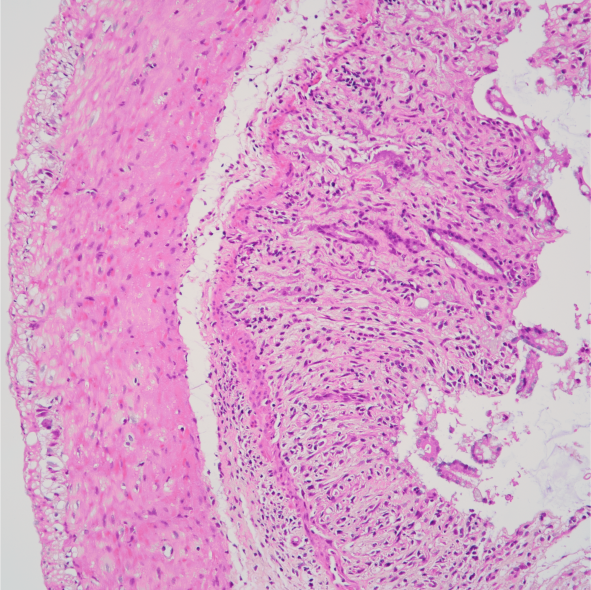

Supplement: Supplementary file 9 — Source data Fig. 7 [file 44318_2024_232_MOESM9_ESM.zip › Figure 7/Figure 7H/3% DSS/Ubac2 shRNA/-.tif]

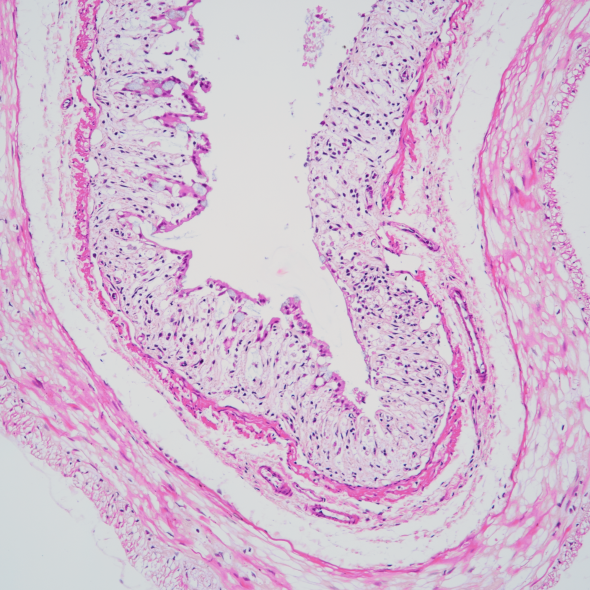

Supplement: Supplementary file 9 — Source data Fig. 7 [file 44318_2024_232_MOESM9_ESM.zip › Figure 7/Figure 7H/3% DSS/Ubac2 shRNA/F279S.tif]

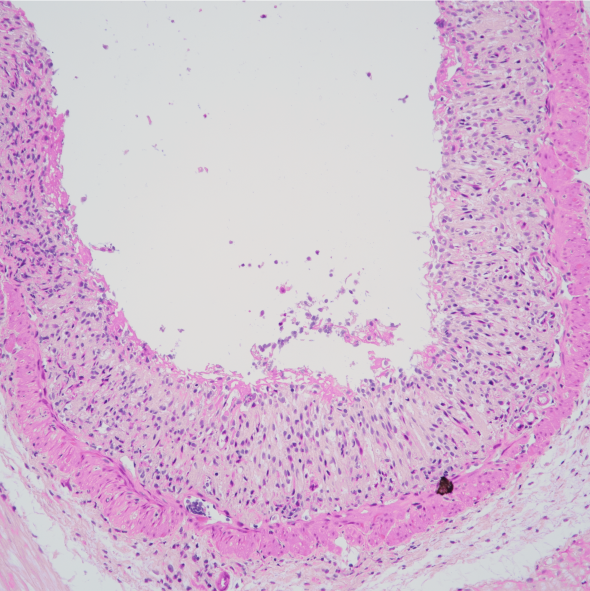

Supplement: Supplementary file 9 — Source data Fig. 7 [file 44318_2024_232_MOESM9_ESM.zip › Figure 7/Figure 7H/3% DSS/Ubac2 shRNA/G293S.tif]

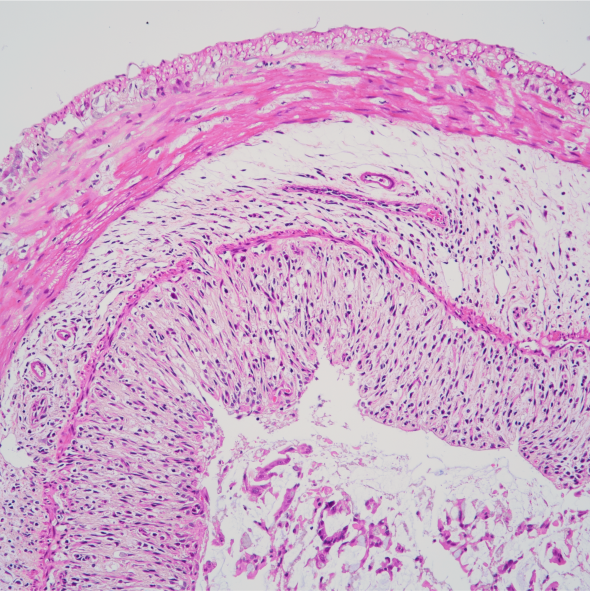

Supplement: Supplementary file 9 — Source data Fig. 7 [file 44318_2024_232_MOESM9_ESM.zip › Figure 7/Figure 7H/3% DSS/Ubac2 shRNA/LIRm.tif]

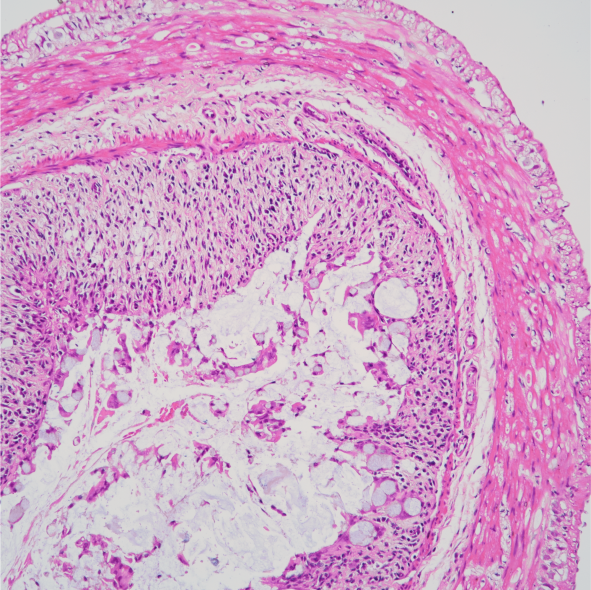

Supplement: Supplementary file 9 — Source data Fig. 7 [file 44318_2024_232_MOESM9_ESM.zip › Figure 7/Figure 7H/3% DSS/Ubac2 shRNA/R277C.tif]

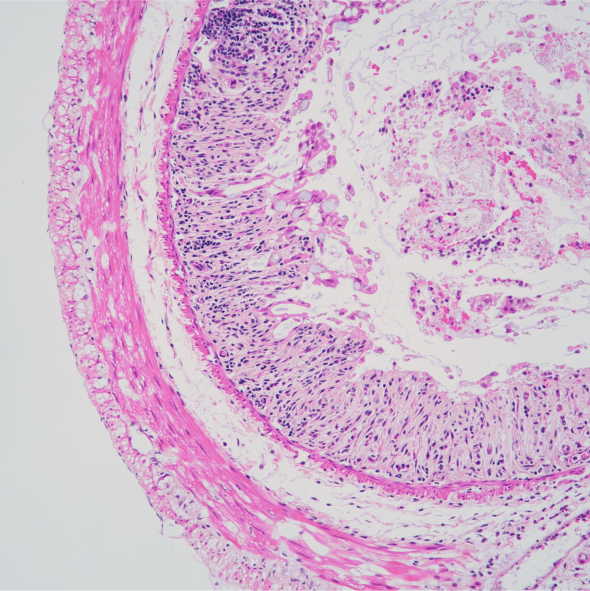

Supplement: Supplementary file 9 — Source data Fig. 7 [file 44318_2024_232_MOESM9_ESM.zip › Figure 7/Figure 7H/3% DSS/Ubac2 shRNA/S223A.tif]

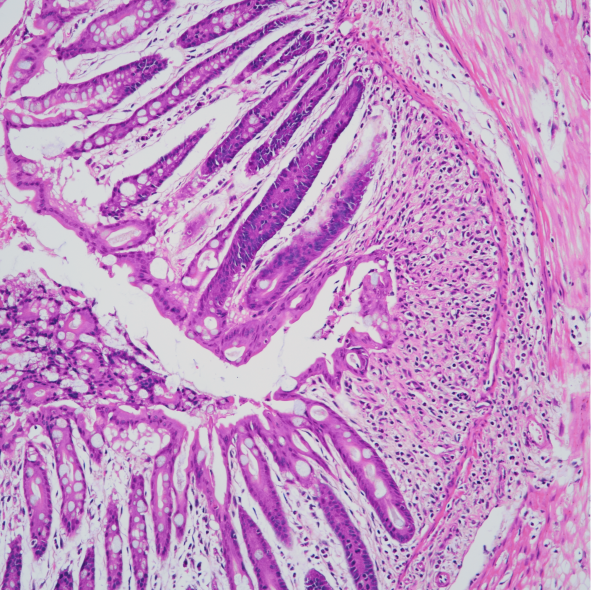

Supplement: Supplementary file 9 — Source data Fig. 7 [file 44318_2024_232_MOESM9_ESM.zip › Figure 7/Figure 7H/3% DSS/Ubac2 shRNA/WT.tif]

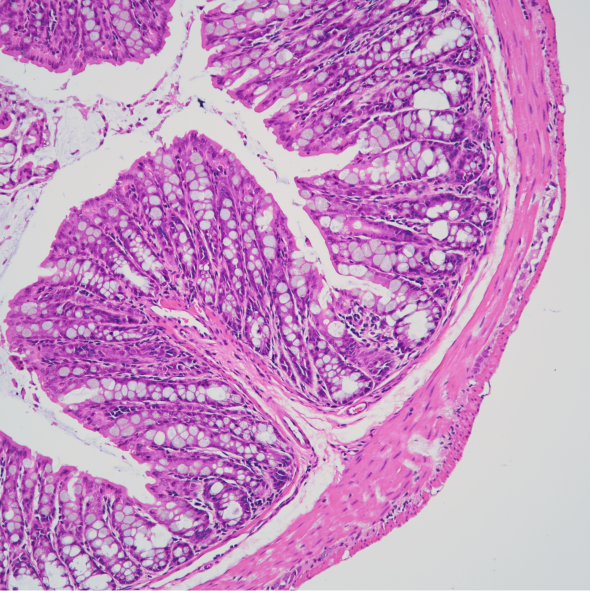

Supplement: Supplementary file 9 — Source data Fig. 7 [file 44318_2024_232_MOESM9_ESM.zip › Figure 7/Figure 7H/Water/Scr shRNA/-.tif]

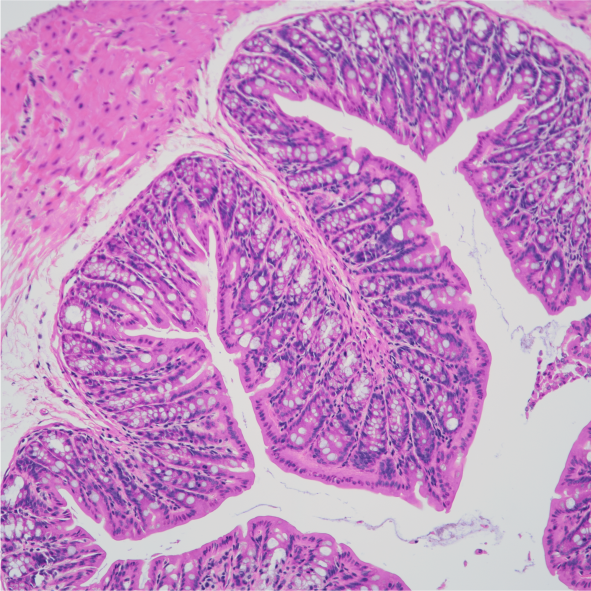

Supplement: Supplementary file 9 — Source data Fig. 7 [file 44318_2024_232_MOESM9_ESM.zip › Figure 7/Figure 7H/Water/Ubac2 shRNA/-.tif]

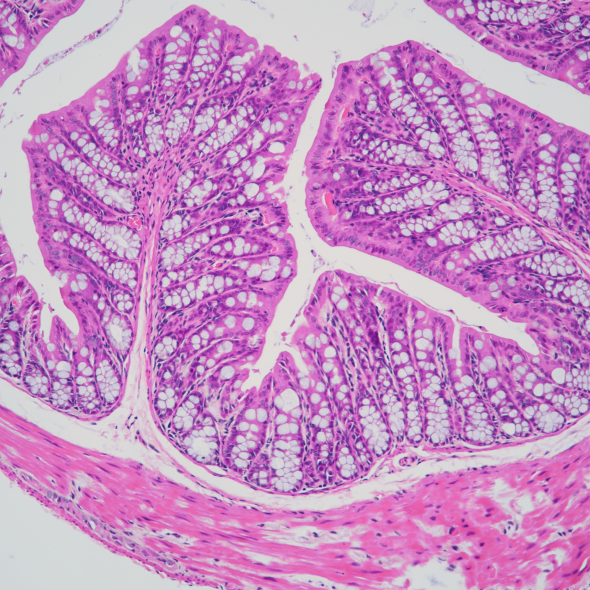

Supplement: Supplementary file 9 — Source data Fig. 7 [file 44318_2024_232_MOESM9_ESM.zip › Figure 7/Figure 7H/Water/Ubac2 shRNA/F279S.tif]

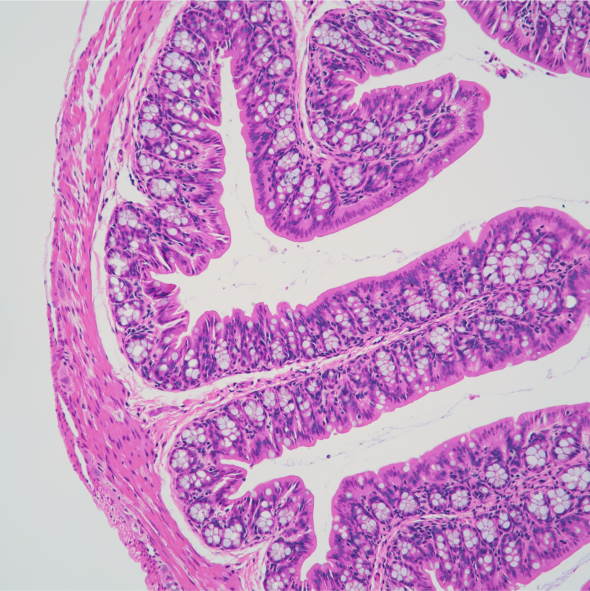

Supplement: Supplementary file 9 — Source data Fig. 7 [file 44318_2024_232_MOESM9_ESM.zip › Figure 7/Figure 7H/Water/Ubac2 shRNA/G293S.tif]

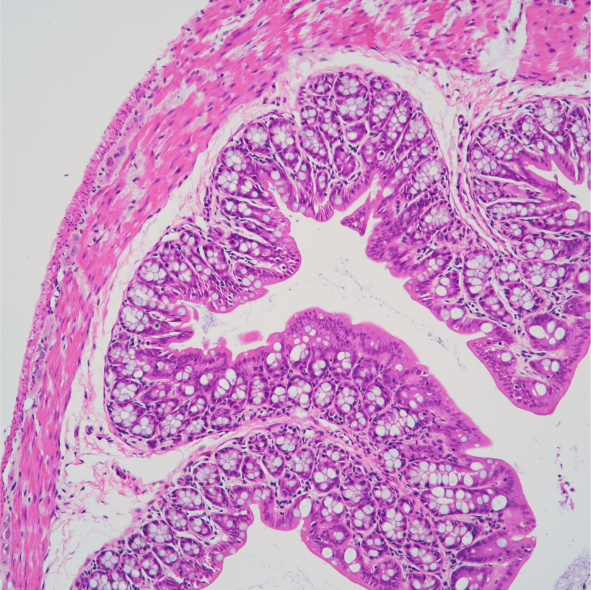

Supplement: Supplementary file 9 — Source data Fig. 7 [file 44318_2024_232_MOESM9_ESM.zip › Figure 7/Figure 7H/Water/Ubac2 shRNA/LIRm.tif]

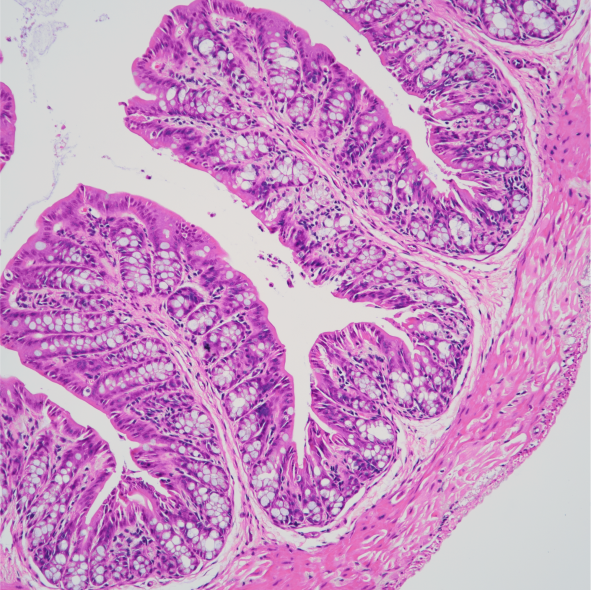

Supplement: Supplementary file 9 — Source data Fig. 7 [file 44318_2024_232_MOESM9_ESM.zip › Figure 7/Figure 7H/Water/Ubac2 shRNA/R277C.tif]

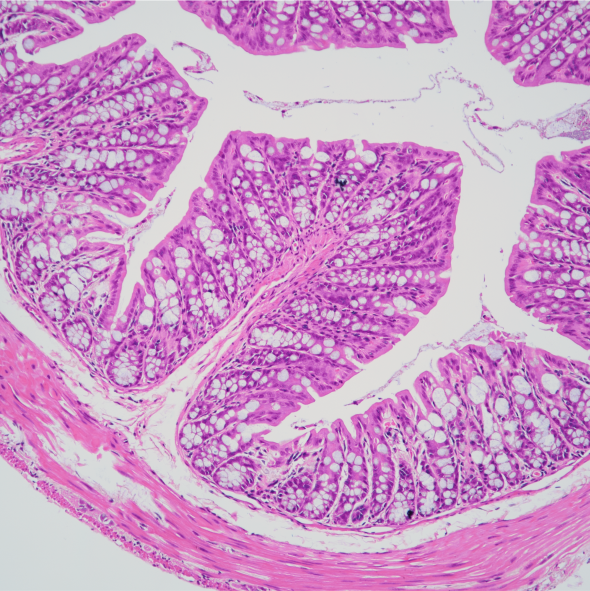

Supplement: Supplementary file 9 — Source data Fig. 7 [file 44318_2024_232_MOESM9_ESM.zip › Figure 7/Figure 7H/Water/Ubac2 shRNA/S223A.tif]

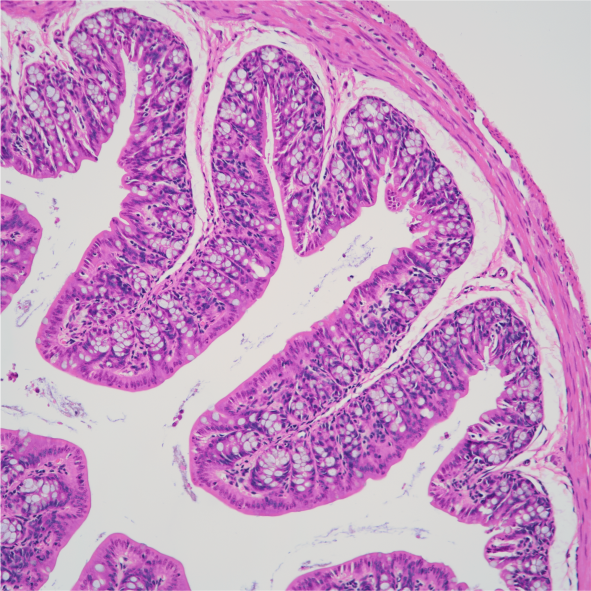

Supplement: Supplementary file 9 — Source data Fig. 7 [file 44318_2024_232_MOESM9_ESM.zip › Figure 7/Figure 7H/Water/Ubac2 shRNA/WT.tif]

Source data: Appendix Figure 1B.

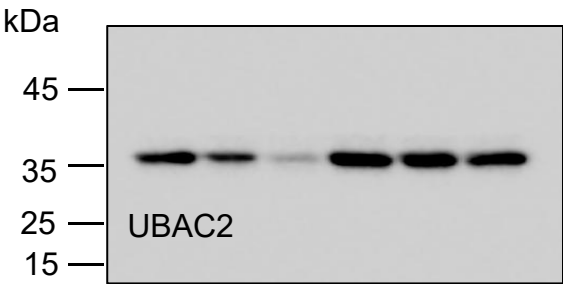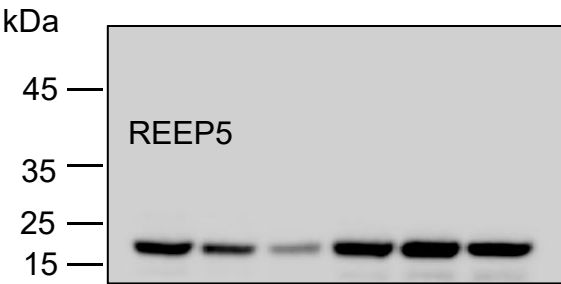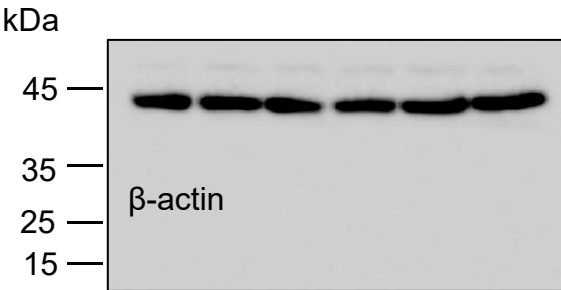

Supplement: Supplementary file 10 — EV and Appendix Figures Source Data [file 44318_2024_232_MOESM10_ESM.zip › EV and Appendix Figures/Appendix Figure 1/Appendix Figure 1B.pdf]

Source data: Appendix Figure 1C.

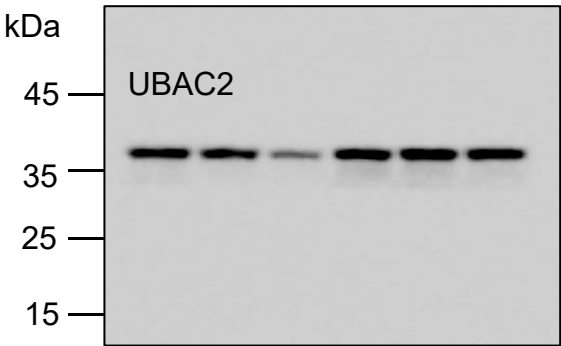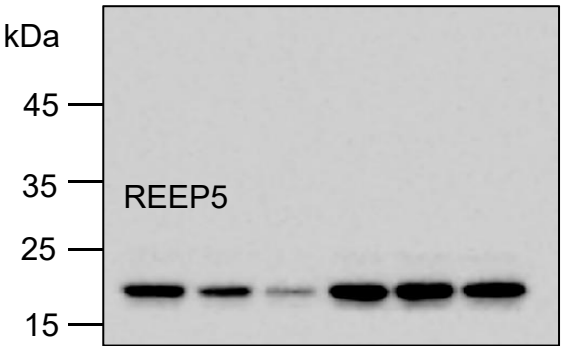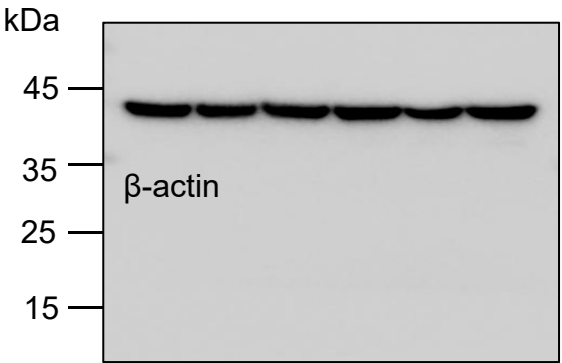

Supplement: Supplementary file 10 — EV and Appendix Figures Source Data [file 44318_2024_232_MOESM10_ESM.zip › EV and Appendix Figures/Appendix Figure 1/Appendix Figure 1C.pdf]

Source data: Appendix Figure 1D.

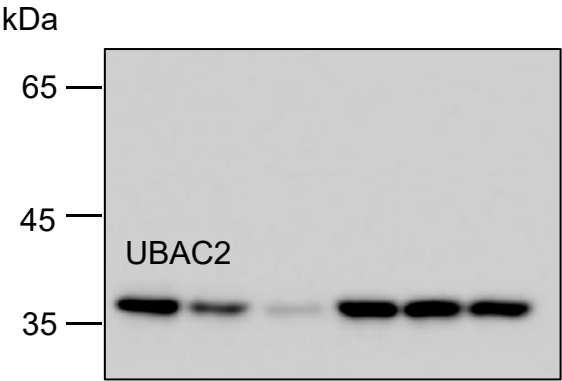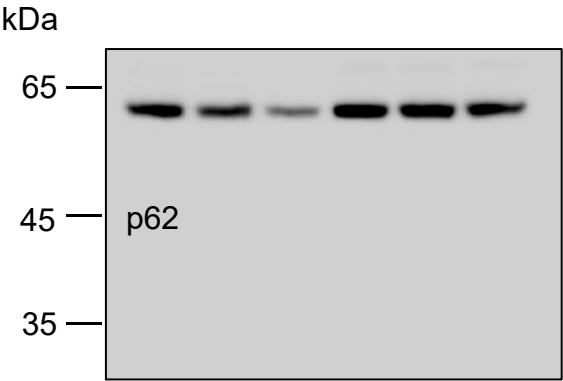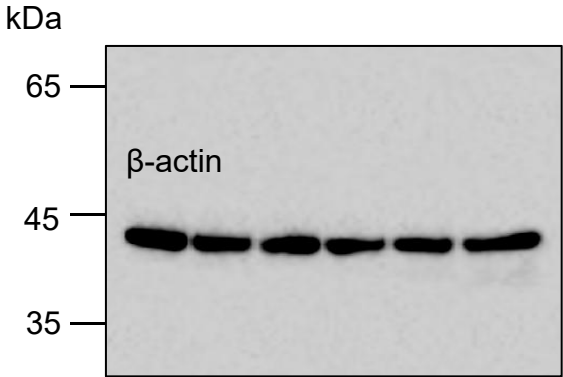

Supplement: Supplementary file 10 — EV and Appendix Figures Source Data [file 44318_2024_232_MOESM10_ESM.zip › EV and Appendix Figures/Appendix Figure 1/Appendix Figure 1D.pdf]

Source data: Appendix Figure 1E.

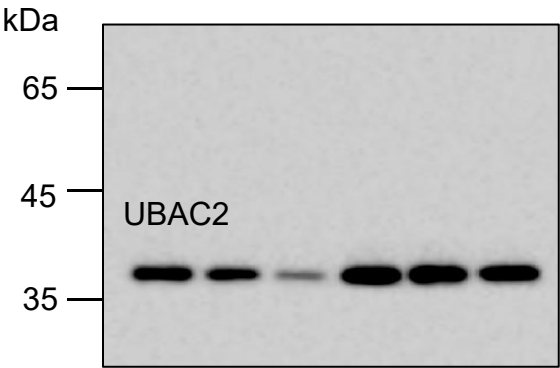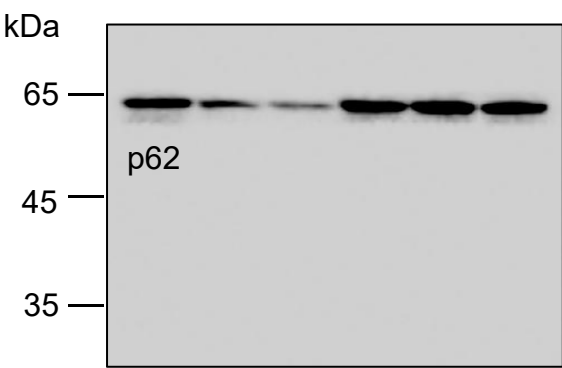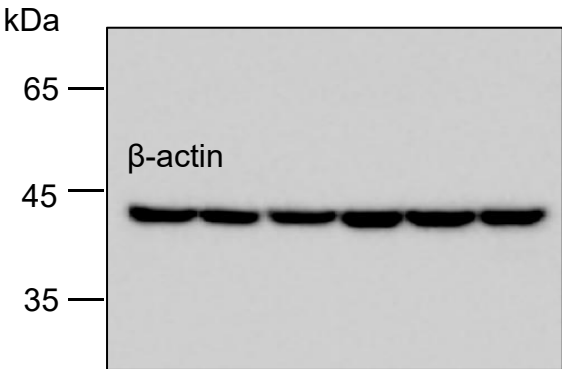

Supplement: Supplementary file 10 — EV and Appendix Figures Source Data [file 44318_2024_232_MOESM10_ESM.zip › EV and Appendix Figures/Appendix Figure 1/Appendix Figure 1E.pdf]

Source data: Appendix Figure 1H.

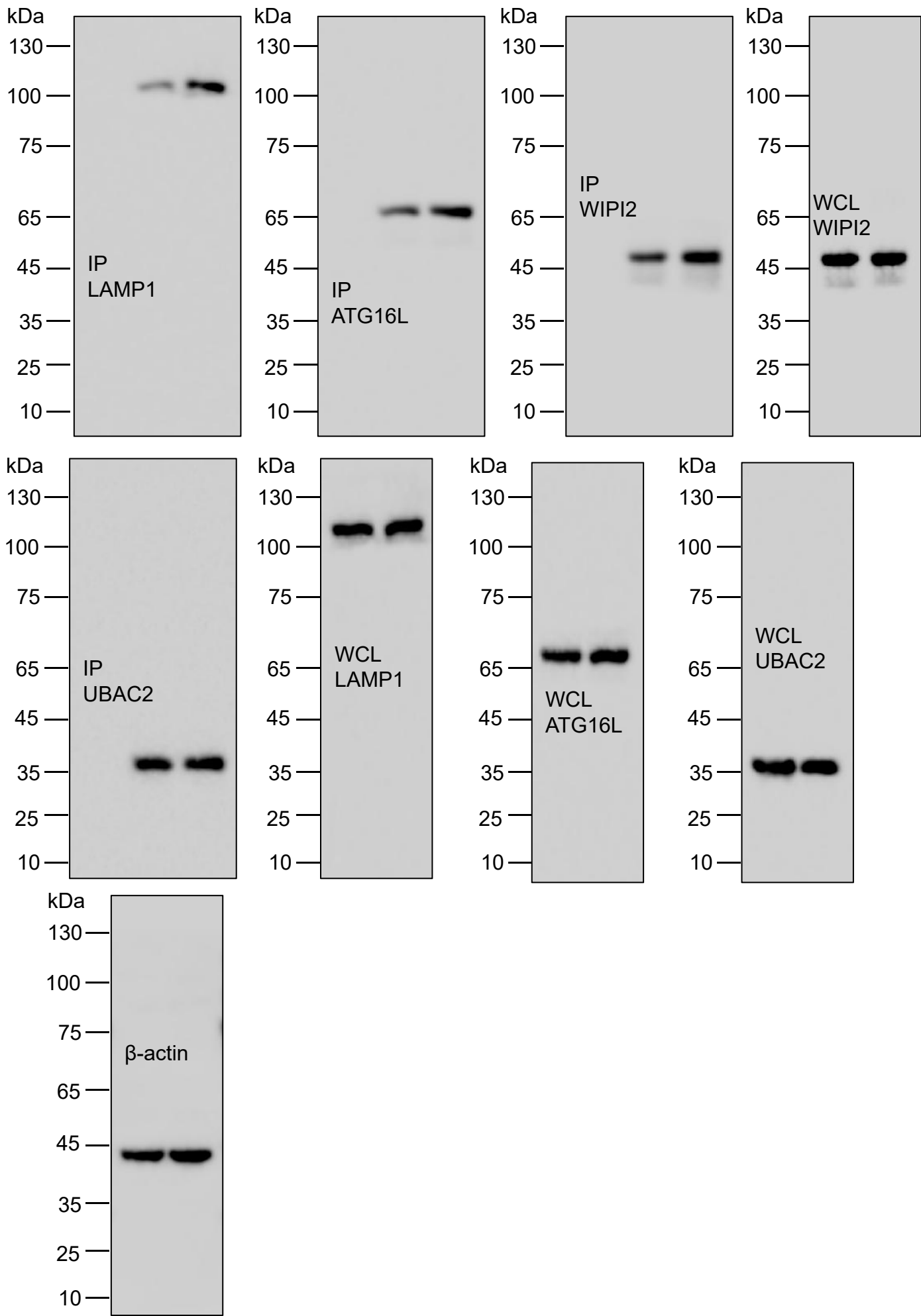

Supplement: Supplementary file 10 — EV and Appendix Figures Source Data [file 44318_2024_232_MOESM10_ESM.zip › EV and Appendix Figures/Appendix Figure 1/Appendix Figure 1H.pdf]

Source data: Appendix Figure 2G.

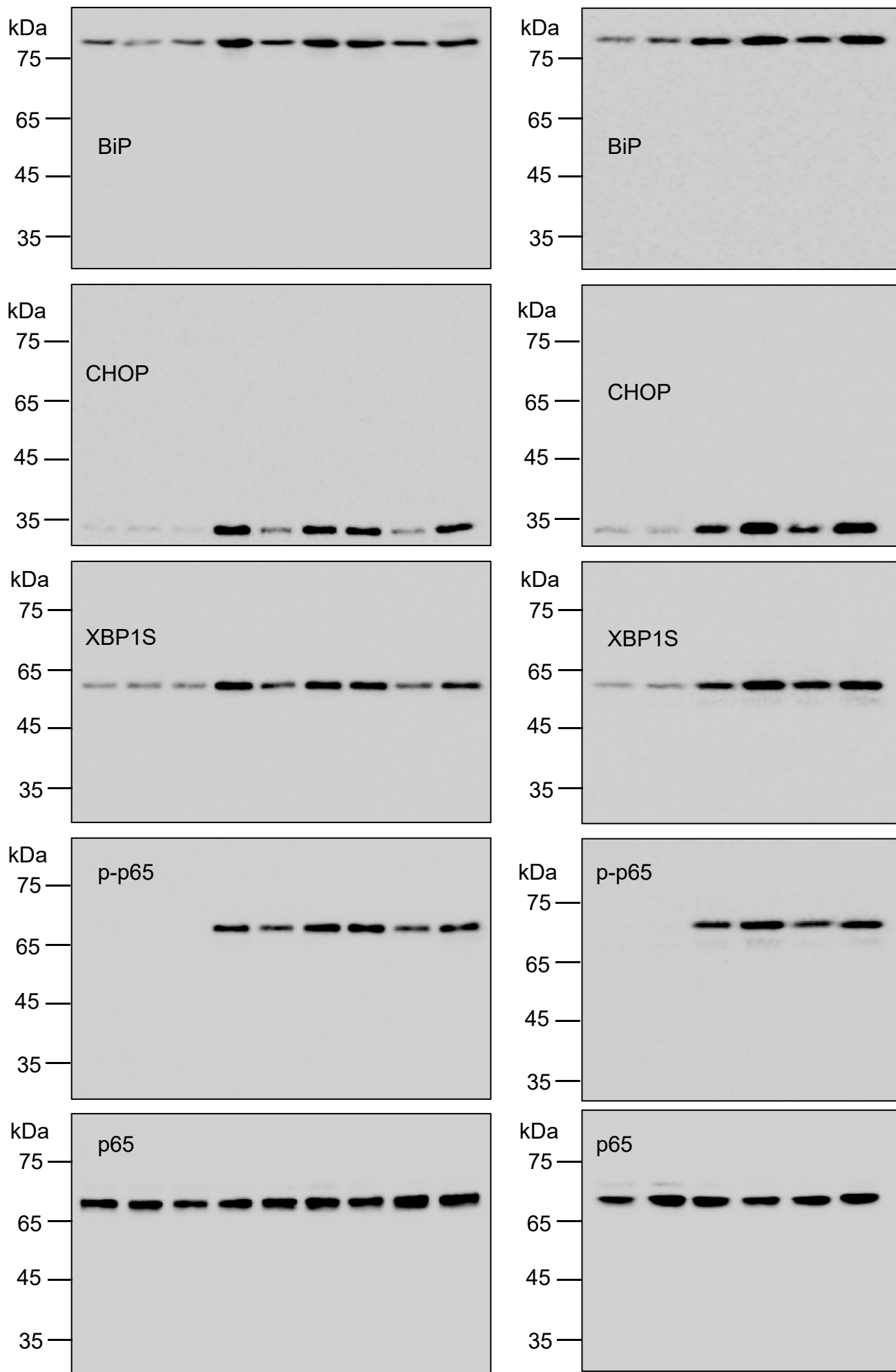

Source data: Appendix Figure 2G.

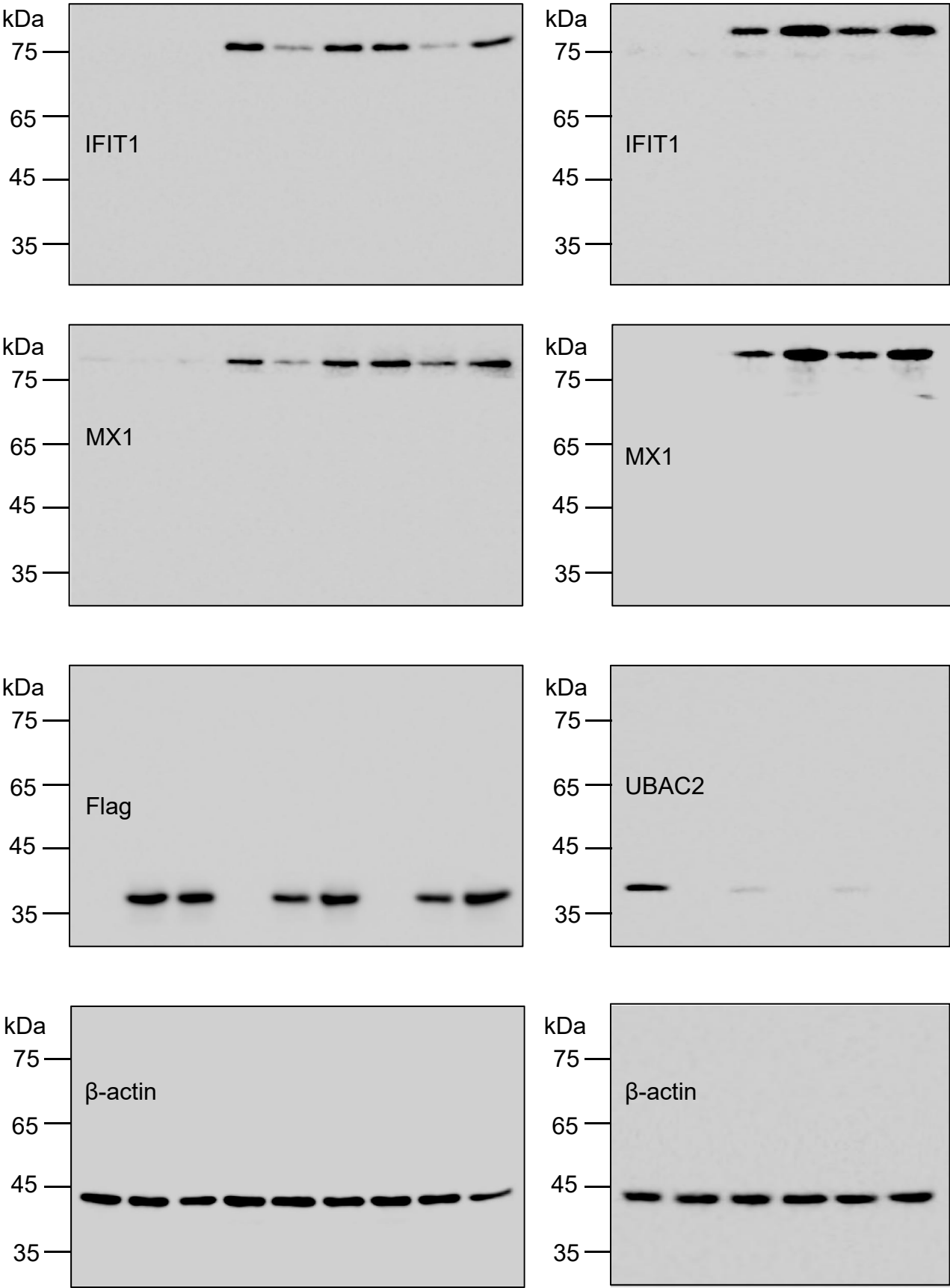

Supplement: Supplementary file 10 — EV and Appendix Figures Source Data [file 44318_2024_232_MOESM10_ESM.zip › EV and Appendix Figures/Appendix Figure 2/Appendix Figure 2G.pdf]

Source data: Figure EV 1B.

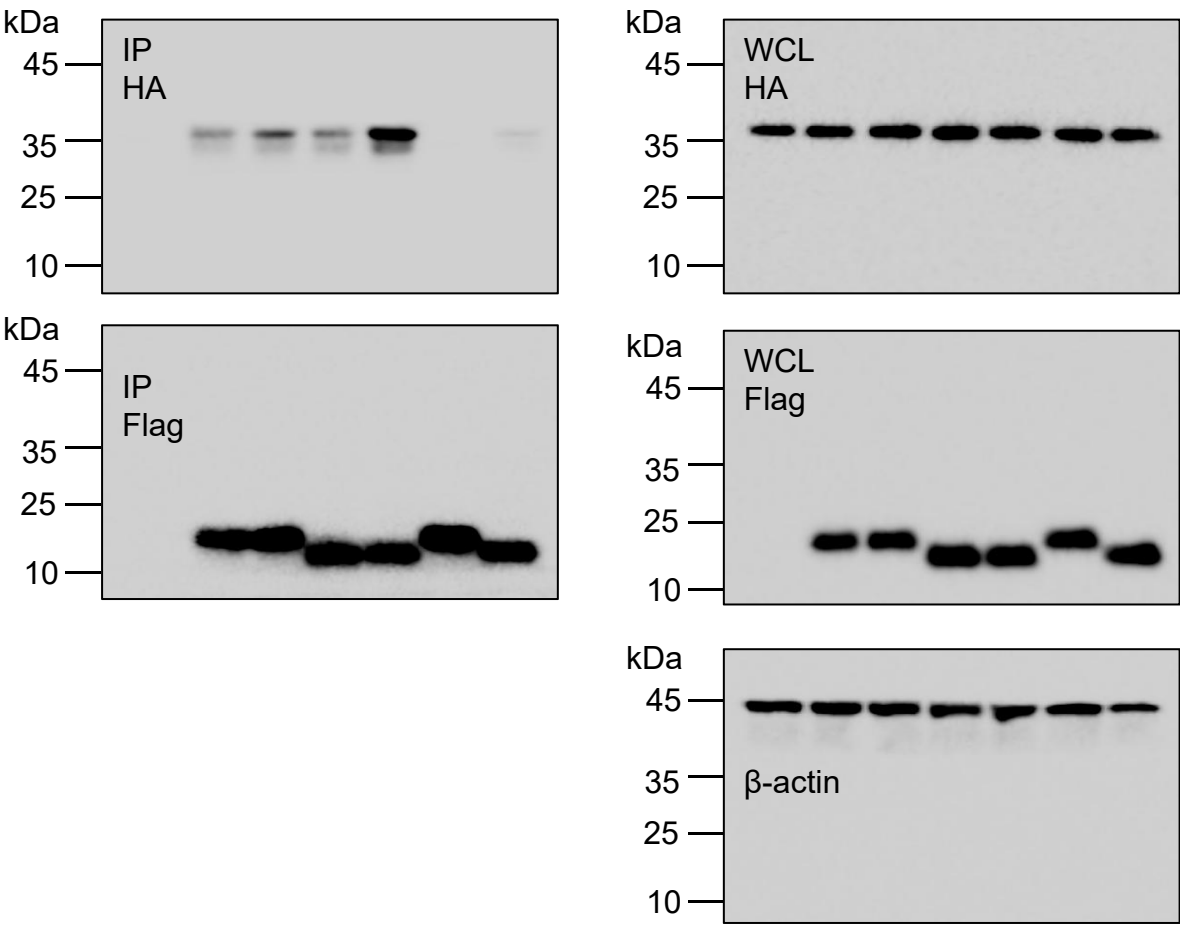

Supplement: Supplementary file 10 — EV and Appendix Figures Source Data [file 44318_2024_232_MOESM10_ESM.zip › EV and Appendix Figures/Figure EV1/Figure EV 1B.pdf]

Source data: Figure EV 1C.

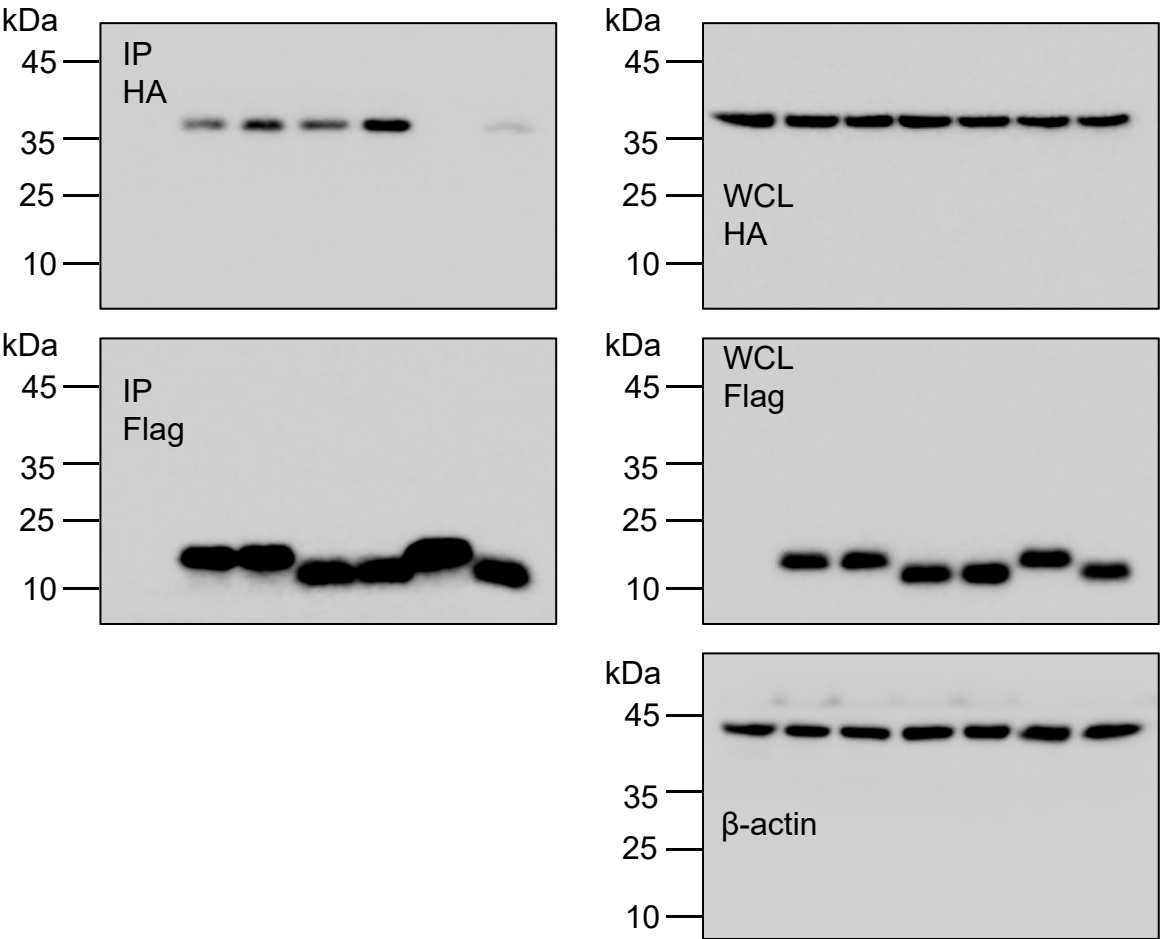

Supplement: Supplementary file 10 — EV and Appendix Figures Source Data [file 44318_2024_232_MOESM10_ESM.zip › EV and Appendix Figures/Figure EV1/Figure EV 1C.pdf]

Source data: Figure EV 1D.

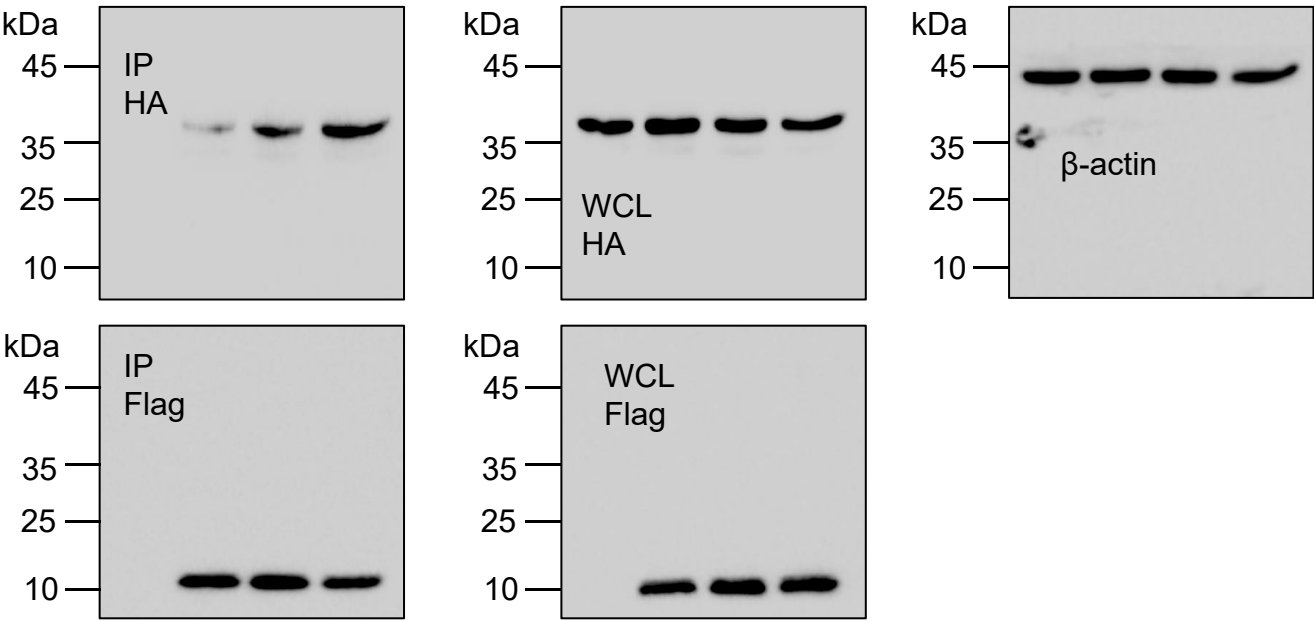

Supplement: Supplementary file 10 — EV and Appendix Figures Source Data [file 44318_2024_232_MOESM10_ESM.zip › EV and Appendix Figures/Figure EV1/Figure EV 1D.pdf]

Source data: Figure EV 1E.

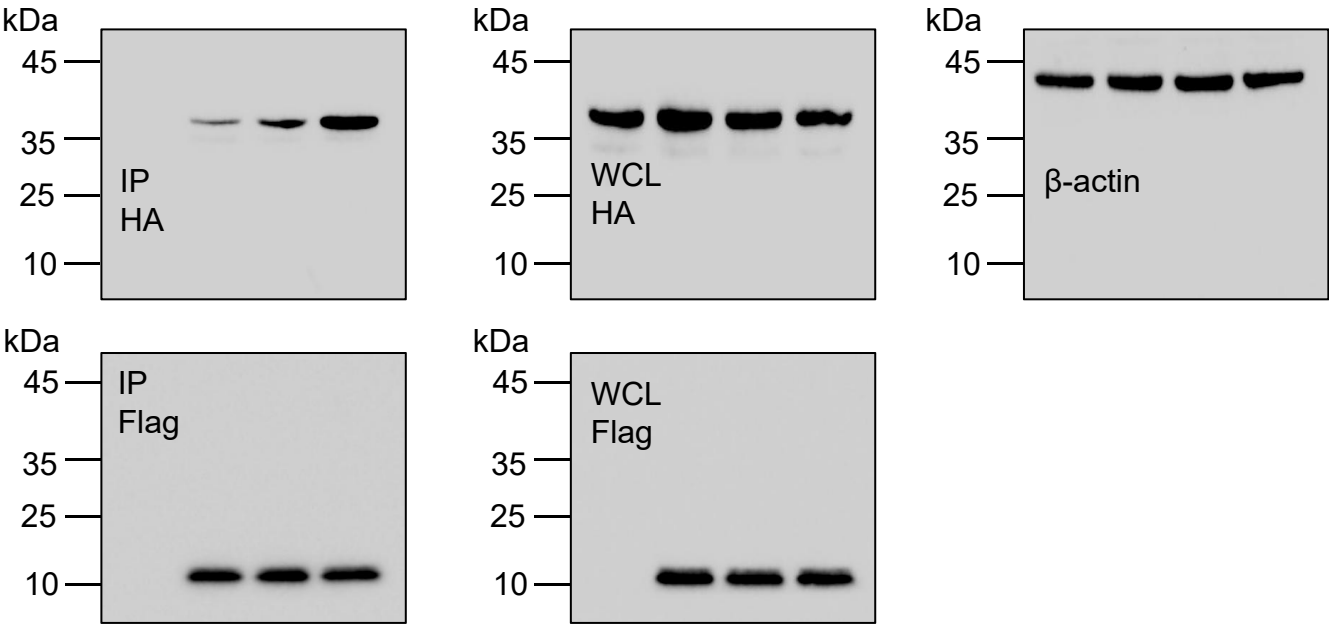

Supplement: Supplementary file 10 — EV and Appendix Figures Source Data [file 44318_2024_232_MOESM10_ESM.zip › EV and Appendix Figures/Figure EV1/Figure EV 1E.pdf]

Source data: Figure EV 1K.

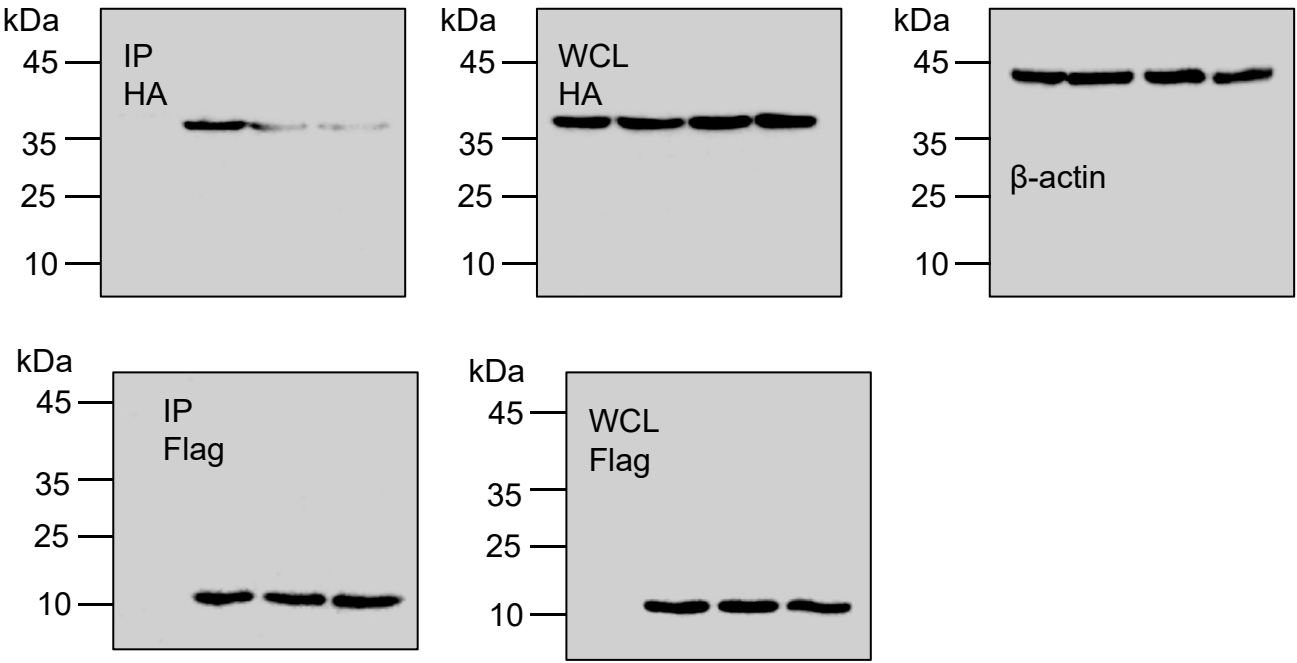

Supplement: Supplementary file 10 — EV and Appendix Figures Source Data [file 44318_2024_232_MOESM10_ESM.zip › EV and Appendix Figures/Figure EV1/Figure EV 1K.pdf]

Source data: Figure EV 2A.

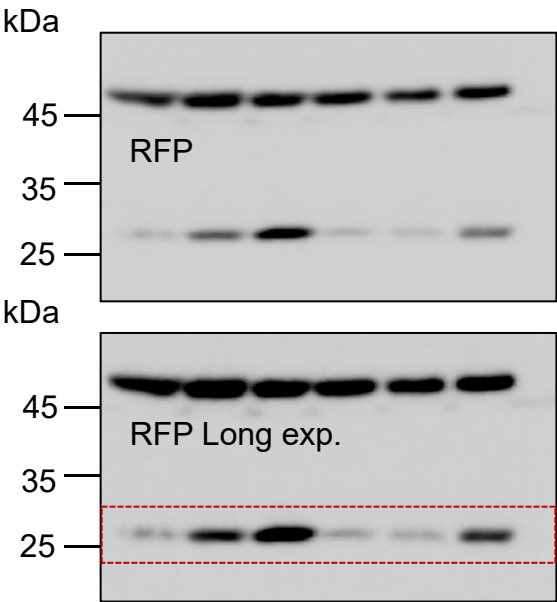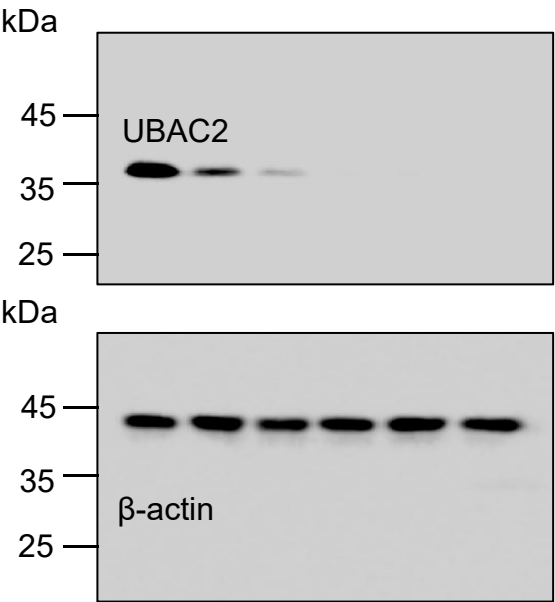

Supplement: Supplementary file 10 — EV and Appendix Figures Source Data [file 44318_2024_232_MOESM10_ESM.zip › EV and Appendix Figures/Figure EV2/Figure EV 2A.pdf]

Source data: Figure EV 2C.

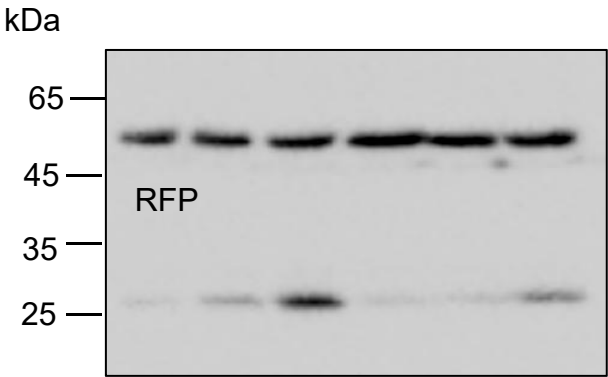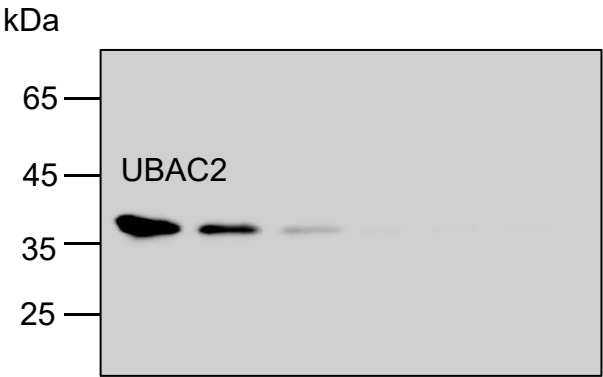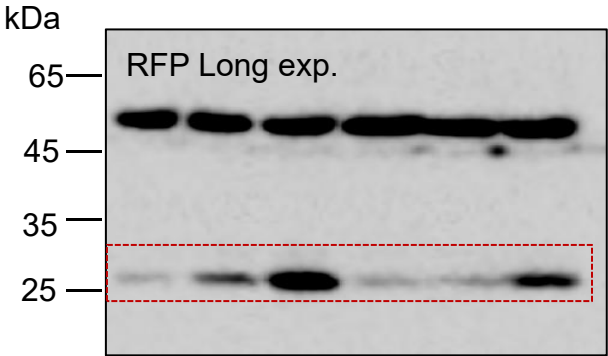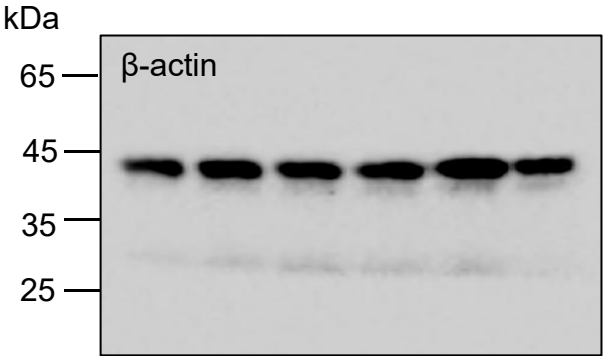

Supplement: Supplementary file 10 — EV and Appendix Figures Source Data [file 44318_2024_232_MOESM10_ESM.zip › EV and Appendix Figures/Figure EV2/Figure EV 2C.pdf]

Source data: Figure EV 2D.

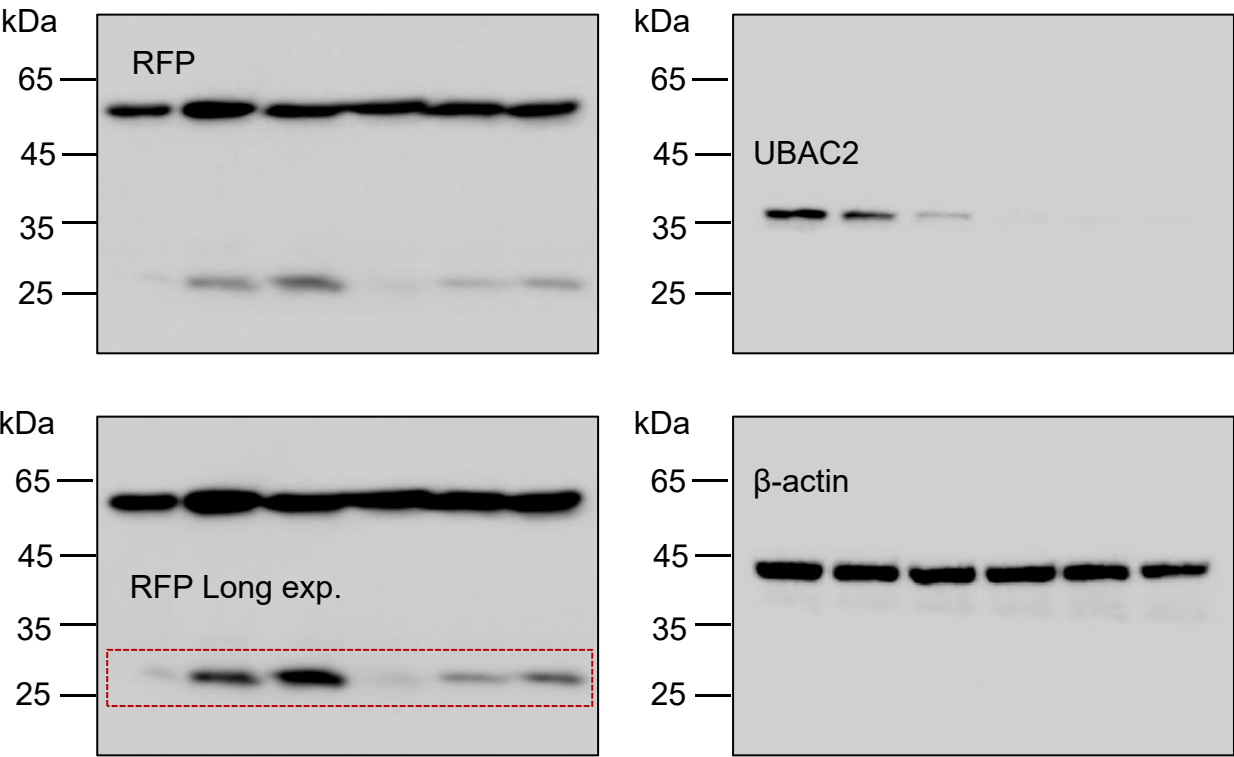

Supplement: Supplementary file 10 — EV and Appendix Figures Source Data [file 44318_2024_232_MOESM10_ESM.zip › EV and Appendix Figures/Figure EV2/Figure EV 2D.pdf]

Source data: Figure EV 2E.

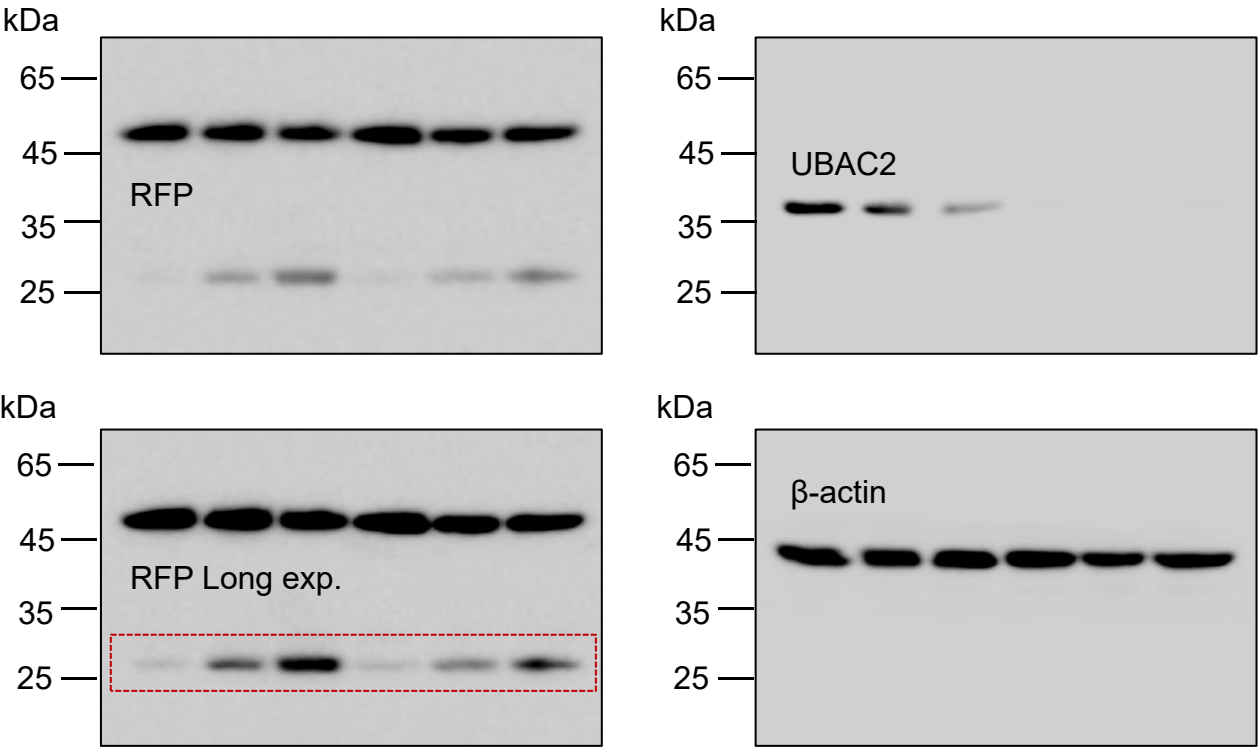

Supplement: Supplementary file 10 — EV and Appendix Figures Source Data [file 44318_2024_232_MOESM10_ESM.zip › EV and Appendix Figures/Figure EV2/Figure EV 2E.pdf]

Source data: Figure EV 2F.

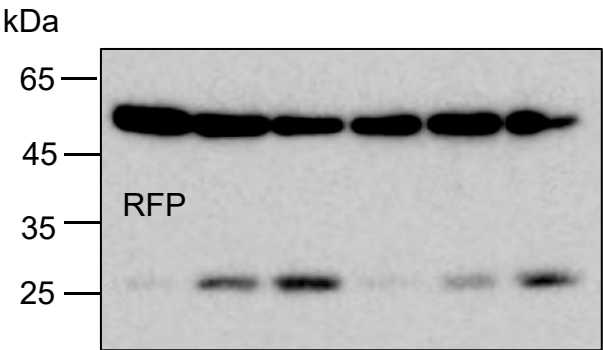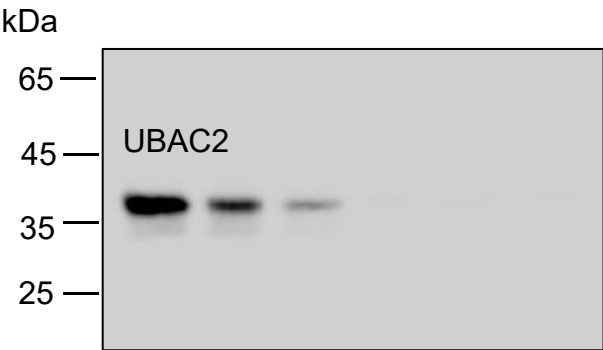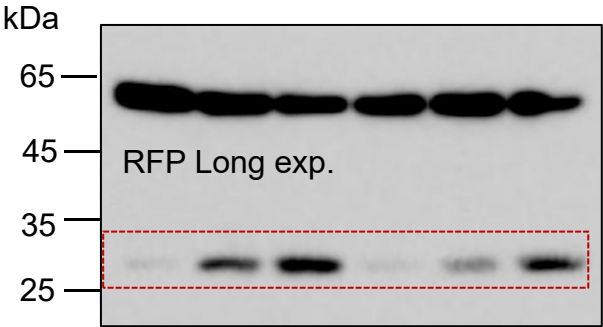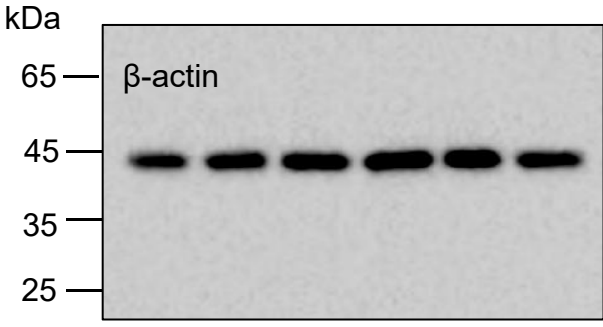

Supplement: Supplementary file 10 — EV and Appendix Figures Source Data [file 44318_2024_232_MOESM10_ESM.zip › EV and Appendix Figures/Figure EV2/Figure EV 2F.pdf]

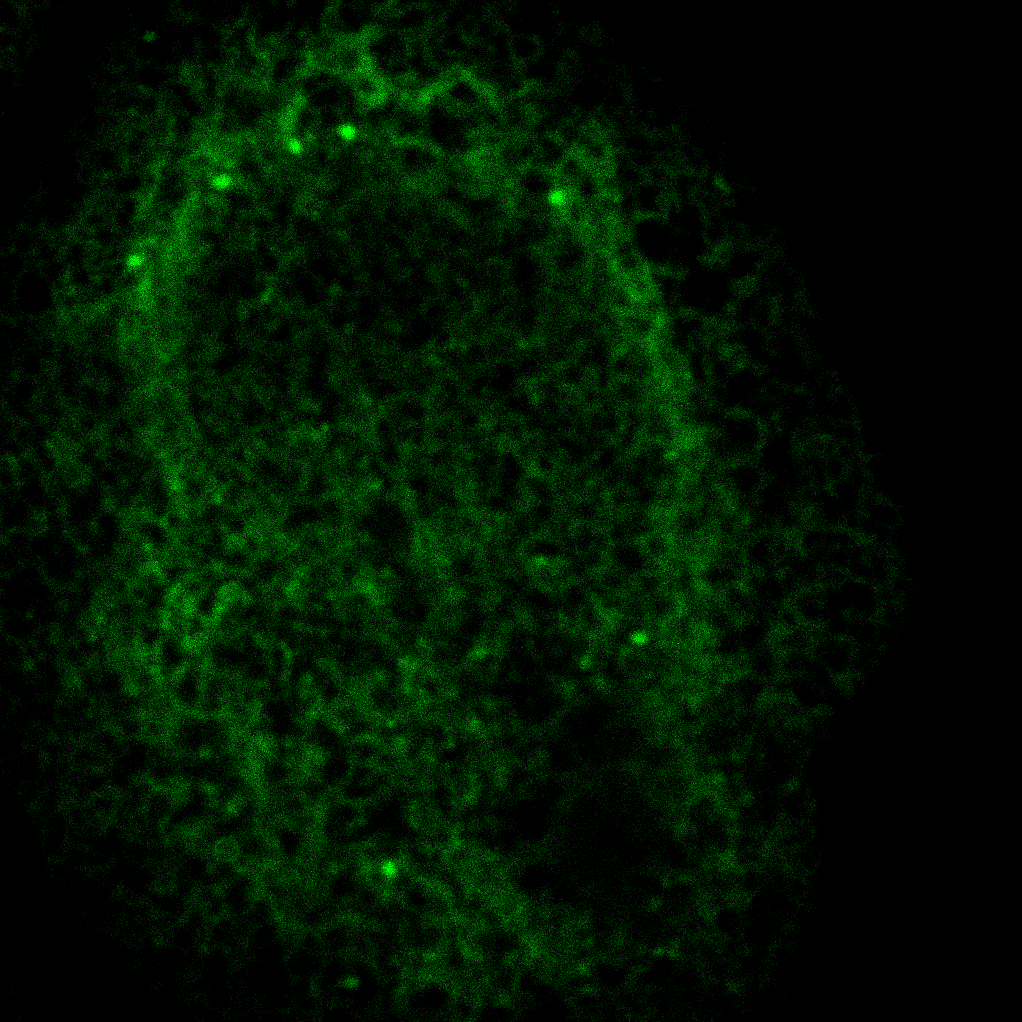

Supplement: Supplementary file 10 — EV and Appendix Figures Source Data [file 44318_2024_232_MOESM10_ESM.zip › EV and Appendix Figures/Figure EV2/Figure EV 2G/Mock/UBAC2 KO/GFP.tif]

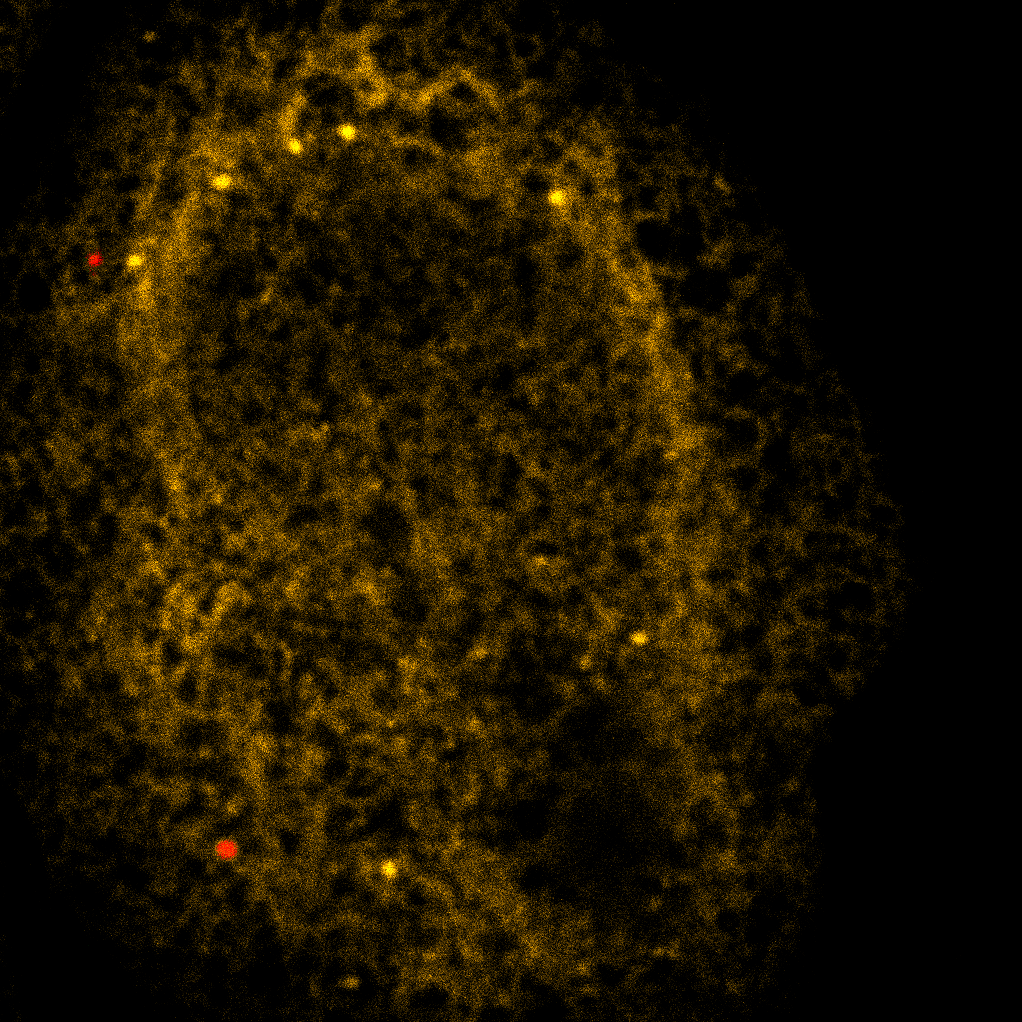

Supplement: Supplementary file 10 — EV and Appendix Figures Source Data [file 44318_2024_232_MOESM10_ESM.zip › EV and Appendix Figures/Figure EV2/Figure EV 2G/Mock/UBAC2 KO/Merge.tif]

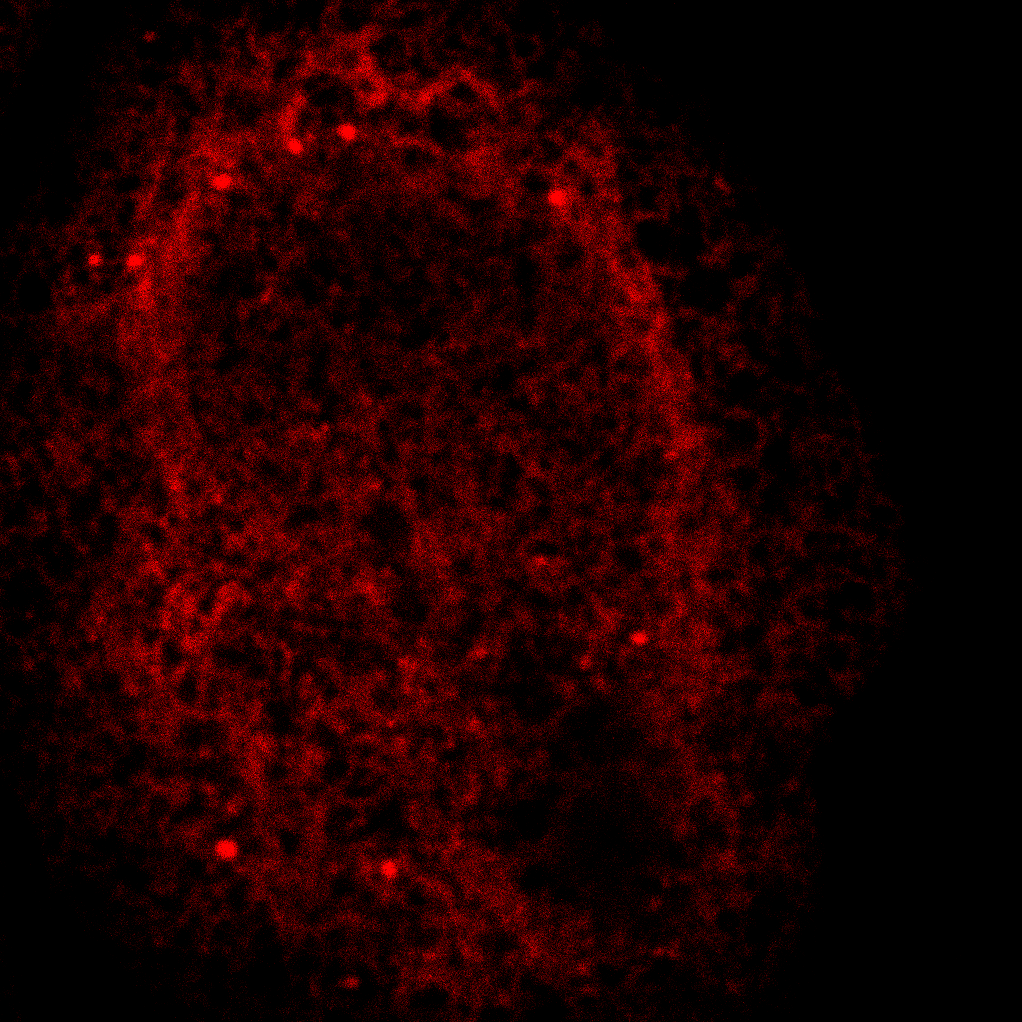

Supplement: Supplementary file 10 — EV and Appendix Figures Source Data [file 44318_2024_232_MOESM10_ESM.zip › EV and Appendix Figures/Figure EV2/Figure EV 2G/Mock/UBAC2 KO/RFP.tif]

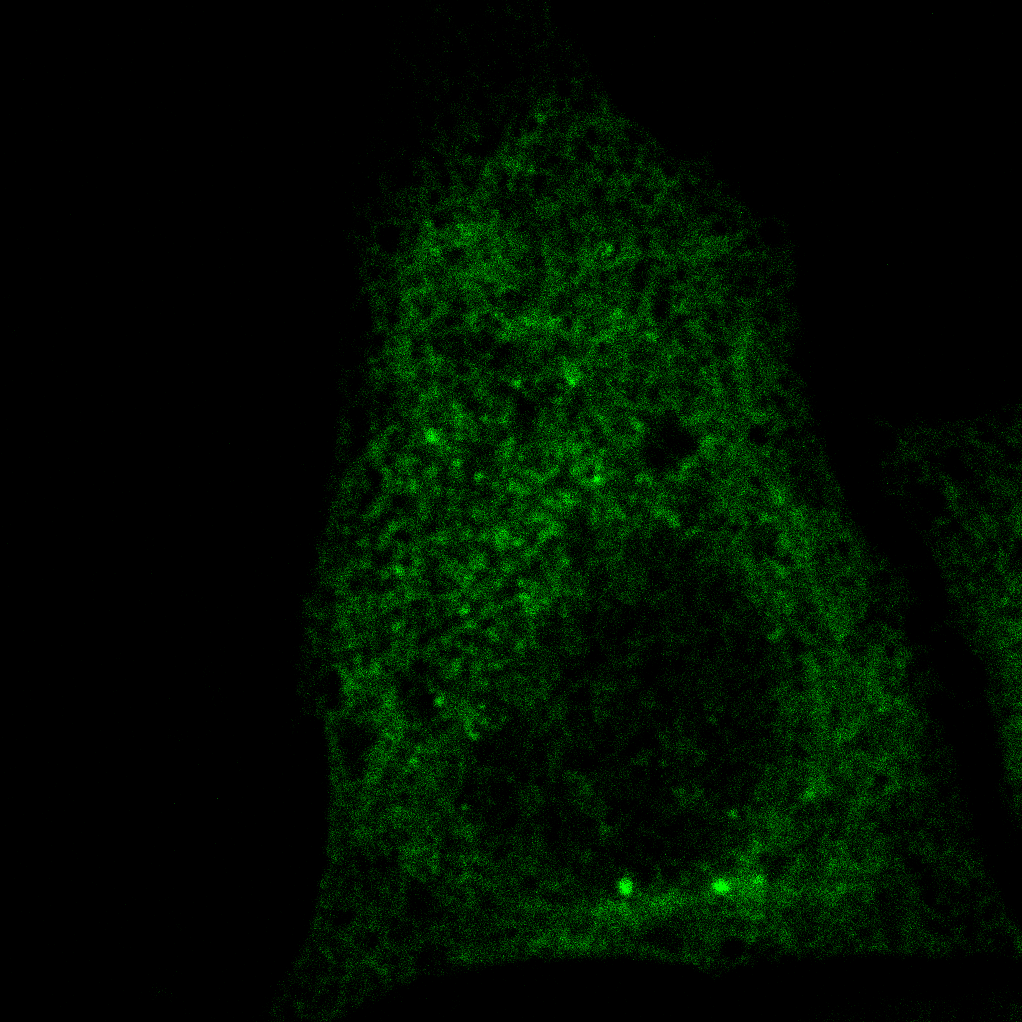

Supplement: Supplementary file 10 — EV and Appendix Figures Source Data [file 44318_2024_232_MOESM10_ESM.zip › EV and Appendix Figures/Figure EV2/Figure EV 2G/Mock/WT/GFP.tif]

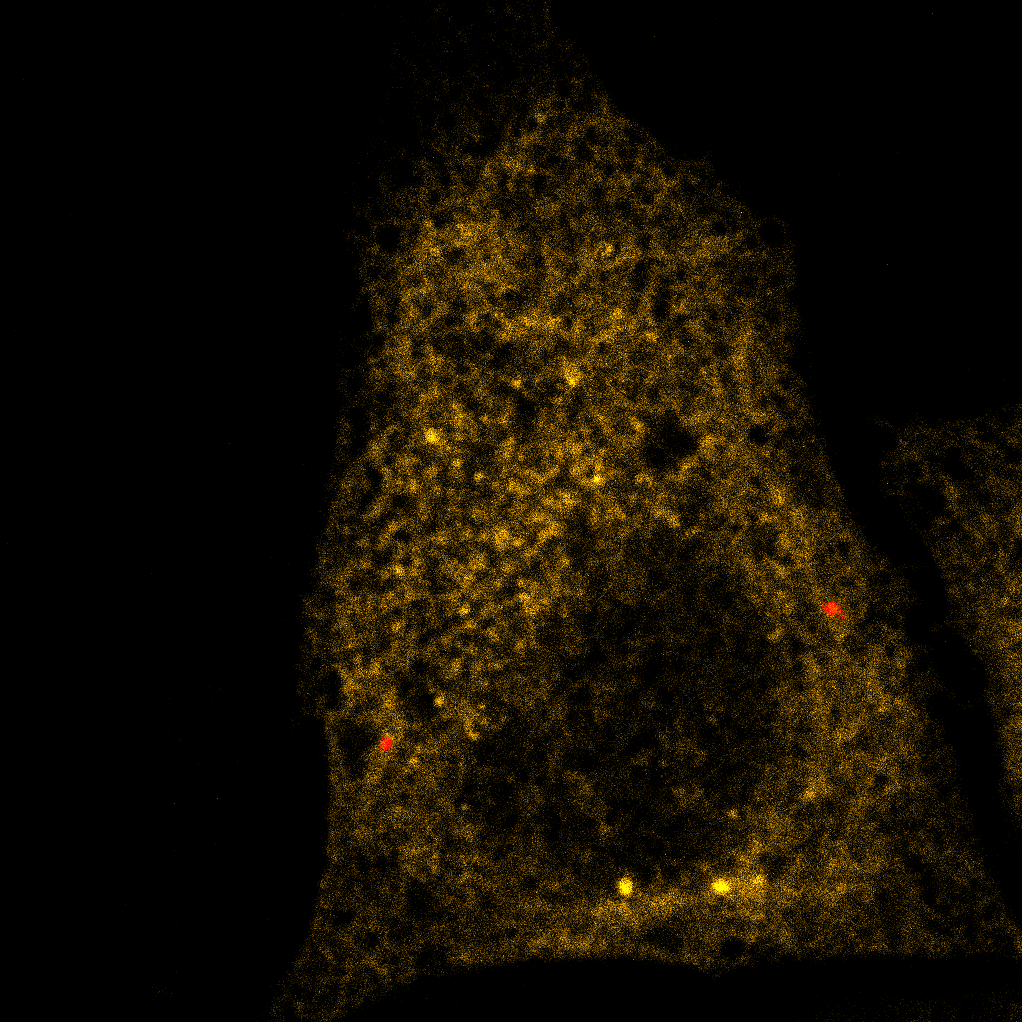

Supplement: Supplementary file 10 — EV and Appendix Figures Source Data [file 44318_2024_232_MOESM10_ESM.zip › EV and Appendix Figures/Figure EV2/Figure EV 2G/Mock/WT/Merge.tif]

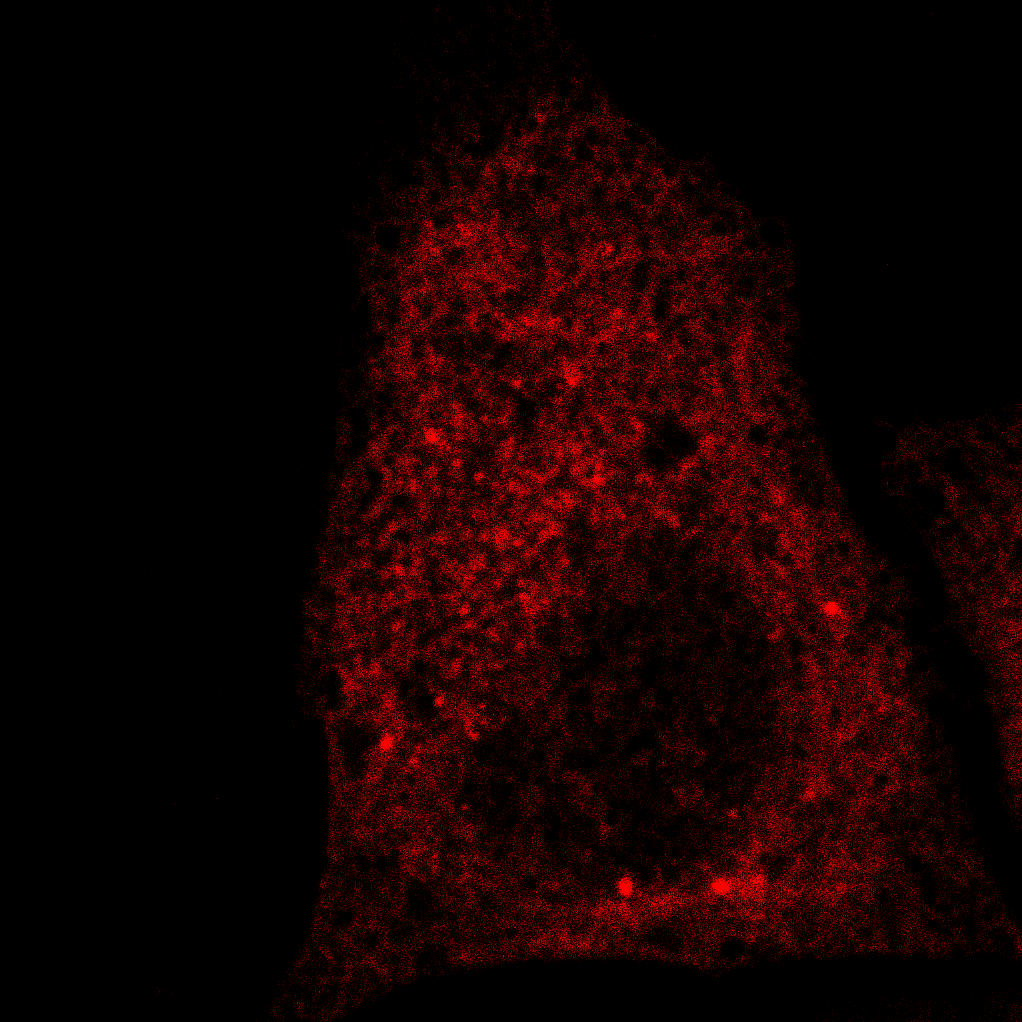

Supplement: Supplementary file 10 — EV and Appendix Figures Source Data [file 44318_2024_232_MOESM10_ESM.zip › EV and Appendix Figures/Figure EV2/Figure EV 2G/Mock/WT/RFP.tif]

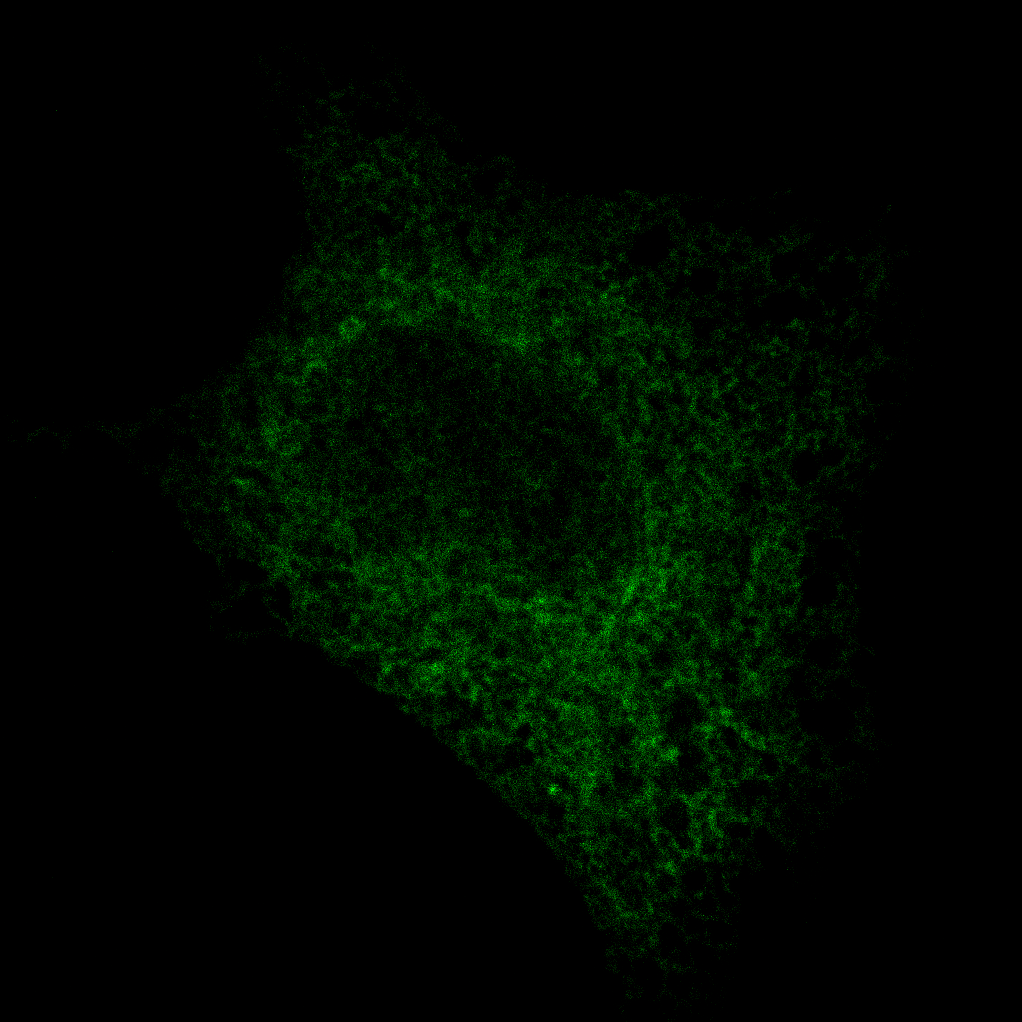

Supplement: Supplementary file 10 — EV and Appendix Figures Source Data [file 44318_2024_232_MOESM10_ESM.zip › EV and Appendix Figures/Figure EV2/Figure EV 2G/TG/UBAC2 KO/GFP.tif]

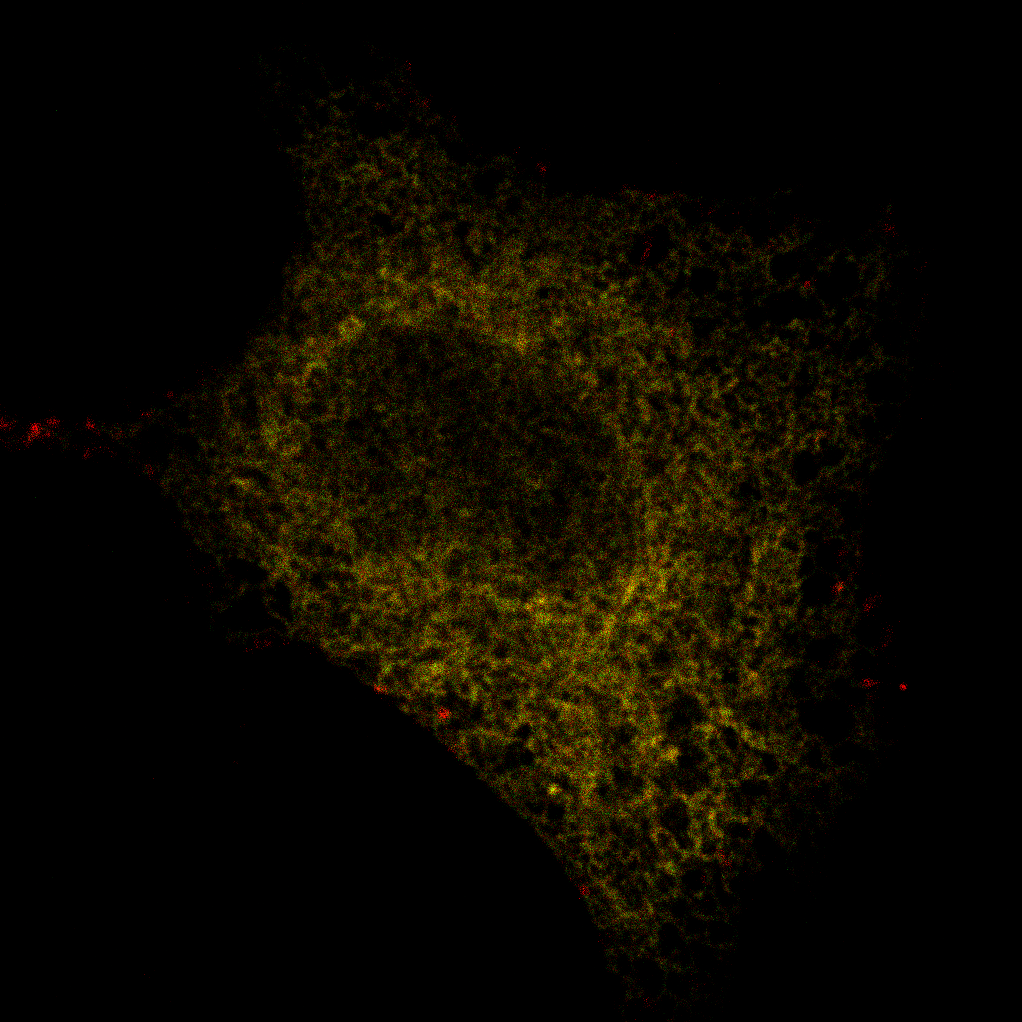

Supplement: Supplementary file 10 — EV and Appendix Figures Source Data [file 44318_2024_232_MOESM10_ESM.zip › EV and Appendix Figures/Figure EV2/Figure EV 2G/TG/UBAC2 KO/Merge.tif]

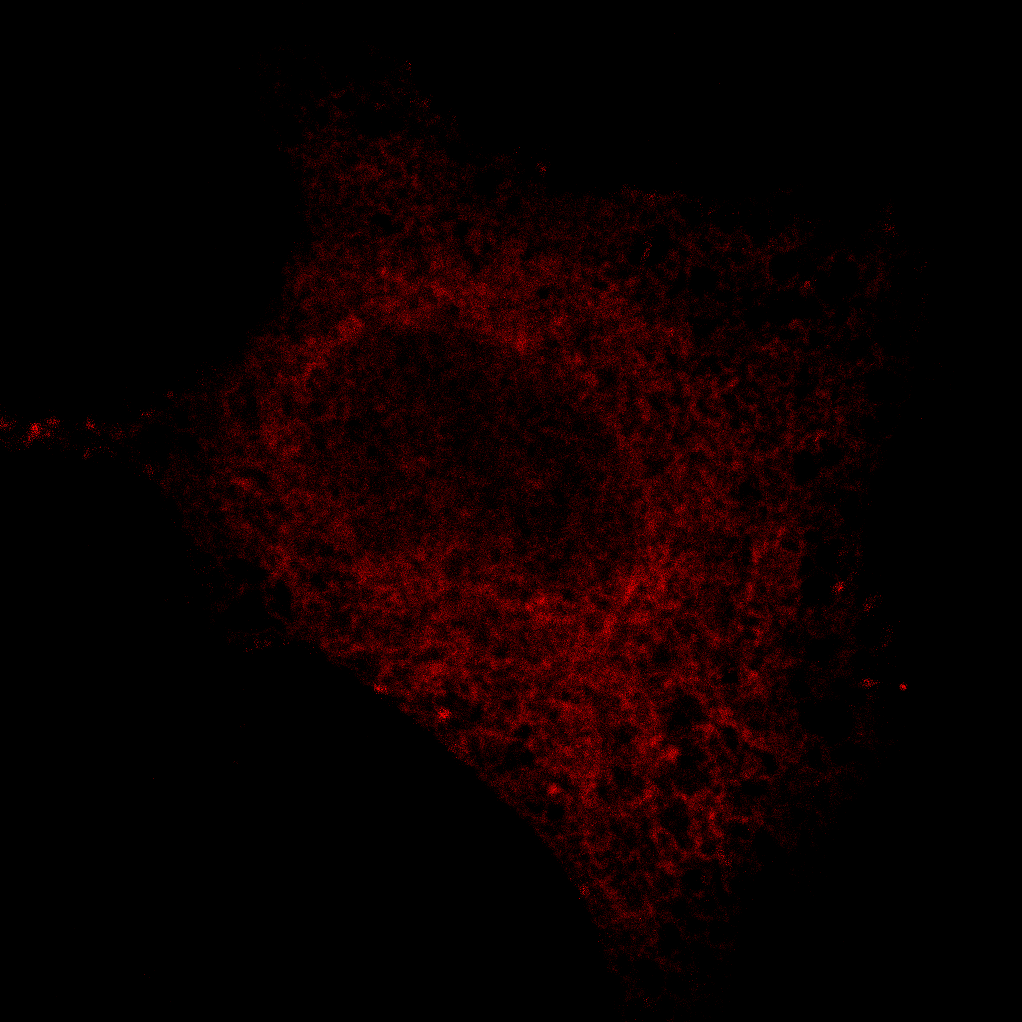

Supplement: Supplementary file 10 — EV and Appendix Figures Source Data [file 44318_2024_232_MOESM10_ESM.zip › EV and Appendix Figures/Figure EV2/Figure EV 2G/TG/UBAC2 KO/RFP.tif]

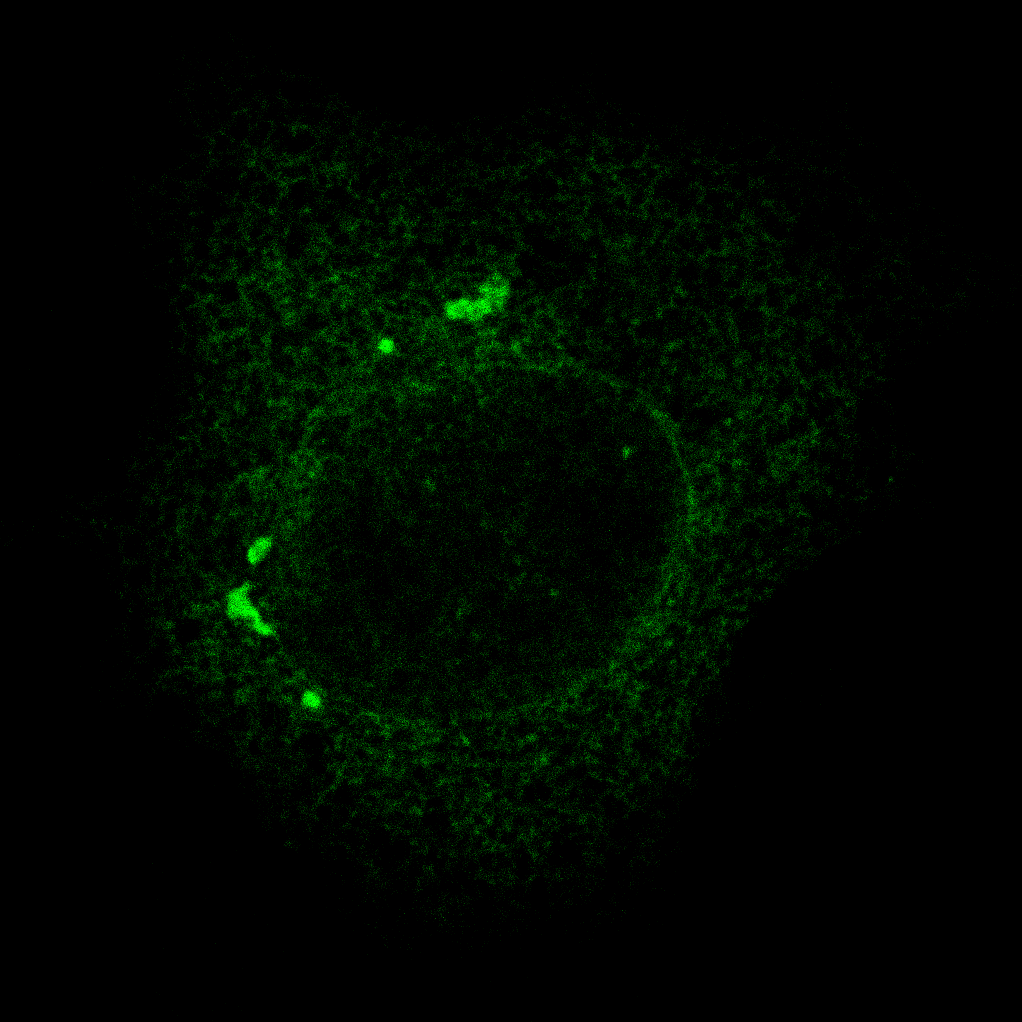

Supplement: Supplementary file 10 — EV and Appendix Figures Source Data [file 44318_2024_232_MOESM10_ESM.zip › EV and Appendix Figures/Figure EV2/Figure EV 2G/TG/WT/GFP.tif]

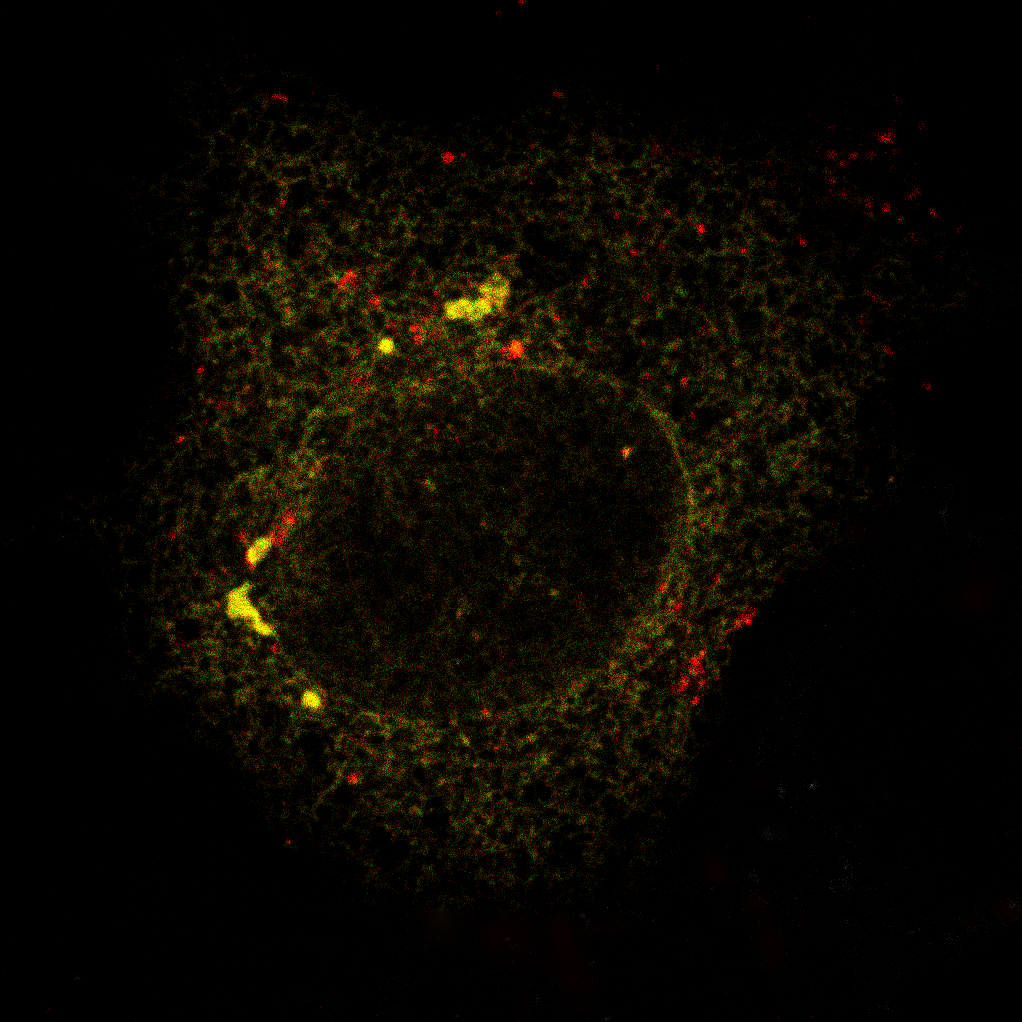

Supplement: Supplementary file 10 — EV and Appendix Figures Source Data [file 44318_2024_232_MOESM10_ESM.zip › EV and Appendix Figures/Figure EV2/Figure EV 2G/TG/WT/Merge.tif]

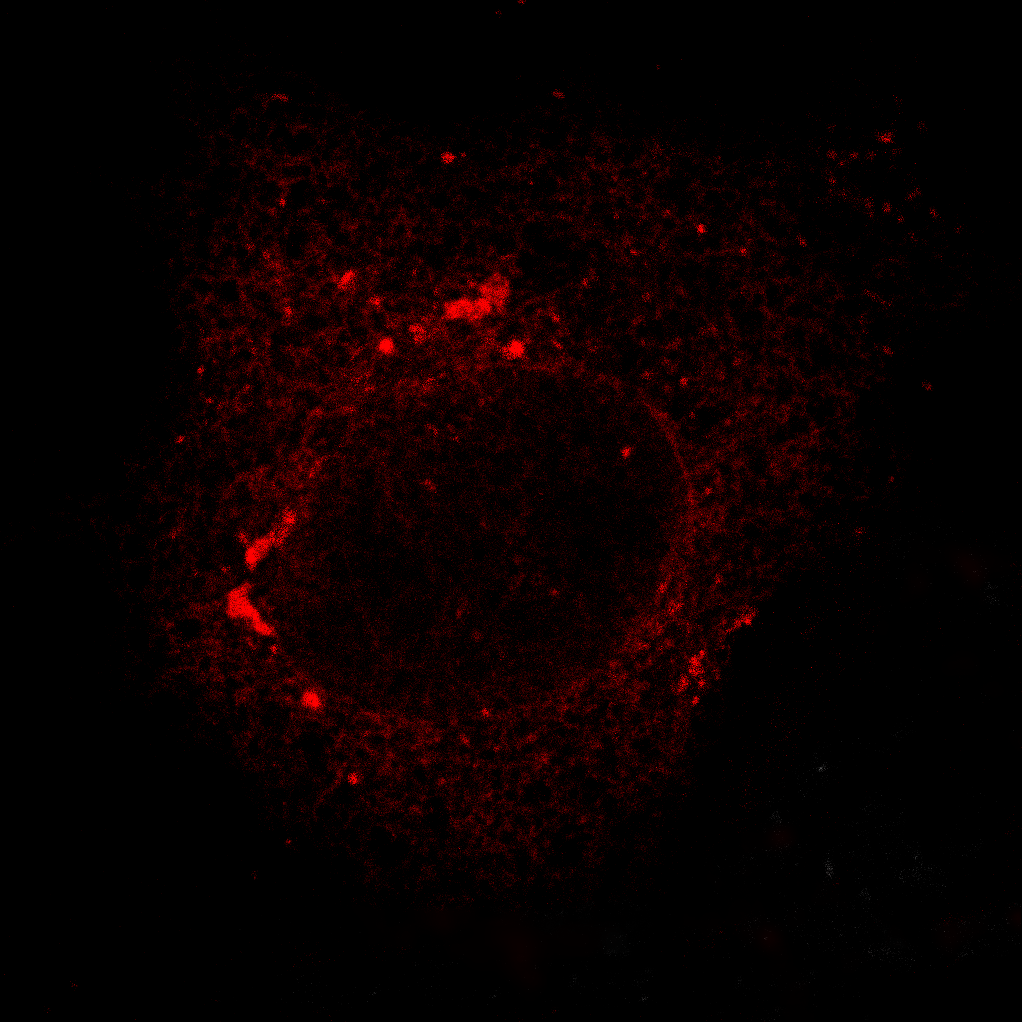

Supplement: Supplementary file 10 — EV and Appendix Figures Source Data [file 44318_2024_232_MOESM10_ESM.zip › EV and Appendix Figures/Figure EV2/Figure EV 2G/TG/WT/RFP.tif]

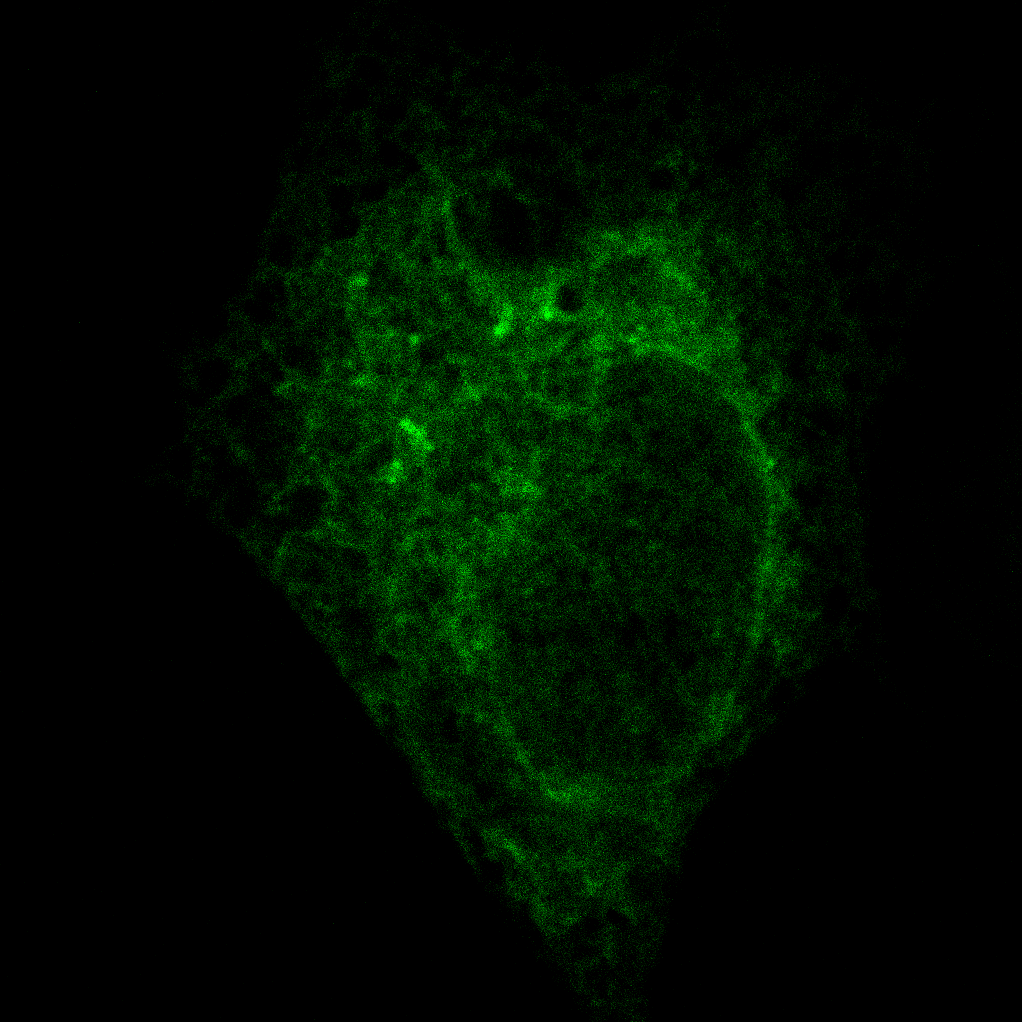

Supplement: Supplementary file 10 — EV and Appendix Figures Source Data [file 44318_2024_232_MOESM10_ESM.zip › EV and Appendix Figures/Figure EV2/Figure EV 2I/Mock/-/GFP.tif]

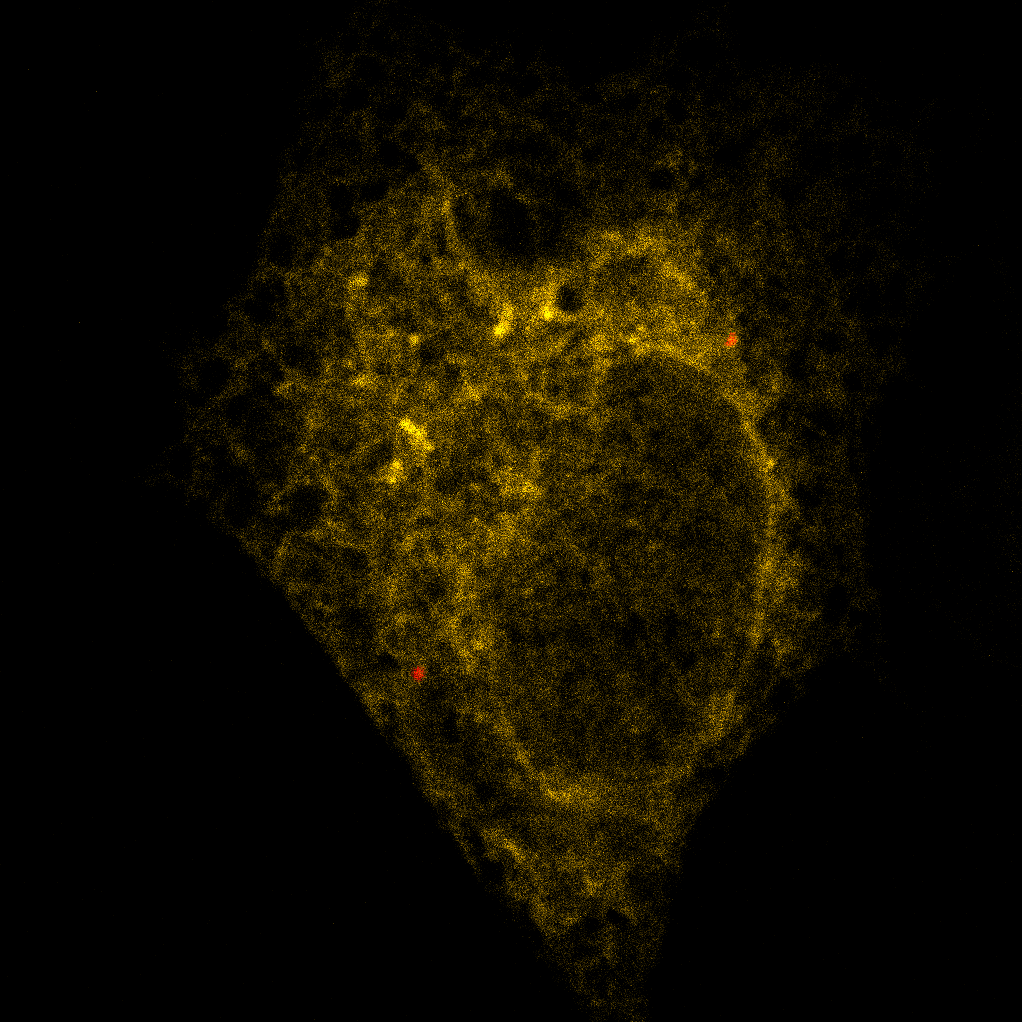

Supplement: Supplementary file 10 — EV and Appendix Figures Source Data [file 44318_2024_232_MOESM10_ESM.zip › EV and Appendix Figures/Figure EV2/Figure EV 2I/Mock/-/Merge.tif]

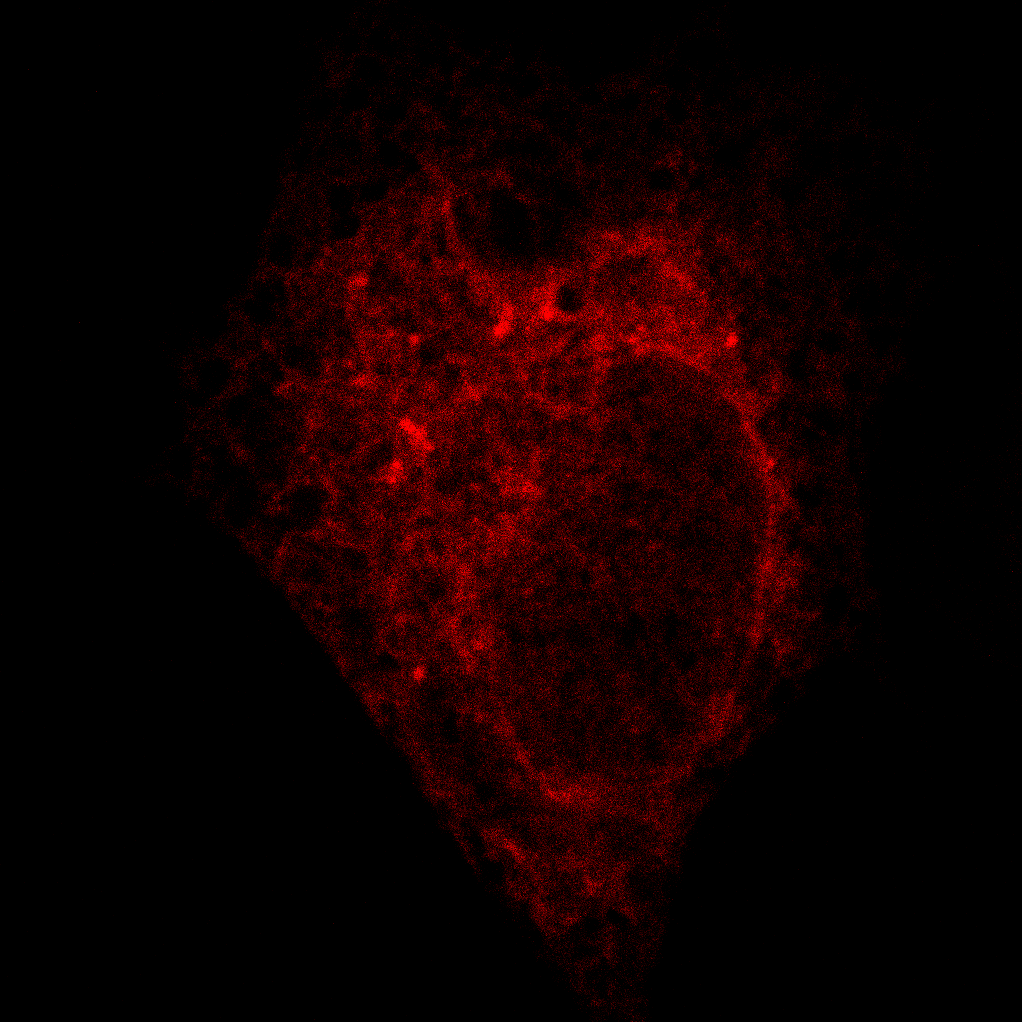

Supplement: Supplementary file 10 — EV and Appendix Figures Source Data [file 44318_2024_232_MOESM10_ESM.zip › EV and Appendix Figures/Figure EV2/Figure EV 2I/Mock/-/RFP.tif]

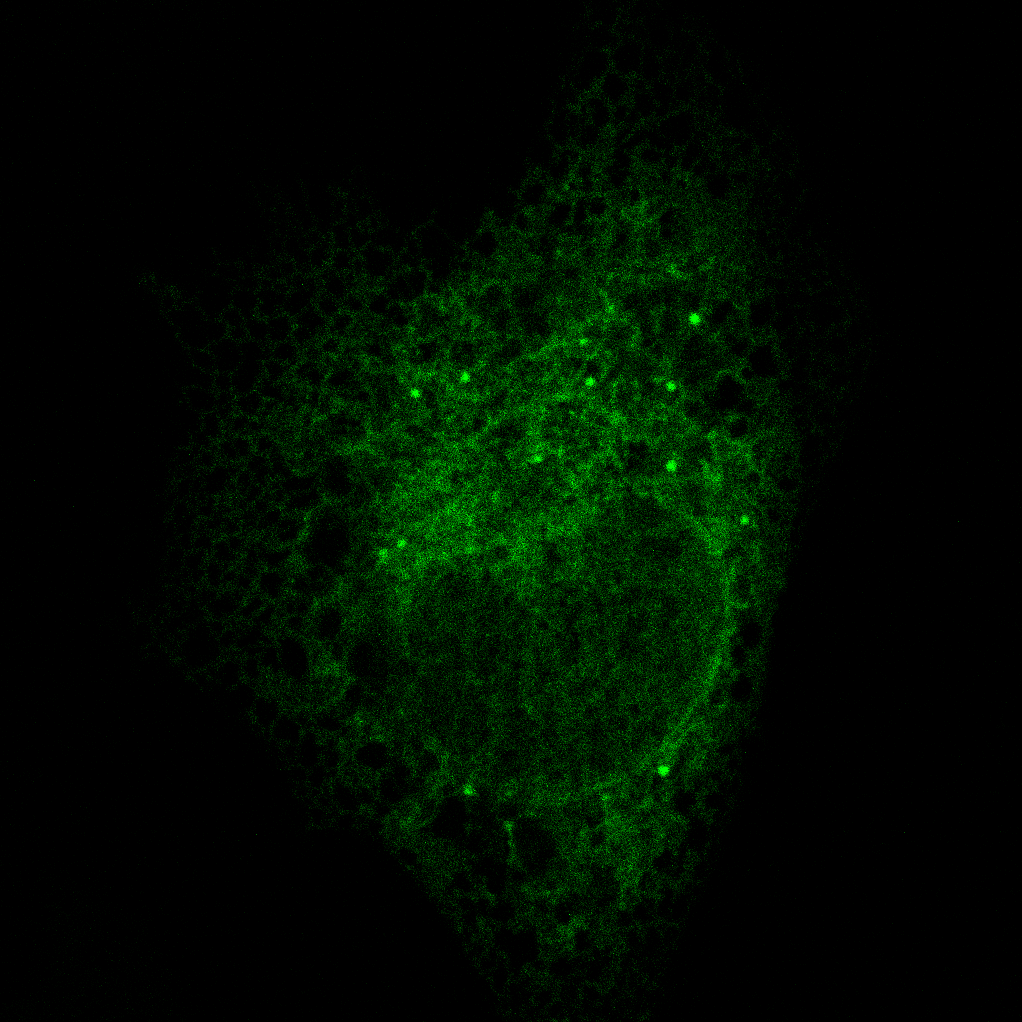

Supplement: Supplementary file 10 — EV and Appendix Figures Source Data [file 44318_2024_232_MOESM10_ESM.zip › EV and Appendix Figures/Figure EV2/Figure EV 2I/Mock/LIRM/GFP.tif]

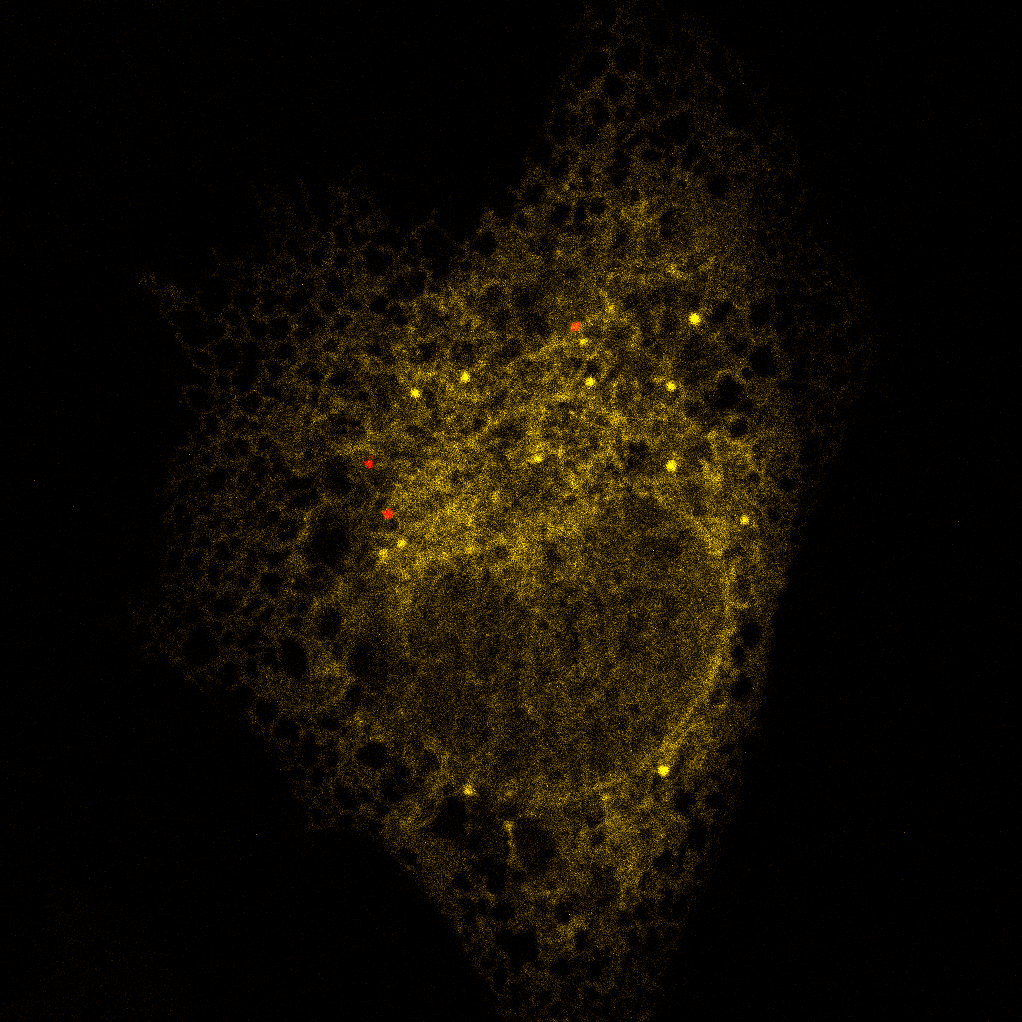

Supplement: Supplementary file 10 — EV and Appendix Figures Source Data [file 44318_2024_232_MOESM10_ESM.zip › EV and Appendix Figures/Figure EV2/Figure EV 2I/Mock/LIRM/Merge.tif]

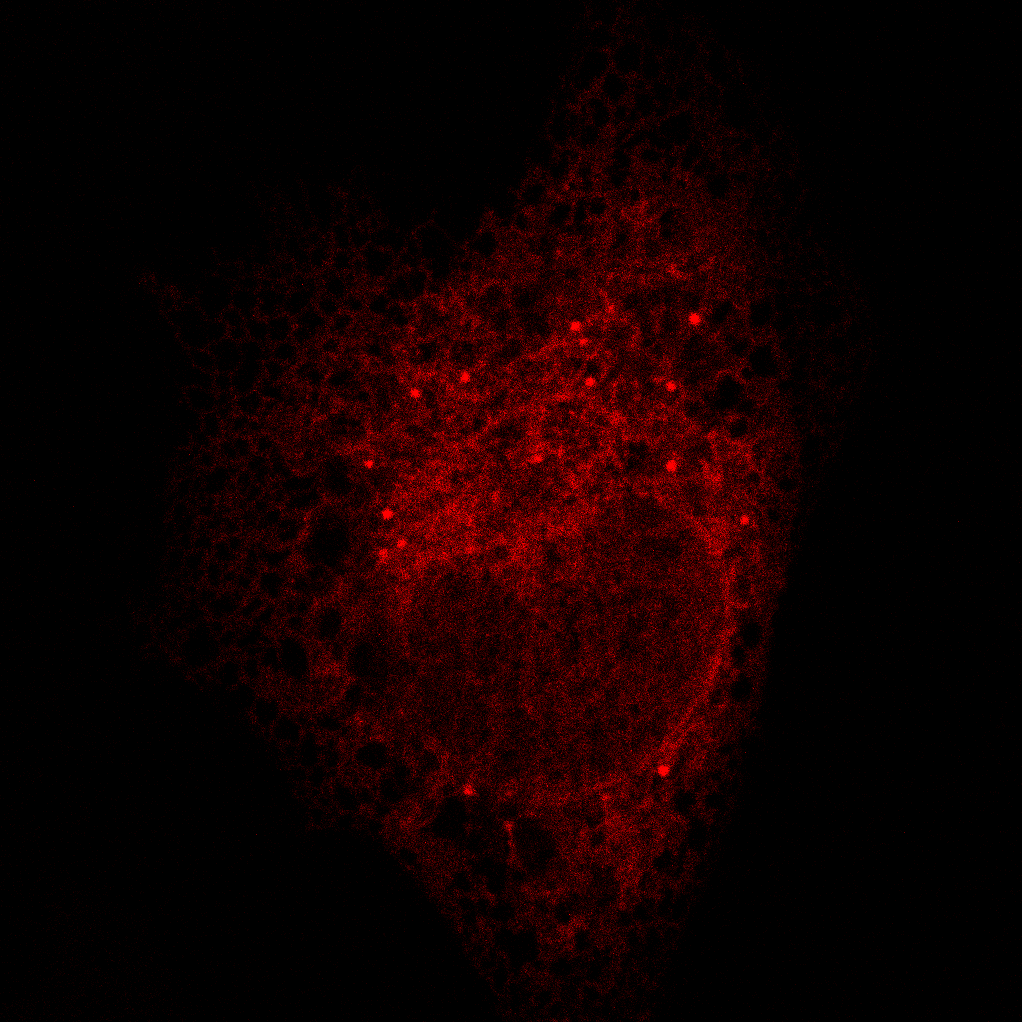

Supplement: Supplementary file 10 — EV and Appendix Figures Source Data [file 44318_2024_232_MOESM10_ESM.zip › EV and Appendix Figures/Figure EV2/Figure EV 2I/Mock/LIRM/RFP.tif]

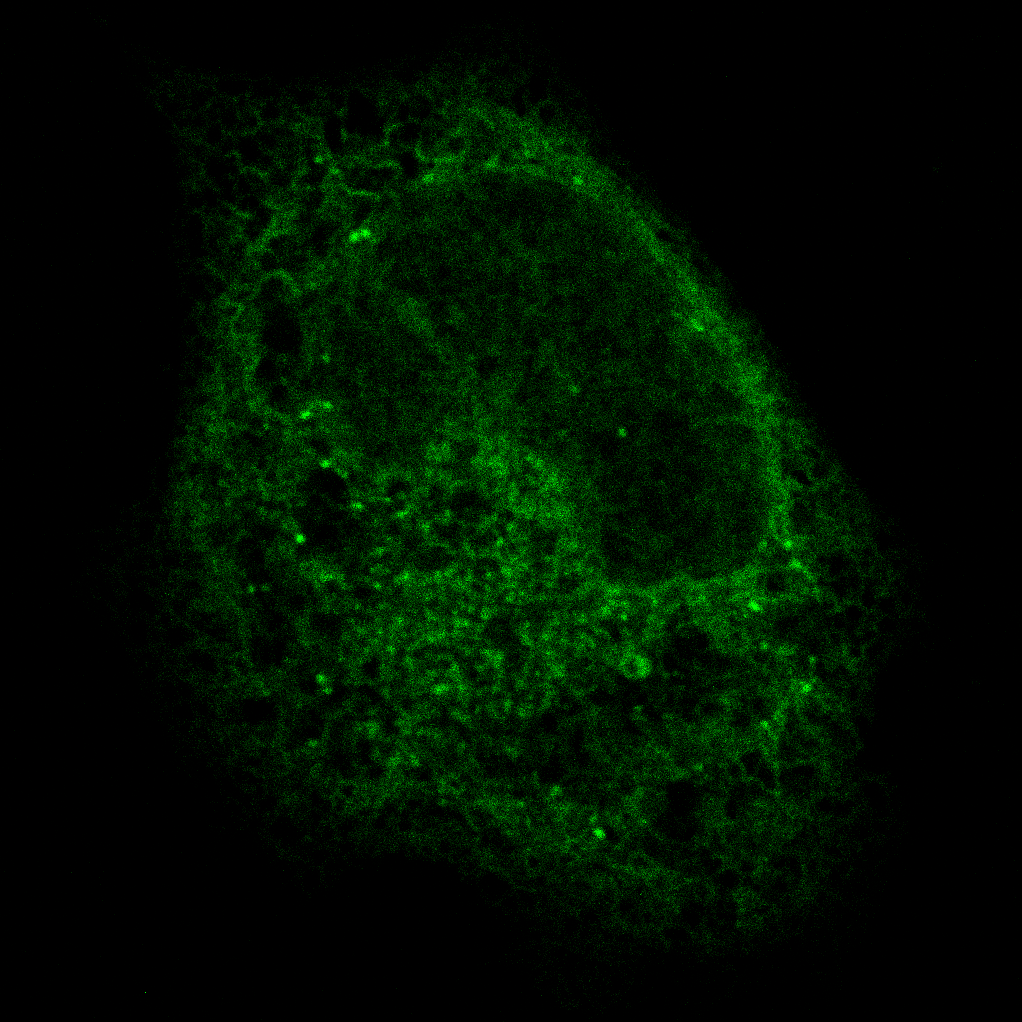

Supplement: Supplementary file 10 — EV and Appendix Figures Source Data [file 44318_2024_232_MOESM10_ESM.zip › EV and Appendix Figures/Figure EV2/Figure EV 2I/Mock/WT/GFP.tif]

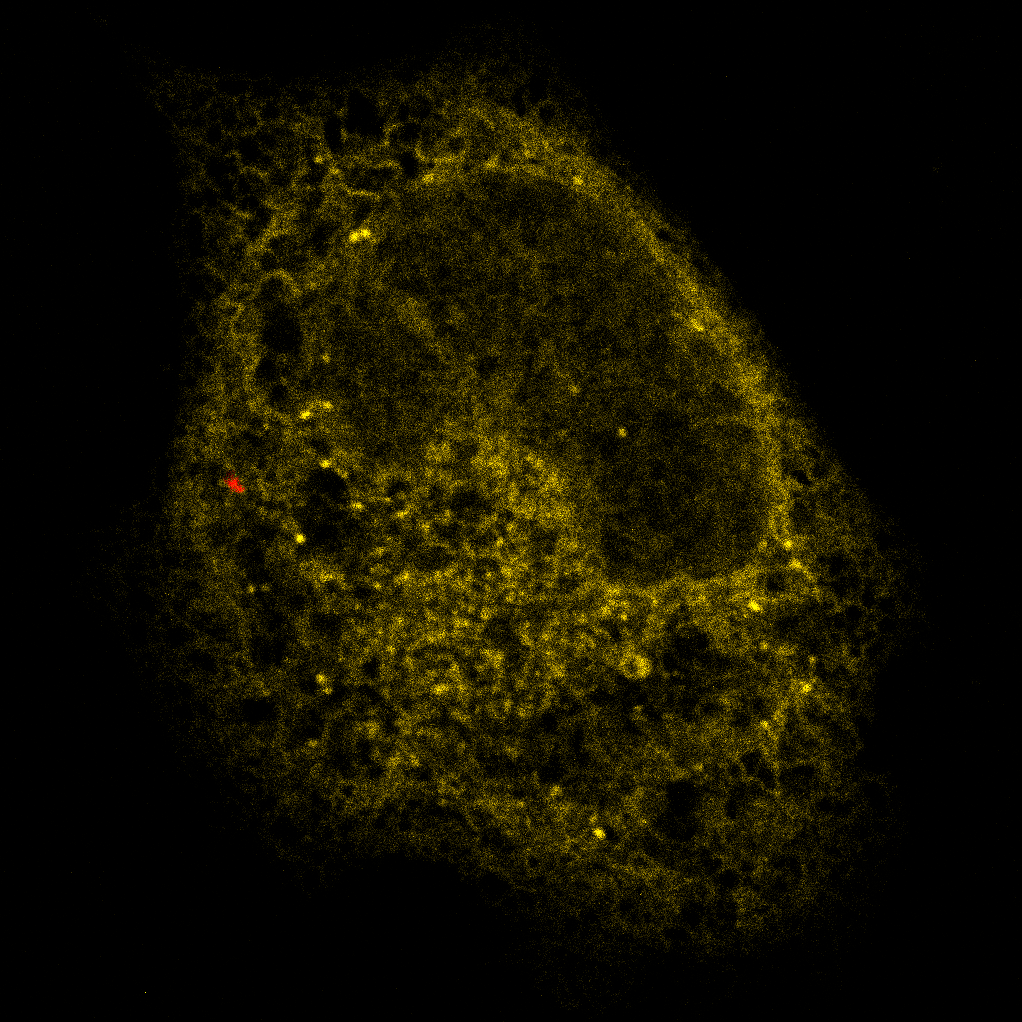

Supplement: Supplementary file 10 — EV and Appendix Figures Source Data [file 44318_2024_232_MOESM10_ESM.zip › EV and Appendix Figures/Figure EV2/Figure EV 2I/Mock/WT/Merge.tif]

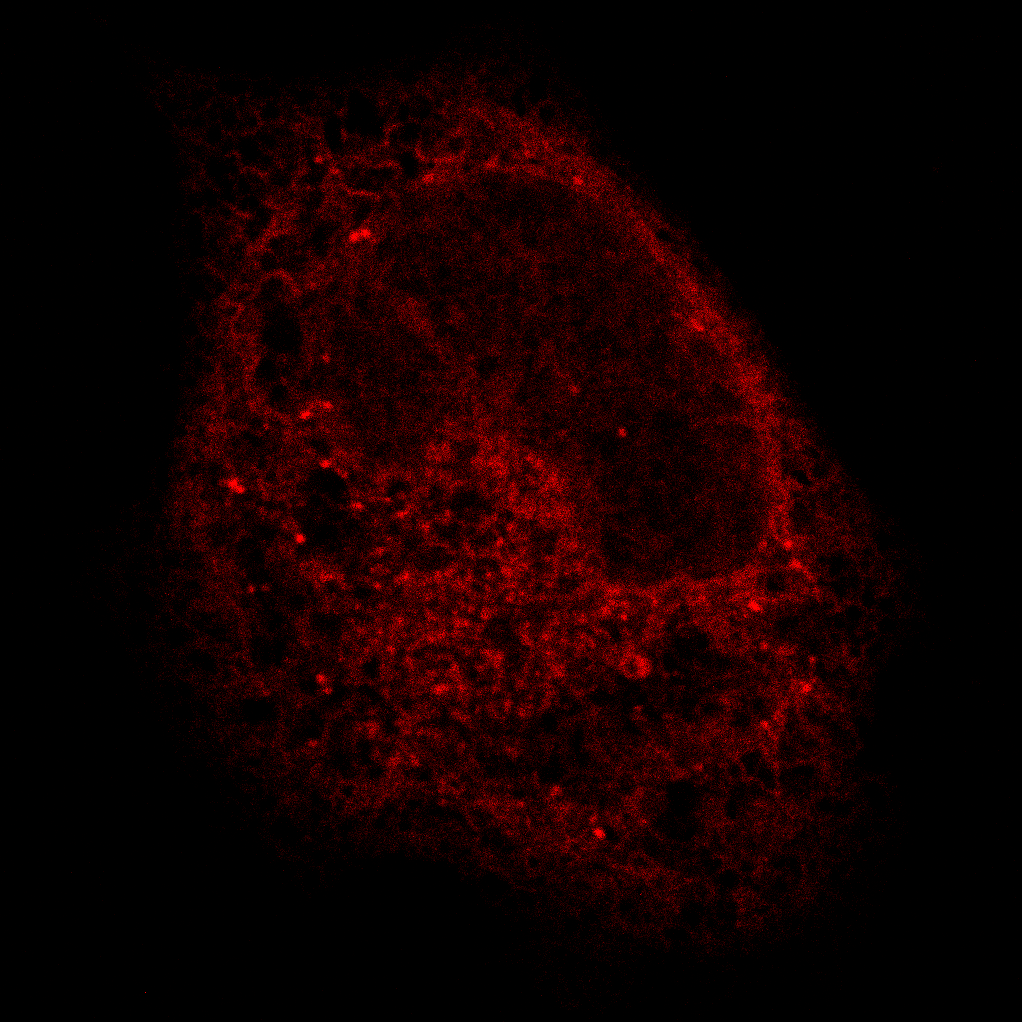

Supplement: Supplementary file 10 — EV and Appendix Figures Source Data [file 44318_2024_232_MOESM10_ESM.zip › EV and Appendix Figures/Figure EV2/Figure EV 2I/Mock/WT/RFP.tif]

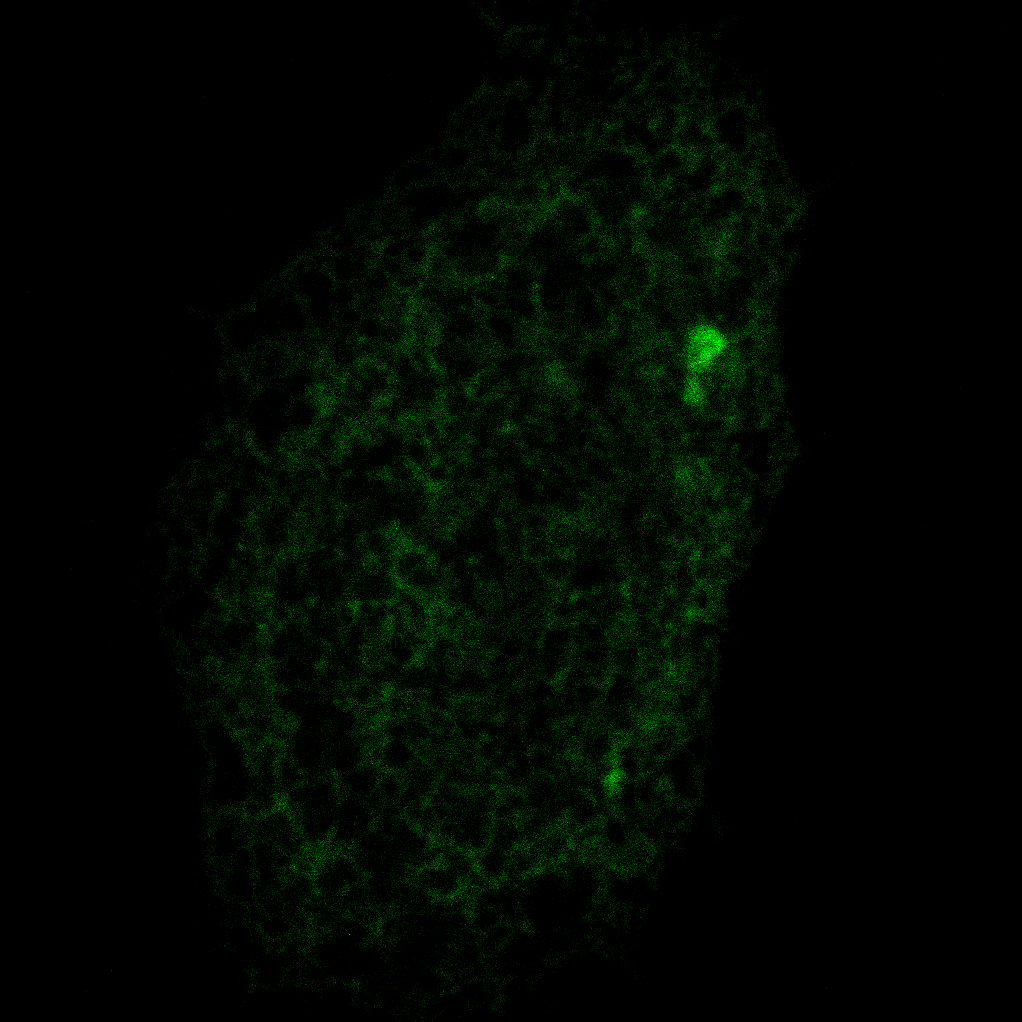

Supplement: Supplementary file 10 — EV and Appendix Figures Source Data [file 44318_2024_232_MOESM10_ESM.zip › EV and Appendix Figures/Figure EV2/Figure EV 2I/TG/-/GFP.tif]

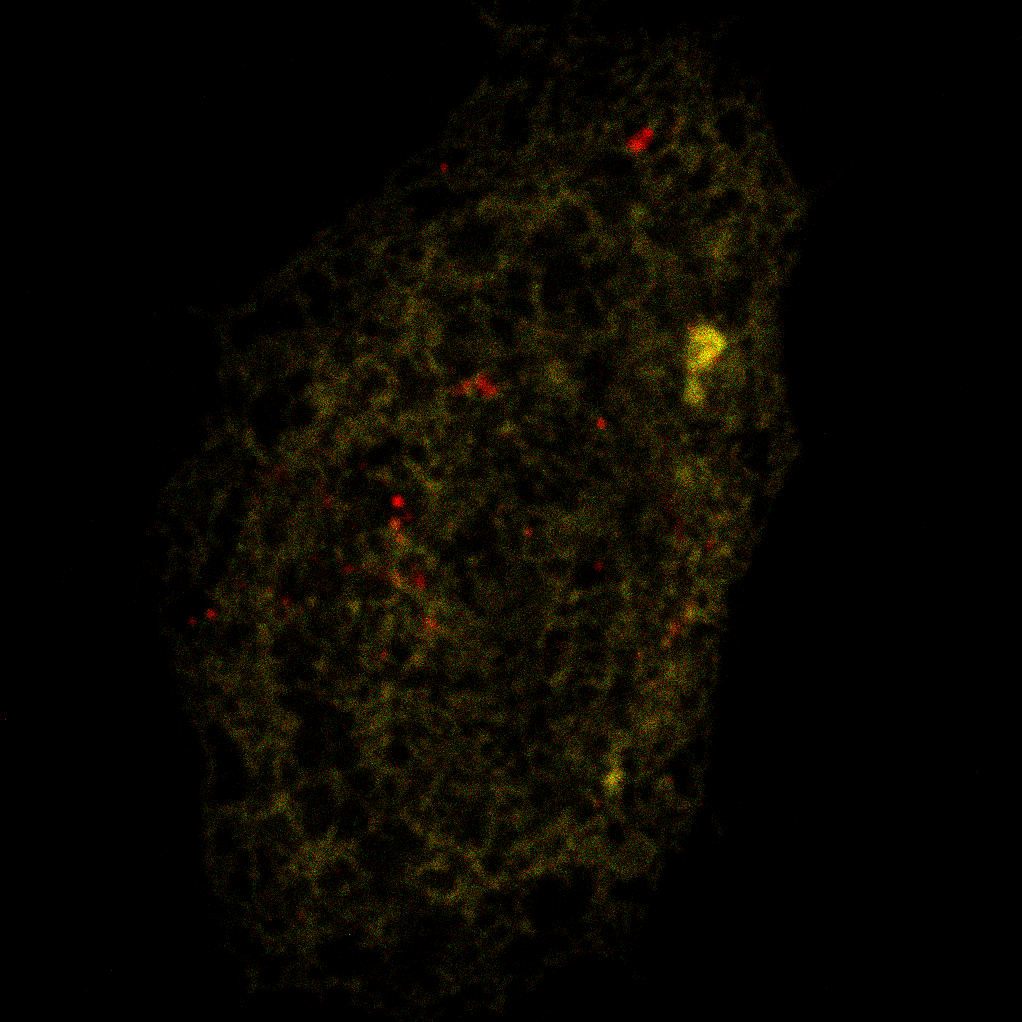

Supplement: Supplementary file 10 — EV and Appendix Figures Source Data [file 44318_2024_232_MOESM10_ESM.zip › EV and Appendix Figures/Figure EV2/Figure EV 2I/TG/-/Merge.tif]

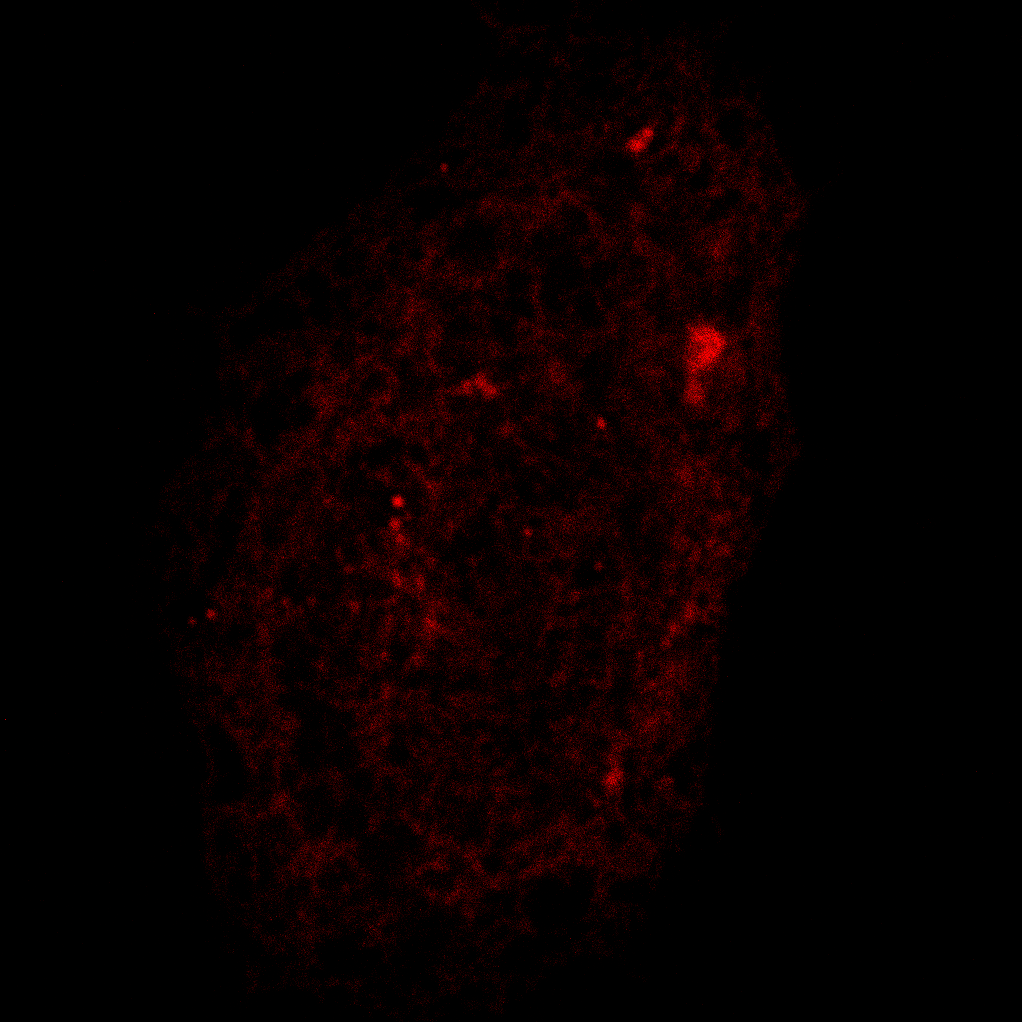

Supplement: Supplementary file 10 — EV and Appendix Figures Source Data [file 44318_2024_232_MOESM10_ESM.zip › EV and Appendix Figures/Figure EV2/Figure EV 2I/TG/-/RFP.tif]

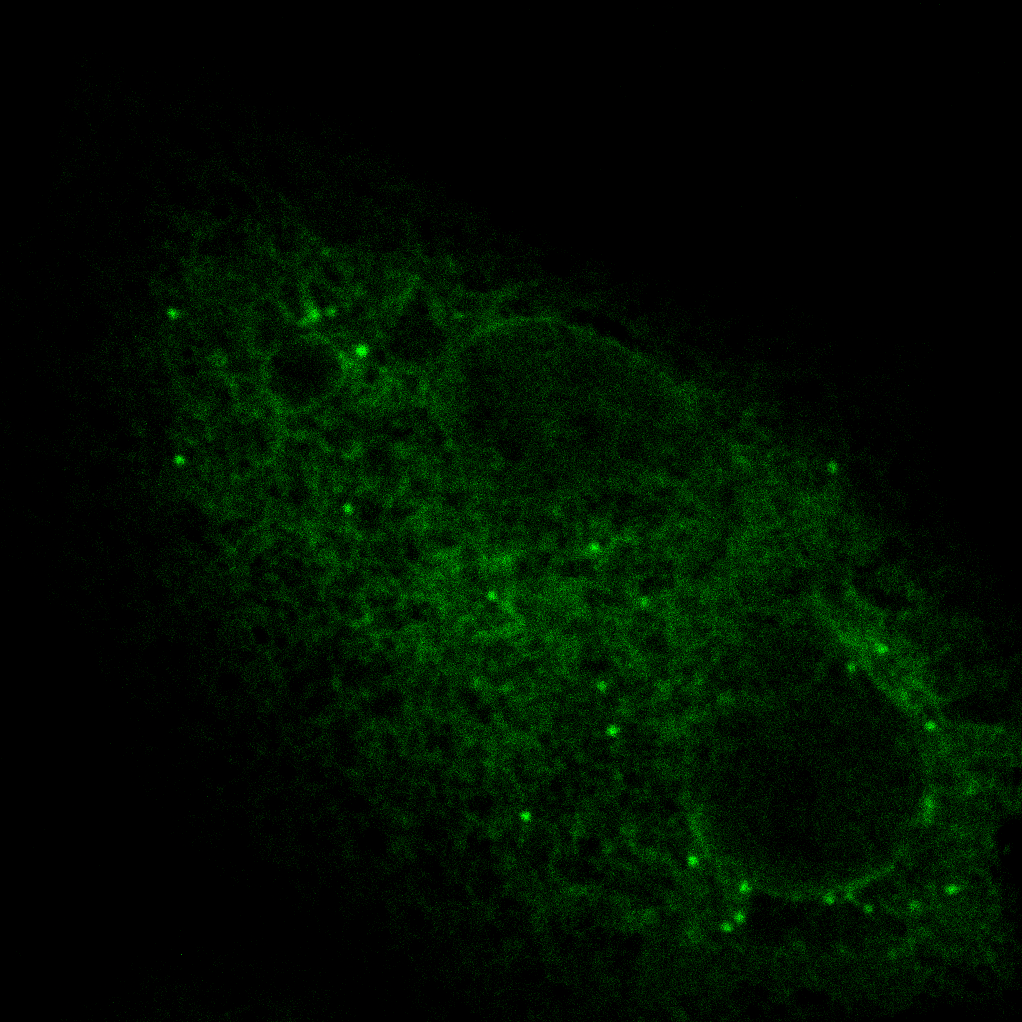

Supplement: Supplementary file 10 — EV and Appendix Figures Source Data [file 44318_2024_232_MOESM10_ESM.zip › EV and Appendix Figures/Figure EV2/Figure EV 2I/TG/LIRM/GFP.tif]

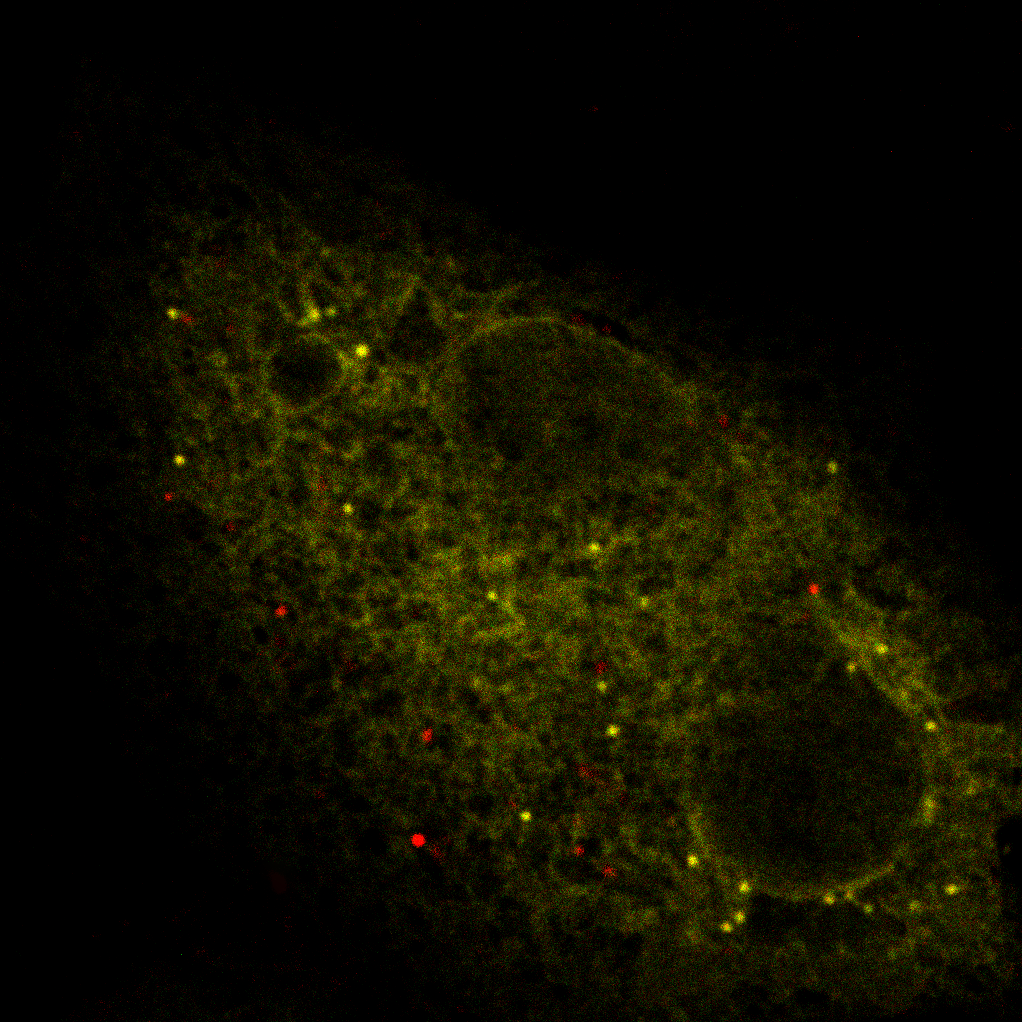

Supplement: Supplementary file 10 — EV and Appendix Figures Source Data [file 44318_2024_232_MOESM10_ESM.zip › EV and Appendix Figures/Figure EV2/Figure EV 2I/TG/LIRM/Merge.tif]

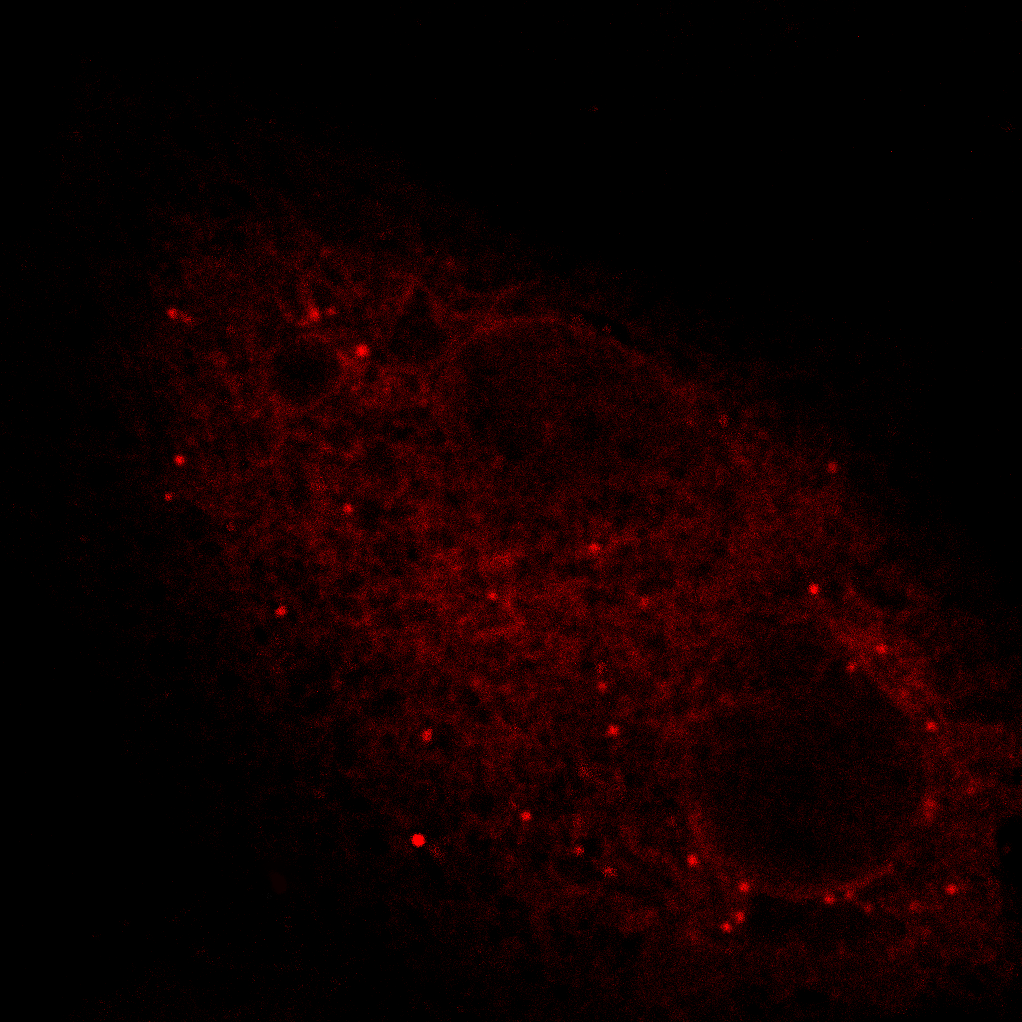

Supplement: Supplementary file 10 — EV and Appendix Figures Source Data [file 44318_2024_232_MOESM10_ESM.zip › EV and Appendix Figures/Figure EV2/Figure EV 2I/TG/LIRM/RFP.tif]

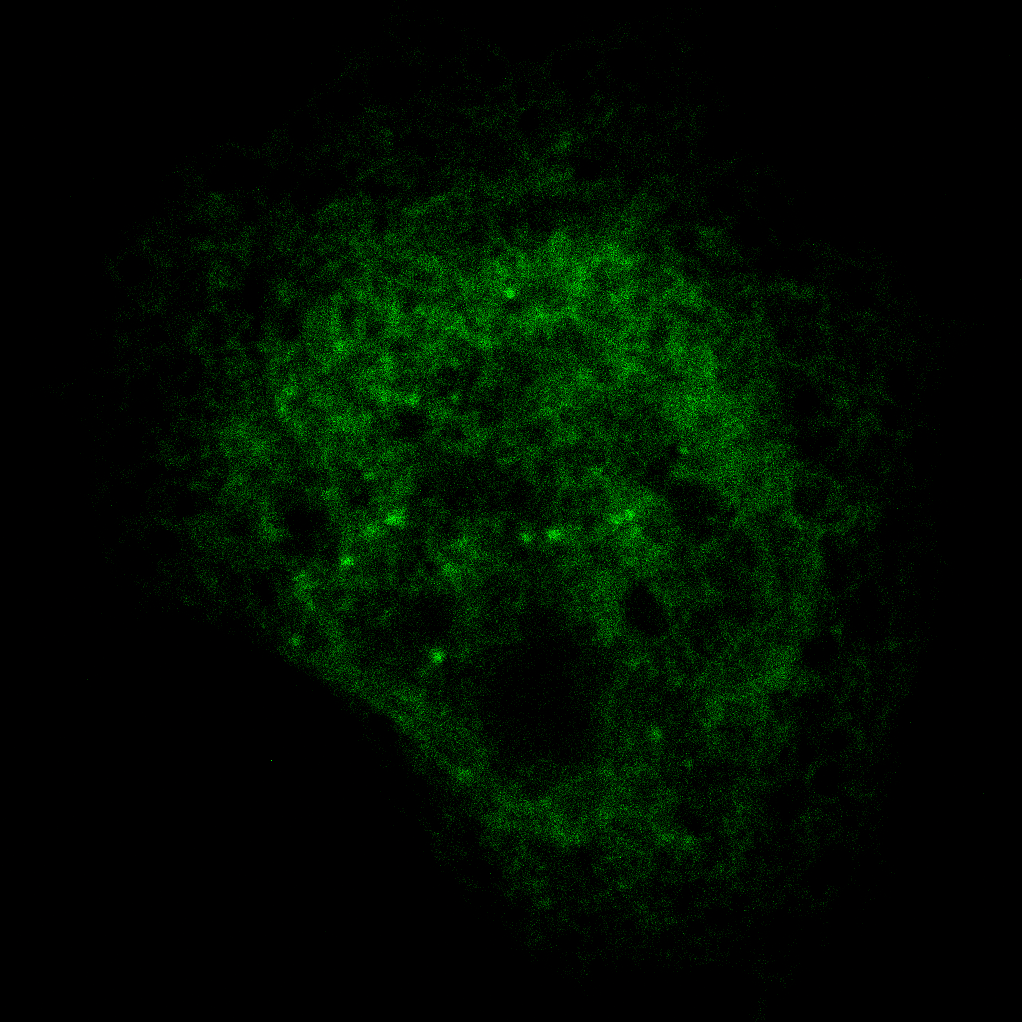

Supplement: Supplementary file 10 — EV and Appendix Figures Source Data [file 44318_2024_232_MOESM10_ESM.zip › EV and Appendix Figures/Figure EV2/Figure EV 2I/TG/WT/GFP.tif]

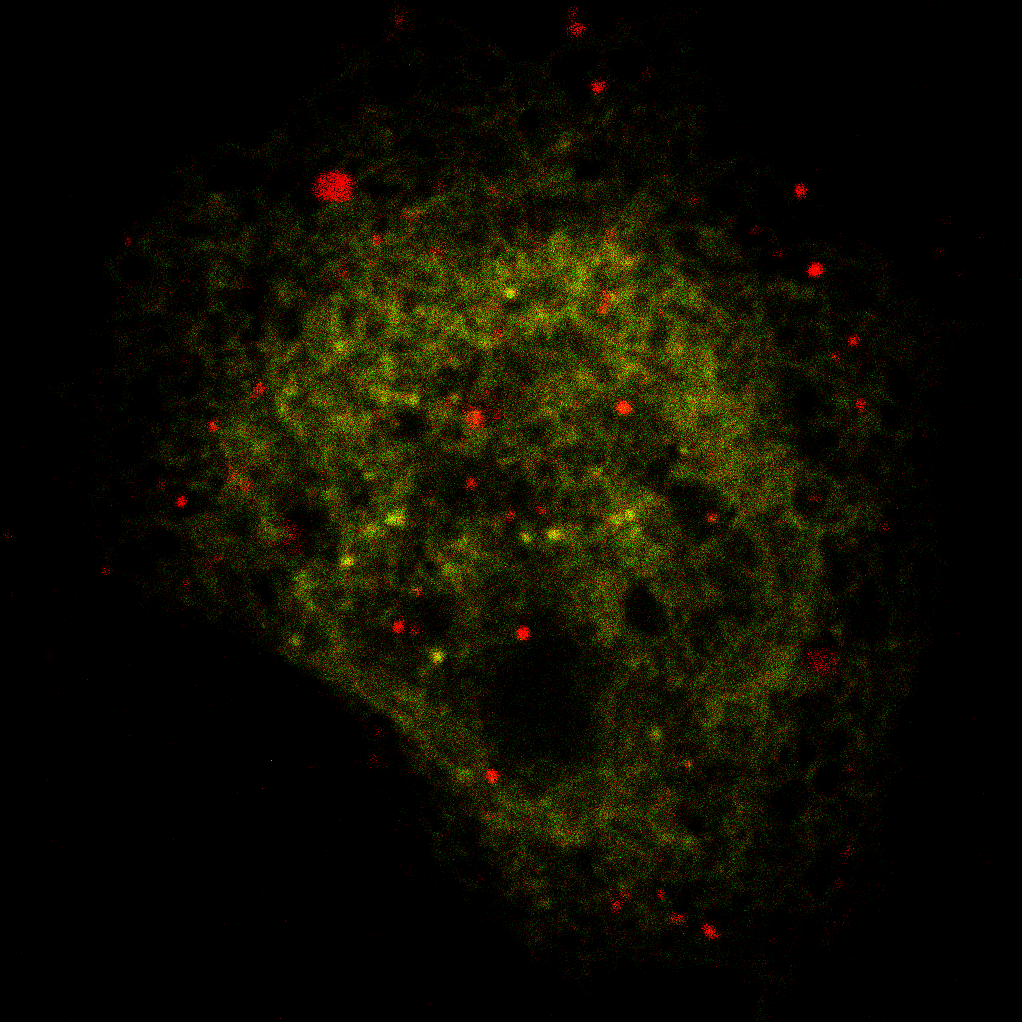

Supplement: Supplementary file 10 — EV and Appendix Figures Source Data [file 44318_2024_232_MOESM10_ESM.zip › EV and Appendix Figures/Figure EV2/Figure EV 2I/TG/WT/Merge.tif]

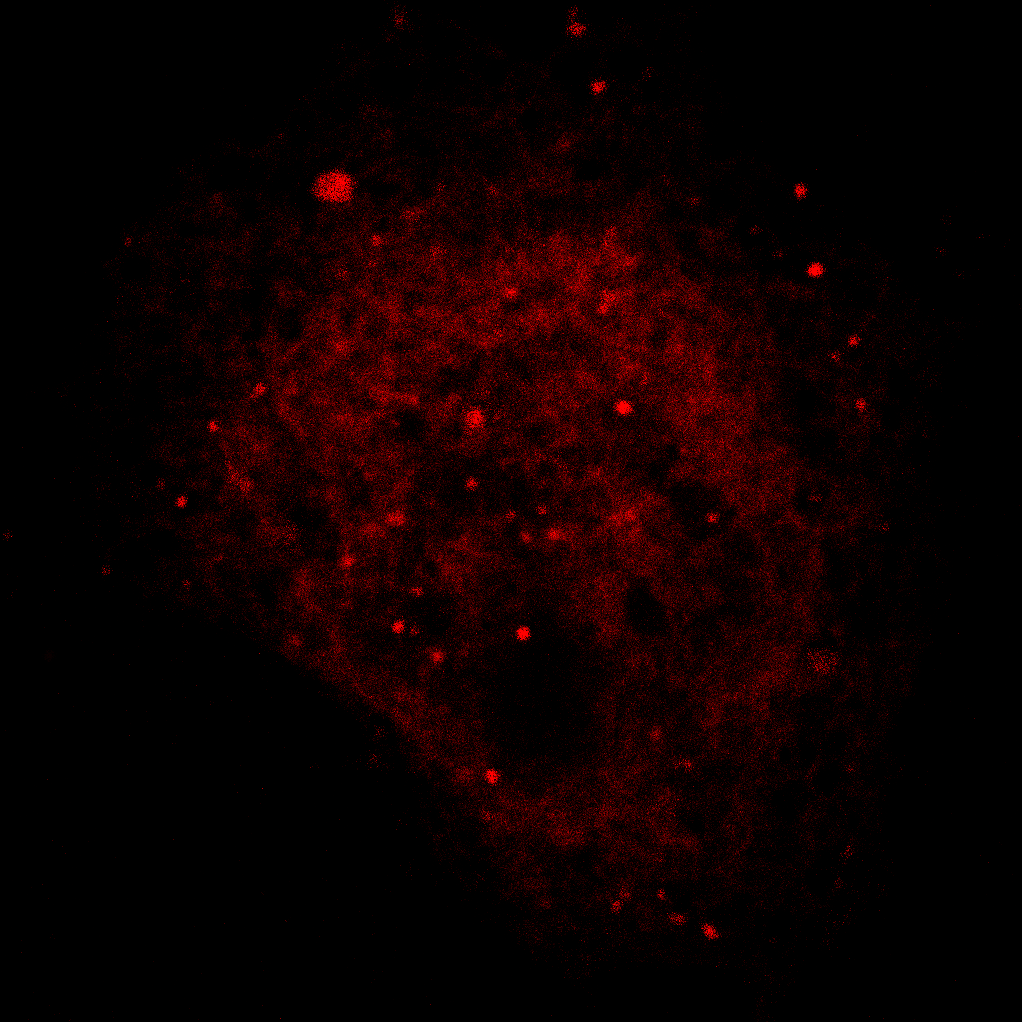

Supplement: Supplementary file 10 — EV and Appendix Figures Source Data [file 44318_2024_232_MOESM10_ESM.zip › EV and Appendix Figures/Figure EV2/Figure EV 2I/TG/WT/RFP.tif]

Source data: Figure EV 3A.

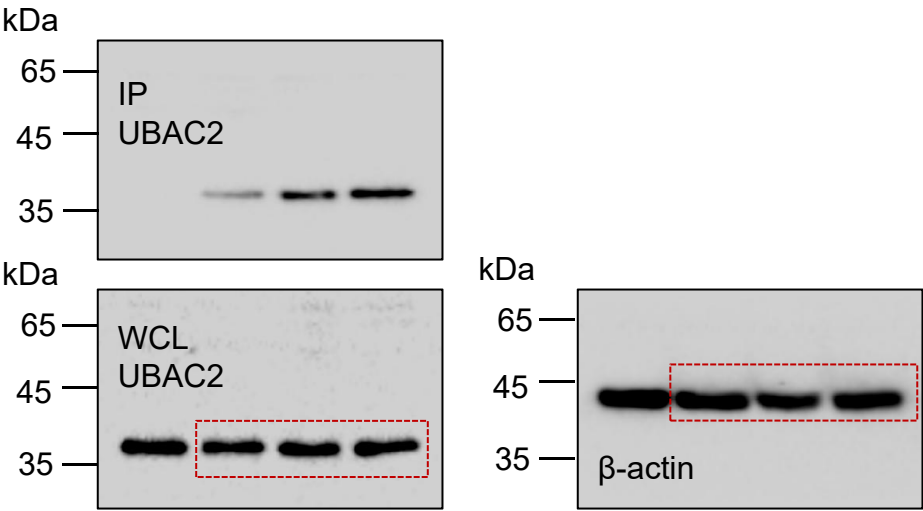

Supplement: Supplementary file 10 — EV and Appendix Figures Source Data [file 44318_2024_232_MOESM10_ESM.zip › EV and Appendix Figures/Figure EV3/Figure EV 3A.pdf]

Source data: Figure EV 3B.

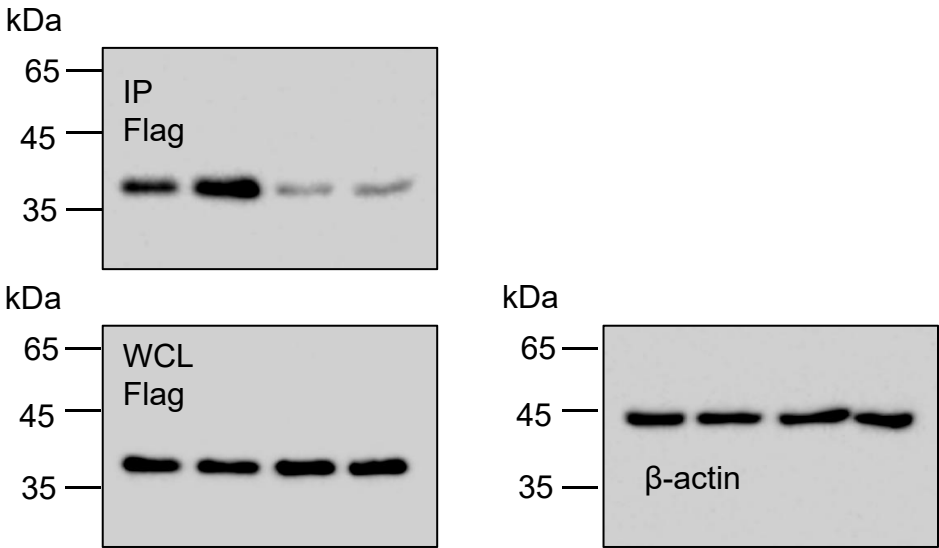

Supplement: Supplementary file 10 — EV and Appendix Figures Source Data [file 44318_2024_232_MOESM10_ESM.zip › EV and Appendix Figures/Figure EV3/Figure EV 3B.pdf]

Source data: Figure EV 3C.

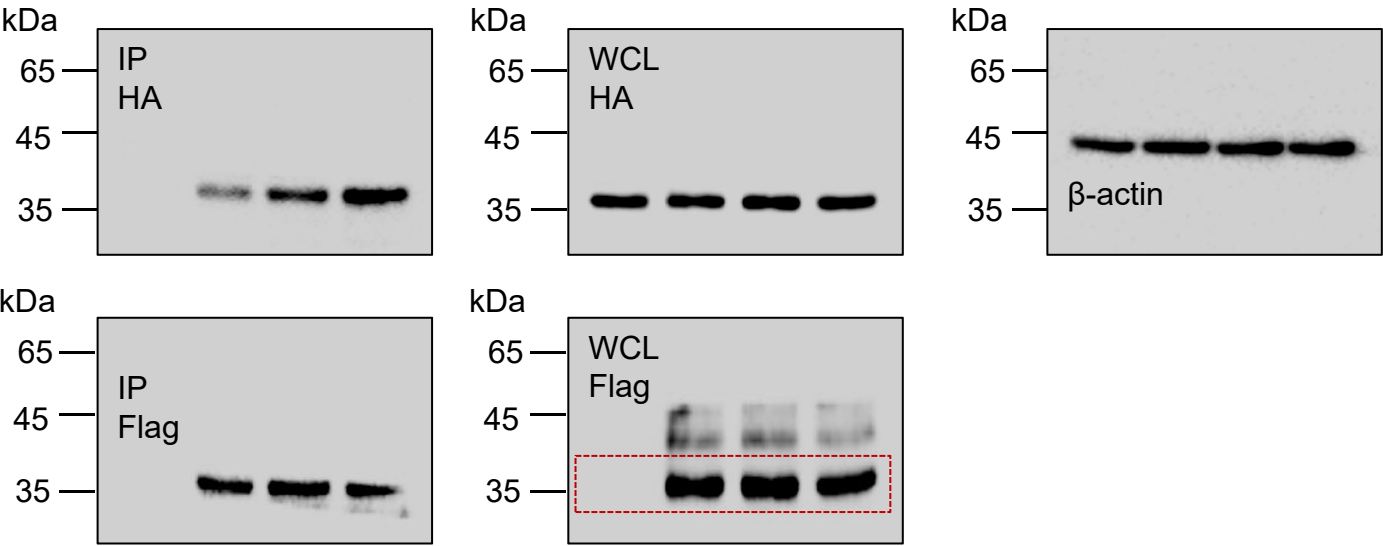

Supplement: Supplementary file 10 — EV and Appendix Figures Source Data [file 44318_2024_232_MOESM10_ESM.zip › EV and Appendix Figures/Figure EV3/Figure EV 3C.pdf]

Source data: Figure EV 3D.

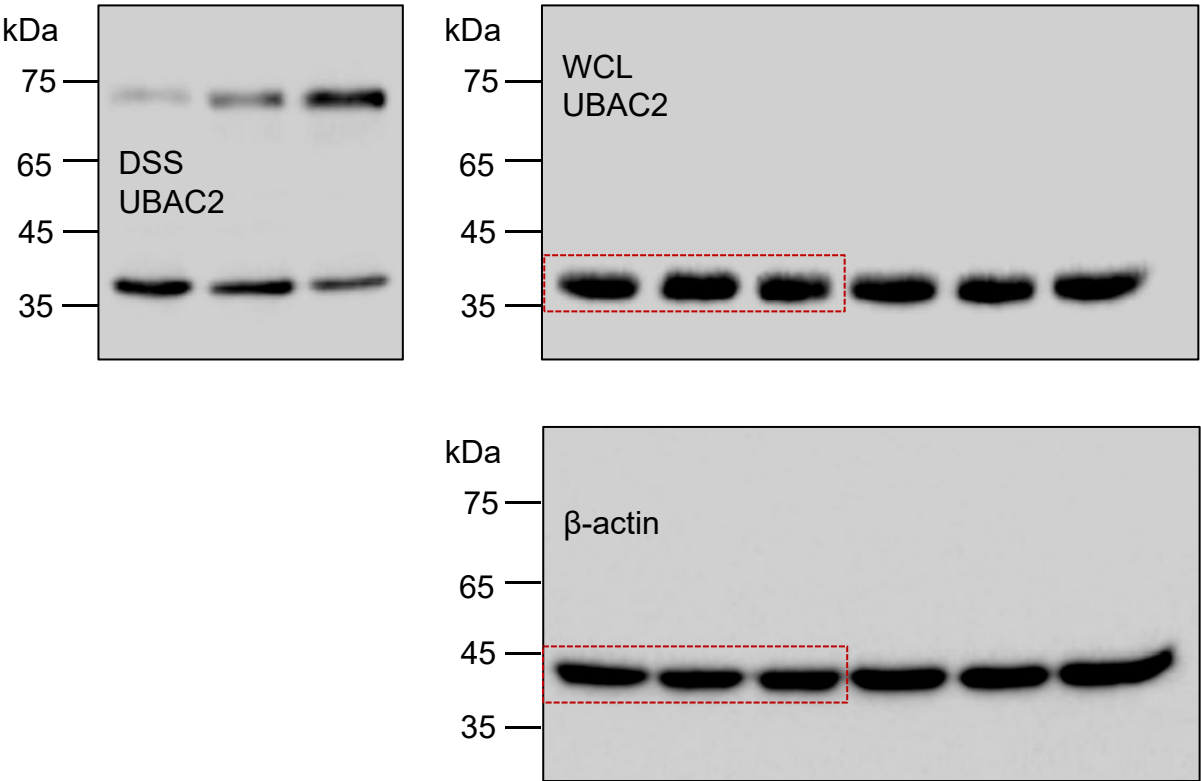

Supplement: Supplementary file 10 — EV and Appendix Figures Source Data [file 44318_2024_232_MOESM10_ESM.zip › EV and Appendix Figures/Figure EV3/Figure EV 3D.pdf]

Source data: Figure EV 3E.

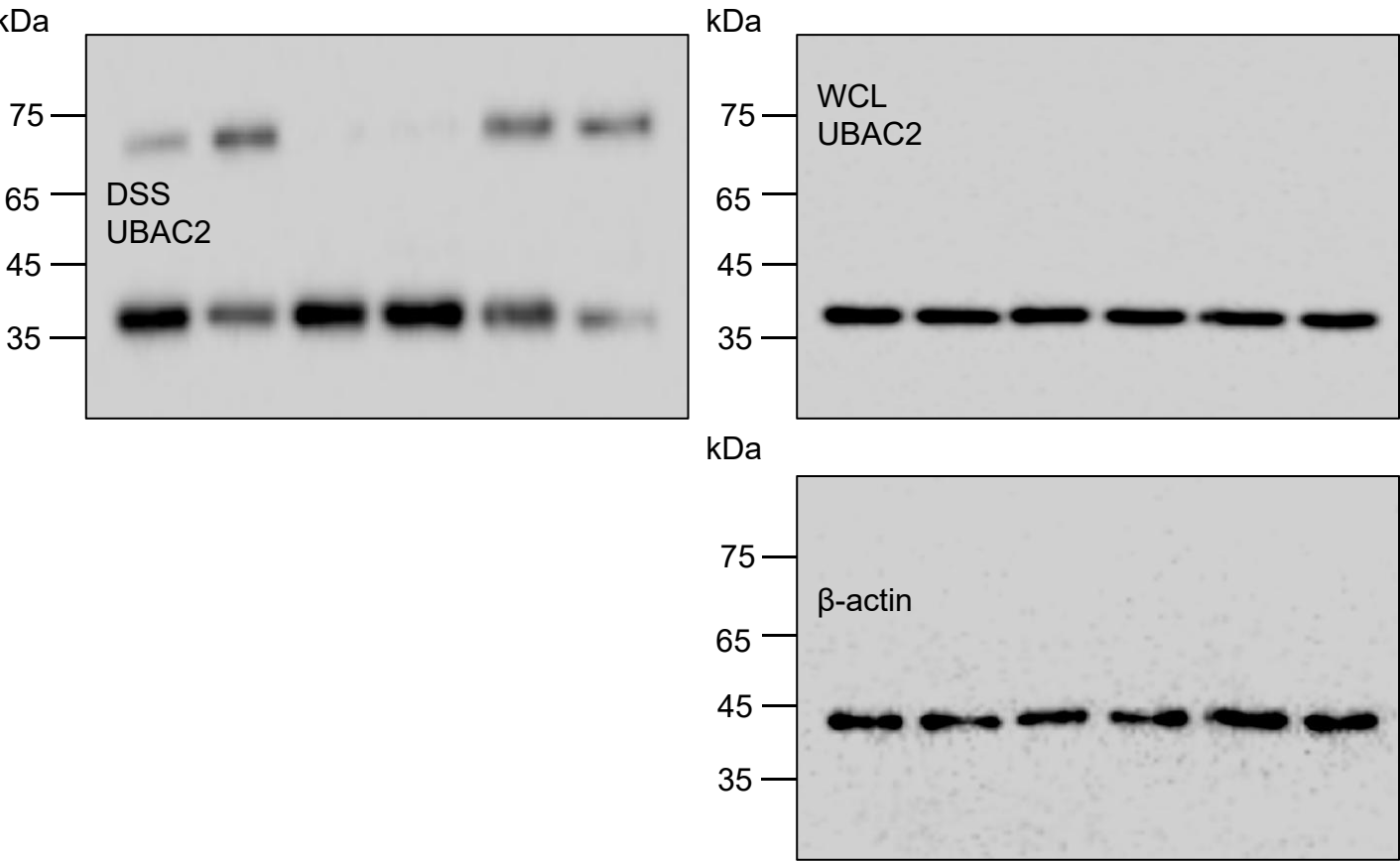

Supplement: Supplementary file 10 — EV and Appendix Figures Source Data [file 44318_2024_232_MOESM10_ESM.zip › EV and Appendix Figures/Figure EV3/Figure EV 3E.pdf]

Source data: Figure EV 3G.

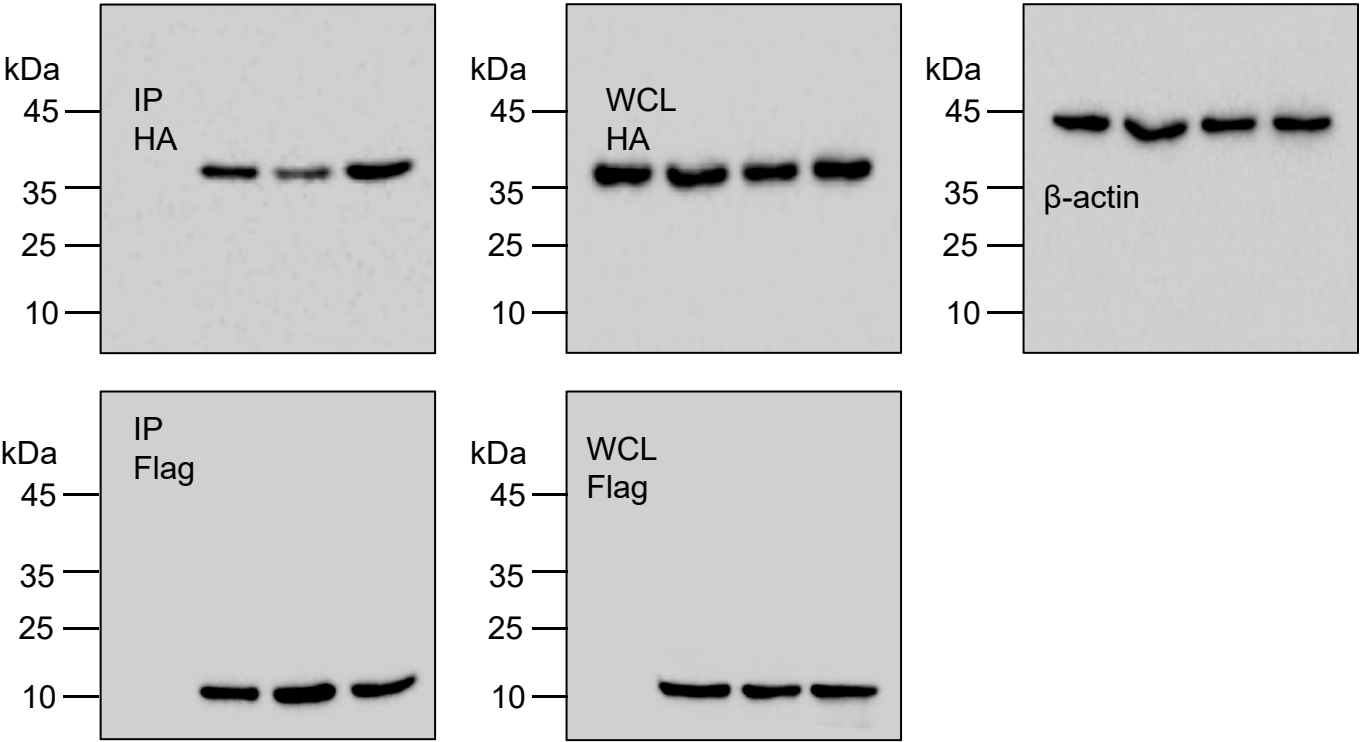

Supplement: Supplementary file 10 — EV and Appendix Figures Source Data [file 44318_2024_232_MOESM10_ESM.zip › EV and Appendix Figures/Figure EV3/Figure EV 3G.pdf]

Source data: Figure EV 4A.

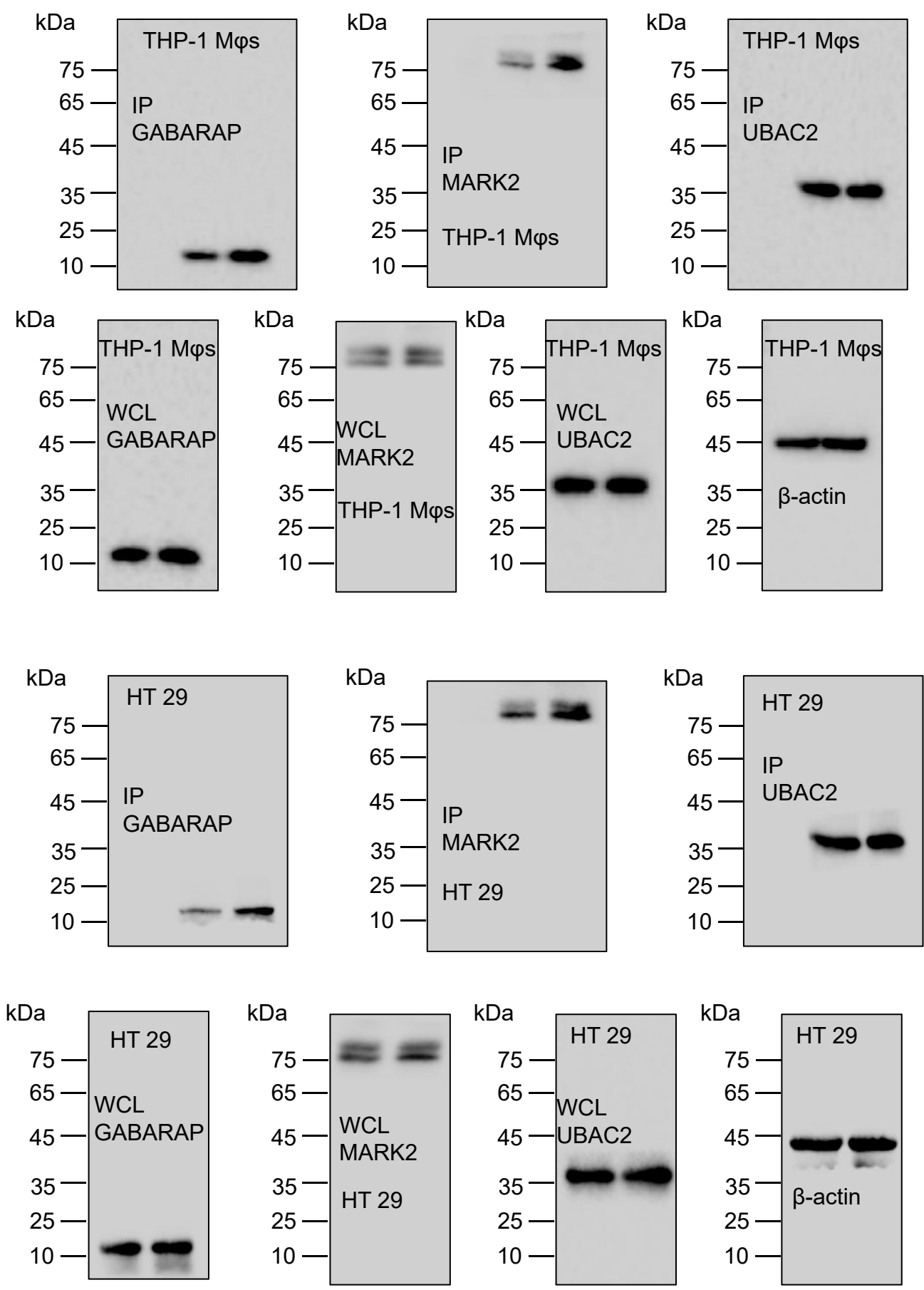

Supplement: Supplementary file 10 — EV and Appendix Figures Source Data [file 44318_2024_232_MOESM10_ESM.zip › EV and Appendix Figures/Figure EV4/Figure EV 4A.pdf]

Source data: Figure EV 4B.

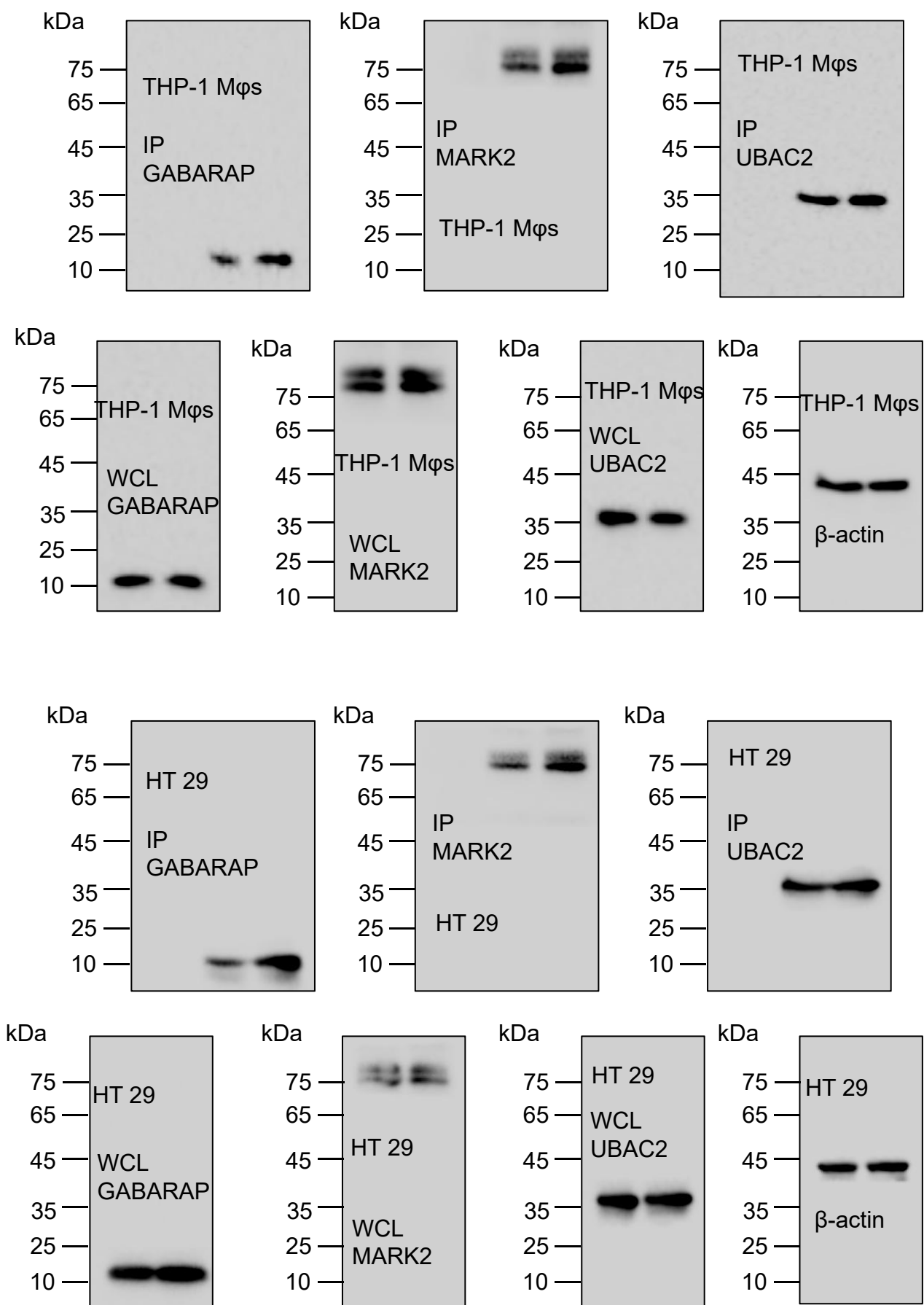

Supplement: Supplementary file 10 — EV and Appendix Figures Source Data [file 44318_2024_232_MOESM10_ESM.zip › EV and Appendix Figures/Figure EV4/Figure EV 4B.pdf]

Source data: Figure EV 4C.

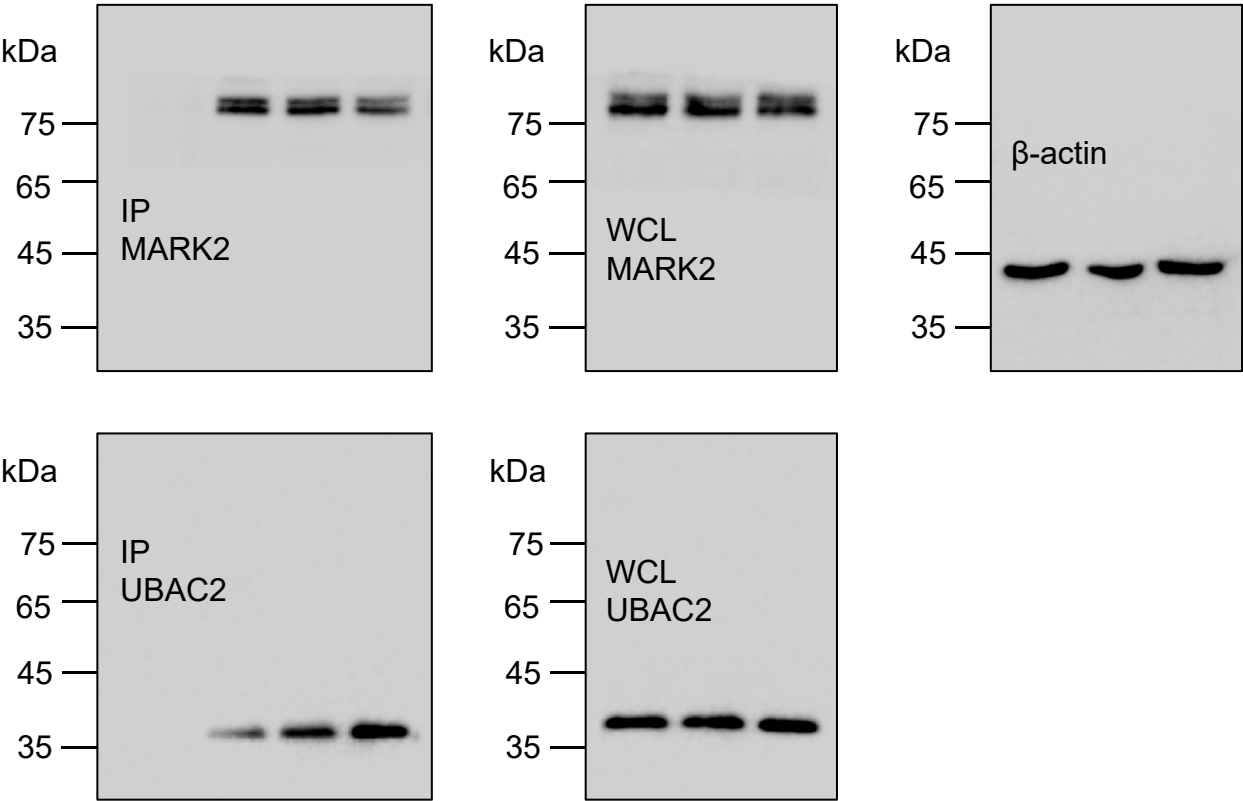

Supplement: Supplementary file 10 — EV and Appendix Figures Source Data [file 44318_2024_232_MOESM10_ESM.zip › EV and Appendix Figures/Figure EV4/Figure EV 4C.pdf]

Source data: Figure EV 4D.

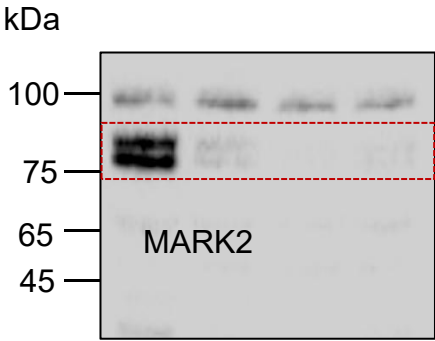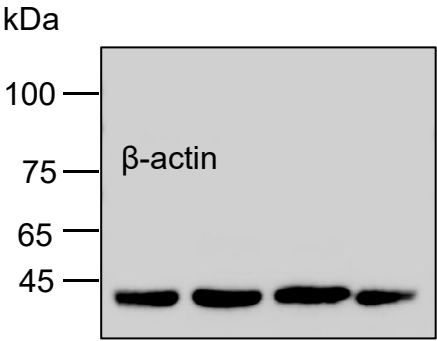

Supplement: Supplementary file 10 — EV and Appendix Figures Source Data [file 44318_2024_232_MOESM10_ESM.zip › EV and Appendix Figures/Figure EV4/Figure EV 4D.pdf]

Source data: Figure EV 4E.

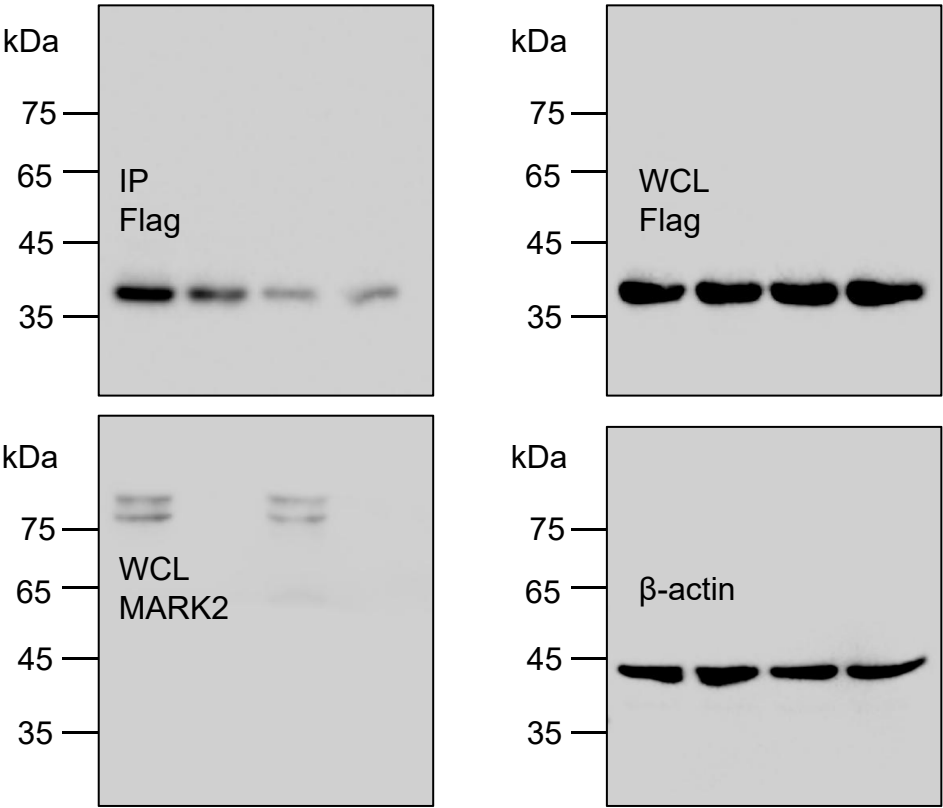

Supplement: Supplementary file 10 — EV and Appendix Figures Source Data [file 44318_2024_232_MOESM10_ESM.zip › EV and Appendix Figures/Figure EV4/Figure EV 4E.pdf]

Source data: Figure EV 4F.

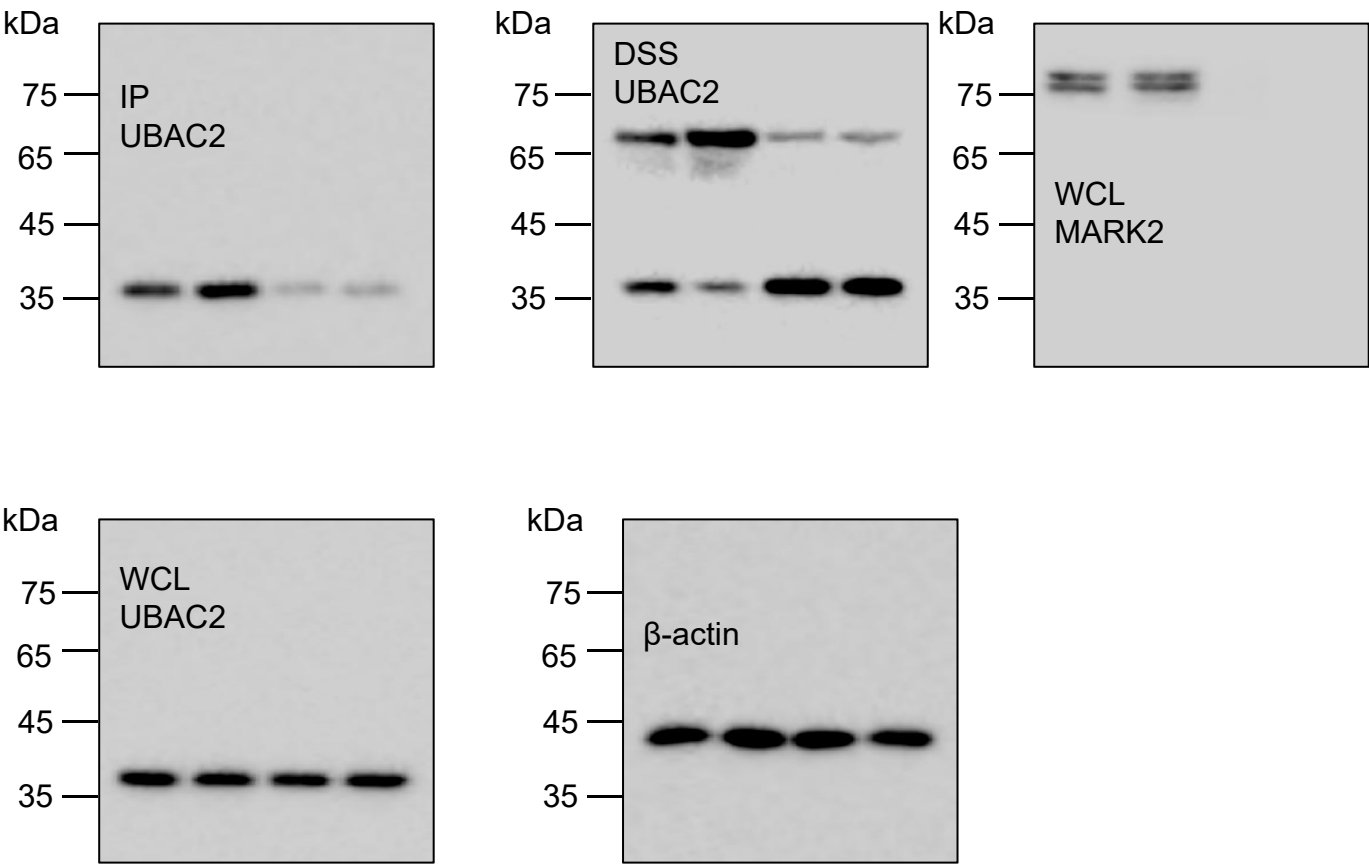

Supplement: Supplementary file 10 — EV and Appendix Figures Source Data [file 44318_2024_232_MOESM10_ESM.zip › EV and Appendix Figures/Figure EV4/Figure EV 4F.pdf]

Source data: Figure EV 4G.

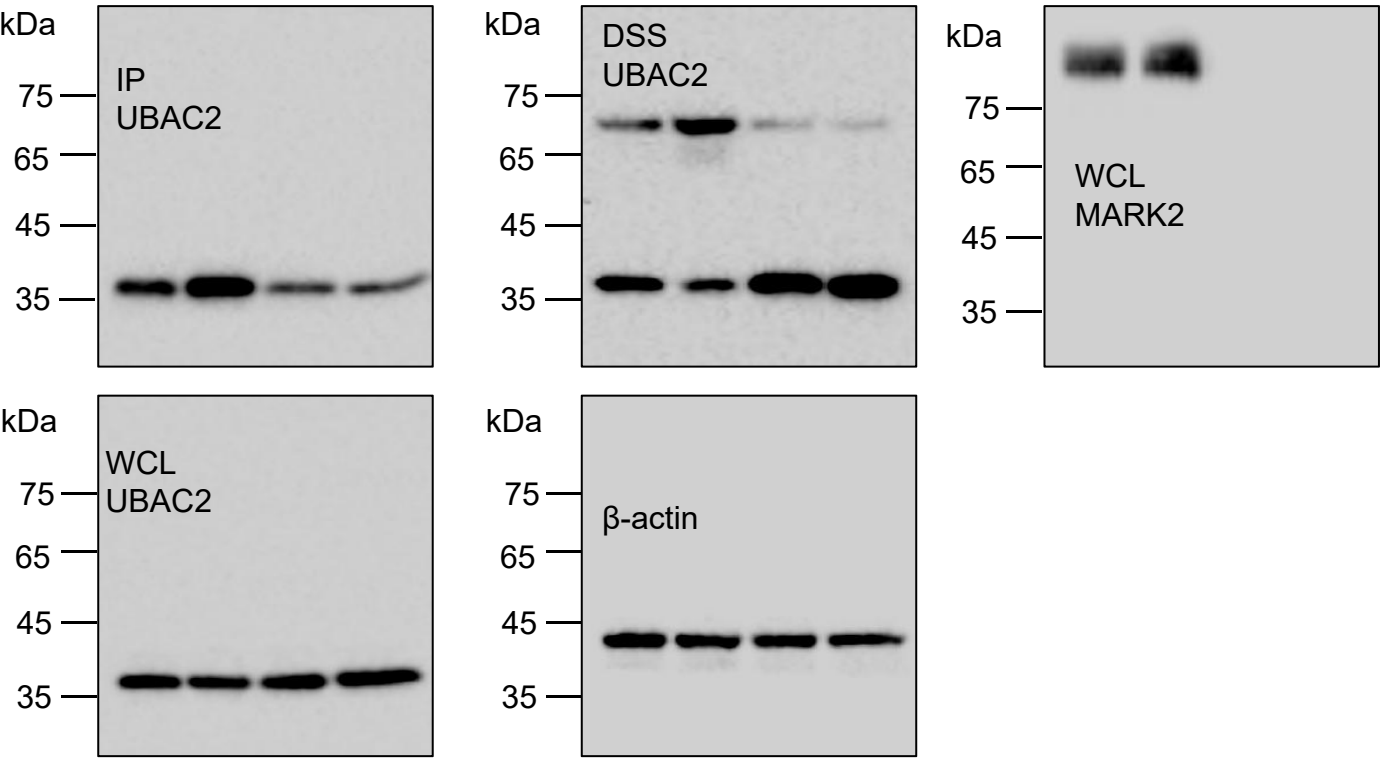

Supplement: Supplementary file 10 — EV and Appendix Figures Source Data [file 44318_2024_232_MOESM10_ESM.zip › EV and Appendix Figures/Figure EV4/Figure EV 4G.pdf]

Source data: Figure EV 4H.

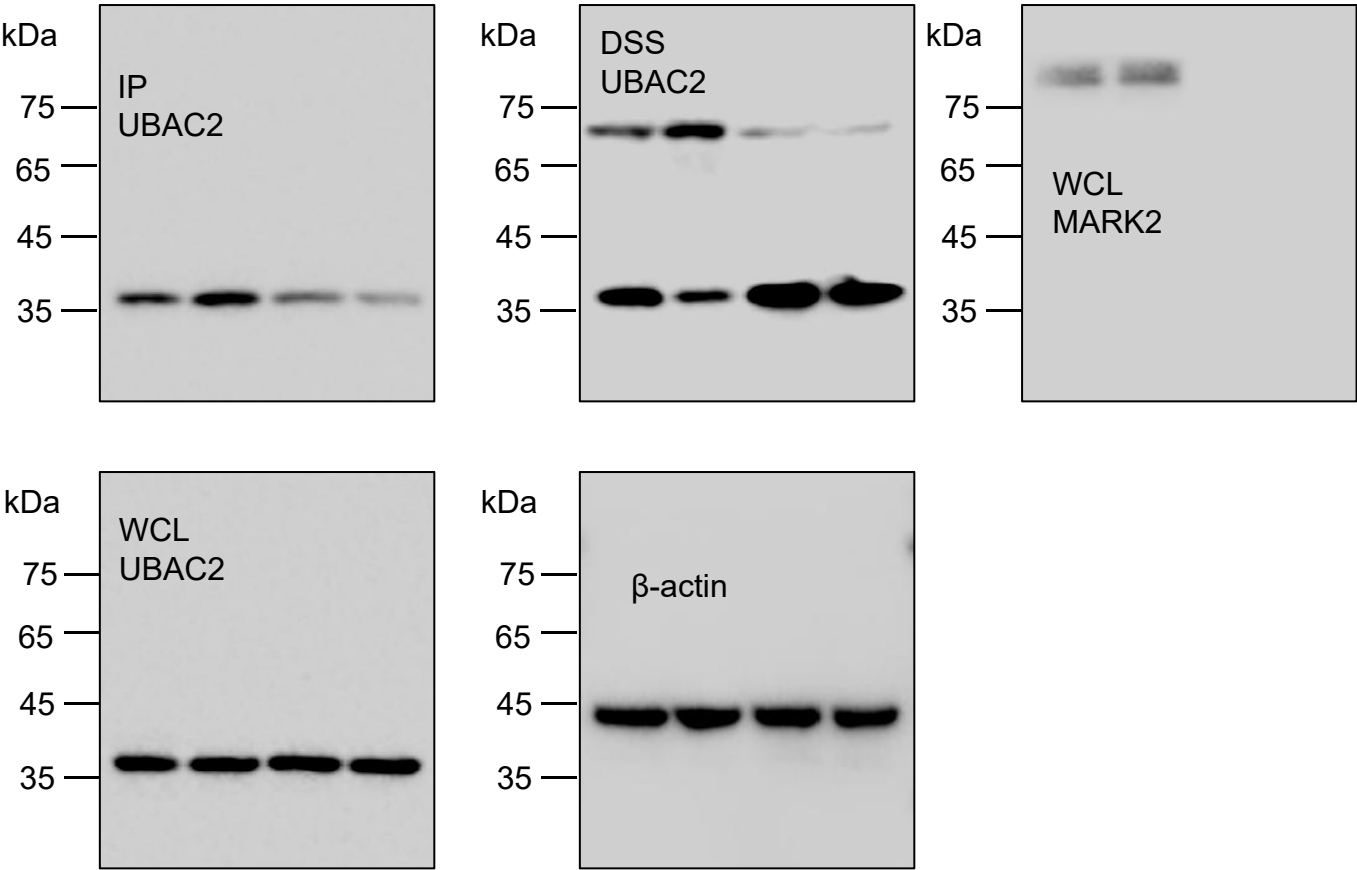

Supplement: Supplementary file 10 — EV and Appendix Figures Source Data [file 44318_2024_232_MOESM10_ESM.zip › EV and Appendix Figures/Figure EV4/Figure EV 4H.pdf]

Source data: Figure EV 4I.

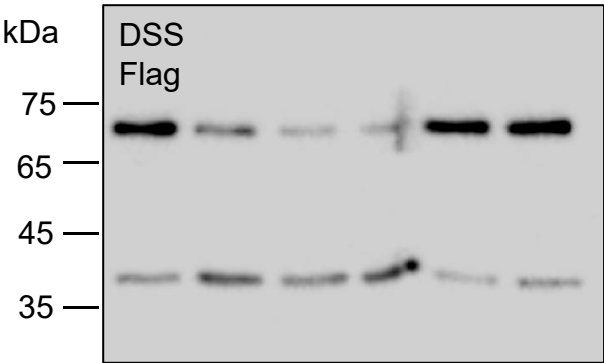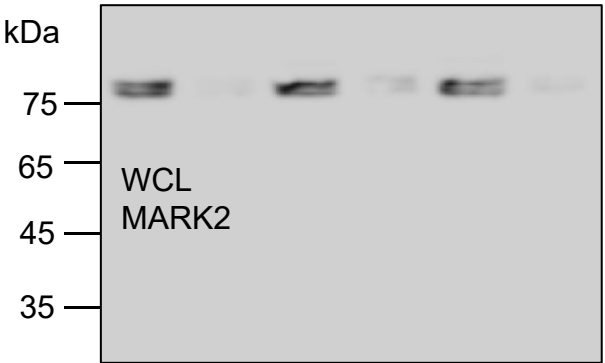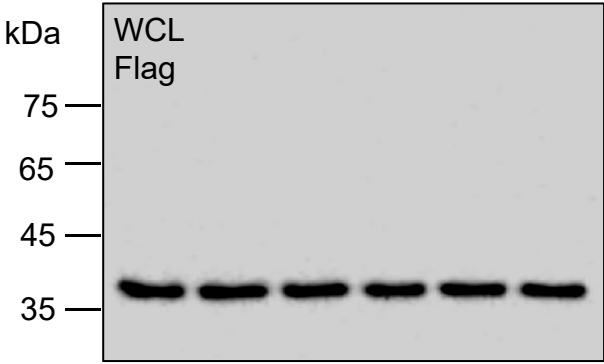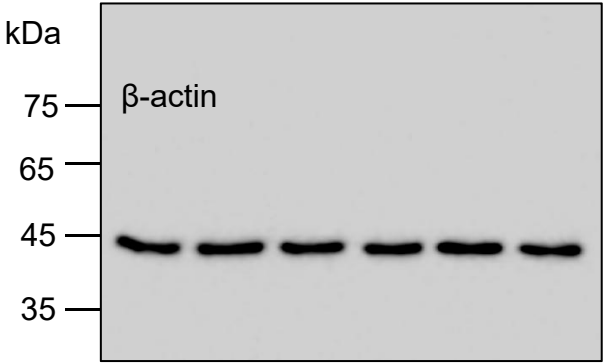

Supplement: Supplementary file 10 — EV and Appendix Figures Source Data [file 44318_2024_232_MOESM10_ESM.zip › EV and Appendix Figures/Figure EV4/Figure EV 4I.pdf]

Source data: Figure EV 4K.

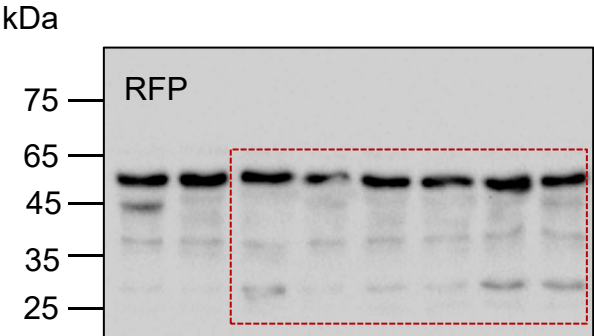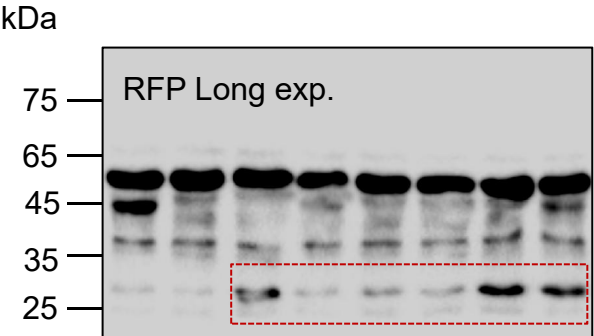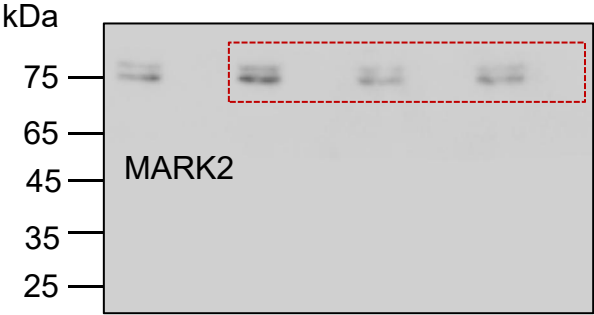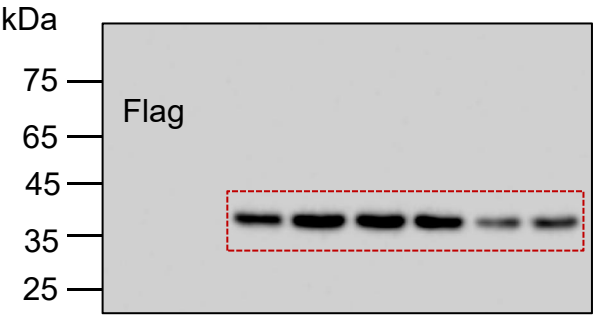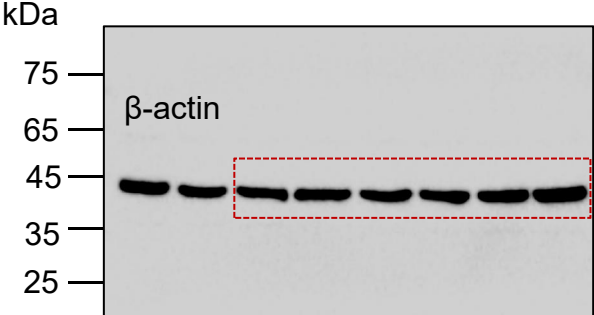

Supplement: Supplementary file 10 — EV and Appendix Figures Source Data [file 44318_2024_232_MOESM10_ESM.zip › EV and Appendix Figures/Figure EV4/Figure EV 4K.pdf]

Source data: Figure EV 4M.

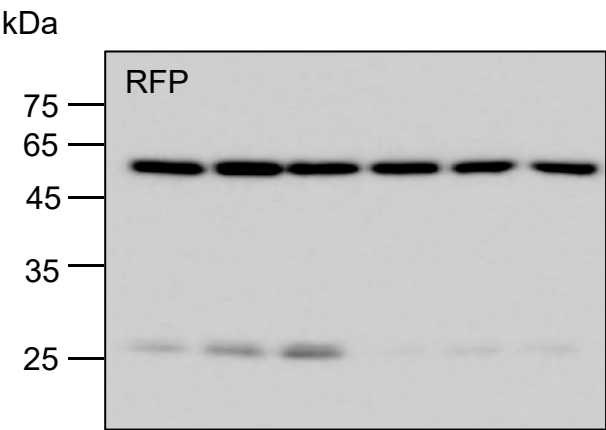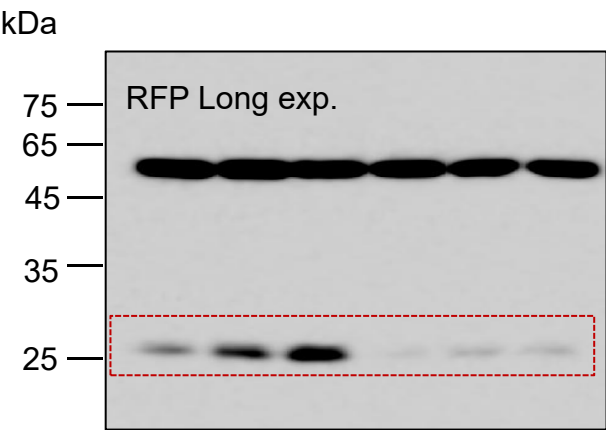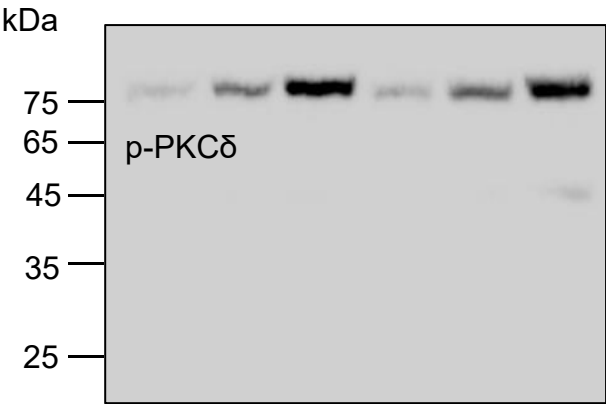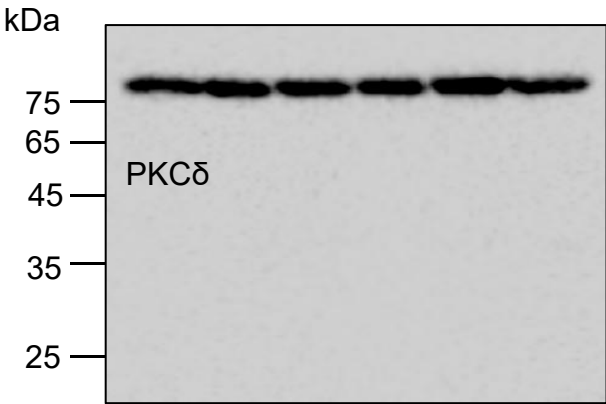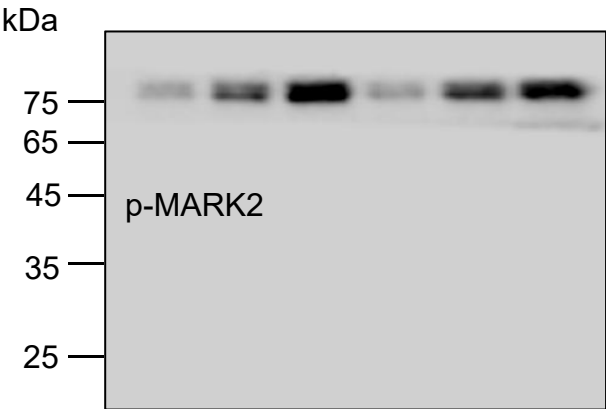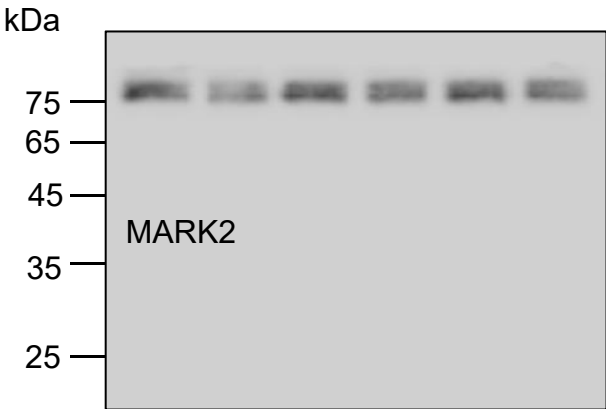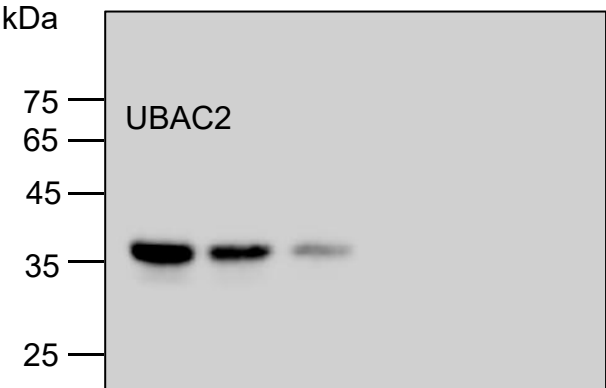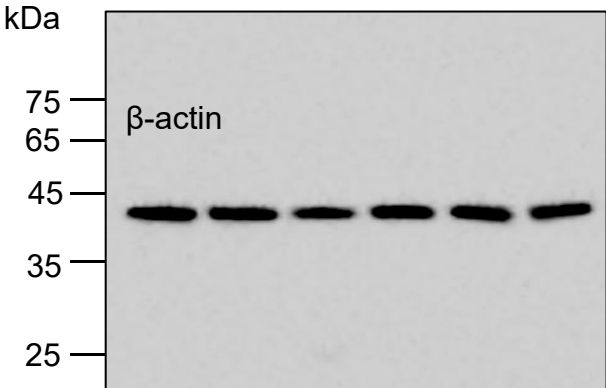

Supplement: Supplementary file 10 — EV and Appendix Figures Source Data [file 44318_2024_232_MOESM10_ESM.zip › EV and Appendix Figures/Figure EV4/Figure EV 4M.pdf]

Source data: Figure EV 5A.

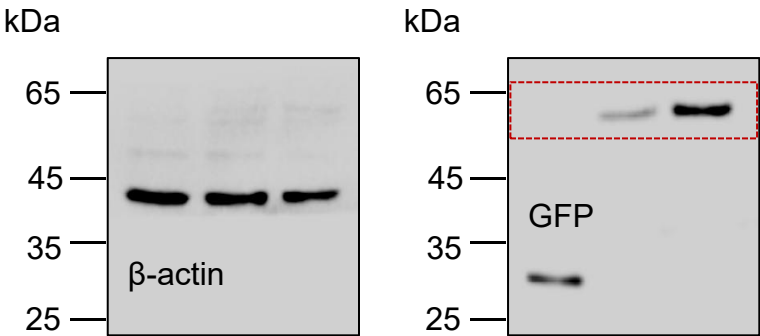

Supplement: Supplementary file 10 — EV and Appendix Figures Source Data [file 44318_2024_232_MOESM10_ESM.zip › EV and Appendix Figures/Figure EV5/Figure EV 5A.pdf]

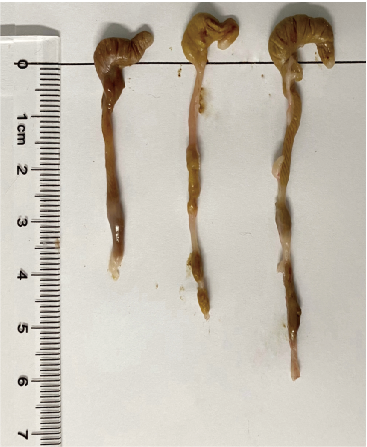

Supplement: Supplementary file 10 — EV and Appendix Figures Source Data [file 44318_2024_232_MOESM10_ESM.zip › EV and Appendix Figures/Figure EV5/Figure EV 5C/5% DSS.png]

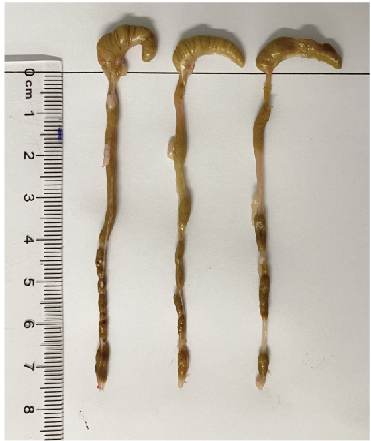

Supplement: Supplementary file 10 — EV and Appendix Figures Source Data [file 44318_2024_232_MOESM10_ESM.zip › EV and Appendix Figures/Figure EV5/Figure EV 5C/water.png]

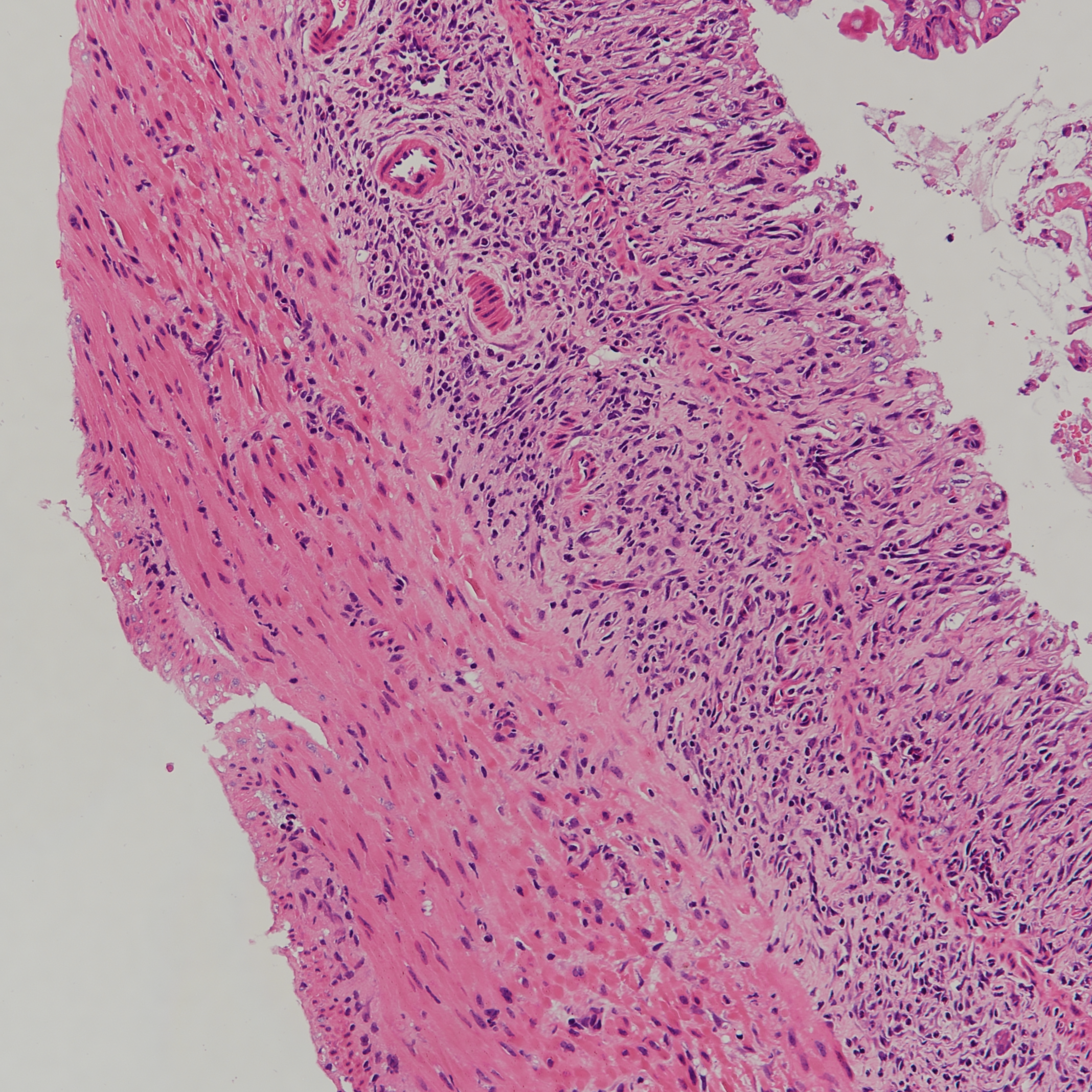

Supplement: Supplementary file 10 — EV and Appendix Figures Source Data [file 44318_2024_232_MOESM10_ESM.zip › EV and Appendix Figures/Figure EV5/Figure EV 5E/5% DSS/0.tif]

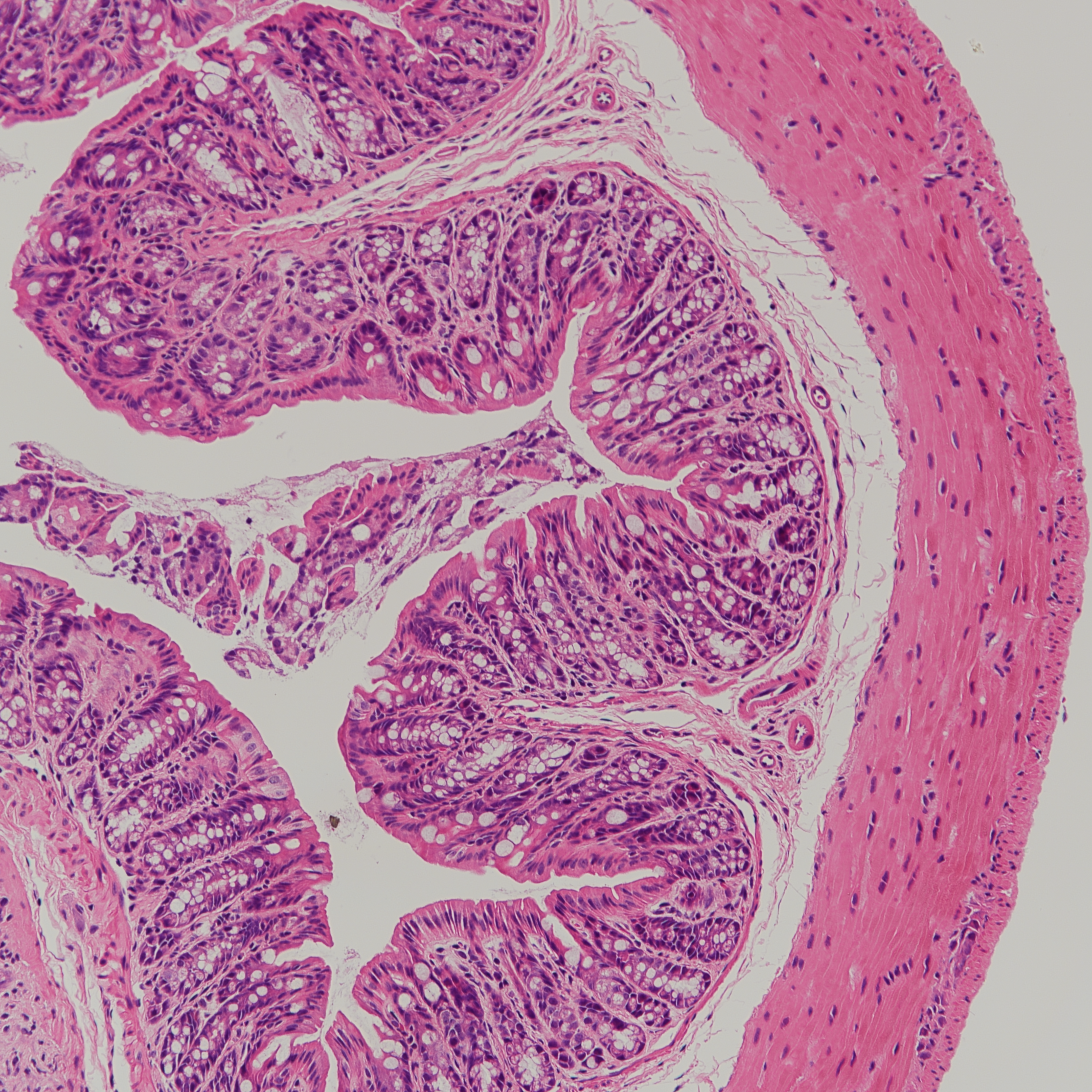

Supplement: Supplementary file 10 — EV and Appendix Figures Source Data [file 44318_2024_232_MOESM10_ESM.zip › EV and Appendix Figures/Figure EV5/Figure EV 5E/5% DSS/100.tif]

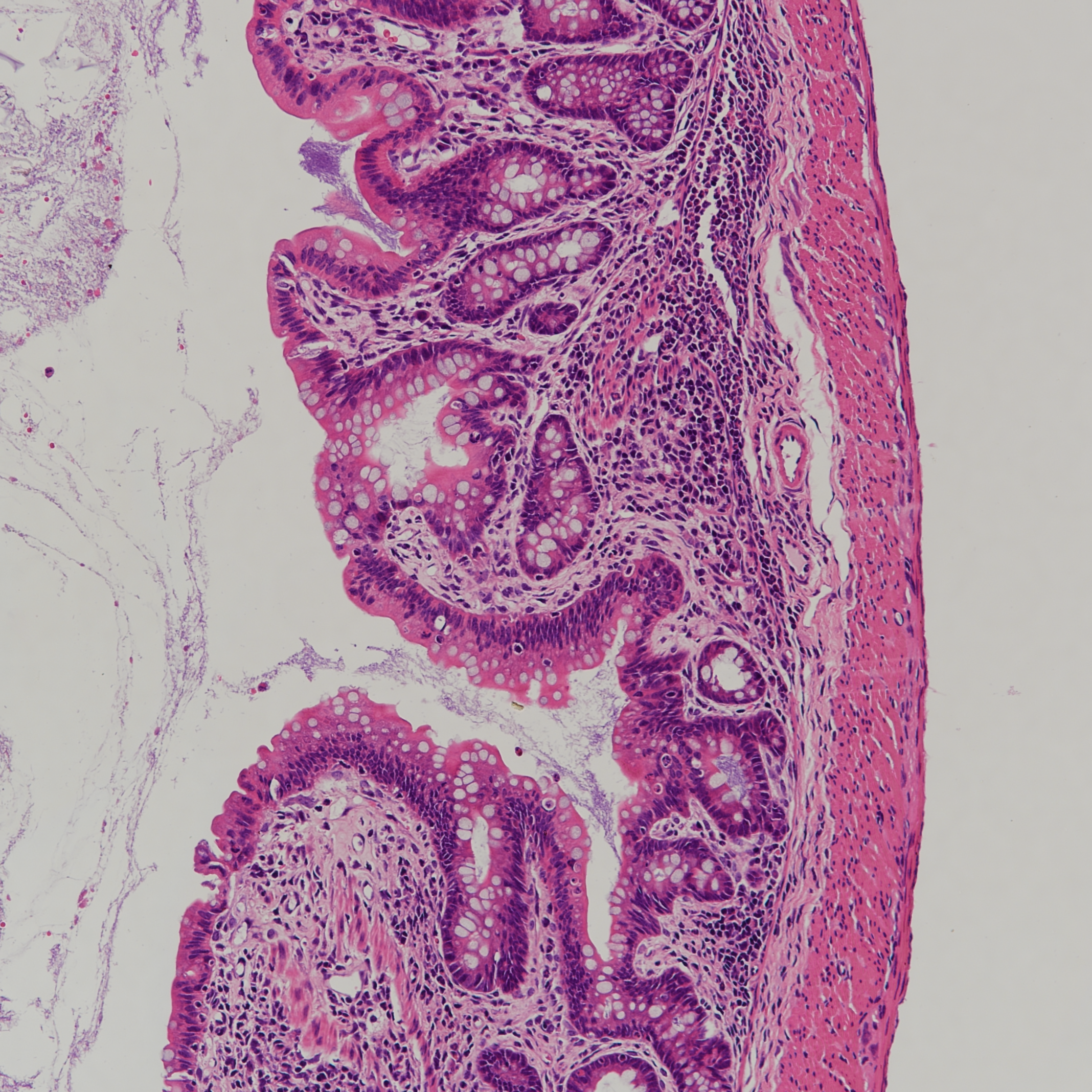

Supplement: Supplementary file 10 — EV and Appendix Figures Source Data [file 44318_2024_232_MOESM10_ESM.zip › EV and Appendix Figures/Figure EV5/Figure EV 5E/5% DSS/25.tif]

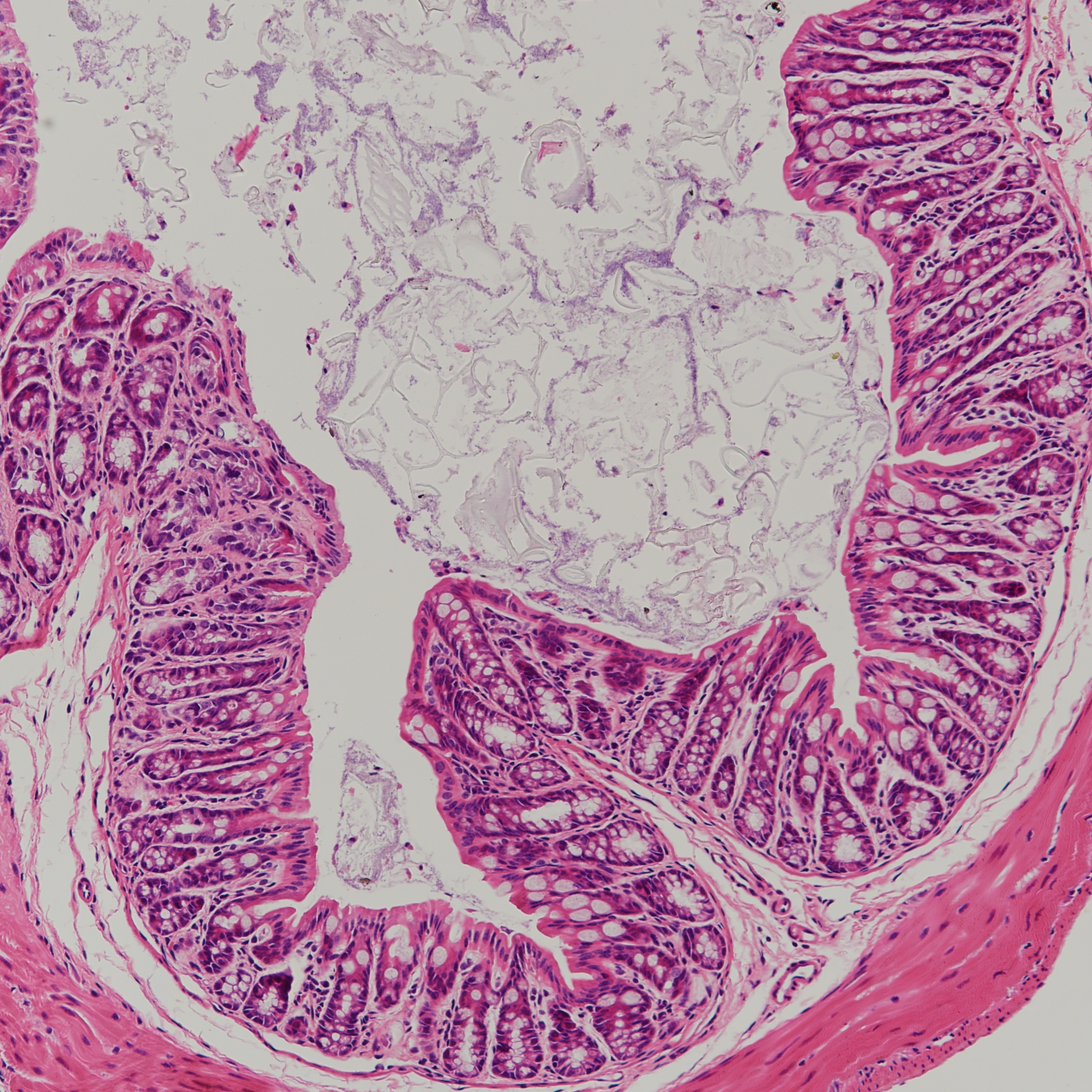

Supplement: Supplementary file 10 — EV and Appendix Figures Source Data [file 44318_2024_232_MOESM10_ESM.zip › EV and Appendix Figures/Figure EV5/Figure EV 5E/Water/0.tif]

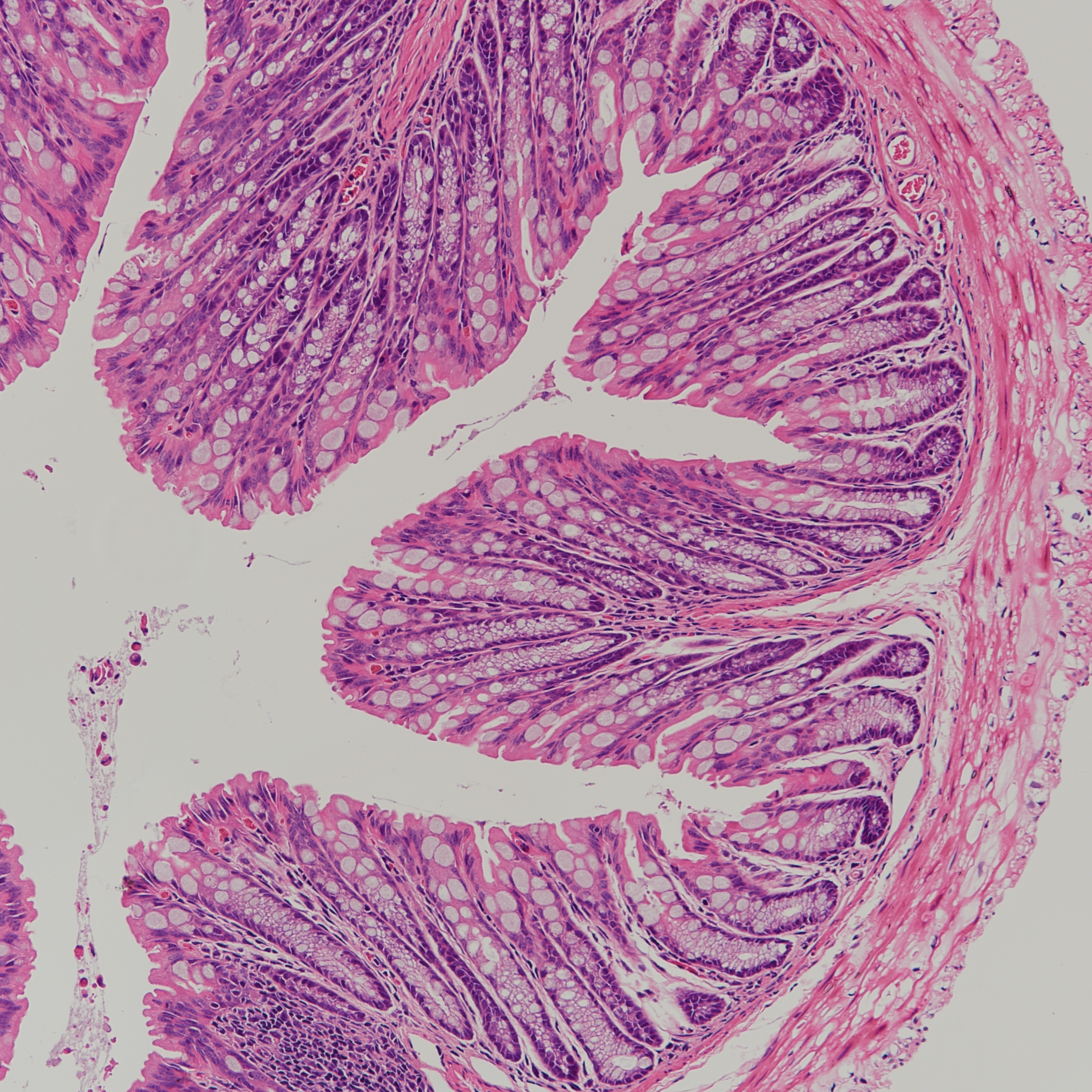

Supplement: Supplementary file 10 — EV and Appendix Figures Source Data [file 44318_2024_232_MOESM10_ESM.zip › EV and Appendix Figures/Figure EV5/Figure EV 5E/Water/100.tif]

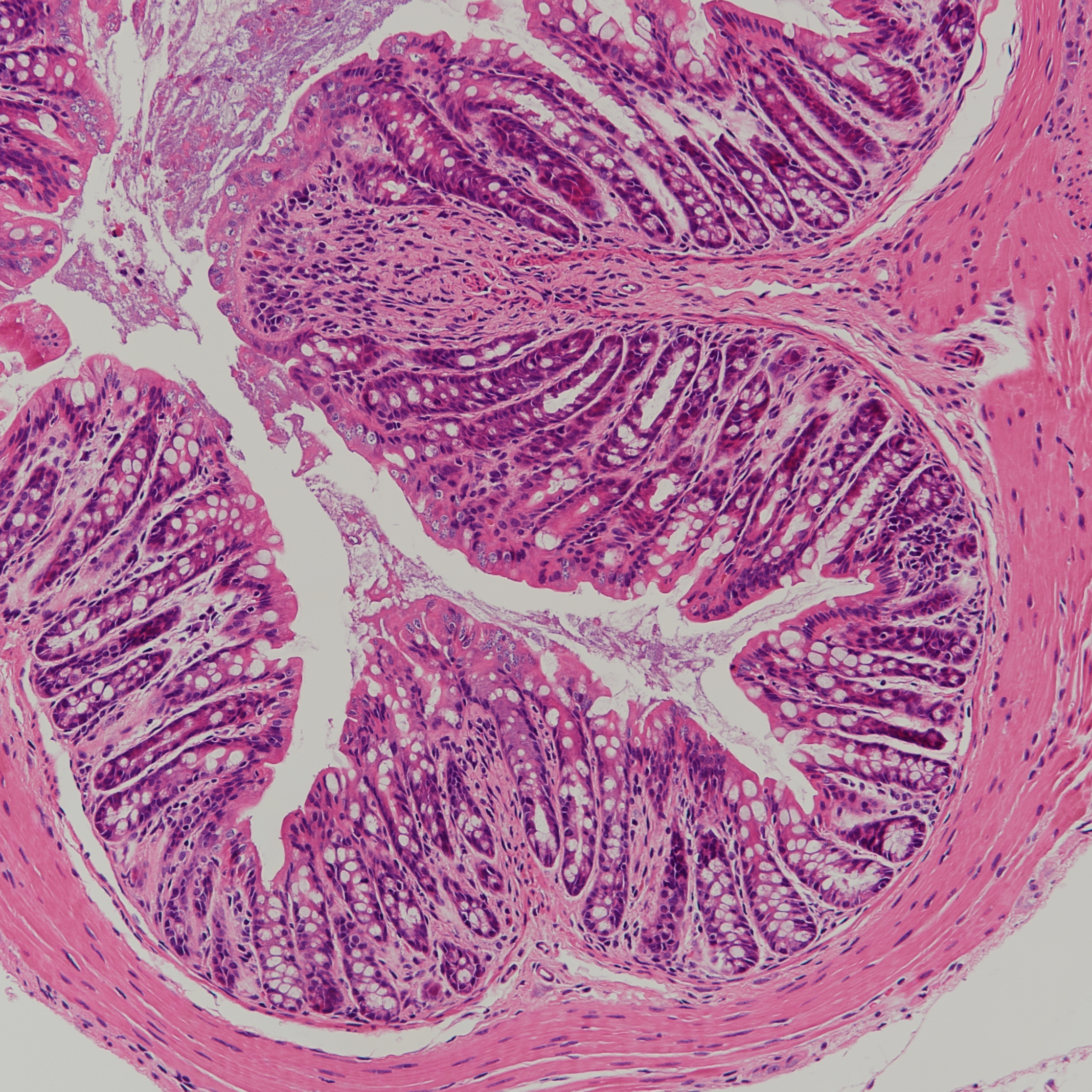

Supplement: Supplementary file 10 — EV and Appendix Figures Source Data [file 44318_2024_232_MOESM10_ESM.zip › EV and Appendix Figures/Figure EV5/Figure EV 5E/Water/25.tif]

Source data: Figure EV 5H.

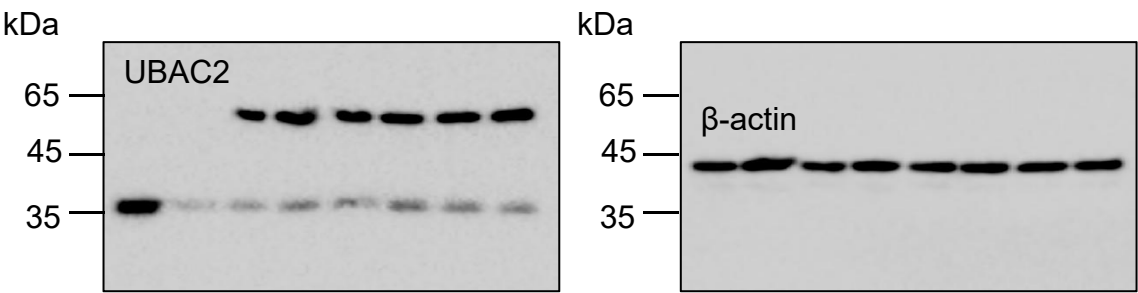

Supplement: Supplementary file 10 — EV and Appendix Figures Source Data [file 44318_2024_232_MOESM10_ESM.zip › EV and Appendix Figures/Figure EV5/Figure EV 5H.pdf]
