# Supplementary material for: ER-phagy restrains inflammatory responses through its receptor UBAC2
Source: EMBO J. 2024 Sep 16;43(21):13. doi: 10.1038/s44318-024-00232-z (PMC11535055; doi:10.1038/s44318-024-00232-z)
Supplement: Supplementary file 11 — Expanded View Figures [file 44318_2024_232_MOESM11_ESM.pdf]

Expanded View Figures

Figure EV1. UBAC2 targets GABARAP vis its LIR.

(**A**) The quantification of UBAC2 protein abundances in the similar samples as Fig. 2A from three independent experiments performed in duplicate. (**B**, **C**) HEK293T cells transfected with plasmids encoding HA-UBAC2 and Flag-tagged ATG8 family members were treated with TG (1 μM) for 1 h (**B**) or EBSS for 3 h (**C**) in the presence Baf A1 (0.2 μM). The lysates were immunoprecipitated with anti-Flag and immunoblotted with anti-HA. (**D**) 293T cells transfected with vectors encoding HA-UBAC2 and Flag-GABARAP were treated with TG (1 μM) for indicated time points in the presence of Baf A1 (0.2 μM), followed by immunoprecipitation with anti-Flag beads and immunoblot analysis with anti-HA. (**E**) 293T cells transfected with vectors encoding HA-UBAC2 and Flag-GABARAP were cultured in EBSS for indicated time points with the existence of Baf A1 (0.2 μM), followed by immunoprecipitation with anti-Flag beads and immunoblot analysis with anti-HA. (**F**, **G**) The quantification of UBAC2 protein abundances in the similar samples as Fig. 2B (**F**) and Fig. 2C (**G**) from three independent experiments performed in duplicate. (**H**) Domain organization of human UBAC2 and alignment of UBAC2 sequences of different species. (**I**, **J**) The quantification of UBAC2 protein abundances in the similar samples as Fig. 2G (**I**) and Fig. 2K (**J**) from three independent experiments performed in duplicate. (**K**) Immunoprecipitation and immunoblot analysis of 293T cells transfected with vectors encoding HA-UBAC2 and Flag-GABARAP as well as its indicated mutants. Data information: For (**B–E**, **K**), one representative experiment out of three was shown. In (**A**, **F**, **G**, **I**, **J**), data are presented as the mean ± SEM of three independent biological experiments. The statistical significance of the difference was analyzed by unpaired two-tailed Student's *t* test, and the *P* values were shown. Source data are available online for this figure.

 © The Author(s)

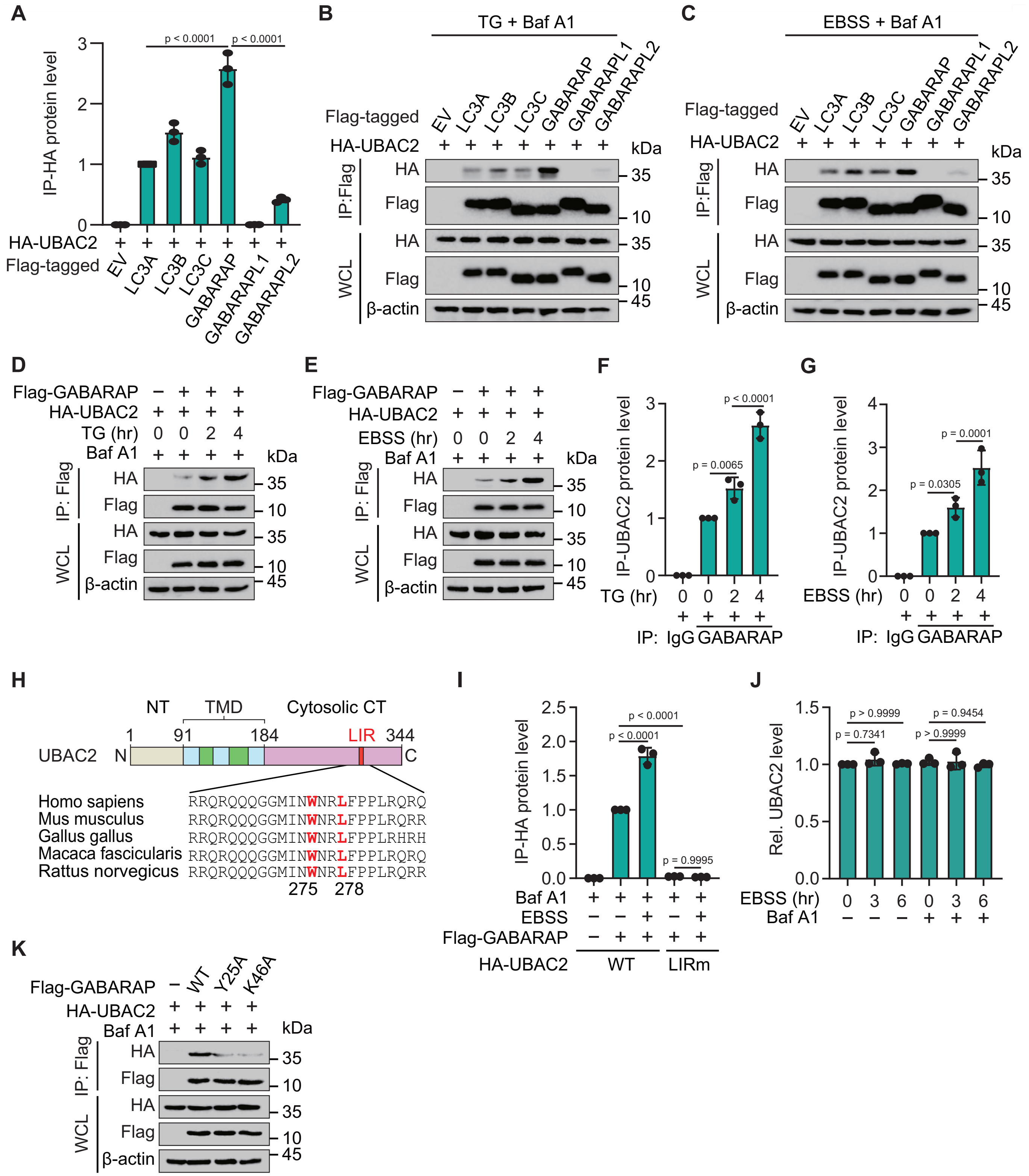
A
p < 0.0001
p < 0.0001
IP-HA protein level
HA-UBAC2
Flag-tagged
EV
LC3A
LC3B
LC3C
GABARAP
GABARAPL1
GABARAPL2
B
TG + Baf A1
C
EBSS + Baf A1
Flag-tagged
HA-UBAC2
kDa
IP:Flag
WCL
HA
Flag
β-actin
D
Flag-GABARAP
HA-UBAC2
TG (hr)
Baf A1
IP: Flag
E
EBSS (hr)
F
p < 0.0001
p = 0.0065
IP-UBAC2 protein level
TG (hr)
IP: IgG GABARAP
G
p = 0.0001
p = 0.0305
EBSS (hr)
H
NT
TMD
Cytosolic CT
UBAC2
N
C
LIR
1
91
184
344
Homo sapiens RRQRQQQGGMINWNRLFPPLRQRQ
Mus musculus RRQRQQQGGMINWNRLFPPLRQRR
Gallus gallus RRQRQQQGGMINWNRLFPPLRHRH
Macaca fascicularis RRQRQQQGGMINWNRLFPPLRQRQ
Rattus norvegicus RRQRQQQGGMINWNRLFPPLRQRR
275 278
I
p < 0.0001
p ≤ 0.0001
p = 0.9995
IP-HA protein level
Baf A1
EBSS
Flag-GABARAP
HA-UBAC2
WT
LIRm
J
p > 0.9999
p = 0.9454
p = 0.7341
p > 0.9999
Rel. UBAC2 level
EBSS (hr)
Baf A1
K
Flag-GABARAP
WT
Y25A
K46A
HA-UBAC2
Baf A1

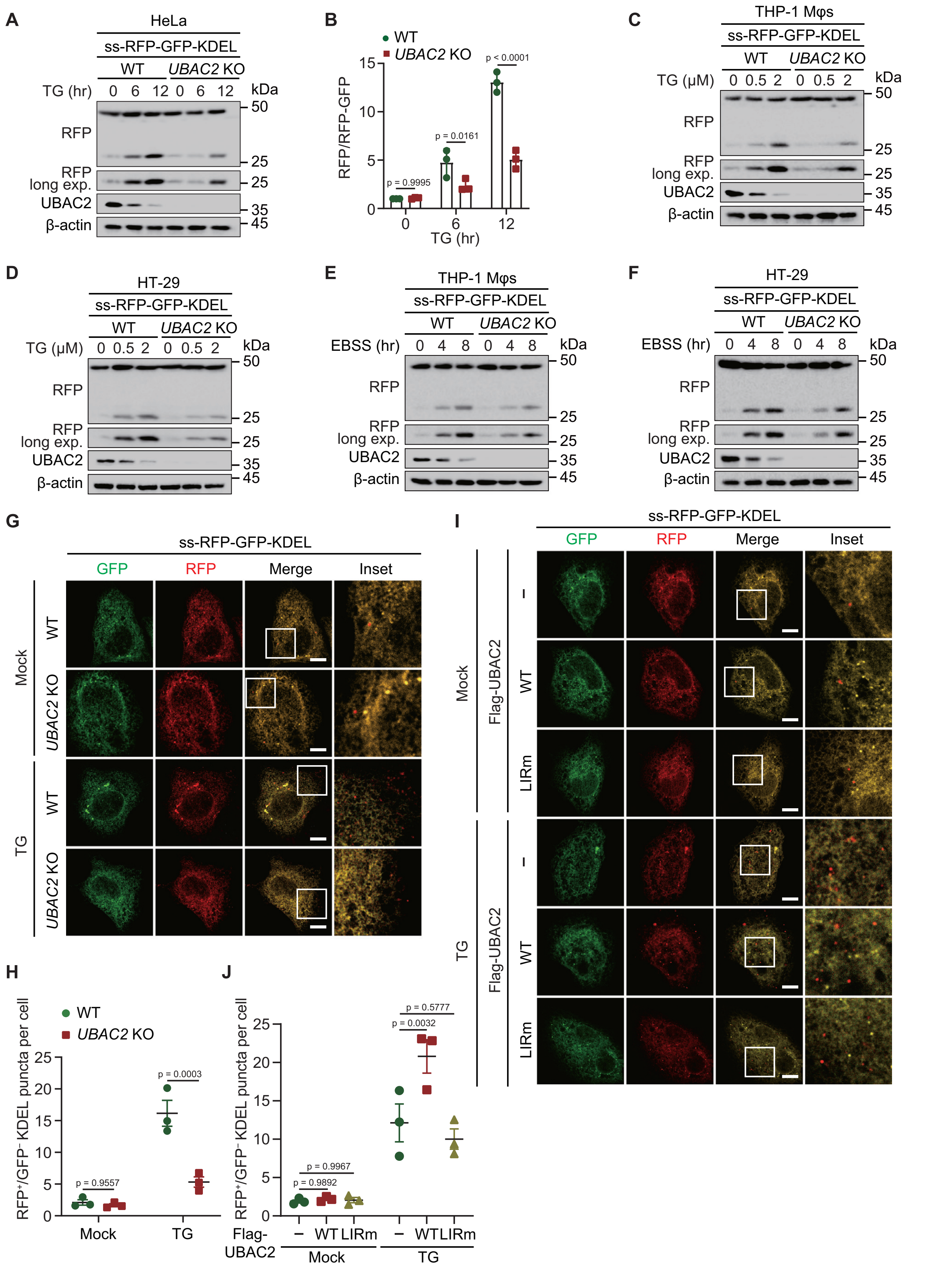
A
HeLa
ss-RFP-GFP-KDEL
WT
UBAC2 KO
TG (hr)
0 6 12 0 6 12
kDa
RFP
RFP long exp.
UBAC2
β-actin
50
25
25
35
45
B
WT
UBAC2 KO
RFP/RFP-GFP
TG (hr)
p = 0.9995
p = 0.0161
p < 0.0001
C
THP-1 Mφs
ss-RFP-GFP-KDEL
WT
UBAC2 KO
TG (μM)
0 0.5 2 0 0.5 2
kDa
RFP
RFP long exp.
UBAC2
β-actin
D
HT-29
ss-RFP-GFP-KDEL
WT
UBAC2 KO
TG (μM)
0 0.5 2 0 0.5 2
E
THP-1 Mφs
ss-RFP-GFP-KDEL
WT
UBAC2 KO
EBSS (hr)
0 4 8 0 4 8
F
HT-29
ss-RFP-GFP-KDEL
WT
UBAC2 KO
EBSS (hr)
0 4 8 0 4 8
G
ss-RFP-GFP-KDEL
GFP
RFP
Merge
Inset
Mock
TG
WT
UBAC2 KO
I
ss-RFP-GFP-KDEL
GFP
RFP
Merge
Inset
Mock
TG
Flag-UBAC2
–
WT
LIRm
H
WT
UBAC2 KO
RFP⁺/GFP⁻ KDEL puncta per cell
p = 0.9557
p = 0.0003
Mock
TG
J
RFP⁺/GFP⁻ KDEL puncta per cell
p = 0.9967
p = 0.9892
p = 0.5777
p = 0.0032
Flag-UBAC2
– WT LIRm – WT LIRm
Mock
TG

 © The Author(s)

Figure EV2. UBAC2 functions as an ER-phagy receptor.

(**A**) HeLa cells stably expressing the ER-phagy reporter transfected with plasmid expressing Flag-UBAC2 were cultured in the presence of doxycycline for 24 h to induce the reporter. Lysates of the cells with TG (1 μM) for indicated time points were harvested for immunoblot analysis. (**B**) The band intensities of RFP and RFP-GFP in the similar samples as (**A**) from three independent experiments performed in duplicate were quantified and the ratios of RFP/RFP-GFP were shown. (**C**, **D**) WT or *UBAC2* knockout (KO) THP-1 Mφs (**C**) or HT-29 cells (**D**) stably expressing the ER-phagy reporter were cultured in the presence of doxycycline (Dox) (200 ng/mL) for 24 h to induce the reporter. Lysates of the cells treated with TG (1 μM) for indicated dosages for 8 h were harvested for immunoblot analysis. (**E**, **F**) WT or *UBAC2* KO Mφs (**E**) or HT-29 cells (**F**) stably expressing the ER-phagy reporter were cultured in the presence of Dox (200 ng/mL) for 24 h to induce the reporter. Lysates of the cells cultured in EBSS for indicated time points were harvested for immunoblot analysis. (**G**) WT or *UBAC2* KO HeLa cells stably expressing the ER-phagy reporter were treated with Dox (200 ng/mL) for 24 h. The cells were treated with or without TG (1 μM) for 8 h and were observed by fluorescence microscopy. Scale bar, 20 μm. (**H**) Quantitative analysis of the similar samples as (**G**) from three biologically independent experiments (20 cells scored per condition per experiment). (**I**) HeLa cells stably expressing the ER-phagy reporter were transfected with plasmids encoding WT or LIRm UBAC2 and treated with Dox (200 ng/mL) for 24 h. The cells were treated with or without TG (1 μM) for 8 h and observed by fluorescence microscopy. Scale bar, 20 μm. (**J**) Quantitative analysis of the similar samples as (**I**) from three biologically independent experiments (20 cells scored per condition per experiment). Data information: For (**A**, **C**–**G**, **I**), one representative experiment out of three was shown. In (**B**, **H**, **J**), data are presented as the mean ± SEM of three independent biological experiments. The statistical significance of the difference was analyzed by unpaired two-tailed Student's *t* test, and the *P* values were shown. Source data are available online for this figure.

© The Author(s)

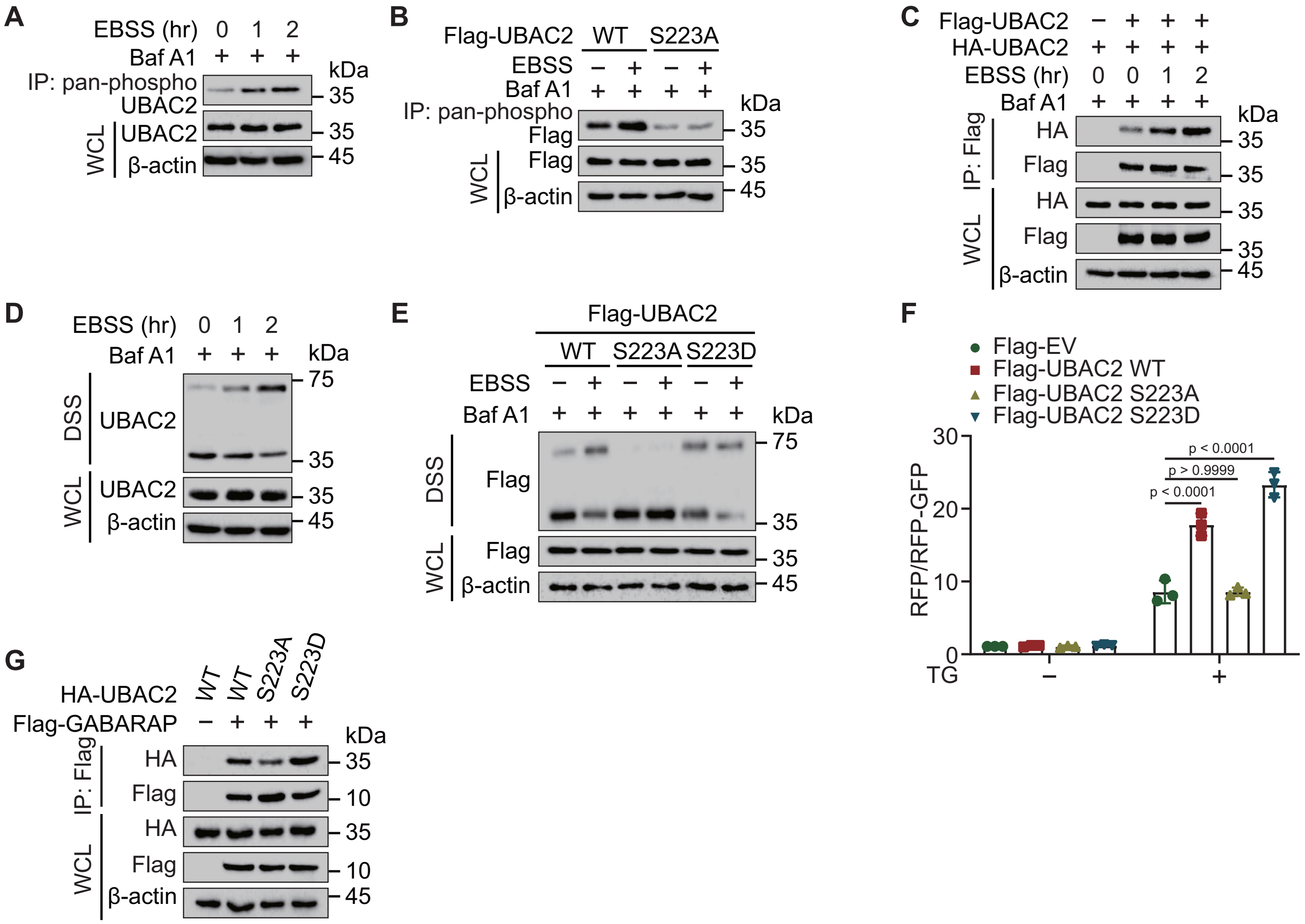

Figure EV3. S223 is critical for the phosphorylation and function of UBAC2.

(**A**) Extracts of HeLa cells cultured with EBSS for indicated time points were immunoprecipitated using phosphoserine/threonine/tyrosine polyclonal antibody and immunoblotted with UBAC2 antibody. (**B**) 293T cells transfected with vector of Flag-UBAC2 or its S223A mutant were cultured within EBSS in the presence of Baf A1 (0.2 μM). The lysates were subjected to coimmunoprecipitation and immunoblot analysis. (**C**) Lysates of 293T cells transfected with plasmids for Flag-UBAC2 and HA-UBAC2, were subjected to immunoprecipitation with anti-Flag and immunoblot analysis with anti-HA. (**D**) Extracts of HeLa cells cultured with EBSS in the presence of Baf A1 (0.2 μM) were treated with 2 mM disuccinimidyl suberate (DSS) cross-linker and analyzed by immunoblotting. (**E**) 293T cells transfected with plasmids encoding WT UBAC2 or its indicated mutants were cultured in EBSS for 3 h with the existence of Baf A1 (0.2 μM). The lysates were treated with 2 mM disuccinimidyl suberate (DSS) cross-linker and analyzed by immunoblotting. (**F**) The band intensities of RFP and RFP-GFP in the similar samples as Fig. 4N from three independent experiments performed in duplicate were quantified and the ratios of RFP/RFP-GFP were shown. (**G**) Coimmunoprecipitation and immunoblot analysis of lysates from 293T cells transfected with vector of Flag-GABARAP and HA-UBAC2 as well as its indicated mutants. Data information: For (**A**–**E**, **G**), one representative experiment out of three was shown. In (**F**), data are presented as the mean ± SEM of three independent biological experiments. The statistical significance of the difference was analyzed by unpaired two-tailed Student's *t* test, and the *P* values were shown. Source data are available online for this figure.

 © The Author(s)

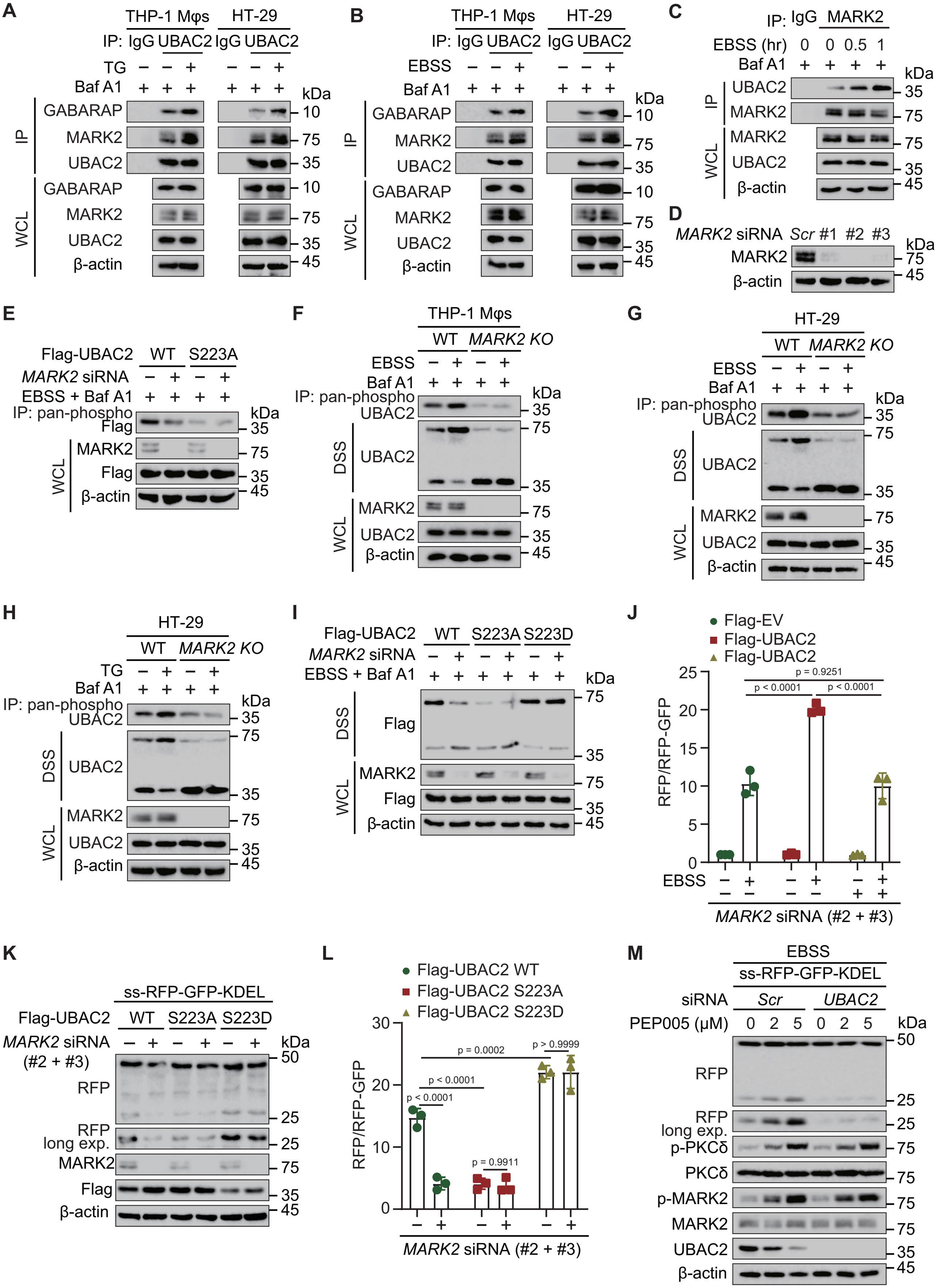
A
THP-1 Mφs
HT-29
IP: IgG UBAC2 IgG UBAC2
TG
Baf A1
kDa
GABARAP
MARK2
UBAC2
β-actin
IP
WCL
B
EBSS
C
IP: IgG MARK2
EBSS (hr) 0 0 0.5 1
D
MARK2 siRNA Scr #1 #2 #3
E
Flag-UBAC2 WT S223A
MARK2 siRNA
EBSS + Baf A1
IP: pan-phospho Flag
Flag
F
WT MARK2 KO
IP: pan-phospho UBAC2
DSS
G
H
I
Flag-UBAC2 WT S223A S223D
J
Flag-EV
Flag-UBAC2
p = 0.9251
p < 0.0001
RFP/RFP-GFP
EBSS
MARK2 siRNA (#2 + #3)
K
ss-RFP-GFP-KDEL
MARK2 siRNA (#2 + #3)
RFP
RFP long exp.
L
Flag-UBAC2 WT
Flag-UBAC2 S223A
Flag-UBAC2 S223D
p = 0.0002
p > 0.9999
p = 0.9911
M
siRNA Scr UBAC2
PEP005 (μM) 0 2 5 0 2 5
p-PKCδ
PKCδ
p-MARK2

Figure EV4. MARK2 is needed for UBAC2-mediated ER-phagy.

(**A**) Lysates of THP-1 Mφs (left) or HT-29 cells (right) treated with TG (1 μM) for 1 h in the presence of Baf A1 (0.2 μM) were subjected to immunoprecipitation and immunoblot analysis. (**B**) Lysates of THP-1 Mφs (left) or HT-29 cells (right) cultured in EBSS for 3 h together with Baf A1 (0.2 μM) treatment were subjected to immunoprecipitation and immunoblot analysis. (**C**) Lysates of HeLa cells treated with EBSS for indicated time points in the presence of Baf A1 (0.2 μM) were subjected to immunoprecipitation and immunoblot analysis. (**D**) Immunoblot analysis of the knockdown efficiency of MARK2 by *MARK2*-specific siRNAs in HeLa cells. (**E**) HeLa cells were transfected with scramble or *MARK2*-specific siRNA and treated with EBSS for 3 h in the presence of Baf A1 (0.2 μM). The lysates were subjected to immunoprecipitation and immunoblot analysis. (**F**) WT or *MARK2* KO THP-1 Mφs were cultured in EBSS for 3 h with the existence of Baf A1 (0.2 μM). The lysates were treated with 2 mM disuccinimidyl suberate (DSS) cross-linker or immunoprecipitated using phosphoserine/threonine/tyrosine polyclonal antibody, and detected by immunoblot. (**G**, **H**) WT or *MARK2* KO HT-29 cells were treated with TG (1 μM) for 1 h (**G**) and cultured in EBSS for 3 h (**H**) in the presence of Baf A1 (0.2 μM). The lysates were treated with 2 mM disuccinimidyl suberate (DSS) cross-linker or immunoprecipitated using phosphoserine/threonine/tyrosine polyclonal antibody, and detected by immunoblot. (**I**) 293T cells were transfected with scramble or *MARK2*-specific siRNA and then transfected with vector of WT or S223A UBAC2. After incubation the cells with EBSS for 3 h in the presence of Baf A1 (0.2 μM), the lysates were harvested for immunoprecipitation and immunoblot analysis. (**J**) The band intensities of RFP and RFP-GFP in the similar samples as Fig. 5K from three independent experiments performed in duplicate were quantified and the ratios of RFP/RFP-GFP were shown. (**K**) HeLa cells stably expressing the ER-phagy reporter were transfected with scramble or *MARK2*-specific siRNA alongside with WT or mutated UBAC2 plasmids. The cells were then cultured in the presence of doxycycline for 24 h to induce the reporter and the protein was harvested for immunoblot analysis. (**L**) The band intensities of RFP and RFP-GFP in the similar samples as (**K**) from three independent experiments performed in duplicate were quantified and the ratios of RFP/RFP-GFP were shown. (**M**) HeLa cells stably expressing the ER-phagy reporter were transfected with scramble or *UBAC2*-specific siRNA and then treated with PEP005 with indicated dosages for 1 h. The cells were cultured in EBSS for 3 h and the lysates were harvested for immunoblot analysis. Data information: For (**A–I**, **K**, **M**), one representative experiment out of three was shown. In (**J**, **L**), data are presented as the mean ± SEM of three independent biological experiments. The statistical significance of the difference was analyzed by unpaired two-tailed Student's *t* test, and the *P* values were shown. Source data are available online for this figure.

© The Author(s)

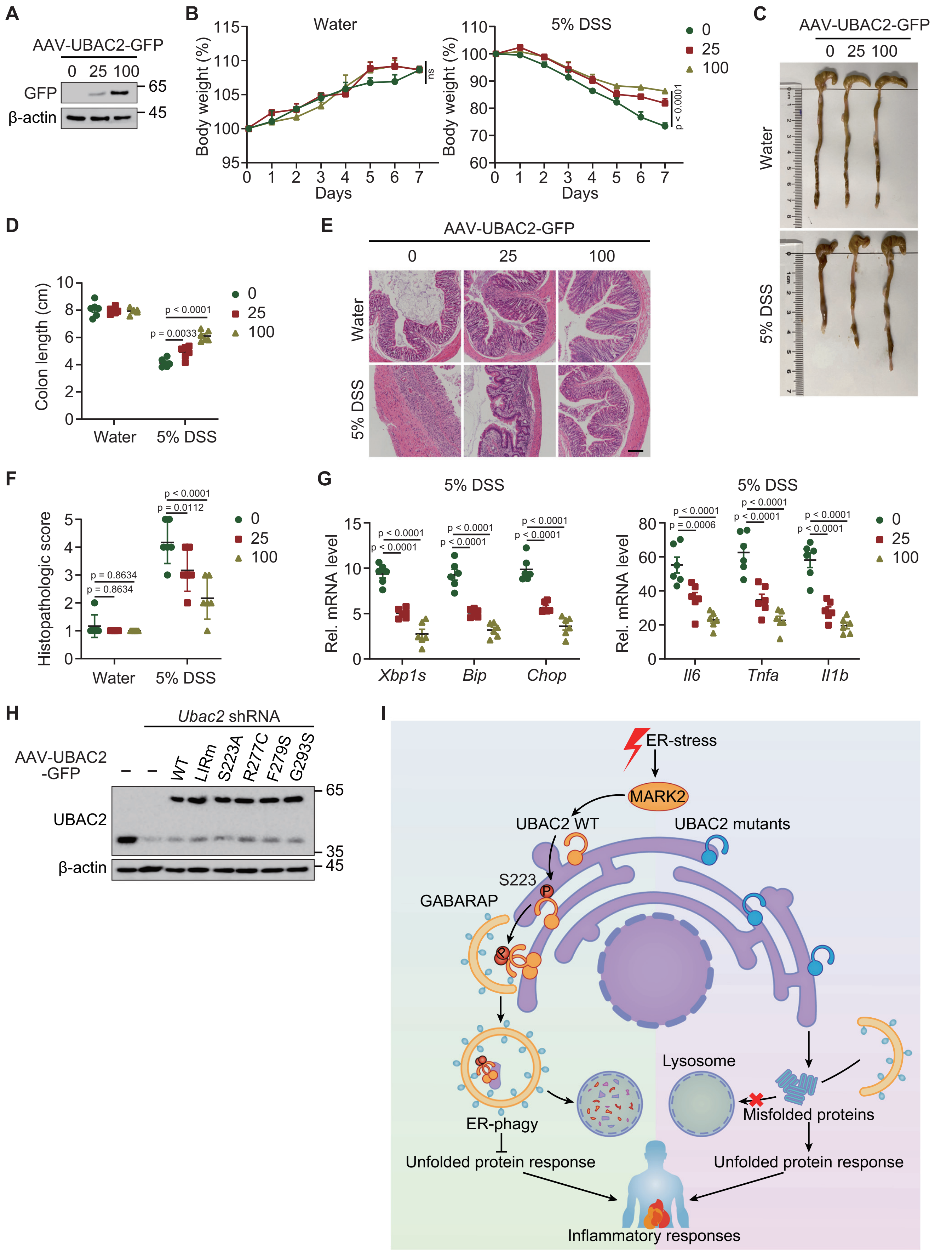
A
AAV-UBAC2-GFP
0 25 100
GFP
β-actin
65
45
B
Water
5% DSS
Body weight (%)
Days
ns
p < 0.0001
0
25
100
C
AAV-UBAC2-GFP
0 25 100
Water
5% DSS
D
Colon length (cm)
Water
5% DSS
p < 0.0001
p = 0.0033
E
AAV-UBAC2-GFP
0
25
100
Water
5% DSS
F
Histopathologic score
p = 0.8634
p = 0.8634
p < 0.0001
p = 0.0112
Water
5% DSS
G
5% DSS
Rel. mRNA level
Xbp1s
Bip
Chop
Il6
Tnfa
Il1b
p < 0.0001
p = 0.0006
H
Ubac2 shRNA
AAV-UBAC2 -GFP
WT
LIRm
S223A
R277C
F279S
G293S
UBAC2
β-actin
65
35
45
I
ER-stress
MARK2
UBAC2 WT
UBAC2 mutants
S223
P
GABARAP
Lysosome
ER-phagy
Misfolded proteins
Unfolded protein response
Unfolded protein response
Inflammatory responses

Figure EV5. UBAC2-mediated ER-phagy inhibits DSS-induced colitis in mice.

(**A**) Representative western blot image of GFP in intestine tissues of DSS-induced mice with increasing dosage of AAV-UBAC2-GFP treatment ($n = 6$ independent biological mice per group). (**B**) Changes in body weight (percentage of original body weight) over time (days) in water- or DSS-treated mice expressing UBAC2 with indicated dose ($n = 6$ independent biological mice per group). (**C**) Representative macroscopic features of colons from water- or DSS-treated mice expressing UBAC2 with indicated dose. (**D**) Colon length of water- or DSS-treated mice expressing UBAC2 with indicated dose ($n = 6$ independent biological mice per group). (**E**, **F**) Representative images (**E**) of H&E staining of colon sections and the histopathologic scores (**F**) from water- or DSS-treated mice expressing UBAC2 with indicated dose. Scale bar, 100 μm. Each experiment was repeated independently three times, and representative results are shown. (**G**) qPCR analysis of ER-stress inducible transcripts (left) and inflammatory genes (right) of intestine homogenates from DSS mice expressing UBAC2 with indicated dose. (**H**) Representative western blot image of UBAC2 in intestine tissues of DSS treated AAV-delivered shRNA *Ubac2* knockdown mice expressing WT and mutated UBAC2 ($n = 6$ independent biological mice per group). (**I**) A proposed working model to illustrate the regulation of UBAC2-directed ER-phagy in suppressing the inflammatory responses. Data information: In (**B**), data are presented as mean ± SEM ($n = 6$ independent biological mice per group). In (**D**, **F**, **G**), data are presented as mean ± SD ($n = 6$ independent biological mice per group). The statistical significance of the difference was analyzed by two-way ANOVA with Bonferroni's multiple comparisons test (**B**) or unpaired two-tailed Student's *t* test (**D**, **F**, **G**), and the *P* values were shown. Source data are available online for this figure.

 © The Author(s)
